# Phylogenetic Reassessment, Taxonomy, and Biogeography of *Codinaea* and Similar Fungi

**DOI:** 10.3390/jof7121097

**Published:** 2021-12-20

**Authors:** Martina Réblová, Miroslav Kolařík, Jana Nekvindová, Kamila Réblová, František Sklenář, Andrew N. Miller, Margarita Hernández-Restrepo

**Affiliations:** 1 Department of Taxonomy, Institute of Botany, The Czech Academy of Sciences, 252 43 Průhonice, Czech Republic; kristina@monoceros.physics.muni.cz (K.R.); frantisek.sklenar@natur.cuni.cz (F.S.); 2 Laboratory of Fungal Genetics and Metabolism, Institute of Microbiology, The Czech Academy of Sciences, 142 20 Prague, Czech Republic; mkolarik@biomed.cas.cz; 3 Department of Clinical Biochemistry and Diagnostics, University Hospital Hradec Králové, 500 05 Hradec Králové, Czech Republic; nekvindova@fnhk.cz; 4 CEITEC—Central European Institute of Technology, Masaryk University, 625 00 Brno, Czech Republic; 5 Illinois Natural History Survey, University of Illinois Urbana-Champaign, Champaign, IL 61820, USA; amiller7@illinois.edu; 6 Westerdijk Fungal Biodiversity Institute, 3508 AD Utrecht, The Netherlands; m.hernandez@wi.knaw.nl

**Keywords:** ancestral inference, appendages, barcodes, GlobalFungi, morphology, molecular systematics, phialidic conidiogenesis, 37 new taxa

## Abstract

The genus *Codinaea* is a phialidic, dematiaceous hyphomycete known for its intriguing morphology and turbulent taxonomic history. This polyphasic study represents a new, comprehensive view on the taxonomy, systematics, and biogeography of *Codinaea* and its relatives. Phylogenetic analyses of three nuclear loci confirmed that *Codinaea* is polyphyletic. The generic concept was emended; it includes four morphotypes that contribute to its morphological complexity. Ancestral inference showed that the evolution of some traits is correlated and that these traits previously used to delimit taxa at the generic level occur in species that were shown to be congeneric. Five lineages of *Codinaea*-like fungi were recognized and introduced as new genera: *Codinaeella*, *Nimesporella*, *Stilbochaeta*, *Tainosphaeriella,* and *Xyladelphia*. Dual DNA barcoding facilitated identification at the species level. *Codinaea* and its segregates thrive on decaying plants, rarely occurring as endophytes or plant pathogens. Environmental ITS sequences indicate that they are common in bulk soil. The geographic distribution found using GlobalFungi database was consistent with known data. Most species are distributed in either the Holarctic realm or tropical geographic regions. The ancestral climatic zone was temperate, followed by transitions to the tropics; these fungi evolved primarily in Eurasia and Americas, with subsequent transitions to Africa and Australasia.

## 1. Introduction

**Figure 1:**
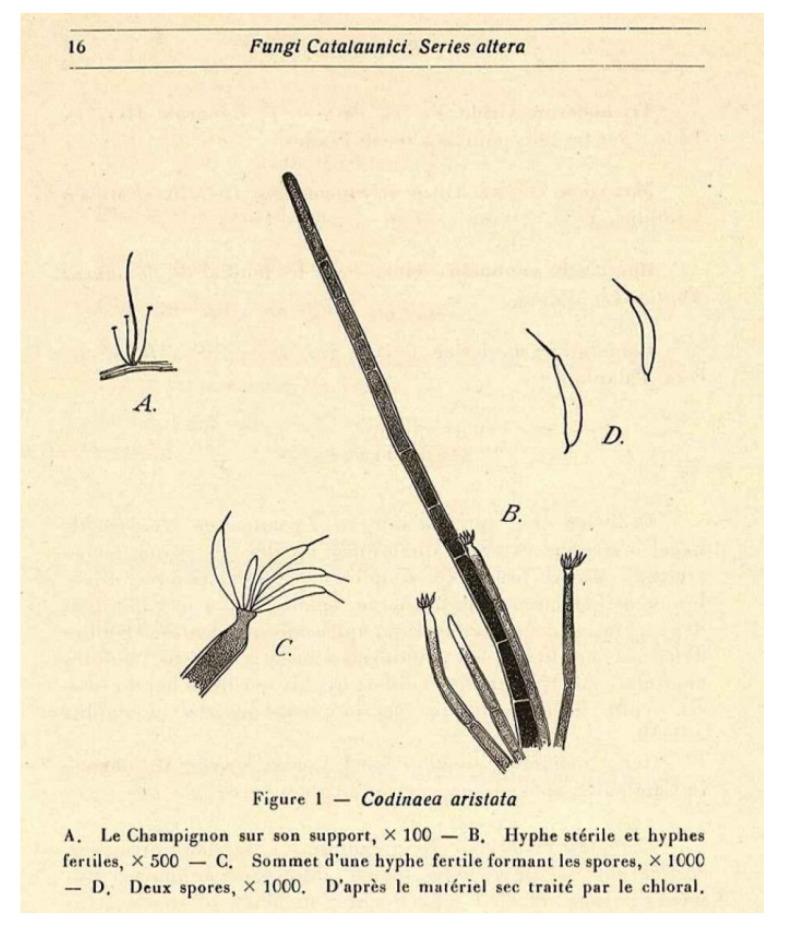
Illustration of a seta, conidiophores, and conidia of *Codinaea aristata* (holotype, Maire 1937).

The genus *Codinaea* [1] is a phialidic dematiaceous hyphomycete and although it was originally clearly defined, its taxonomy has undergone many changes over the years. The genus name was created by Maire [1] as a tribute to his good friend Joaquim Codina i Vinyes, a Catalan physician and mycologist. *Codinaea* was introduced for a single species, *C. aristata*, occurring on a decaying stem of *Rubus* sp. lying on the ground in Spain. According to the original description and illustration, it was characterized by colonies composed of sterile, thick-walled, dark brown, several-septate setae accompanied by shorter, simple, pale brown conidiophores arising with these setae in tufts, terminal phialidic conidiogenous cells abruptly contracted at the apex (indicating the presence of a flared collarette) and hyaline, falcate, aseptate conidia with an indistinct basal scar and a simple setula at both ends (Figure 1). *Codinaea aristata* has since become a taxonomic enigma. This species has not been recorded in the literature since its description. The holotype material could not be traced [2], and neither living cultures nor molecular data are available. Hughes and Kendrick [2] examined the holotype of *Menisporella assamica*, the type species of *Menisporella* [3], originating from India; they concluded that this species is congeneric with *Codinaea* and transferred the genus to its synonymy.

To date, the genus *Codinaea* is composed of 55 species [4] with a worldwide distribution. In addition to the morphotype of *C. aristata*, other morphological characteristics were consequently included, leading to the expansion of the generic concept and the acceptance of species with extralimital morphotypes. Some admitted species lacked setae or setae were present but grew separately among the conidiophores; some species had discrete conidiogenous cells, either lateral on the conidiophore and stalks or on collar-like and nodose hyphae, sometimes conidiophores branched or were synnematous, and the conidia contained septa and sometimes lacked setulae e.g., [2,5,6,7,8,9,10].

The genus *Dictyochaeta* [11,12], typified by *D. fuegiana*, comprises fungi very similar to *Codinaea* in habitat and morphology, but differs mainly in the conidia without setulae, which are more distinctly asymmetrical, and in the arrangement of setae and conidiophores. Arambarri and Cabello [13] proposed a solution to stabilize the generic concept of *Codinaea* by reducing it to synonymy with the rival genus *Dictyochaeta*. They based their study on a numerical taxonomic analysis of 114 species belonging to 13 genera and a set of 28 characters to which they assigned different weights. Arambarri and Cabello [13] emphasized the importance of characters of the conidiophores including their branching pattern, shape of conidiogenous cells, and their position on the conidiophore, whereas the characters of the conidia (septation, symmetry, presence or absence of setulae) and collarettes were down weighed. The morphological similarity between *Codinaea* and *Dictyochaeta*, the multitude of morphotypes that have been associated with them over time, and the lack of knowledge of the phylogenetic significance of some taxonomic characters used in the classification of hyphomycetes have led to a preference for using the broad generic concept of *Dictyochaeta*. Kuthubutheen and Nawawi [14] questioned the mass transfer of *Codinaea* names to *Dictyochaeta* proposed by Arambarri and Cabello [13] without resolving the ambiguity regarding the teleomorphs, which would further complicate the taxonomic status of this already problematic conglomeration. They provided a key to species to distinguish between *Codinaea* and *Dictyochaeta* and proposed four morphological groups of species based on characters of conidia. Réblová and Winka [15] suggested that species with and without setulae should be kept in two separate genera, *Codinaea* and *Dictyochaeta*. This opinion, followed by Seifert et al. [16], was based on phylogenetic analysis of ribosomal DNA of a limited number of representatives of the Chaetosphaeriaceae.

A total of 116 species [4] have been accommodated in *Dictyochaeta* so far. The taxonomic studies of these fungi have relied mainly on morphological criteria, and until recently species of *Dictyochaeta* have rarely been studied using molecular data. In addition, the studies have been hampered by the lack of living cultures and ex-type strains. The first insights into the taxonomy and diversity of *Dictyochaeta* (including *Codinaea*) were provided mainly by Hughes and Kendrick [2], Matsushima [5,17,18], and Kuthubutheen and Nawawi e.g., [14,19,20,21,22,23,24,25]. Hughes and Kendrick [2] made a significant contribution to the taxonomy of this group, demonstrating a rich representation of species native to temperate and subtropical regions of New Zealand. Species described by Matsushima, largely studied in culture, come from various parts of East and Southeast Asia, such as Guadalcanal, Japan, Papua New Guinea, and the Republic of China (Taiwan). Kuthubutheen and Nawawi e.g., [14,19,20,21,22,23,24,25] investigated *Dictyochaeta* in Malaysia, often on submerged plant remnants; they also frequently isolated species in axenic cultures and published their observations in a series of papers in the 1990s. Other authors e.g., [6,26,27] presented a rich diversity of *Dictyochaeta* from the temperate zone of Europe and North America. Recently, several new species were described from China and Thailand e.g., [28,29,30]. Other, mostly single records, originate from different parts of the world e.g., [31,32,33,34,35,36,37,38].

The morphological heterogeneity of *Dictyochaeta*, which has been used as an umbrella name for similar fungi for more than three decades, has been addressed several times. Species with branched conidiophores and lateral phialides were the first to be excluded and segregated into new genera. The existence of extralimital and intermediary species that share features partly of *Chloridium* [39], *Codinaea*, *Dictyochaeta*, *Gonytrichum* [40], and *Menispora* [41], causing the morphology-based generic concept of *Dictyochaeta* to become unsustainable, led to the description of genera such as *Codinaeopsis* [42] and *Dictyochaetopsis* [43]. Morgan-Jones [42] introduced *Codinaeopsis* for *Codinaea gonytrichodes* [6], an intermediate species that is similar to *Gonytrichum* in terms of lateral phialides and branches arising from the collar hyphae. Later, the significance of taxonomic characters of *C. gonytrichodes* was re-evaluated and the species was combined in *Dictyochaeta* [21] and *Dictyochaetopsis* [44]. Following the practice of emphasizing conidiophore and phialide characters over conidial characters at the generic level, Arambarri and Cabello [43] proposed the genus *Dictyochaetopsis* for species of *Dictyochaeta* with branched conidiophores and lateral phialides, however, they accepted species with septate and aseptate conidia with and without setulae. Samuels and Müller [45] described *Striatosphaeria* for ascomycetes with dark brown, furrowed ascospores, unitunicate asci, and perithecial ascomata producing a *Codinaea*-like state in vitro with brown, septate, ellipsoidal-fusiform conidia that were later discovered to form setulae at each end [46].

Recent phylogenetic studies have provided the first DNA sequence data for this group of fungi and demonstrated polyphyly of *Dictyochaeta* e.g., [12,28,29,30,46,47,48,49]. Lin et al. [30] presented an overview of the Chaetosphaeriaceae and applied the name *Dictyochaeta* to five monophyletic clades. Réblová et al. [12] revised the concept of *Dictyochaeta* and showed that the core of the genus is only a small part of a large species complex. *Dictyochaeta* was circumscribed as a monophyletic, morphologically well-defined genus of the Chaetosphaeriaceae. Using morphological and molecular characters, several genera were separated from *Codinaea* and *Dictyochaeta*, such as *Achrochaeta* [12], *Multiguttulispora* [30], *Phialolunulospora* [50], and *Tubulicolla* [12]. Réblová et al. [49] provided phylogenetic evidence that *Dictyochaeta dimorpha* [51] and *D. triseptata* [18] are congeneric with *Multiguttulispora sympodialis* [30], the type species of the genus, and reduced the later species to synonymy of *M. dimorpha*. In addition, *Striatosphaeria* with the *Codinaea*-like anamorph was confirmed as a separate genus, distantly related to other *Codinaea* species with setulae [15].

The fungi that remain classified in *Codinaea* and *Dictyochaeta* are dematiaceous phialidic hyphomycetes that, based on literature and herbarium records, are globally distributed in major centers of diversity in Australasia, Holarctic realm, and the tropics, and thrive on decaying plant material such as bark, wood, wooden fruits, bamboo culms, palm fronds, fallen leaves and petioles in freshwater and terrestrial environments e.g., [2,5,6,20,21,22,23,24,25,26,27,28,29,30,31,32,33,34,35,36,37,38,52]. They also occur as endophytes in living plants e.g., [53], or plant pathogens [54,55,56]. Seldom, they have been linked with their sexual counterparts classified in the genus *Chaetosphaeria* [2,57].

Although recent molecular studies of members of *Codinaea* and *Dictyochaeta* have improved our understanding of this heterogeneous assemblage, the systematic placement of most of their representatives remains unexplored. The unknown placement of *C. aristata*, presumably in one of the five *Dictyochaeta* clades in the Chaetosphaeriaceae [30], remains a pressing issue. Therefore, we studied a large set of strains including all available ex-type strains of *Codinaea* and similar fungi. Based on our preliminary multilocus analysis, these taxa formed several monophyletic groups that seem to correlate with morphological data. The present study aims to clarify the delimitation of *Codinaea*, its relationship with *Dictyochaeta* and other unnamed clades containing *Codinaea*-like and *Dictyochaeta*-like fungi using phylogenetic analyses for three ribosomal and protein-coding loci combined with morphological and RNA structural data and data on geographic range. We evaluate their intraspecific and interspecific relationships and the diagnostic value of taxonomic characters used in the classification of these fungi. The performance of used loci as barcoding markers is evaluated. We also attempt to assess their global geographic distribution and ecology. To date, research on geographical distribution of fungi has relied on traditional approaches (i.e., field sampling, cultivation) and public nucleotide sequences databases, such as NCBI GenBank [58] and UNITE [59]. However, most of the barcode sequences generated to date have come from massively parallel sequencing technologies. The data have been stored in various public repositories, which does not allow easy retrieval of data from multiple studies. This gap has been filled by creating the GlobalFungi ITS barcode database [60,61], an important tool for studying fungi, especially those that are poorly known due to their slow growth and rarity. In this context, our research is a pioneering study evaluating the applicability of the GlobalFungi database in exploring geographic distribution at a fine species level scale. In addition, we perform reconstruction of ancestral states of morphological characters and ancestral climate zones and areas of species distribution of *Codinaea* and similar fungi.

## 2. Materials and Methods

### 2.1. Fungal Strains and Morphology

Material for this study was collected in the temperate regions in the Northern and Southern Hemispheres in lowland and mountain forests in Europe (Czech Republic, France and The Netherlands), North America (USA), New Zealand, and in the tropical region in the evergreen rainforest in Puerto Rico. Other specimens were obtained from the Canadian National Mycological Herbarium, Ottawa, Canada (DAOM) and the Kew herbarium, Kew, United Kingdom (K and IMI). Type material designated in this study and other specimens, as dried voucher specimens and dried cultures, were deposited at Herbarium of the Institute of Botany, Průhonice, Czech Republic (PRA), New Zealand Fungarium, Auckland, New Zealand (PDD) and Westerdijk Fungal Biodiversity Institute, Utrecht, The Netherlands (CBS). Axenic cultures were derived from freshly collected specimens and deposited at CBS and the International Collection of Microorganisms from Plants, Auckland, New Zealand (ICMP). Other living strains were obtained from American Type Culture Collection, Rockville, Maryland, USA (ATCC), BCCM/MUCL Agro-food & Environmental Fungal Collection, Louvain, Belgium (MUCL), CABI-IMI Culture Collection, Egham, United Kingdom (IMI), CBS, ICMP, and Texas Therapeutics Institute, Houston, Texas, USA (TTI). Fungal novelties were registered in MycoBank.

The descriptions of fungi given in this study are based on morphological characters obtained from our observations on natural substrates and in culture. Fresh and herbarium material were examined with an Olympus SZX12 dissecting microscope (Olympus America, Inc., Melville, NY, USA); ascomata, conidiophores and conidia were rehydrated with tap water. Microscopic preparations containing sections of ascomatal wall, asci, ascospores, paraphyses, conidiophores with conidiogenous cells and conidia were mounted in 90% lactic acid, water or Melzer’s reagent. Measurements were taken in Melzer’s reagent. Means ± standard deviation (SD) based on a minimum of 20–25 measurements were given for sizes of asci, ascospores, and conidia. Microscopic observations were performed using an Olympus BX51 compound microscope with differential interference contrast (DIC) and phase-contrast (PC) illumination. An Olympus DP70 camera operated by Imaging Software Cell^D (Olympus) was used to capture images. Macroscopic images of colonies were documented using a Canon EOS 77D digital camera with Canon EF 100 mm f/2.8 L Macro IS USM objective (Canon Europe Ltd., Middlesex, UK) with daylight spectrum 5500 K 16 W LED lights. All images were processed with Adobe Photoshop CS6 (Adobe Systems, San Jose, CA, USA).

Fungi from fresh collections were isolated into axenic cultures. Slimy conidial masses and the content of ascomata were removed with the tip of a sterile needle and placed on water agar (WA) (1000 mL distilled water, agar 10 g, Oxoid Limited, Hampshire, UK) and incubated for 2–5 days. Germinating spores were transferred to a new medium, i.e., single and multiple ascospore and conidial isolates were obtained with the aid of a single-spore isolator (Meopta, Přerov, Czech Republic) and incubated on WA or Modified Leonian’s agar (MLA) [62] at a temperature of 20–25 °C. Species were cultured on several different nutrient media for observation of microscopic characters, comparison of colony characters, and evaluation of pigment production and growth optimum. Fungi were inoculated on cornmeal dextrose agar (CMD) (Oxoid Limited; 2% dextrose), MLA, oatmeal agar (OA) and potato-carrot agar (PCA) [63]. Characteristics of colonies were based on 4-week-old cultures grown in darkness at 22–23 °C. Strains were also inoculated on cornmeal agar (CMA) with sterile stems of *Urtica dioica* and synthetic nutrient agar (SNA) with pine needles [63] to induce sporulation and growth of setae.

### 2.2. DNA Extraction, PCR Amplification, and Sequencing

Total genomic DNA was extracted from mycelium removed from 3–4-week-old cultures grown on MLA using the DNeasy^®^ UltraClean^®^ Microbial Kit (Qiagen GmbH, Hilden, Germany) following the manufacturer’s protocol for filamentous fungi. PCR amplifications were carried out in 25 μL volume reactions using a Q5 High Fidelity DNA polymerase kit (New England Biolabs Inc., Hitchin, UK) according to the manufacturer’s protocol.

Three regions were amplified with the following primers: V9G/LR8 primer pair [64,65] was used for the internal transcribed spacer (ITS1-5.8S-ITS2) (ITS) of the nuclear rRNA cistron and the nuclear large subunit 28S rDNA gene (28S) (approximately the first half, 1800 base pairs at the 5′-end), and EF1-983F/EF1-2218R [66] for the intermediate section of the coding region of the translation elongation factor 1-alpha (*tef1-α*).

PCR was carried out in a BioRad C1000 thermal cycler (Bio-Rad Laboratories Inc., Hercules, CA, USA) as follows: (ITS-28S) 98 °C for 30 s; 40 cycles of denaturation (98 °C for 10 s), annealing (62 °C for 30 s) and elongation (72 °C for 90 s) and a final extension step at 72 °C for 5 min; (*tef1-α*) 98 °C for 30 s; 40 cycles of denaturation (98 °C for 10 s), annealing (57 °C for 10 s) and elongation (72 °C for 60 s) and a final extension step at 72 °C for 2 min. Amplicons were purified from agarose gels using a NucleoSpin^®^ Gel and PCR Clean-up Kit (Macherey-Nagel GmbH & Co. KG, Düren, Germany) following the manufacturer’s instructions, with an elution volume of 25 μL. The DNA concentration was assessed fluorimetrically using Quant-iT^TM^ PicoGreen^TM^ dsDNA Assay Kit and Qubit fluorometer (Invitrogen/Thermo Fisher Scientific Inc., Waltham, MA, USA) to assure the required sequencing concentrations adjusted for the length of amplicons/number of reads required.

The obtained amplicons were sequenced in both directions using the PCR and nested primers ITS5, ITS4, JS1, JS7, JS8, and LR7 [65,67,68,69]. Automated sequencing was carried out by Eurofins GATC Biotech Sequencing Service (Cologne, Germany), the Roy J. Carver Biotechnology Center at the University of Illinois Urbana-Champaign (Champaign, IL, USA), and Westerdijk Fungal Biodiversity Institute (Utrecht, The Netherlands). Raw sequence data were analyzed using Sequencher v.5.4.6 (Gene Codes Corp., Ann Arbor, MI, USA).

### 2.3. Gene Markers, Alignments and Phylogenetic Analyses

Representatives of the Chaetosphaeriaceae have traditionally been studied using ITS and 28S and more recently also *tef1-α* markers. The same loci were analyzed in the present study. Strains, their sources, and the GenBank accession numbers of sequences of ITS, 28S and *tef1-α* determined in this study are listed in Table 1. Accession numbers for sequences retrieved from GenBank and published in other studies [12,15,28,29,30,47,48,49,50,70,71,72,73,74,75,76,77,78,79,80,81,82,83,84,85,86] are listed in Table 1 and the Supplementary File: Table S1.

Sequences were aligned in Mafft v.7.487 [87] implemented in the CIPRES Science Gateway v.3.3 [88,89] and manually corrected in Bioedit v.7.1.8 [90], if necessary. Consensus secondary structure (2D) models for the ITS1 and ITS2 of *Codinaea* and related fungi and other members of the Chaetosphaeriaceae (see Section 2.4.) were used to improve the alignment by comparing nucleotides at homologous positions (in helices and loops). A predicted 2D model of the 28S of *Saccharomyces cerevisiae* [91] was used to improve the alignment of this gene. The three individual datasets, for which we assumed rate heterogeneity, were evaluated using MrModeltest v.2.4 [92] to find the best partitioning scheme and to select best-fit models under Akaike information criteria. The GTR+I+G model was selected for all partitions. The concatenated dataset of three loci (ITS, 28S and *tef1-α*) contained 181 sequences. The final alignment (deposited in TreeBASE 29053) was subjected to phylogenetic analysis. It consisted of 3424 characters including gaps (ITS = 640 characters; 28S = 1784; *tef1-α* = 1000) and 1682 unique character sites (RAxML). We analyzed the entire dataset with the exception of 90 nucleotides (nt) at the 5′-end of 28S, because most sequences retrieved from GenBank were incomplete. *Tracylla aristata* and *T. eucalypti* (Tracyllales) served as outgroup.

Phylogenetic reconstructions were performed using Bayesian Inference (BI) and Maximum Likelihood (ML) analyses through the CIPRES Science Gateway v.3.3. The ML analysis was conducted with RAxML-HPC v.8.2.12 [93] with a GTRCAT approximation. Nodal support was determined by non-parametric bootstrapping (BS) with 1000 replicates. BI analysis was performed in a likelihood framework as implemented in MrBayes v.3.2.7 [94]. Two Bayesian searches were performed using default parameters. The B-MCMCMC (Bayesian-Metropolis-coupled Markov chain Monte Carlo) analyses lasted until the average standard deviation of split frequencies was below 0.01 with trees saved every 1000 generations with burn-in 25%. The BI and ML phylogenetic trees were compared visually for topological conflict among supported clades.

For ITS and *tef1-α* markers, histograms of intraspecific and interspecies distances of *Codinaea*, *Codinaeella,* and *Stilbochaeta* were generated to illustrate the degree of overlap (i.e., barcoding gap) for both genes. Matrix of pairwise distances was computed with MEGAX [95] using the Kimura two parameters (K2P) model [96], and the histogram was plotted in GraphPrism software v.7.03 (Graphpad Software Inc., LaJolla, CA, USA) using a bin size of 0.001 and 0.0005 (only ITS data in *Codinaeella*). The estimates of evolutionary divergence between ITS rDNA and *tef1-α* sequences of members of *Codinaea*, *Codinaeella,* and *Stilbochaeta* are provided in the Supplementary File: Table S2.

### 2.4. Prediction of 2D Structure Models ITS2

Multiple sequence alignment of homologous RNA sequences can be improved using secondary (2D) structure information by comparing positions in helices and loops. We employed PPfold program v.3.0 [97], which uses an explicit evolutionary model and a probabilistic model of structures and takes as input multiple sequence alignment of related RNA sequences in order to build consensus ITS2 2D structure models. Final 2D models were adjusted with UNAfold web server [98,99] which employs mFold program [100] to improve the initial multiple sequence alignment of members of the Chaetosphaeriaceae. The 2D models were visualized using VARNA: Visualization Applet for RNA program [101]. We also employed CBC analyzer program [102], which calculates a number of CBC (Compensatory Base Change) changes for input sequences and their 2D structures.

### 2.5. Identification of Base-Pair Changes in 2D Structures of ITS

We performed an in-depth analysis of the ITS2 molecule of *Codinaea* and two newly proposed segregate genera *Codinaeella* and *Stilbochaeta* to further explore inter- and intraspecific variability of their species at the structural level. All three genera have good species representation and available DNA sequence data. The evolutionary processes at the RNA structural level, responsible for preserving the RNA helix structure (described below), are widely accepted as a basis for the CBC species concept used to delimit biological species [103,104,105,106,107,108]. 

There are three different types of substitutions in helixes. First event represents compensatory base change (CBC), which perfectly maintains 2D structure of helices, for instance G=C pair can change to C=G, A-U or U-A. These canonical base pairs (bp) are isosterical [109], i.e., they can be substituted for each other within stems without causing structural perturbations. Second event is hemi-compensatory base change (hCBC), where standard canonical pairs A-U and G=C change to near-isosteric “wobble pairs” G/U, A+/C (adenine is protonated in this pair), or vice versa. The wobble pairs are similar, retaining the base pairing in RNA molecules, but are not spatially identical to C=G, A-U, so they induce minor perturbation in the helix structure [109]. Third event is non-compensatory base change (non-CBC) that involves the replacement of a canonical pair or a wobble pair with any non-canonical pair and produces the largest structural perturbation into the helix structure. Incorporation of non-WC pairs (WC = standard Watson-Crick base pairs or canonical base pairs) into helix does not mean that helix structure disrupts, rather the tertiary structure of the helix is changed, which can subsequently modulate its function.

### 2.6. Ancestral State Reconstruction

Based on the groups that were determined in the current phylogenetic tree, we have selected 16 well-supported monophyletic lineages that represent *Codinaea*, its segregates and other morphologically similar taxa, and we have scored five characteristics traditionally used to separate hyphomycetous genera. Software package RASP v.4.2 [110] was used for the reconstruction of ancestral states of morphological characters and ancestral areas of species distribution. The phylogenetic trees used as input were calculated in MrBayes v.3.2.7 [94] using partitioned analysis of ITS, 28S, and *tef1-α*; suitable models of evolution for each locus were selected in jModelTest v.2.1.7 [111].

The reconstruction of morphological states was performed using Multistate function in Bayestraits with the following options: mcmc; number of iterations 5,050,000; sample 10,000; burn-in 50,000. We tested five morphological characters with the following states: I. Setae: (a) absent (b) isolated (c) in tufts with conidiophores; II. Conidiophores: (a) branched, with sterile extension (b) branched without sterile extension (c) unbranched, with sterile extension (d) unbranched without sterile extension; III. Phialides: (a) terminal, integrated, on main stalk or branches, discrete, lateral, sessile or on stalk (b) only terminal, integrated, on main stalk or branches (c) only discrete, lateral, sessile or on stalk; IV. Conidial features: (a) setulae and septa both present (b) setulae present, septa absent (c) setulae absent, septa present (d) setulae and septa both absent V. Conidial shape (a) falcate, lunate, narrowly fusiform, curved (b) other (ellipsoidal, ellipsoidal-fusiform, oblong, reniform, globose, pyriform, ellipsoidal, papillate, obovoid, ellipsoidal-clavate, cylindrical-clavate). 

Bayesian Binary MCMC (Markov chain Monte Carlo) (BBM) method was used for the reconstruction of ancestral areas with the following settings: number of cycles 1,000,000; number of chains 10; frequent of samples 100; discard samples 100; temperature: 0.1; state frequencies: estimated (F81); maximum number of areas: 3. The analysis was split into two parts. In the first part, the distribution of species in climatic zones was tested. The species were split into three groups, the first one occurring only in tropical regions, the other in subtropical and temperate regions and the third group consisted of species occurring in all of these climate zones. In the second part, we classified the species into four geographic areas (Eurasia, Australasia, America, and Africa). The allocation of areas to species was based on published distribution of each species and also on information obtained from GlobalFungi database [60] using Sequence search (Exact hit) function.

### 2.7. Phylogeny of Environmental Sequences and Biogeography

The biogeographic analysis focused on *Codinaea* and two main segregate genera *Codinaeella* (*Ca*.) and *Stilbochaeta*, which are species rich with available ITS sequence data. Basically, we followed the workflow of Réblová et al. [86]. We used GlobalFungi database (dataset release 3), which contains 36,684 samples from 367 studies, 582,264,149 unique ITS1 and 526,638,147 unique ITS2 sequences. Because the GlobalFungi database contains separated ITS1 and ITS2 sequences, these two regions were analyzed separately. First, we determined the interspecies genetic distances for each ITS spacer. The detailed comparison of ITS sequences among individual species showed that for some species, only one of the ITS spacers is distinctive and that only the threshold of the full sequence identity should be used to link known species with metabarcoding data unequivocally. In ITS1, two species of *Codinaeella*, *Ca. minuta* and *Ca. filamentosa*, have identical sequences. The variability between *Stilbochaeta aquatica* and *S. submersa* and that between *Ca. lambertiae* and *Ca. lutea* was in a single position (similarity of 99.4%). For ITS2, all species can be differentiated; only one bp difference (99.4%) was found between *Ca. lutea* and *Ca. yunnanensis*, *Ca. minuta* and *Ca. filamentosa* differ in two positions (98.7%). Therefore, we subjected all ITS1 and ITS2 haplotypes from our study (Table 1) to BLASTn [112] similarity search in GlobalFungi with the 100% similarity threshold and rule of the full length sequence coverage. Because we found that ITS spacers deposited in GlobalFungi, originally extracted using ITSx extractor [113], do not always represent full-length sequences (usually 1–2 bp at the 5′ end of ITS2 are missing), we used ITS2 sequences trimmed accordingly. Sites were classified into climatic regions according to Köppen-Geiger [114] and plotted on map using Tableau Desktop v.2021.3 (Tableau Software, Seattle, WA, USA). Primary data about occurrence and respective metadata, e.g., location, substrate, biome, pH and climatic data such as the mean annual precipitation (MAP) and mean annual temperature (MAT) were obtained for each taxon from the GlobalFungi database (Supplementary File: Table S3).

## 3. Results

### 3.1. Phylogenetic and Barcode Analyses

**Figure 2:**
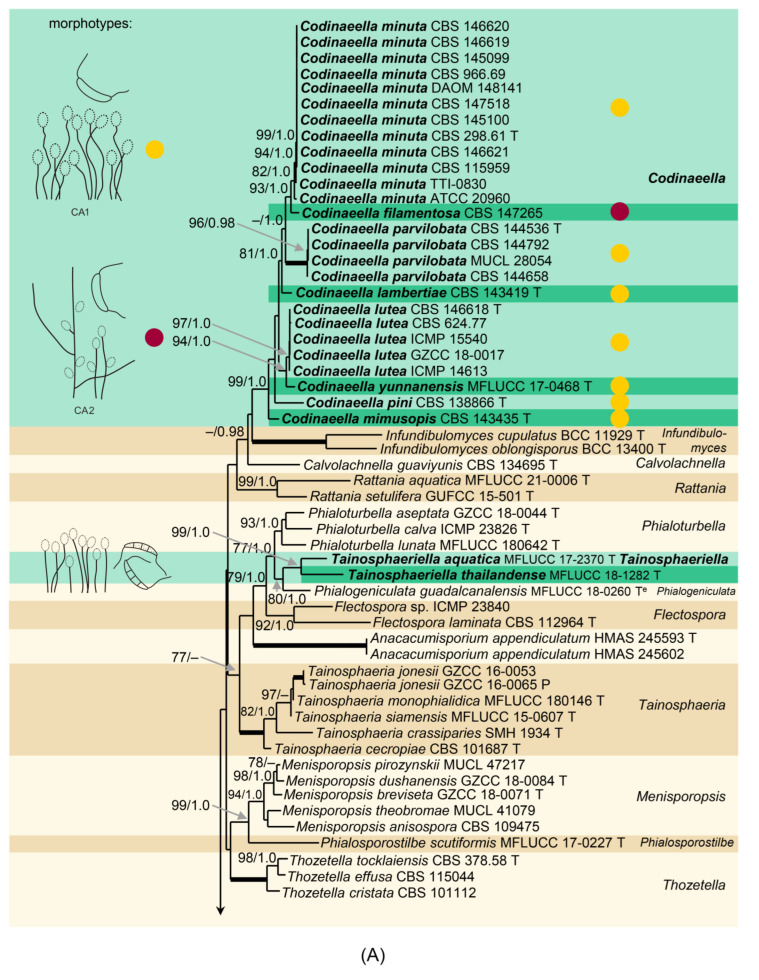

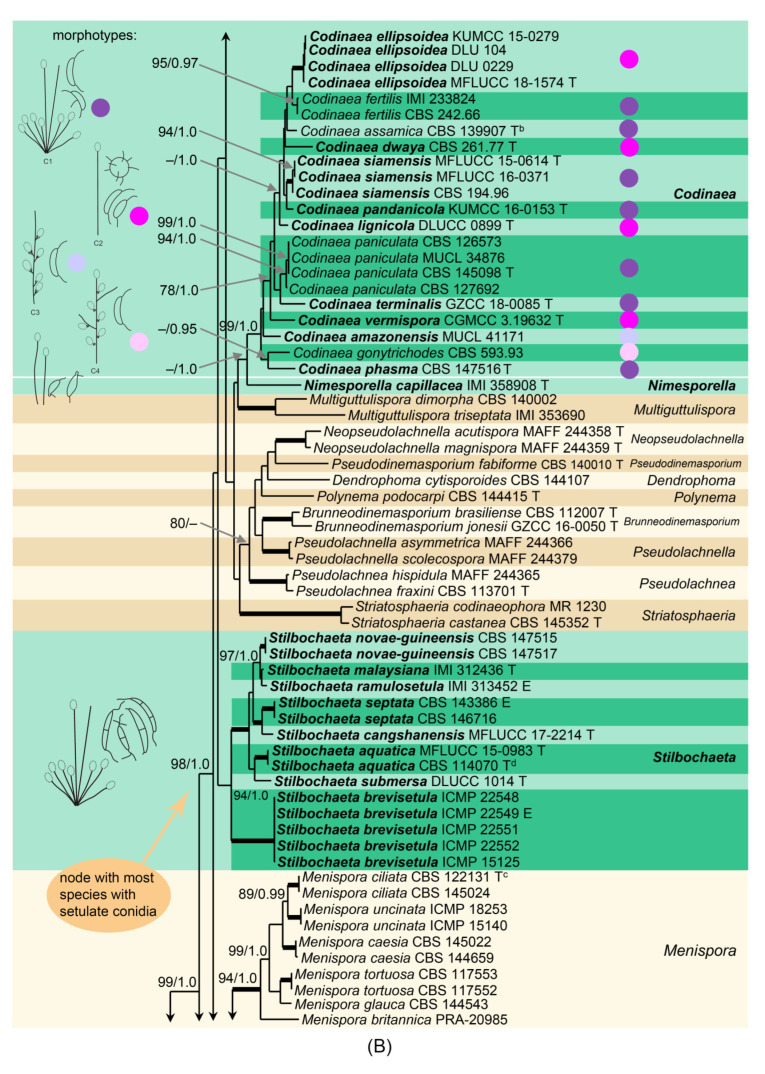

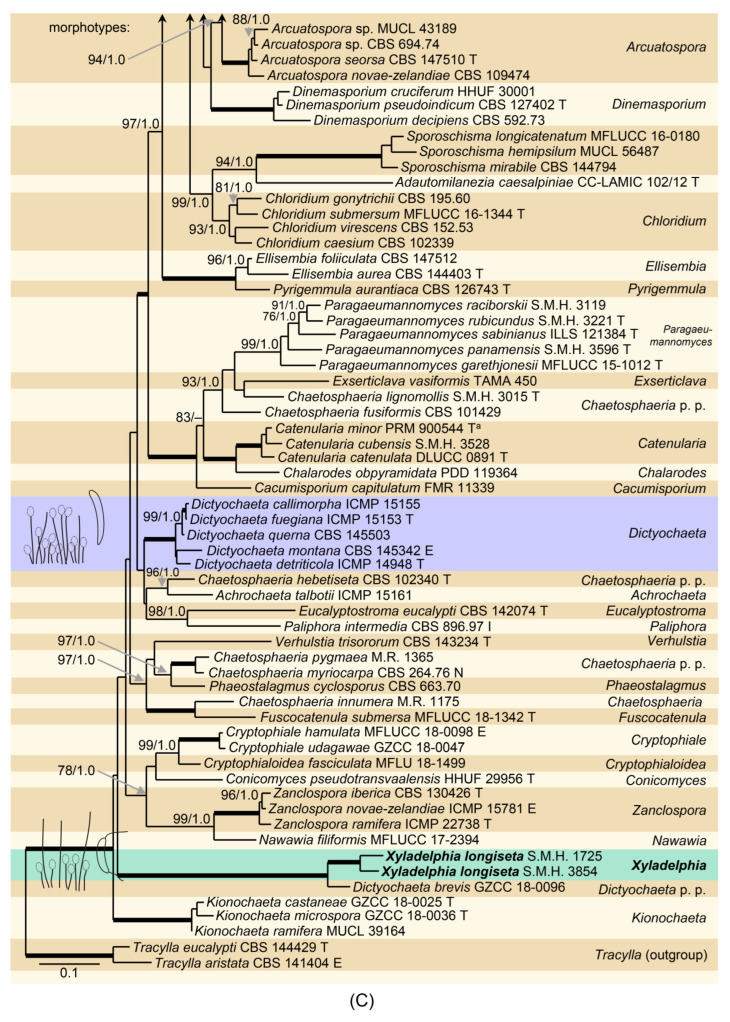
(**A**) Phylogenetic tree based on the combined ITS-28S-*tef1-α* rDNA sequences constructed by maximum likelihood (RAxML) of members of the Chaetosphaeriaceae. Species names given in bold and placed in green boxes are taxonomic novelties. Each novelty is accompanied by a respective morphotype(s); their distribution within the genus is indicated by circles of different colors. *Codinaea*: morphotypes C1 (dark violet), C2 (fuchsia), C3 (lila), C4 (pink); *Codinaeella*: morphotypes CA1 (yellow), CA2 (burgundy). The clade containing *Dictyochaeta* s. str. is placed in a violet box. Abbreviations T, E, I, N, and P indicate ex-type, ex-epitype, ex-isotype, ex-neotype and ex-paratype strains; letters in the upper case after a species name indicate: (a) holotype of *Chaetosphaeria trianguloconidia*, (b) ex-type strain of *Codinaea acaciae*, (c) ex-type strain of *Chaetosphaeria ciliata*, (d) ex-type strain of *Dictyochaeta curvispora* (contamination), (e) ex-type strain of *Tainosphaeria obclavata*. Thickened branches indicate branch support with ML BS = 100% and PP values = 1.0. Branch support of nodes ≥ 75% ML and ≥0.95 PP is indicated above or below branches. (**B**,**C**) Phylogenetic trees based on the combined ITS-28S-*tef1-α* rDNA sequences of the Chaetosphaeriaceae (continued). For legend refer to (**A**). Abbreviation: p.p. after a genus name (*pro parte*).

Phylogenetic relationships were resolved based on the analysis of ITS, 28S, and *tef1-α* sequences of 179 strains of members of the Chaetosphaeriaceae. The phylogenetic trees generated by BI and ML analyses were largely congruent; the nodes with support values of ≥75% ML BS and ≥0.95 BI PP were considered well-supported. The ML tree is shown in Figure 2. The family Chaetosphaeriaceae included 57 well-supported lineages that represent genera or natural groups of species. Analyzed species of *Codinaea*, *Codinaea*-like, and *Dictyochaeta*-like fungi were resolved in eleven major groups. *Codinaea* is shown as a strongly supported clade (99 ML BS/1.0 BI PP); it contains 22 strains representing 13 species including the ex-type strains of *Bahusutrabeeja dwaya* CBS 261.77 and *Phialolunulospora vermispora* CGMCC 3.19632 and a non-type strain of *Codinaeopsis gonytrichodes* CBS 593.93; all three species are generic types. Several other species with setulate conidia formed two well-supported undescribed lineages introduced below as the new genera *Codinaeella* (99/1.0) including 26 strains of eight species and *Stilbochaeta* (94/1.0) with 15 strains representing eight species. A *Codinaea*-like species formed a separate lineage basal to *Codinaea*, although statistically unsupported in the ML analysis, and is proposed as a new species in the new genus *Nimesporella*. In addition, several other species with *Codinaea*-like and *Dictyochaeta*-like morphotypes clustered as distantly related lineages. The genera *Dictyochaeta* (100/1.0) and also *Multiguttulispora* (100/1.0), *Striatosphaeria* (100/1.0), and *Tainosphaeria* (100/1.0) containing former *Codinaea* and *Dictyochaeta* species were resolved as strongly supported clades. The ex-type strains of two *Tainosphaeria* species, *T. aquatica* MFLUCC 17-2370 and *T. thailandensis* MFLUCC 18-1282, formed a strongly supported lineage (99/1.0). Their relationship with the type species of *Tainosphaeria*, *T. crassiparies*, was not confirmed. Specifically, they nested as a sister to *Phialogeniculata guadalcanalensis* in a statistically unsupported clade containing several other genera, such as *Anacacumisporium* (100/1.0), *Flectospora* (92/1.0), and *Phialoturbella* (93/1.0). Therefore, a new genus *Tainosphaeriella* is proposed for the lineage of the two former *Tainosphaeria* species. Two non-type strains of *Chaetosphaeria longiseta* S.M.H. 1725 and S.M.H. 3854 and the ex-type strain of *Dictyochaeta brevis* GZCC 18-0096 grouped as sister clades in a well-supported lineage (100/1.0) near the base of the tree. Their taxonomic treatment is proposed below.

**Figure 3:**
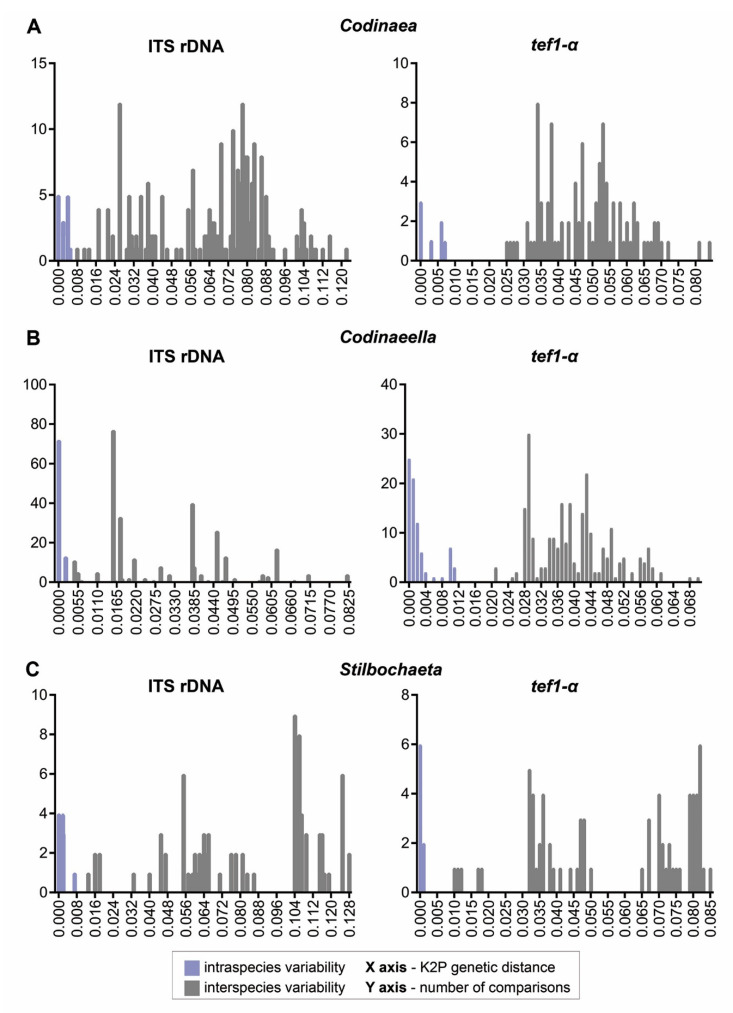
The frequency distribution graphs of the Kimura 2-parameter distances of ITS and *tef1-α* DNA sequences (barcoding gaps). (**A**) *Codinaea*; (**B**) *Codinaeella*; (**C**) *Stilbochaeta*. The intraspecific distances are shown as blue bars and interspecific distances as grey bars. The bin size of 0.001 and 0.0005 (only ITS, *Codinaeella*) were used.

For two markers tested (ITS and *tef1-α*), the barcoding gap separated intraspecific from interspecific variation in all species (Figure 3). However, the length of the barcoding gap ranged between genera and markers. For the ITS, the barcoding gap was rather narrow, i.e., *Codinaea* 0.4–0.8%, *Codinaeella* 0.2–0.4%, and *Stilbochaeta* 0.6–1.3%. On the other hand, the *tef1-α* marker showed a large gap between intraspecific and interspecific variability, i.e., *Codinaea*: 0.7–2.4%, *Codinaeella:* 1.5–2.1%, and *Stilbochaeta:* 0.1–1.0%. The lowest interspecific divergence for ITS ranged from 0.4% (*Ca. filamentosa* vs. *Ca*. *minuta*) and 0.8% (*C. pandanicola* vs. *C. siamensis*) to 1.3% (*S. malaysiana* vs. *S. ramulosetula*), while the highest ranged from 8.2% (*Ca. parvilobata* vs. *Ca. pini*) and 12% (*C. ellipsoidea* vs. *C. phasma*) to 13% (*S. aquatica* vs. *S. brevisetula*). For the *tef1-α* gene, the lowest interspecific divergence ranged from 1.0% (*S. malaysiana* vs. *S. ramulosetula*) and 2.1% (*Ca. lutea* vs. *Ca. yunnanensis*) to 2.4% (*C. assamica* vs. *C. siamensis*) and the highest from 7% (*Ca. mimusopis* vs. *Ca*. *pini*) to 8.1% (*C. amazonensis* vs. *C. dwaya*) and 8.5% (*S. brevisetula* vs. *S. septata*). Based on these results, the markers ITS and *tef1-α* are recommended as barcodes for *Codinaea*, *Codinaeella,* and *Stilbochaeta*.

### 3.2. Consensus 2D Structure of ITS2

**Figure 4:**
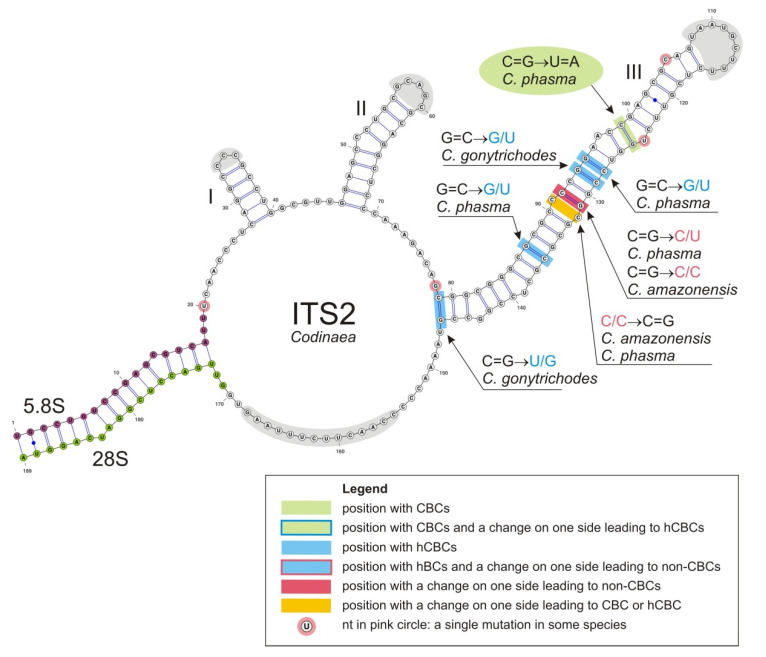
ITS2 secondary structure of *Codinaea assamica* CBS 139907 and 5.8S-28S rRNA gene hybridization (proximal stem region) (GenBank accessions ITS: OL654077, 28S: OL654134). ITS2 helices are numbered I–III. All substitutions recorded among members of *Codinaea* are mapped on the 2D model. Identified substitutions are color-coded: CBC (green), hCBC (blue), and non-CBC (red). Parts of the text in green frames refer to CBCs, parts of the text in blue and red refer to hCBCs and non-CBCs, respectively. Hairpin loops and other parts of the ITS2 molecule highlighted with grey color represent regions with a variable number of nucleotides or sequence variation; positions highlighted with a pink color represent single mutations.

**Figure 5:**
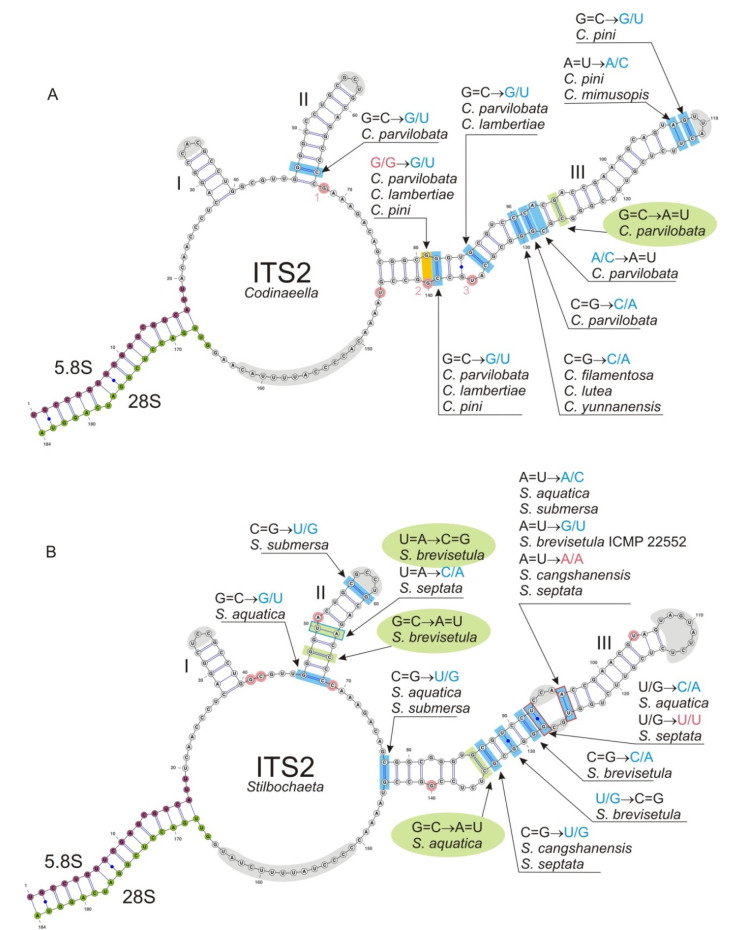
ITS2 secondary structure of *Codinaeella* and *Stilbochaeta* and 5.8S-28S rRNA gene hybridization (proximal stem region). (**A**) *Codinaeella minuta* CBS 146620 (GenBank accessions ITS: OL654095, 28S: OL654152) (**B**) *Stilbochaeta malaysiana* IMI 312436 (GenBank accessions ITS: OL654121, 28S: OL654178). Details as in Figure 4.

The CBC species concept is based on the co-evolution of nucleotides involved in CBCs and hCBCs in the two most conserved helices H2 and H3 [103] and also the H1 helix [108] of the ITS2 molecule. The 2D consensus structure models for *Codinaea* (mapped onto *C. assamica*, Figure 4), *Codinaeella* (mapped onto *Ca. minuta*, Figure 5A), and *Stilbochaeta* (mapped onto *S. malaysiana*, Figure 5B) were created. Once all existing substitutions were identified, they were mapped onto the predicted 2D structure models of ITS2 of the three genera. In particular, we focused on CBC substitutions in the ITS2 molecule, which are significant for detecting species that are sexually incompatible. The ITS2 has a common core structure in all three genera; the secondary structure folds into a ring structure with three main helices (H1–H3), while helix H4 is missing due to large variability. Base changes were detected only in H2 (except for *Codinaea*) and H3. The H1 is a short, conserved helix consisting of five base pairs (in all three structures) with a variable 3 nt hairpin loop. The H2 is a nine base pairs long helix with one bulge in the middle of the duplex and a variable 3–5 nt hairpin loop. In *Stilbochaeta*, we detected five base changes, three of them were different hCBCs and two were CBCs, both between *S. brevisetula* and other species of the genus. Only one hCBC change was observed in *Codinaeella*. The H3 is the largest duplex (length 66–68 nt) with a number of base pair changes. All three models consistently contained one CBC substitution but various numbers of hCBC and non-CBC changes. Specifically, we observed one CBC in *C. phasma* (against other species of the genus), and four hCBCs and two non-CBC changes in *Codinaea*. More events were detected in *Codinaeella*, in particular one CBC between *Ca. parvilobata* and other species, 15 different hCBCs, and three non-CBC changes. Substitutions identified are *Stilbochaeta* included one CBC in *S. aquatica* and two in *S. brevisetula* against other species of the genus, but also 11 different hCBCs among species and two combined base changes (non-CBS and hCBC) in *S. cangshanensis* and *S. septata* on both sides of the asymmetric loop of the duplex. Primary sequence variations among other species of *Codinaea*, *Codinaeella,* and *Stilbochaeta* were low and included single mutations in hairpin loops of helices H1, H2, and H3, in the junction areas between helices and also in the helices.

### 3.3. Ancestral State Reconstruction

**Figure 6:**
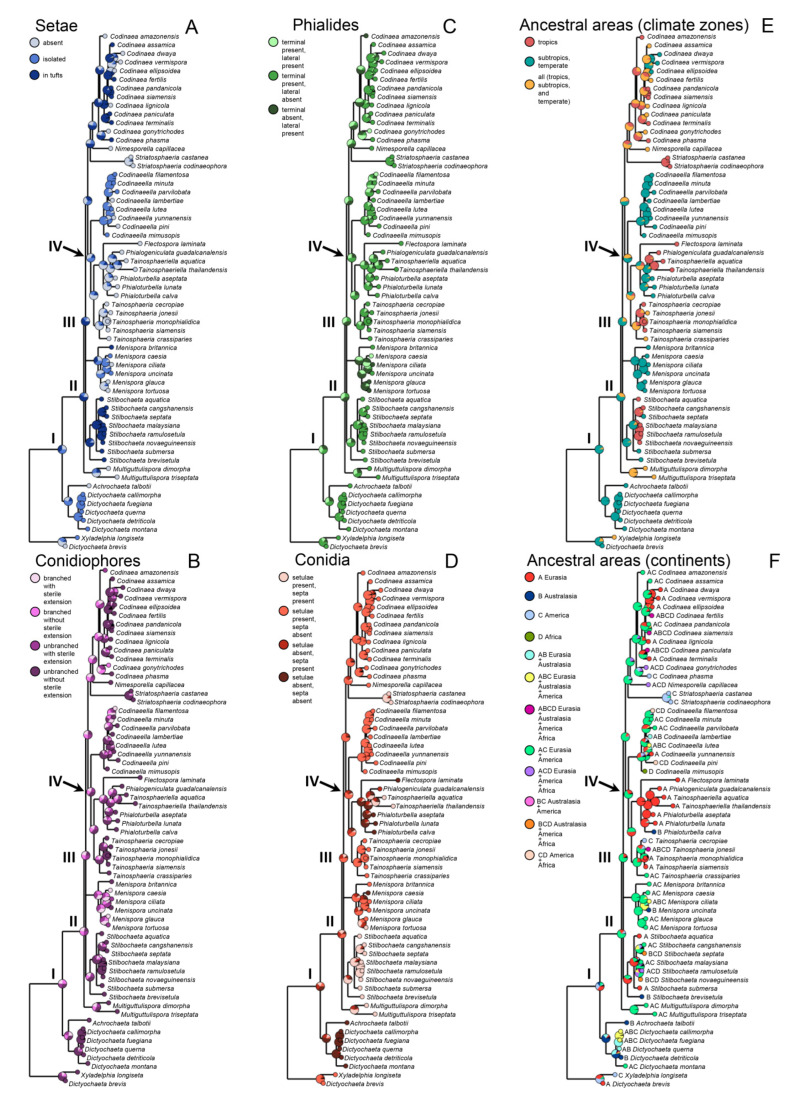
Reconstruction of ancestral states and areas in Rasp v.4.2 using phylogenetic trees calculated in MrBayes v.3.2.7. (**A**–**D**) Reconstruction of morphological characters using Multistate function in Bayestraits (**E**,**F**) ancestral areas reconstruction of species distribution using Bayesian Binary MCMC (BBM) method.

Our analysis showed that the ancestral state of setae type (Figure 6A, node I) was the presence of all states (setae absent, present, tufts) and only the genus *Menispora* still retained this ancestral polymorphism. In other lineages, we can see the evolution to the particular morphological type, where setae occurred solitarily (*Codinaeella* and *Dictyochaeta*), in tufts (*Codinaea, Stilbochaeta*), or were lost (genera evolving from the node IV and *Multiguttulispora*). In the conidiophore type evolution, we can see the trend from the simple types, such as unbranched conidiophore without (Figure 6B, node I) and with sterile extension (node II) toward the more complex conidiophore types occurring rarely in *Codinaea* and *Codinaeella*. Again, *Menispora* showed the diversity of all conidiophore types. Similar to setae evolution, the analysis showed that the evolution of the phialide position started with all states (Figure 6C, node I). During the evolution, the presence of the terminal phialide became the probable ancestral state (node II) and this character occurs in most of the lineages. Other types occur randomly and rarely in *Codinaea*, *Codinaeella*, and *Menispora*. During conidia evolution, the simplest type (no setulae, no septa) was ancestral (Figure 6D, node I) but more complex conidia types (with setulae and septa) subsequently evolved in several lineages (*Stilbochaeta*, *Multiguttulispora, Tainosphaeriella*). Concerning conidia type evolution, the most common type was conidia with setulae but without septa. *Menispora* showed the diversity of all conidia types (Figure 6D). The evolution of the conidia shape started from elongated types (falcate, lunate, narrowly fusiform, curved) to broader conidial shapes (ellipsoidal, ellipsoidal-fusiform, oblong, reniform, globose, pyriform, papillate, obovoid, ellipsoidal-clavate, cylindrical-clavate) (Supplementary File: Figure S1).

Reconstruction of the ancestral climatic zone revealed the temperate zone as the ancestral area (Figure 6E, nodes I and II) with the subsequent transition to the tropical zones. This transition occurred independently in five lineages and seems to be irreversible. Most lineages showed a clear preference to the particular climatic zone. The reconstruction of the ancestral geographical distribution showed Eurasia (Figure 6F, node I) or Eurasia and Americas (nodes II and IV) as ancestral and the presence in African and Australasian areas as derived. Most of the clades showed broader geographical distribution and only a few lineages are more or less restricted to other areas such as Eurasia (lineages of *Flectospora*, *Phialoturbella*, *Phialogeniculata*, and *Tainosphaeriella*) or Americas (*Striatosphaeria*).

### 3.4. Diversity Analysis of Codinaea and Related Fungi in Environmental Samples, Biogeography, and Ecology

**Figure 7:**
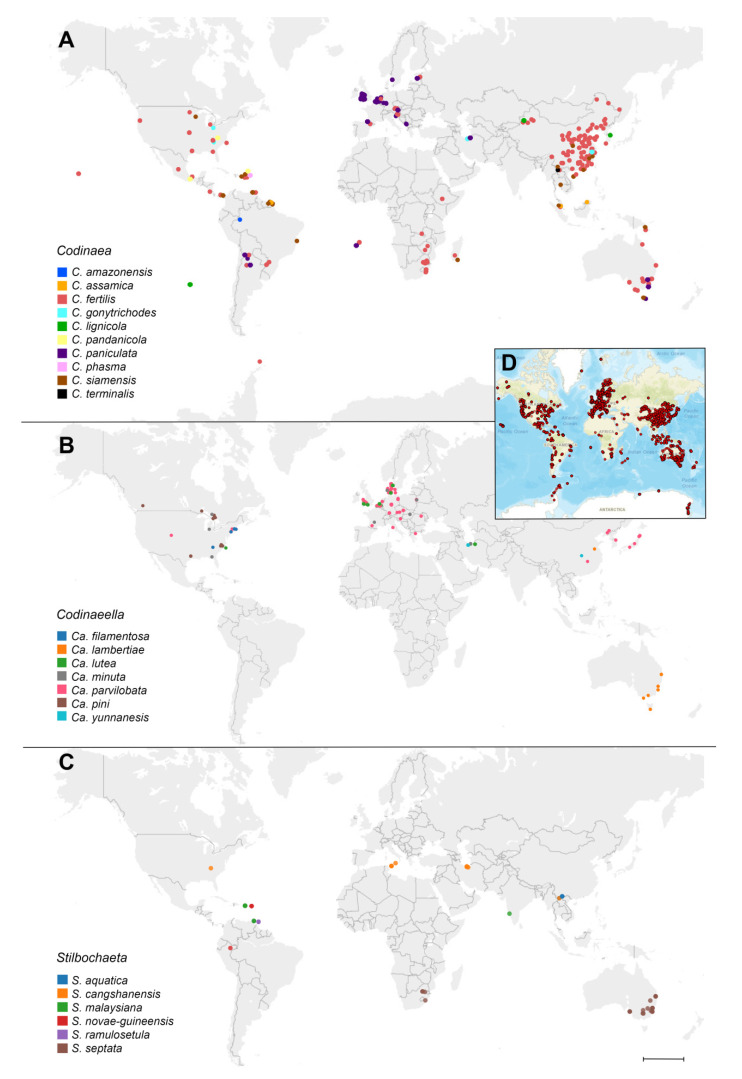
Geographical distribution of (**A**) *Codinaea*, (**B**) *Codinaeella,* and (**C**) *Stilbochaeta* species based on the GlobalFungi database. See Table S2 for primary data. Species are differentiated by colors. (**D**) The circles represent smaller geographical areas with one or more observations of the particular species. Some of the smaller areas contained observations of multiple species (e.g., Caspian coast, Puerto Rico); circles were manually dispersed over the actual collection site. Scale bar: 2000 km.

The datamining resulted in 185,176 reads that can be assigned to excerpted species of *Codinaea*, *Codinaeella,* and *Stilbochaeta*. Several species were not found in GlobalFungi database, such as *C. dwaya*, *C. ellipsoidea, C. vermispora, Ca. mimusopis*, *S. brevisetula,* and *S. submersa*, and can be considered rare or highly geographically restricted. In average, each species was found in 57 samples (min. one sample, max. 655). Most of the samples originate from bulk soil (65% of all samples), followed by roots (16%), rhizosphere soil (11%), shoots (3.5%), litter (2.4%), and other substrates. Majority of samples were collected in forests (55%), croplands (19%), and grasslands (17%) followed by other biomes. If we consider the density of sampling across various world regions, we can draw several well supported conclusions (Figure 7, Supplementary Files: Figure S2, Table S2).

Basically, the species distribution followed mainly the climatic zones and can be classified into the three distribution types: (1) tropical climate, i.e., *C. amazonensis*, *C. pandanicola, C. phasma*, *C. siamensis*, *C. terminalis, S. novae-guineensis, S. malaysiana,* and *S. ramulosetula*; (2) temperate, and/or in cold humid or continental humid climates, i.e., the whole genus *Codinaeella*, also *C. lignicola*, *C. paniculata*, *S. aquatica*, and *S*. *cangshanensis*; (3) usually distributed across temperate, tropical, or colder and drier climates, i.e., *C. assamica* and *C. fertilis*. With a few exceptions (*Ca. lutea*, *Ca. pini*), these taxa are almost absent in the well sampled areas of western part of North America. The distribution of members of *Codinaea, Codinaeella,* and *Stilbochaeta* never reaches boreal climatic zone and the northern limit of their distribution is southern Sweden, Wales (UK), and US/Canada border. Ninety-six per cent of the samples were collected from sites with MAT bellow 6 °C and MAP lower than 500 mm, and there is also a marked intolerance to colder and drier climates. Species of Holarctic distribution have been found in Nearctic (*C. gonytrichodes*, *Ca. filamentosa*, *Ca. pini*), Palearctic (*C. lignicola*, *Ca. yunnanensis*, *S. aquatica*) or in both regions (*Ca. lutea*, *Ca. minuta*, *Ca. parvilobata*, *S. cangshanensis*). Species with tropical distribution were found in Neotropics (*C. amazonensis*, *C. phasma*, *S. novae-guineensis*, *S. ramulosetula*), Paleotropics (*C. terminalis*) or in both tropical realms (*C. assamica*, *C. pandanicola*, *S. malaysiana*). Some species were present in Australasia and Southeast Asia (*C. lambertiae*) or South Africa (*S. septata*). The global distribution was found in *C. paniculata* (mostly temperate region), *C. siamensis* (tropical or very humid regions) and *C. fertilis* (all climatic zones). The most abundant species were *C. fertilis* (655 samples), *Ca. parvilobata* (227 samples), and *C. paniculata* (111 samples).

Using the GlobalFungi database, we identified diversity hotspots in Caspian Hyrcanian mixed forests, where we found five species (*C. gonytrichodes*, *Ca. lutea*, *Ca. minuta*, *Ca. yunnanensis*, *S. cangshanensis*) in samples from the study of Bayranvand et al. [115]. The centre of diversity of tropical species was found in Central America in Puerto Rico (*C. siamensis*, *C. phasma*, *C. fertilis*, *C. pandanicola*, *S. novae-guineensis S. malaysiana*) in samples studied by Peay et al. [116], Tedersoo et al. [117], Urbina et al. [118], and Zhou et al. [119].

### 3.5. Taxonomy

Based on the results of multilocus phylogenetic analysis, ancestral inference, barcode gap, morphological data, and assessment of biogeography, the new taxonomic treatment of *Codinaea* and similar fungi is presented. The current circumscription of *Codinaea* is revised and several new genera, species, and combinations are proposed below. A key to *Codinaea*, its segregates, and similar fungi is provided (Table 2).

#### 3.5.1. The Genus Codinaea

***Codinaea*** Maire, Publ. Inst. Bot. Barcelona 3: 15. 1937.

= *Menisporella* Agnihothr., Proc. Natl. Acad. Sci. India B 56: 98. 1962.

= *Codinaeopsis* Morgan-Jones, Mycotaxon 4: 166. 1976.

= *Bahusutrabeeja* Subram. & Bhat, Can. J. Bot. 55: 204. 1977.

= *Phialolunulospora* Z.F. Yu & R.F. Castañeda, MycoKeys 76: 23. 2020.

Type species. *Codinaea aristata* Maire, Publ. Inst. Bot. Barcelona 3: 15. 1937.

Emended description: Colonies on natural substrate effuse, hairy, brown to black, composed of setae and conidiophores, mycelium semi-immersed or immersed. **Anamorph.** Setae present or absent, if present single or arise in groups from stromatic cells or knots of hyphal cells, erect, straight or flexuous, septate, pigmented, thick-walled, paler and thinner-walled toward the apex, unbranched, apex sterile, tapering or modified into a phialide with a terminal or several lateral openings. Conidiophores macronematous, mononematous, single or arise in fascicles from stromatic cells or knots of hyphal cells around the base of the setae, erect, straight or flexuous to slightly geniculate or undulate, unbranched or branched, occasionally with nodose or collar-like hyphae formed just below the septa, septate, smooth, pigmented, paler toward the apex, terminating into a phialide or with a sterile setiform extension. Conidiogenous cells integrated, terminal or discrete, lateral, mono- or polyphialidic, extending percurrently and sympodially, paler than the conidiophore; collarettes funnel-shaped. Conidia of two morphologically distinct types; macroconidia predominantly falcate to lunate, sometimes oblong-falcate, ellipsoid to ellipsoid-fusiform or broadly oblong, occasionally vermiform, slightly truncate at the base, with an inconspicuous basal scar, with a straight or gently curved setula at each end, sometimes setulae also inserted ventrally and dorsally, or also globose to pyriform with setulae distributed over the surface of the conidium, aseptate, hyaline, conidia accumulate in slimy fascicles; microconidia (formed occasionally and only in vitro) ellipsoidal to oblong, aseptate, hyaline, with a miniature setula. **Teleomorph.** Unknown.

Habitat and geographical distribution: *Codinaea* includes mainly saprobes that have been recorded from soil, decaying fruits, leaves, petioles, palm fronds, bark, wood, and roots, as well as leaf spots of a wide range of plant hosts. Some species have also been isolated as endophytes. In addition, *C. fertilis* has been recorded as a plant pathogen. The genus *Codinaea* has a wide geographic distribution with occurrence in the tropical and temperate climatic zones supported by literature, field records, and environmental sequence data from the GlobalFungi database.

Note: In the three-gene phylogeny (Figure 2), *Codinaea* is shown as a strongly supported monophyletic clade. Based on the results of the phylogenetic analysis, the generic concept of *Codinaea* was emended and 14 species were accepted in the genus; the inclusion of 13 species was verified with DNA data. The teleomorph of *Codinaea* is unknown. Although we were not successful in recollecting *C. aristata*, we analyzed seven other species that match the *Codinaea* s. str. Morphotype in all details, i.e., dark brown, thick-walled central seta or setae arranged in a fascicle with shorter, paler, and thinner-walled conidiophores bearing terminal phialides and falcate, aseptate, hyaline conidia with setulae at each end. All seven species of the *C. aristata* morphotype clustered in the *Codinaea* clade, namely *C. assamica, C. fertilis*, *C. pandanicola*, *C. paniculata*, *C. phasma*, *C. siamensis,* and *C. terminalis*.

**Figure 8:**
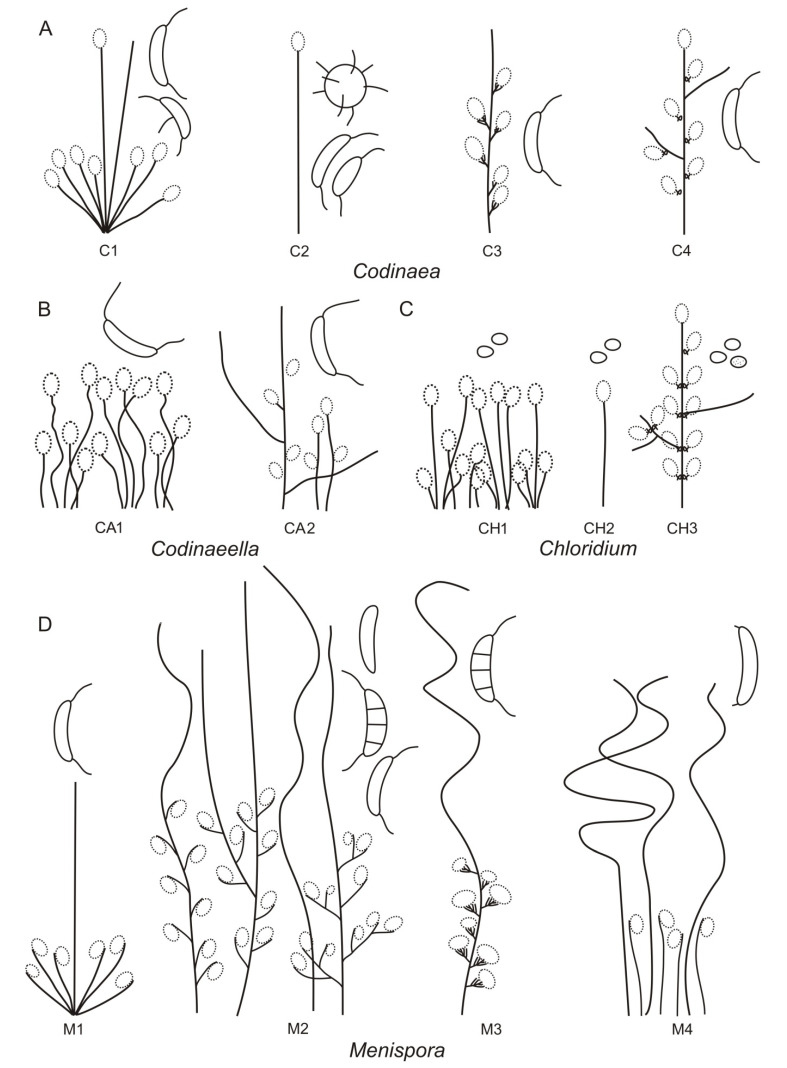
Illustrations of morphotypes based on the arrangement of setae, conidiophores and conidiogenous cells. (**A**) *Codinaea* morphotypes C1–C4 (**B**) *Codinaeella* morphotypes CA1–CA2 (**C**) *Chloridium* morphotypes CH1–CH3 (**D**) *Menispora* morphotypes M1–M4.

**Figure 9:**
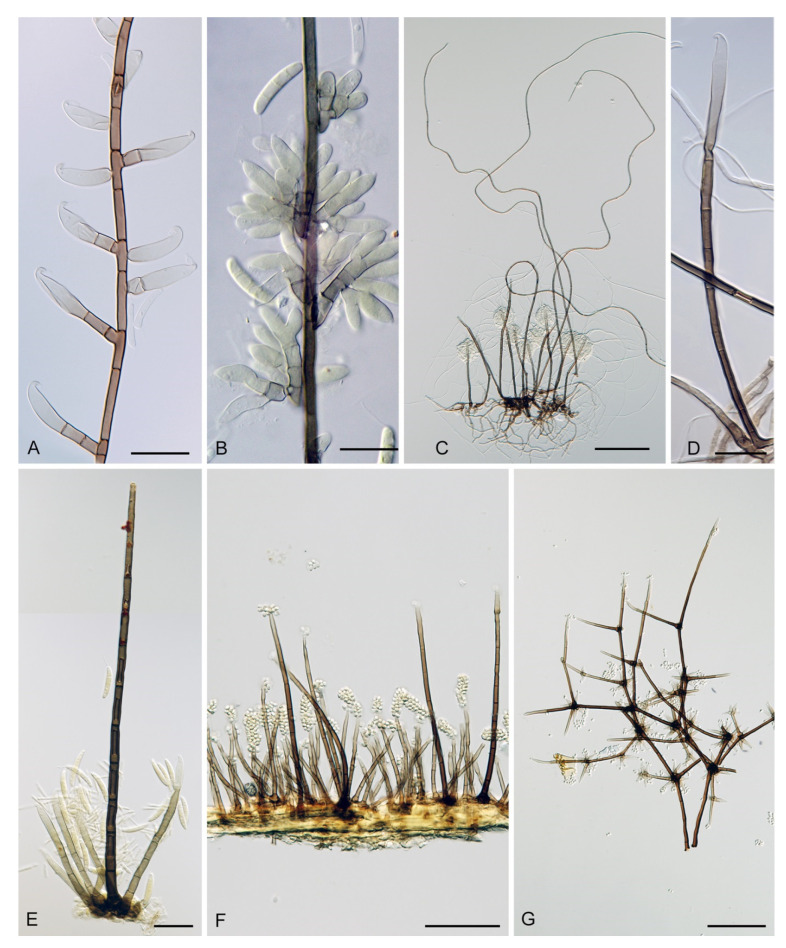
Selected morphotypes of *Menispora* and *Chloridium*. (**A**) *M. ciliata* morphotype M2 (CBS 122131); (**B**) *M. tortuosa* morphotype M3 (CBS 117552) (**C**,**D**) *M. uncinata* morphotype M4 (ICMP 18253) (**E**) *M. britannica* morphotype M1 (PRA-20985) (**F**) *Chl. virescens* morphotype CH1 (M.R. 3886) (**G**) *Chl. caesium* morphotype CH3 (M.R. 1883). Scale bars: (**A**,**B**,**D**,**E**) 20 μm; (**C**) = 100 μm; (**F**,**G**) 50 μm.

Based on the arrangement of setae and conidiophores and their branching pattern, we distinguish four morphotypes C1–C4 (Figure 8) in *Codinaea*. In addition to species with the *Codinaea* s. str. morphotype (C1), other lineages of species characterized by three other morphotypes were nested among them. Four species with unbranched, dark brown, thick-walled conidiophores that closely resemble the setae and terminate into a monophialide (C2: e.g., *C. ellipsoidea* and *C. lignicola*); one species with conidiophores with a sterile setiform extension and integrated, terminal phialides borne in groups on short unilateral, branched stalks (C3: *C. amazonensis*), and one species with branched conidiophores and discrete, lateral phialides borne on nodose hyphae or directly on conidiophores (C4: *C. gonytrichodes*). The conidiophore variability found in *Codinaea* compares well with that of *Chloridium* (*Chl*.) and *Menispora* (Figure 8 and Figure 9) (see Discussion, Section 4.1).

Conidia also show some variability. Although they are always aseptate and hyaline, they vary in shape. They are mostly falcate to lunate, curved, occasionally vermiform but several species have also ellipsoidal-fusiform to more or less ellipsoidal conidia with setulae inserted at the apical and basal ends. The globose to pyriform conidia of *C. dwaya* with numerous setulae irregularly distributed over the surface represent a character that is unusual in *Codinaea*. The falcate conidia of *C. phasma* form a simple setula at each end under natural conditions, however, in culture, they become irregularly ellipsoidal and have 3(–5) setulae inserted also on the ventral and dorsal sides.

Interestingly, the ex-type strains of *Bahusutrabeeja dwaya* CBS 261.77 [120] and *Phialolunulospora vermispora* CGMCC 3.19632 [50], and a non-type strain of *Codinaeopsis gonytrichodes* CBS 593.93 [42] clustered in this clade. The two former species form simple conidiophores terminating into a monophialide and correspond to the C2 morphotype, while *C. gonytrichodes* is the only representative of the C4 morphotype. Based on the phylogenetic evidence, *Bahusutrabeeja*, *Codinaeopsis,* and *Phialolunulospora* are proposed as generic synonyms of *Codinaea.* Although *Codinaeopsis* and *Phialolunulospora* are monotypic genera, *Bahusutrabeeja* accommodates five other species, but only two of them resemble *C. dwaya*.

Several other morphologically similar species, whose living cultures or DNA sequence data are not available for study, are discussed below. These taxa should be considered candidates for inclusion in *Codinaea*, however, such relationships need to be supported by molecular data. Species such as *Dictyochaeta gyrosetula* [24], *D. intermedia* [121], *D. plovercovensis* [122], *D. tilikfrei* [123], and *D. vittata* [24] correspond to the *Codinaea* s. str. morphotype (C1). The C2 morphotype also occurs in *C. aquatica* [124], *C. leomaiae* [125], *C. tropicalis* [126], *D. fimbriaspora* [44], *D. multifimbriata* [34], *D. multisetula,* and *D. renispora* [44], which often have ellipsoidal to reniform or drop-shaped conidia with two or more setulae. Morphotype C4 with discrete phialides borne on nodose hyphae was also recorded in *D. pahangensis* [21] and *Dictyochaetopsis polysetosa* [127]. A synopsis table with diagnostic features of accepted species of *Codinaea* is provided in the Supplementary File: Table S4.

**Figure 10:**
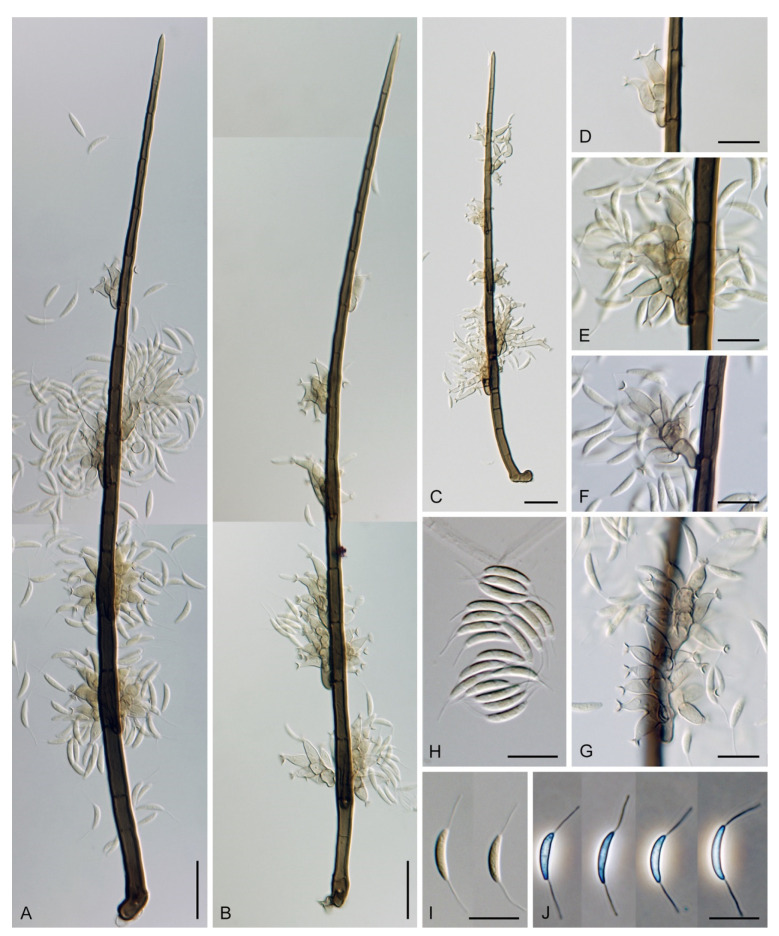
*Codinaea amazonensis* (MUCL 41171). (**A**–**C**) Conidiophores (**D**–**G**) conidiogenous cells (**H**–**J**) conidia. Images: (**A**–**J**) on MLA after 4 weeks. Scale bars: (**A**–**C**) 20 μm; (**D**–**J**) 10 μm.

**Figure 11:**
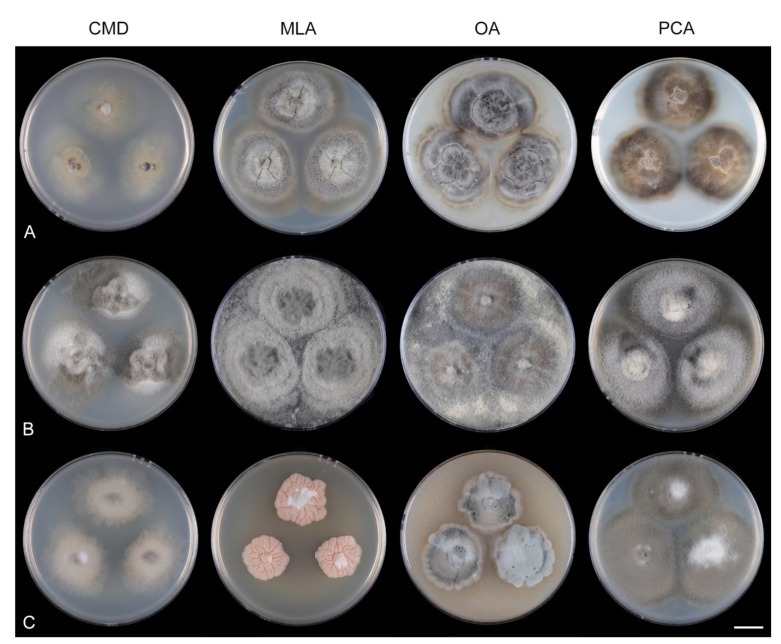
Colony morphology of *Codinaea* spp. after 4 weeks. (**A**) *C. amazonensis* MUCL 41171 (**B**) *C. gonytrichodes* CBS 593.93 (**C**) *C. phasma* CBS 147516 ex-type. Scale bar: (**A**–**C**) 1 cm.

***Codinaea amazonensis*** (Matsushima) Réblová & Hern.-Restr., **comb. nov.** MycoBank MB 842191 (Figure 10 and Figure 11).

Basionym. *Menispora amazonensis* Matsush., Matsush. Mycol. Mem. 7: 57. 1993.

Culture characteristics: On CMD: colonies 33–35 mm diam, circular, flat, margin entire, cobwebby, beige-brown, beige toward the margin, reverse isabelline. On MLA: colonies 65–67 mm diam, circular, raised, margin entire, velvety-lanose, floccose, furrowed, zonate, smoke grey centrally, dark grey toward the margin, white-grey at the margin, with a grey-brown outer zone of submerged growth, reverse dark grey-brown. On OA: colonies 57–58 mm diam after 28 d, circular, raised, margin entire, velvety to cobwebby, mucoid at the margin, furrowed, zonate, with an intermediate zone of sparse growth, beige-brown, olivaceous brown at the margin, reverse dark brown. On PCA: colonies 58–60 mm diam, circular, slightly raised, flat at the margin, margin entire to curled, cobwebby, floccose locally, mucoid toward the periphery, beige-brown, dark brown with an orange tinge at the margin, reverse dark brown. Sporulation was sparse on CMD, OA, and PCA, moderate on MLA.

Colonies on MLA effuse, hairy, mycelium composed of branched, septate, hyaline to subhyaline hyphae 1.5–2 μm diam. **Anamorph.** Setae absent. Conidiophores 260–320 μm long, 4.5–6.5 μm wide near the base, macronematous, single, erect, septate, smooth, dark brown, slightly paler toward the apex, with a sterile setiform extension, apex acute, pale brown, conidiophores with unilateral phialide-bearing stalks, which arise just below the septa, 19–36 μm long including the phialides, 3.5–5 μm wide at the base, stalks are curved upwards, composed of a cylindrical basal cell and several compact, densely aggregated cells bearing 1–15 phialides, basal cell is medium to dark brown, other cells are pale brown to subhyaline. Conidiogenous cells 8.5–13.5 × 3–4.5(–5) μm tapering to 1–2 μm below the collarette, discrete, lateral, arise on stalks or on conidiophores, sometimes stalked or sessile phialides arise on vegetative mycelium, mostly monophialidic, occasionally with 1–2 lateral openings, extending percurrently and sympodially, lageniform, pale brown, paler than the conidiophore; collarettes 2.5–4 μm wide, funnel-shaped, subhyaline. Conidia 9–11.5 × 1.5–2(–2.5) μm (mean ± SD = 9.9 ± 0.7 × 2.0 ± 0.2 μm), falcate, tapering toward both ends, slightly truncate at the base with an inconspicuous scar, aseptate, hyaline, with straight or gently curved setula at each end 5.5–9 μm long, inserted terminally at the apex, subterminally at the base, conidia accumulate in pale ochre fascicles. **Teleomorph.** Unknown.

Specimen examined: BRAZIL, Mata Avenca-Santa Rita, on rotten leaf, 10 September 1997, J. Guarro (culture MUCL 41171).

Habitat and geographical distribution: According to literature, it is a rare species isolated from soil and decaying leaves of *Quercus dentata* and other unidentified hosts in South America and Asia: Brazil and Japan [5,128,129]. Czeczuga and Orłowska [130] reported this species (as *Menispora amazonensis*) in the water from melting ice collected from branches of coniferous trees in Poland. However, given the similarity of *C. amazonensis* to *Menispora tortuosa* [131,132,133], which is common in the temperate zone of Europe and North America, and the known distribution of *C. amazonensis* in tropical and subtropical areas, it is possible that the fungus from melting snow may have been misidentified.

According to GlobalFungi, the identical sequences were found in one soil sample from the forest in South America (Amazonian river basin, Brazil) [117], which is in agreement with the original data and where the type was collected [128]. The location has a tropical climate (MAT avg. 25 °C, MAP avg. 2803 mm).

Notes: *Codinaea amazonensis* was originally described from a decaying leaf in the Amazon river basin in Peru [128]. However, no type or authentic material is available. Our strain MUCL 41171, originally published under the name *Codinaea gonytrichodes* [129], matches the protologue and original illustration of *C. amazonensis* in all details.

This species differs from other *Codinaea* species in the branching pattern of the conidiophores, which have a sterile extension. In addition, short stalks arise unilaterally almost the entire length of the conidiophores; they consist of several compact cells that bear a group of phialides. *Codinaea amazonensis* superficially resembles *C. gonytrichodes*, which forms collar-like and nodose hyphae at the conidiophore septa, from which arise discrete phialides and occasionally branches and setae. Interestingly, the fungus reported as *C. gonytrichodes* from Japan and described and illustrated by Matsushima [5], is in fact *C. amazonensis* [128].

***Codinaea aristata*** Maire, Publ. Inst. Bot. Barcelona 3: 15. 1937. (Figure 1).

≡ *Dictyochaeta aristata* (Maire) Aramb. & Cabello, Mycotaxon 34: 681. 1989. (Nom. inval., Art. 41.4).

≡ *Dictyochaeta aristata* (Maire) Whitton, McKenzie & K.D. Hyde, Fungal Divers. 4: 136. 2000.

Typification: SPAIN, Catalonia, Sant Miquel de Cladells, on decaying stem of *Rubus* sp. lying on the ground, date unknown, R. Maire (**holotype** not available). Lectotype illustration: Maire, Fungi Catalaunici: Series altera. Contributions a l’étude de la flore mycologique de la Catalogne. Publ. Inst. Bot. Barcelona 3: 15, Figure 1. 1937 (**lectotype illustration** designated here MBT 10004618, Figure 1).

Habitat and geographical distribution: Saprobe on a stem of *Rubus* sp. in Europe, Spain.

Notes: Maire [1] described *C. aristata* on a decaying stem of *Rubus* sp. lying on the ground close to the torrent near Sant Miquel de Cladells in Catalonia, Spain. The species was characterized with setae 250 μm long and longer, sterile, arising singly or in a group of two, erect, dark brown, thick-walled, several-septate, smooth, unbranched, sterile at the apex; conidiophores up to 100 μm long, in bundles with setae, pale brown, thin-walled, flexuous, 1–2-septate, tapering apically; conidia 12–14 × 2 μm, falcate, aseptate, hyaline, with a setula at each end, 4–6 μm long at the apical end and ca. 1 μm long at the basal end, soon evanescent.

Because the holotype or other authentic material of *C. aristata* could not be found [2] and other collections or cultures of this species are not available, the illustration accompanying the protologue [1] is designated here as a lectotype (Figure 1). *Codinaea aristata* resembles *C. fertilis* [2] and *C. siamensis* [47], but differs in conidia with shorter setulae of a different length.

**Figure 12:**
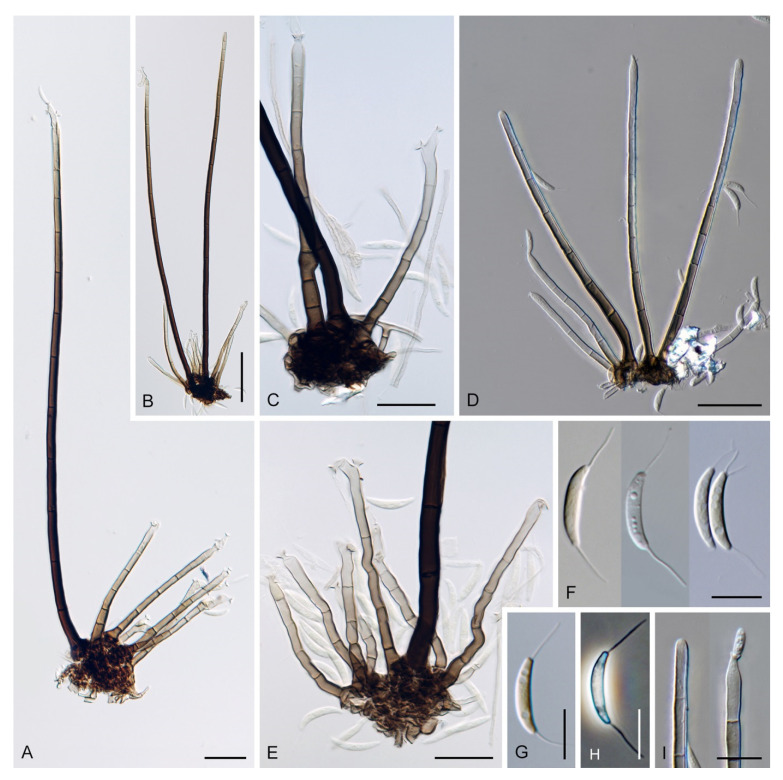
*Codinaea assamica* (CBS 139907 ex-type of *C. acaciae*). (**A**–**E**) Setae with conidiophores (**F**–**H**) conidia (**I**) apical part of the setae. Images: (**A**–**I**) on pine needles on SNA after 4 weeks. Scale bars: (**A**,**D**) 25 μm; (**B**) 50 μm; (C,E) 20 μm; (**F**–**I**) 10 μm.

**Figure 13:**
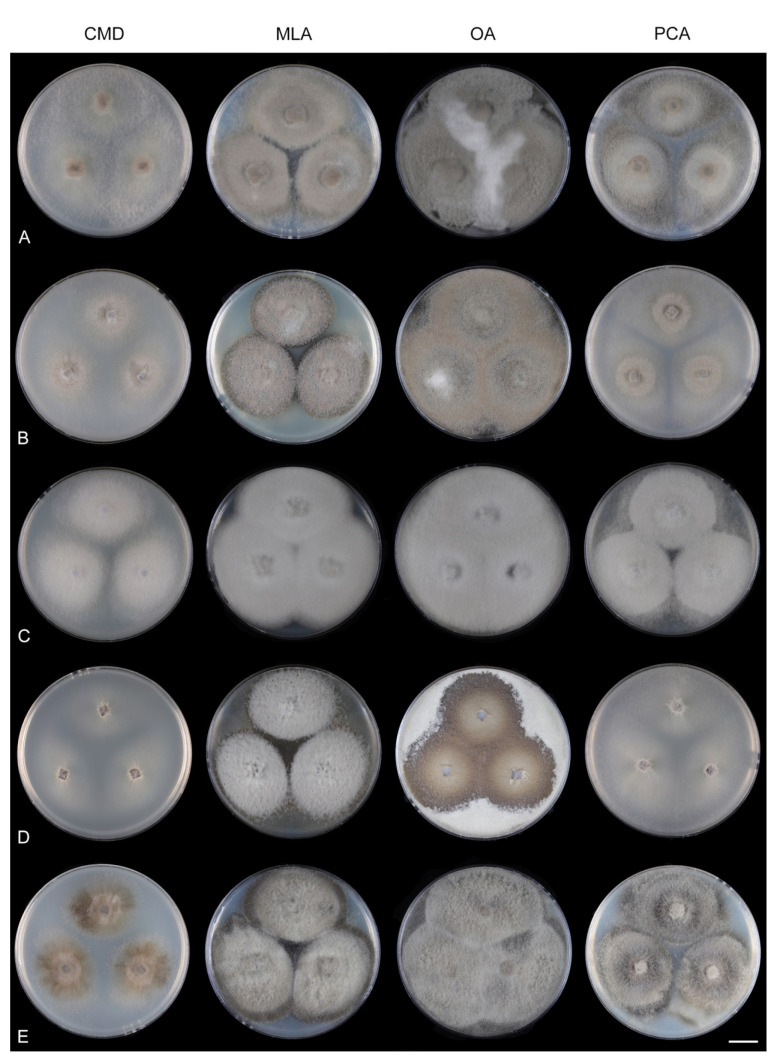
Colony morphology of *Codinaea* spp. after 4 weeks. (**A**) *C. assamica* CBS 139907 ex-type of *C. acaciae* (**B**) *C. fertilis* IMI 233824 (**C**) *C. fertilis* MUCL 15427 (**D**) *C. dwaya* CBS 261.77 ex-type (**E**) *C. siamensis* CBS 194.96. Scale bar: (**A**–**C**) 1 cm.

***Codinaea assamica*** (Agnihothr.) S. Hughes & W.B. Kendr., N. Z. J. Bot. 6: 334. 1968. (Figure 12 and Figure 13).

Basionym. *Menisporella assamica* Agnihothr., Proc. Indian natn Sci. Acad., Part B. Biol. Sci. 56: 99. 1962.

≡ *Dictyochaeta assamica* (Agnihothr.) Aramb., Cabello & Mengasc., Darwiniana 28: 297. 1988. (Nom. inval., Art. 41.4).

= *Codinaea acaciae* Crous & M.J. Wingf., Persoonia 34: 181. 2015.

Culture characteristics: On CMD: colonies 75–78 mm diam, circular, flat, margin fimbriate, sparsely lanose to cobwebby, funiculose at the inoculation block, olivaceous beige, grey-brown centrally, aerial mycelium with numerous colorless exudates, reverse olivaceous beige. On MLA: colonies 48–50 mm diam, circular, raised, margin fimbriate, lanose, grey-brown, olivaceous brown at the margin, reverse dark brown. On OA: colonies 69–70 mm diam, raised, margin fimbriate, lanose, floccose, grey with irregular white patches, dark olivaceous grey at the margin, reverse dark grey. On PCA: colonies 70–72 mm diam, circular, flat, margin fimbriate, cobwebby, lanose at the inoculation block, zonate, pale brown at the centre becoming olivaceous beige toward the periphery, dark olivaceous brown at the margin, reverse of the same colors. Sporulation was abundant on MLA, moderate on PCA and OA, absent on CMD.

Colonies on SNA with pine needles effuse, hairy, brown, mycelium composed of branched, septate, subhyaline to pale brown hyphae 1.5–2.5 μm diam. **Anamorph.** Setae 273–360 μm long, 5–7 μm wide near the swollen base, arise solitary or in groups of two from conspicuous dark brown stromatic cells, erect, straight or flexuous, septate, smooth, unbranched, dark brown, thick-walled, paler and thinner-walled toward the apex, apex pale brown to subhyaline, sterile when young, broadly rounded, later with a terminal or 1–3 lateral phialidic openings. Conidiophores 42–125 μm long, 3.5–4.5(–5) μm wide near the base, macronematous, arise in fascicles of 3–9 from stromatic cells around the base of the setae, unbranched, erect, straight or flexuous or geniculate to undulate, septate, smooth, pale to medium brown, gradually paler toward the apex. Conidiogenous cells 14.5–27 × 3.5–4.5(–5) μm tapering to 1.5–2 μm below collarette, integrated, terminal, mono- or polyphialidic with 1–3 lateral openings while internally septa can be formed, extending percurrently and sympodially, cylindrical, slightly swollen bellow the collarette, pale brown, subhyaline at the apex; collarettes 3.5–4.5 μm wide, 2–2.5 μm deep, funnel-shaped, subhyaline to pale brown. Conidia 14–18 × 2.5–3.5 μm (mean ± SD = 15.9 ± 0.9 × 2.9 ± 0.3 μm), falcate, tapering toward both ends, narrowly rounded apically, slightly truncate at the base with an inconspicuous scar, aseptate, hyaline, with straight or gently curved setula at each end (5.5–)7–13.5 μm long, inserted terminally at the apex, subterminally at the base, conidia accumulate in slimy whitish fascicles. **Teleomorph.** Unknown.

Specimen examined: MALAYSIA, Sarawak, from leaf spots of *Acacia mangium*, May 2014, M.J. Wingfield (ex-type strain of *Codinaea acaciae* CBS 139907).

Habitat and geographical distribution: Saprobe on woody roots, leaf litter, leaf spots and decaying petioles of *Acacia mangium*, *Camelia sinensis*, *Cedrela odorata*, *Cyperus radians*, and other unidentified hosts in Central and South America and Asia: Brazil, Cuba, India, and Malaysia [3,25,36,53,134,135]. Rambelli et al. [35] reported *C. assamica* on fallen leaves of 10 hosts from Africa from Ivory Coast, namely *Anthonotha fragrans*, *Calpocalyx brevibracteatus*, *Chrysophyllum taiense*, *Dialium aubrevillei*, *Hypselodelphys violacea*, *Lophira alata*, *Manniophyton fulvum*, *Memecylon lateriflorum*, *Pentaclethra macrophylla,* and *Piptadeniastrum africanum*.

According to GlobalFungi, the identical sequences were found in two soil samples from the forest in Asia on Malaysian peninsula and Kalimantan [117], and in two root samples from forest in South America in French Guiana [136]. The sites have a tropical climate (MAT avg. 24 °C, MAP avg. 2612 mm).

Notes: Hughes and Kendrick [2] gave a detailed description of *C. assamica* by examining the holotype material of *Menisporella assamica* from India [3]. Unfortunately, no culture derived from the type or other material of *C. assamica* is available. Our study of the ex-type strain of *C. acaciae* CBS 139907 revealed it is remarkably similar to *C. assamica*. Crous et al. [53] isolated *C. acaciae* from leaf spots of *Acacia mangium* in Malaysia and based the description on observations in culture. However, the setae were absent and the conidiophores were somewhat smaller, 15–30 × 3–3.5 μm. Based on our experience, some *Codinaea* species do not form setae readily on agar media and conidiophores may not be fully developed, which can complicate a correct identification. However, on SNA with pine needles, the fungus formed setae arranged in typical bundles with conidiophores growing from stromatic basal cells. Although conidia have one setula at each end, we have rarely observed conidia in culture with two setulae inserted at one end (two conidia in total), a character observed for example in *C. phasma* (this study). Based on a detailed comparison of our observations of *C. acaciae* with *C. assamica* on natural material [2,3] and in culture [25], we concluded that they are conspecific. Therefore, *C. acaciae* is reduced to synonymy with *C. assamica*.

*Codinaea assamica* resembles *C. paniculata* [46], *C. siamensis* [47], and *C. terminalis* [30]. *Codinaea paniculata* mostly occurs in the Holarctic zone and differs in shorter setae, monophialidic conidiogenous cells and conidia with shorter setulae. *Codinaea siamensis* possesses broader conidia, and *C. terminalis* differs in monophialidic conidiogenous cells and larger conidia. *Dictyochaeta plovercovensis* [122], whose culture and DNA data are not available, also resembles *C. assamica* but differs in shorter conidiophores and narrower conidia with shorter setulae.

**Figure 14:**
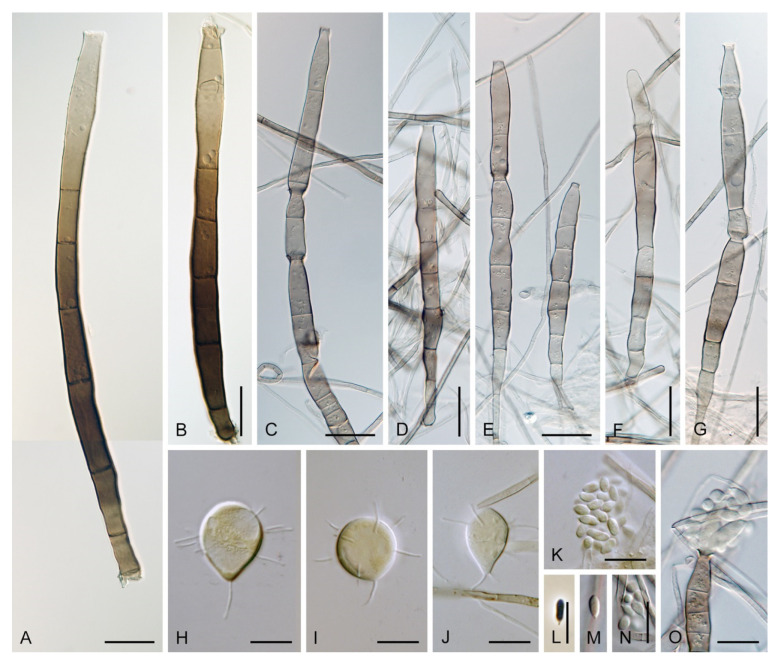
*Codinaea dwaya* (CBS 261.77 ex-type). (**A**–**G**) Conidiophores (**H**–**J**) conidia (**K**–**O**) microconidia. Images: (**A**–**O**) on stems of *U. dioica* on CMA after 8 weeks. Scale bars: (**A**–**G**) 20 μm; (**H**–**O**) 10 μm.

***Codinaea dwaya*** (Subram. & Bhat) Réblová & Hern.-Restr., **comb. nov.** MycoBank MB 842192. (Figure 13 and Figure 14).

Basionym. *Bahusutrabeeja dwaya* Subram. & Bhat, Can. J. Bot. 55: 2204. 1977.

Culture characteristics: On CMD: colonies 75–76 mm diam, circular, flat, margin entire, mucoid, smooth, funiculose at the inoculation block, isabelline, reverse of the same color. On MLA: colonies 59–61 mm diam, circular, raised to slightly convex, margin weakly fimbriate, lanose, floccose, mucoid at the margin, white-grey with an olivaceous brown outer zone, reverse dark olivaceous brown. On OA: colonies 75–78 mm diam, circular, flat to slightly raised, margin fimbriate, lanose becoming mucoid, whitish-beige, pale brown becoming cinnamon-brown centrally when mucoid, reverse dark grey. On PCA: colonies 82–83 mm diam, circular, flat, margin fimbriate, mucoid centrally, cobwebby toward the periphery, funiculose at the inoculation block, pale olivaceous-isabelline centrally, white grey at the margin, reverse whitish. Sporulation was absent on all media.

Colonies on CMA with *U. dioica* stems effuse, hairy, mycelium composed of branched, septate, hyaline to brown hyphae 1–2.5 μm diam. **Anamorph.** Setae absent. Conidiophores (33–)74–254 μm long, tapering toward the base 3–5.5(–8.6) μm wide, gradually widening upwards, 5–10(–11.5) μm wide at the broadest point, macronematous, solitary, unbranched, straight or flexuous, smooth, medium brown becoming dark brown at maturity, darkest in the middle, paler toward the apex and base. Conidiogenous cells 18–66 × 6.5–11.5 μm, tapering to 3–6.5(–7.5) μm below the collarette, integrated, terminal, monophialidic, extending percurrently, subcylindrical with a swollen venter, fulvous, gradually paler toward the apex; collarette 4.5–10 μm wide, 1.5–3 μm deep, pale brown, flared, shallow, indistinct. Conidia of two morphologically distinct types: macroconidia variable in shape and size, the first conidium is broadly oblong or pyriform 15.5–22 × 10.5–14.5 μm (mean ± SD = 18.7 ± 2.1 × 13 ± 2.8 μm), subsequent conidia globose 15–16 μm diam (mean ± SD = 15.5 ± 0.5 μm), thick-walled, aseptate, hyaline, with 6–10 straight or gently curved setulae distributed over the surface, 6–10.5 μm long; microconidia 4.5–6.5 × 2–2.5 μm (mean ± SD = 5.1 ± 0.8 × 2.4 ± 0.2 μm), formed from the same conidiogenous loci, ellipsoidal to oblong, aseptate, hyaline, with a miniature setula at one end 1–1.5 μm long; macroconidia and microconidia accumulate in slimy whitish fascicles. **Teleomorph.** Unknown.

Specimen examined: INDIA, Karnataka, Coorg district, near Abby Falls, on dead twigs of *Coffea arabica*, 10 May 1976, D.J. Bhat (ex-type culture CBS 261.77 = JCM 6357 = IMI 213921).

Habitat and geographical distribution: Saprobe on decaying bark and wood of *Coffea arabica* and other unknown hosts, known from Asia from China and India [120,135,137,138]. The GlobalFungi database did not contain similar sequences (≥98% sequence identity) of this species.

Notes: In the phylogenetic analysis, the ex-type strain of *B. dwaya*, the generic type, clustered in the *Codinaea* clade among species with falcate to ellipsoidal conidia with two or more setulae, setae and variability in conidiophores. Based on this result, *Bahusutrabeeja* is reduced to synonymy with *Codinaea* and a new combination is proposed. The unique characters of *C. dwaya* are globose or pyriform conidia with multiple appendages. Our observations of the ex-type strain of *C. dwaya* are consistent with those of Subramanian and Bhat [120]. In addition, we observed microconidia with a rudimentary appendage, which formed alongside the macroconidia from the same conidiogenous locus (Figure 14K–O).

Two other *Bahusutrabeeja* species, *B. bunyensis* [139] and *B. globosa* [126], closely resemble *C. dwaya* in having globose to subglobose, multi-setulate conidia. *Bahusutrabeeja bunyensis* differs in having smaller conidia with only 2–3 setulae, while *B. globosa* has larger conidia compared to *C. dwaya*. The conidia of *B. angularis* [140] have an angular outline and should be compared with *Nawawia* [141]. Other species currently referred to *Bahusutrabeeja* differ in conidial shape and lack setulae, and their taxonomic treatment needs to be resolved using phylogenetic arguments.

***Codinaea ellipsoidea*** (Z.L. Luo, K.D. Hyde & H.Y. Su) Réblová & Hern.-Restr., **comb. nov.** MycoBank MB 842193.

Basionym. *Dictyochaeta ellipsoidea* Z.L. Luo, K.D. Hyde & H.Y. Su, Fungal Divers. 99: 593. 2019.

For description, illustration, and comparison with morphologically similar species see Luo et al. [29].

Habitat and geographical distribution: A saprobe on submerged wood of unidentified hosts, known only from Asia, China [29]. The GlobalFungi database did not contain similar sequences (≥98%) of this species.

Notes: The species is known from several collections from China. It is characterized by the absence of setae and having dark pigmented, setiform conidiophores terminating into a monophialide with setulate ellipsoidal conidia.

**Figure 15:**
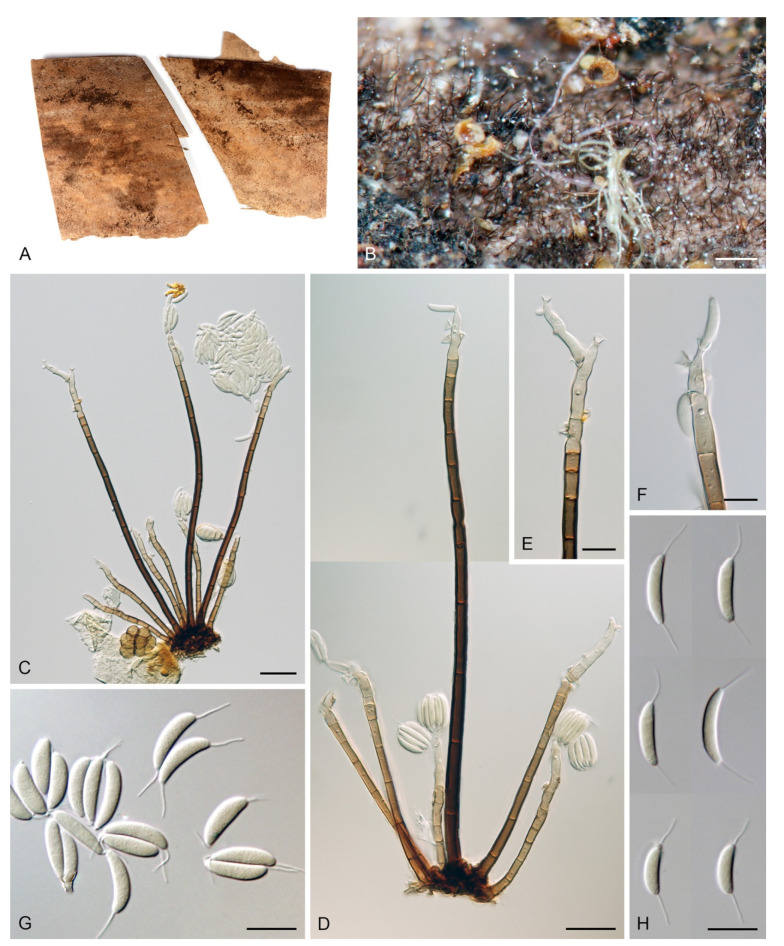
*Codinaea fertilis* (DAOM 93548c isotype). (**A**) Substrate with colonies (**B**) colony on a dead leaf (**C**,**D**) conidiophores (**E**,**F**) apices of setae with phialidic apertures (**G**,**H**) conidia. Images: (**A**–**H**) from nature. Scale bars: (**B**) 500 μm; (**C**) 25 μm; (**D**) 20 μm; (**E**–**H**) 10 μm.

**Figure 16:**
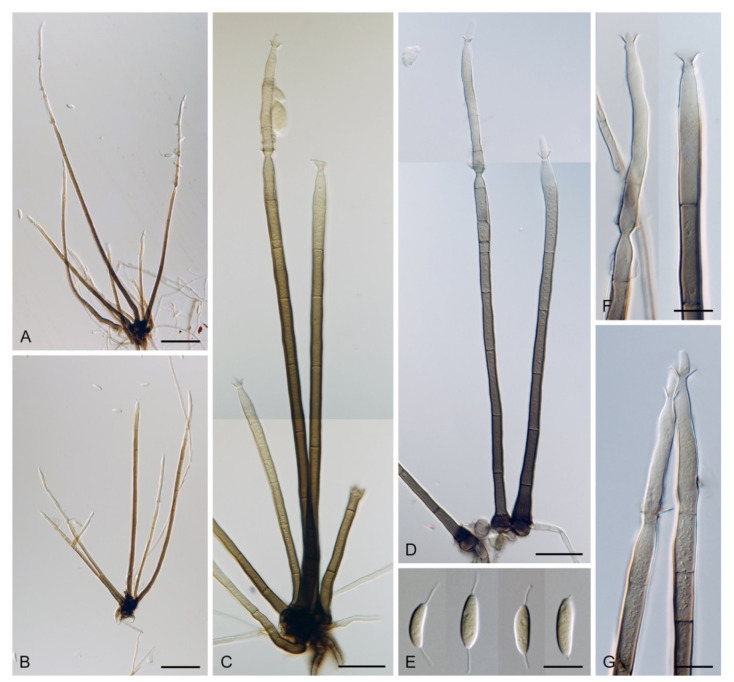
*Codinaea fertilis* (IMI 233824). (**A**–**D**) Setae with conidiophores (**E**) conidia (**F**,**G**) conidiogenous cells at the tip of the setae. Images: (**A**–**G**) on PCA after 6 weeks. Scale bars: (**A**,**B**) = 50 μm; (**C**,**D**) 20 μm; (**E**–**G**) 10 μm.

***Codinaea fertilis*** S. Hughes & W.B. Kendr., N. Z. J. Bot. 6: 347. 1968. (Figure 13, Figure 15 and Figure 16).

≡ *Dictyochaeta fertilis* (S. Hughes & W.B. Kendr.) Hol.-Jech., Folia geobot. phytotax. 19: 426. 1984.

Culture characteristics: On CMD: colonies 75–77 mm, circular, flat, margin fimbriate, lanose, somewhat funiculose centrally, white-beige, reverse olivaceous beige. On MLA: colonies 66–68 mm diam, circular, convex, margin entire, lanose, floccose, with funiculose projections at the centre, becoming finely furrowed, grey-brown with a dark olivaceous brown outer zone, isabelline at the colony margin, reverse dark grey-brown. On OA: colonies >100 mm diam (colony reached the edge of 10 mm Petri dish), circular, flat, margin entire, lanose, olivaceous brown, camel brown toward the periphery with a dark olivaceous grey outer zone, reverse dark grey. On PCA: >100 mm diam (colony reached the edge of 10 mm Petri dish), flat, lanose, cobwebby toward the margin, beige, olivaceous beige at the margin, reverse dark olivaceous grey. Sporulation was abundant on all media after prolonged incubation (>6 weeks).

Colonies on PCA effuse, hairy, mycelium composed of branched, septate, subhyaline to pale brown hyphae 1.5–3 μm diam. **Anamorph.** Setae 152–340 μm long, 4.5–6.6 μm wide near the base, arise solitary or in group of 2–4 from dark brown stromatic cells, erect, straight or flexuous, septate, smooth, unbranched, rarely branched, dark brown, paler, and thinner-walled toward the apex, apex pale brown to subhyaline, with a terminal phialidic opening. Conidiophores 66–146 μm long, 4–5(–5.5) μm wide near the base, macronematous, single or arise in fascicles of 3–7 from dark brown hyphal cells around the bases of the setae, erect, straight or flexuous, septate, smooth, unbranched, occasionally branched in the upper part, pale to medium brown, thick-walled, gradually paler and thinner-walled toward the apex. Conidiogenous cells (15–)30–47 × 4.5–5.5(–6) μm tapering to 2–2.5 μm below the collarette, integrated, terminal, monophialidic, rarely polyphialidic with one lateral opening, extending percurrently (with up to five percurrent proliferations) or sympodially, subcylindrical, slightly swollen bellow the collarette, pale brown, subhyaline at the apex; collarettes 5.5–7.5 μm wide, 2.5–3 μm deep, funnel-shaped, subhyaline to pale brown. Conidia (10–)11–14.5 × 3.5–4.5 μm (mean ± SD = 12.8 ± 1.1 × 3.9 ± 0.4 μm), falcate to ellipsoidal-fusiform, slightly truncate at the base, with straight or gently curved setula at each end, 3.5–6.5(–9) μm long, conidia accumulate in slimy whitish fascicles. **Teleomorph.** Unknown.

Specimens examined: FRANCE, Guadeloupe region, on root of *Musa* sp., 1966, J. Brun (culture CBS 242.66 = MUCL 15427). NEW ZEALAND, Auckland province, Waitakere Range, Waiatarua, on basis of dead leaves of *Rhopalostylidis sapidae*, 8 May 1963, S.J. Hughes 706c (**isotype** of *Codinaea fertilis* DAOM 93548c). NEW ZEALAND, on root of *Betula* sp., 1979, L. Mattson LEV 13960 (culture IMI 233824).

Habitat and geographical distribution: Based on literature records, *C. fertilis* commonly occurs on decaying leaves, petioles, palm fronds, bark, wood, woody fruits and roots of *Alchornea cordifolia*, *Calathea stromata*, *Carya ovata*, *Cedrela odorata*, *Ctenanthe oppenheimiana*, *Euterpe oleracea*, *Fraxinus excelsior*, *Freycinetia* sp., *Maranta bicolor*, *Nothofagus menziesii*, *Pandanus tectorius*, *Pandanus* sp., *Quercus ilex*, *Q. robur*, *Quercus* sp., *Rhopalostylis sapida*, *Stromanthe sanguinea* and other unknown hosts in terrestrial and freshwater habitats. The species was reported from Africa, Australasia, Europe, South and North America, and Southeast Asia: Brazil, Brunei, Canada, Czech Republic, China, Ivory Coast, Malaysia, New Zealand, The Netherlands, Philippines, Slovak Republic, and USA [2,25,26,30,35,36,37,44,121,142,143,144,145,146,147,148]. Strains examined in this study were isolated from roots of *Betula* sp. from New Zealand and *Musa* sp. from France (Guadeloupe).

According to GlobalFungi, of all the fungi studied, this species is the most abundant worldwide in different climatic regions. The identical sequences were found in 655 samples, originating from 46 studies, collected on all continents including Antarctica. It was found in Africa (Ethiopia, Madagascar, South Africa, St. Helena island, Zambia, Zimbabwe), Asia (China, Malaysia, Papua New Guinea), East and South Australia, Europe (Northern Spain, Estonia, Germany, Austria, the Netherland, Slovenia), Hawaii, Central America and Central America (Mexico, Costa Rica, Puerto Rico, Trinidad and Tobago, Panama), North America (Oregon, Kansan, South Carolina, Minnesota, Tennessee, Luisiana, Georgia, Michigan), South America (Argentina). The sample types were soil (58%), rhizosphere (20%), root (15%) and shoot (6%) collected in cropland (37%), forest (30%), grassland (23%), woodland (7%), shrubland (2%) and desert (0.2%) habitats. The locations, with very few exceptions, have a tropical or temperate climate (MAT avg. 15 °C, MAP avg. 1066 mm).

*Codinaeella fertilis* has also been recorded as a plant pathogen causing root rot of *Lolium perenne*, *Medicago sativa*, *Trifolium repens*, *T. praetense*, *T. subterraneum* and other important perennial pasture legumes in Australasia and North America: New Zealand and USA e.g., [54,56,149,150,151].

Notes: Hughes and Kendrick [2] described *C. fertilis* from leaf bases of *Rhopalostylidis sapidae* in New Zealand with conidia 9–15.4 × 2–3 μm and setulae 5–10 μm long. The revision of the isotype (Figure 15) revealed certain variability in the shape and width of conidia. Although conidia were mostly falcate, 13–15 × 2.5(–3) μm, some conidia were also ellipsoidal-falcate and somewhat wider, 10–13 × (2.5–)3(–3.5) μm. Our observations of *C. fertilis* in culture are consistent with those of the isotype; conidia of the strain IMI 233824 were predominantly ellipsoidal-falcate, however wider (3.5–4.5 μm) when grown in culture (Figure 16). Although *C. fertilis* sporulated abundantly on all four media, conidiophores associated with setae were formed only on MLA and PCA.

In characters of conidia, *C. fertilis* is comparable to other species such as *C. aristata* [1], *C. paniculata* [46], and *Dictyochaeta plovercovensis* [122]. *Codinaea aristata* differs by narrower conidia with two asymmetrical setulae at each end, *C. paniculata* possesses conidia longer in their upper range with longer setulae and *D. plovercovensis* has narrower conidia with longer setulae.

According to literature, *C. fertilis* is a common species with a worldwide distribution. It is possible, however, that the arrangement of setae and conidiophores in the bundles may have been misinterpreted or overlooked. In such cases, *C. fertilis* could be confused with *Ca. lutea* or *Ca. parvilobata* (this study). They differ in setae, which grow independently and conidiophores are interspersed among them. The suspicion that some strains might be misidentified as *C. fertilis* was confirmed by molecular data. Three of the analysed strains, initially identified as *C. fertilis* (CBS 624.77, ICMP 14613, and ICMP 15540), were confirmed as members of the genus *Codinaeella* and represent *Ca. lutea*.

**Figure 17:**
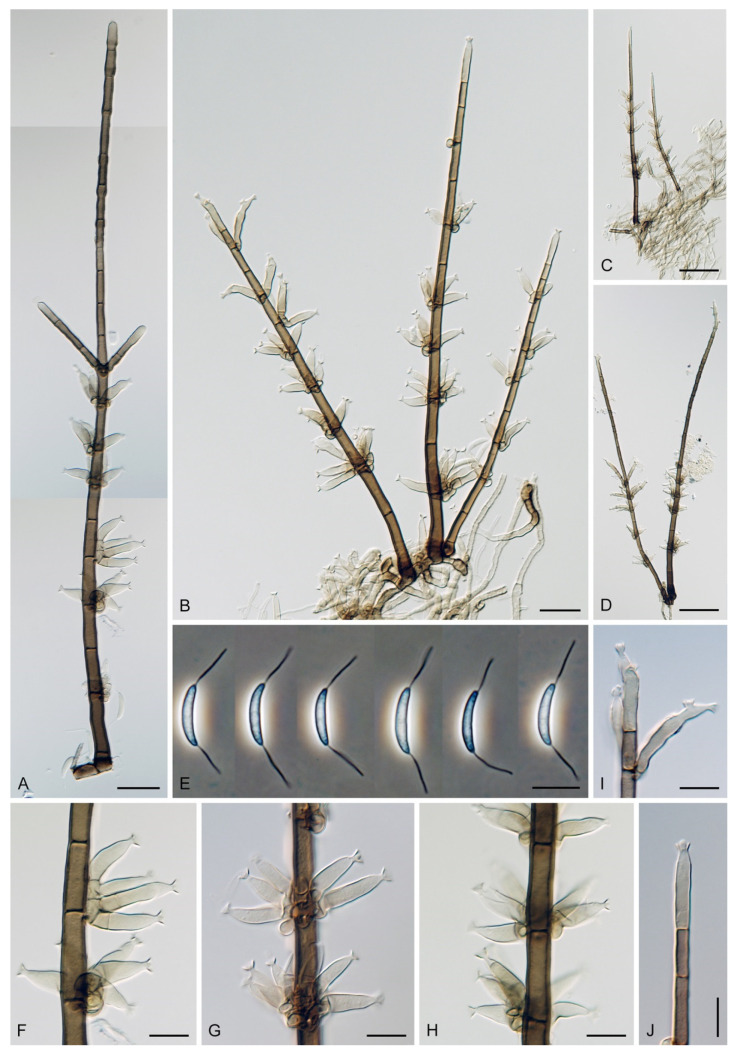
*Codinaea gonytrichodes* (CBS 593.93). (**A**–**D**) Conidiophores (**E**) conidia (**F**–**H**) conidiogenous cells arising from collar hyphae (**I**,**J**) conidiogenous cells at the tip of the conidiophore. Images: (**A**–**J**) on OA after 8 weeks. Scale bars: (**A**,**B**) 20 μm; (**C**,**D**) 50 μm; (**E**–**J**) 10 μm.

***Codinaea gonytrichodes*** Shearer & J.L. Crane, Mycologia 63: 245. 1971. (Figure 11 and Figure 17).

≡ *Codinaeopsis gonytrichodes* (Shearer & J.L. Crane) Morgan-Jones [as “*gonytrichoides*”], Mycotaxon 4: 167. 1976.

≡ *Dictyochaeta gonytrichodes* (Shearer & J.L. Crane) Kuthub. & Nawawi [as “*gonytrichoides*”], Mycol. Res. 95: 845. 1991.

≡ *Dictyochaetopsis gonytrichodes* (Shearer & J.L. Crane) Whitton, McKenzie & K.D. Hyde [as “*gonytrichoides*”], Fungal Divers. 4: 156. 2000.

Culture characteristics: On CMD: colonies 45–50 mm diam, circular, raised, margin entire to fimbriate, lanose, floccose, sparsely funiculose, cobwebby at the margin, whitish-brown, reverse dark brown. On MLA: colonies 75–78 mm diam, circular, raised, margin lobate, lanose, floccose, funiculose at the inoculation block, zonate, mouse grey centrally, paler toward the periphery dark olivaceous grey at the margin, aerial mycelium with numerous miniature colorless exudates, reverse dark grey to black. On OA: colonies 72–75 mm diam, circular, flat, margin lobate, cobwebby centrally, lanose toward the periphery, brown at the colony centre, beige-grey toward the periphery, reverse dark beige centrally, dark grey to black toward the margin. On PCA: colonies 75–76 mm diam, circular, flat, raised centrally, margin fimbriate, lanose, floccose, cobwebby at the margin, becoming mucoid locally, whitish-beige, olivaceous brown at the margin, reverse dark olivaceous brown. Sporulation was abundant on MLA, OA, and PCA, sparse on CMD.

Colonies on OA effuse, hairy, mycelium composed of branched, septate, subhyaline to brown hyphae that become encrusted upon aging, 2.5–4 μm diam. **Anamorph.** Setae absent. Conidiophores 160–355 μm long, 4–7 μm wide above the swollen base, gradually tapering toward the apex, macronematous, arise singly or in groups of 2–3 from knots of hyphal cells, erect, straight or slightly flexuous, septate, smooth, brown, nodose hyphae are formed laterally below the septa, pale to medium brown. Conidiophores unbranched, sometimes branched in the upper part; branches 28–45(–61) × 3–5 μm arise from the nodose and collar-like hyphae, apex pale brown to subhyaline, sterile or terminating into a mono- or polyphialide. Conidiogenous cells 11.5–17 × 4–6 μm tapering to 1.5–2 μm below the collarette, mono- occasionally polyphialidic with 1–3 lateral openings, extending sympodially, discrete, lateral, arise as stalked or sessile phialides on collar-like and nodose hyphae or directly on conidiophores, mostly in groups of (2–)3–6(–7), or integrated, terminal at the apex of the conidiophore or phialide-bearing branches, subcylindrical to lageniform, pale brown, subhyaline at the apex; collarette 3–4 μm wide, 1.5–2(–2.5) μm deep, funnel-shaped, subhyaline to pale brown. Conidia 12–14.5 × 2–2.5(–3) μm (mean ± SD = 13.4 ± 0.6 × 2.4 ± 0.3 μm), falcate, tapering toward both ends, slightly truncate at the base with an inconspicuous scar, aseptate, hyaline, with straight or gently curved setula at each end 7.5–11.5 μm long, inserted terminally at the apex, subterminally at the base, conidia accumulate in slimy whitish fascicles. **Teleomorph.** Unknown.

Specimen examined: JAPAN, Saitama Prefecture, Ogawa-machi, Seikoji Temple, decaying plant material, 4 September 1993, W. Gams & G. Okada (culture CBS 593.93).

Habitat and geographical distribution: Saprobe on decaying leaves, seeds, herbaceous stems and fruits of *Acer* sp., *Carya* sp., *Castanopsis cuspidata*, *Liriodendron tulipifera*, *Ochroma pyramidale*, *Rubus* sp. and other unknown hosts in freshwater and terrestrial habitats. The species is found in Asia and South and North America: Brazil, Japan, Malaysia and USA [6,21,35,37,42,127,152,153]. Occasionally it occurs on submerged wood of *Ochroma pyramidale* (balsa wood) in USA [6]. Rambelli et al. [35] recorded *C. gonytrichodes* on leaf litter of *Combretum dolichopetalum*, *Manniophyton fulvum*, *Newtonia duparquetiana,* and *Xylopia acutiflora* from Africa, Ivory Coast.

According to GlobalFungi, the identical sequences were found in 10 soil samples from forests in Asia in Iran and East China [115,117], and North America in Ohio and Georgia [119,154]. The sites have a temperate or humid continental climate (MAT avg. 15 °C, MAP avg. 1096 mm).

Notes: For descriptions and illustrations on natural substrate, see Shearer and Crane [6], Morgan-Jones [42] and Kuthubutheen and Nawawi [21]. The morphological characteristics of conidiophores, conidiogenous cells and conidia of the strain CBS 593.93 match the protologue in all details and are well-comparable with other known specimens of this species. Only conidia of CBS 593.93 were slightly broader in vitro, 2–2.5(–3) μm wide, compared to those in nature, 1.2–2.3 μm [6], 1.5–2.5 μm [42], and 1.5–2.5 μm [21].

*Codinaea gonytrichodes* is unique among other *Codinaea* in having nodose hyphae at the septa, from which lateral phialides and branches grow. In older cultures, the apex is modified into a polyphialide and discrete, lateral polyphialides frequently occur in the upper part of the conidiophore. *Dictyochaetopsis polysetosa* [127] closely resembles *C. gonytrichodes* in conidial morphology and branched conidiophores, but branches of the former species are setose, acicular, dark brown to black, arise in verticilli from nodose hyphae in the upper part of the conidiophore and remain sterile. *Dictyochaeta pahangensis* [21] is similar to *C. gonytrichodes* in having knots of hyphae at the septa along the conidiophore axis, but differs in the absence of branches, longer conidia and nodose hyphae that are bulbose and dark brown with 1–2 discrete phialides.

***Codinaea lignicola*** (Z.L. Luo, H.Y. Su & K.D. Hyde) Réblová & Hern.-Restr., **comb. nov.** MycoBank MB 842194.

Basionym. *Dictyochaeta lignicola* Z.L. Luo, H.Y. Su & K.D. Hyde, Fungal Divers. 99: 595. 2019.

For description, illustration and comparison of *C. lignicola* with similar species, see Luo et al. [29].

Habitat and geographical distribution: Saprobe on submerged wood, known only from Asia, China [29]. According to GlobalFungi, the identical sequences were found in 12 samples of rhizosphere soil (92%) or soil (8%) collected in forest (92%) or grassland (8%) habitats in Asia in North East China and South Korea [155,156]. The locations have a cold and humid climate (MAT avg. 8 °C, MAP avg. 1285 mm).

Notes: Among *Codinaea* species, *C. lignicola* resembles *C. ellipsoidea* with simple, dark pigmented conidiophores terminated by monophialidic conidiogenous cells and absence of setae, but differs in having falcate vs. ellipsoidal conidia.

***Codinaea pandanicola*** (Tibpromma & K.D. Hyde) Réblová & Hern.-Restr., **comb. nov.** MycoBank MB 842195.

Basionym. *Dictyochaeta pandanicola* Tibpromma & K.D. Hyde, Fungal Divers. 93: 127. 2018.

For description, illustration of *C. pandanicola* and its comparison with similar species, see Tibpromma et al. [48].

Habitat and geographical distribution: Saprobe on decaying leaves of *Pandanus* sp., known only from Southeast Asia, Thailand [48]. According to GlobalFungi, the identical sequences were found in 12 soil and one root samples from forests of Central America and the Caribbean (10 samples, Puerto Rico, Panama, Trinidat and Tobago, French Guiana) [119], North America (1 sample, Georgia) [157] and Asia (1 sample, Malaysia) [158]. The locations have tropical climate (MAT avg. 21 °C, MAP avg. 2955 mm).

Notes: Although the description of *C. pandanicola* did not include setae, on the photograph accompanying the protologue ([48], Figure 95b), a dark brown, thick-walled seta (broken) terminating into a phialide is accompanied by shorter, paler, and thinner-walled conidiophores terminating into mono- or polyphialides.

**Figure 18:**
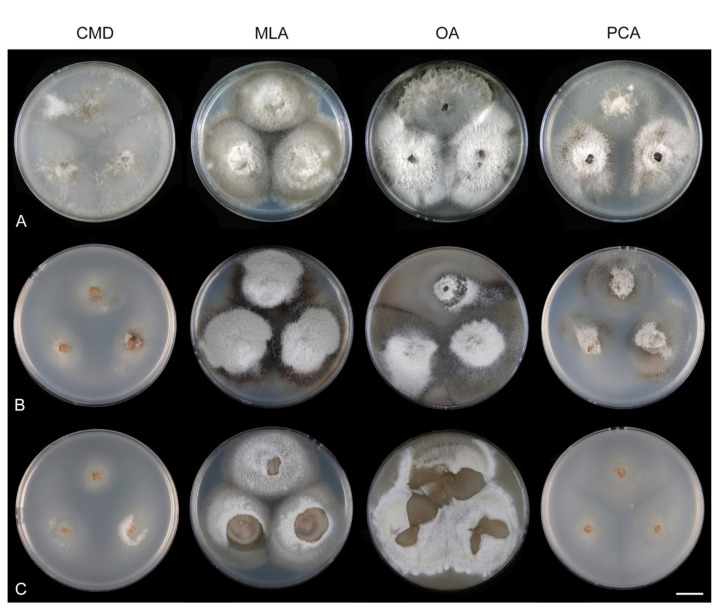
Colony morphology of *Codinaea paniculata* after 4 weeks. (**A**) CBS 145098 ex-type (**B**) CBS 127692 (**C**) MUCL 34876. Scale bar: (**A**–**C**) 1 cm.

***Codinaea paniculata*** Réblová & J. Fourn., MycoKeys 74: 14. 2020. (Figure 18).

For description, characteristics in culture, illustrations, and comparison with similar species, see Réblová et al. [46].

Habitat and geographical distribution: Saprobe on decaying wood and submerged leaves of *Alnus glutinosa*, *Fraxinus excelsior* and other unidentified hosts in Europe in France and United Kingdom [46]. Based on comparison of the ITS sequences of the ex-type strain with environmental sequences deposited in GenBank, *C. paniculata* was also isolated as a root endophyte from a beach grass *Elymus mollis* in North America in the USA, Oregon (ITS: KU838460, KU839605) [159] and from soil samples from an ancient woodland in the United Kingdom (ITS: KM374380) [160].

According to GlobalFungi, the identical sequences were found in 111 samples from soil (64%) or roots (36%) in grassland (43%), forest (38%), cropland (10%), or woodland (8%) habitats. Ninety-two samples originate from Europe (Belgium, Estonia, Germany, Monte Negro, Northern Spain, South Slovenia, Sweden, Wales), five samples from Africa (St. Helena island), one from Asia (Malaysia), five samples from South America (North Argentina), and eight samples were from Southeast Australia and Tasmania. The samples originate from 13 studies and the sites have temperate climate (MAT avg. 10 °C, MAP avg. 881 mm).

Specimens examined: FRANCE, Ariège, Pyrénées Mts., Rimont, La Maille brook, alt. 550 m, 28 May 2018 (incubated in moist chamber for 1 week), on submerged decaying wood, J. Fournier M.R. 3950 (**holotype** PRA-16319, ex-type culture CBS 145098). FRANCE, Ariège, Pyrénées Mts., Rimont, Le Baup stream, ca. 1.5 km from the village along D18 road, alt. 550 m, 12 June 2009, on submerged wood of *Fraxinus excelsior*, J. Fournier J.F. 09153 (PRA-16320, culture CBS 127692), *Ibid*., 23 May 2008, on submerged wood of *Alnus glutinosa*, J. Fournier & M. Delpont J.F. 08124 (PRA-16321, culture CBS 126573). UNITED KINGDOM, Liverpool, University Campus Liverpool, 1992, on submerged dead leaf in a pool, G.L. Hennebert (culture MUCL 34876).

Notes: *Codinaea paniculata* represents the C1 morphotype. We observed variability among strains of *C. paniculata* from France (CBS 127692, CBS 145098) and the United Kingdom (MUCL 34876), which is shown in Figure 18. None of the three isolates produced pigments diffusing into the agar. The most prominent growth and abundant aerial mycelium, sometimes with submerged growth, was observed on MLA and OA. The growth on CMD and PCA was rather sparse.

**Figure 19:**
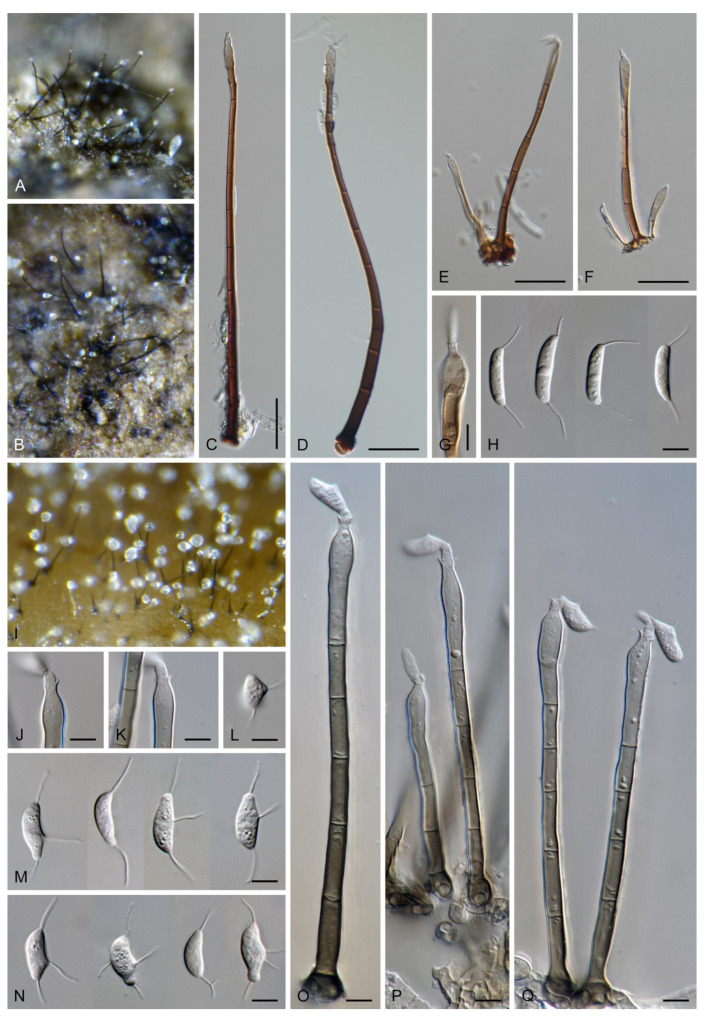
*Codinaea phasma* (CBS 147516 ex-type). (**A**,**B**,**I**) Colonies (**C**,**D**) setae (**E**,**F**) setae with conidiophores (**G**) conidiogenous cell (**H**,**M**,**N**) conidia (**J**,**K**) apices of the conidiogenous cells, conidiogenous locus slightly protruding above the collarette (**L**) conidium, from the top view (**O**–**Q**) conidiophores. Images: (**A**–**H**) from nature (**I**–**Q**) on stems of *U. dioica* on CMA after 4 weeks. Scale bars: (**C**–**F**) 25 μm; (**G**,**H**,**J**–**Q**) 5 μm.

***Codinaea phasma*** Hern.-Restr. & Réblová, **sp. nov.** MycoBank MB 842198. (Figure 11 and Figure 19).

Etymology: *Phasma* (L) ghost, phantom, referring to the mysterious appearance of the multisetulate conidia in culture.

Typification: PUERTO RICO, on decaying twig of an unidentified plant, 19 July 2018, M. Hernández-Restrepo M.H.R. 18014 (**holotype** CBS H-24747, culture ex-type CBS 147516).

Description on the natural substrate: Colonies effuse, hairy, black. **Anamorph.** Setae 160–380 μm long, 5–10 μm wide near the base, gradually tapering upwards, arise solitary or in groups of two from dark brown stromatic cells, erect, straight or flexuous, septate, smooth, dark brown, thick-walled, paler, and thinner-walled toward the apex, unbranched, apex pale brown with a terminal or one to several lateral phialidic openings. Conidiophores 35–97 μm long, 2.5–4.5 μm wide near the base, macronematous, single or arise in fascicles of 2–5 from stromatic cells around the base of the setae, erect, straight or slightly bent, septate, smooth, medium to pale brown, paler toward the apex.

Conidiogenous cells 16–43 × 2.5–4 μm, 5–7 μm wide at the broadest point, tapering to 1.5–2.5 μm below the collarette, integrated, terminal, mono- or polyphialidic, occasionally extending percurrently, subcylindrical, swollen bellow the collarette, pale brown; collarettes 2–3.5 μm wide, 2–3 μm deep, funnel-shaped to slightly tubular, pale brown. Conidia 13.5–18 × 3–4 μm (mean ± SD = 15.1 ± 3.8 × 1.2 ± 0.4 μm), oblong to falcate, slightly truncate at the base with an inconspicuous basal scar, aseptate, hyaline, with a straight or gently curved setula at each end 5–13 μm long, inserted terminally at the apex, subterminally at the base, conidia accumulate in whitish slimy fascicles. **Teleomorph.** Unknown.

Culture characteristics: On CMD: colonies 27–30 mm diam, circular, flat to slightly convex, margin fimbriate, lanose, floccose, whitish-beige with a darker olivaceous beige zone at the margin, pale ochre pigment diffusing into the agar, reverse of the same colors. On MLA: colonies 14–17 mm diam, circular, raised, margin lobate, lanose becoming mucoid, aerial mycelium restricted to the centre of the colony, deeply furrowed with nearly cerebral-like folds, whitish centrally, pink-beige toward the margin, pale apricot pigment diffusing into the agar, reverse apricot. On OA: colonies 20–22 mm diam, circular, raised, margin undulate, weakly furrowed, lanose, aerial mycelium with colorless exudates, whitish-grey, olivaceous to mouse grey toward the margin with a whitish outer zone, pale ochre pigment diffusing into the agar, reverse apricot-grey. On PCA: colonies 42–55 mm diam, circular, slightly convex centrally with flat margin, margin entire to weakly fimbriate, lanose, whitish centrally, brown to olivaceous-brown toward the periphery, darker at the margin, reverse dark brown. Sporulation was abundant on MLA, OA, and PCA, sparse on CMD.

Colonies on CMA with *U. dioica* stems effuse, hairy, mycelium composed of branched, septate, hyaline to pale brown hyphae 2–4.5 μm diam. Setae, conidiophores and conidiogenous cells similar to those from nature. Setae 97–236 μm long, 3.5–6 μm above the swollen base, dark olivaceous brown, paler toward the apex, apex pale brown to subhyaline, with a terminal phialidic opening. Conidiophores 44–104 μm long, 3.5–7 μm wide above the base, arise singly or in groups, interspersed among the setae, medium brown to olivaceous brown, paler toward the apex. Conidiogenous cells 24–34 × 3.5–5 μm, 4.5–7.5 μm wide at the broadest part, tapering to 1.5–4 μm below the collarette; collarette 2–6 μm wide, 1.5–8 μm deep, funnel-shaped to tubular, subhyaline. Conidia 12–18.5 × 4.5–8 μm (mean ± SD = 14.3 ± 5.6 × 1.5 ± 1.0 μm), irregularly ellipsoidal, tapering toward both ends, sometimes slightly contracted near the base, basal end truncate to obtuse with an inconspicuous basal scar, aseptate, hyaline, with 3(–5), straight or gently curved setulae 3.5–11 μm long, inserted terminally at the apex, subterminally at the base and also dorsally and ventrally, accumulate in slimy whitish fascicles.

Habitat and geographical distribution: Saprobe on decaying wood, known only from the Caribbean from Puerto Rico. According to GlobalFungi, the identical sequences were found in two soil samples from forest in Puerto Rico [118]. The sites have tropical climate (MAT avg. 22.45 °C, MAP avg. 3013).

Notes: On natural substrate, *C. phasma* forms typical bundles of setae and conidiophores and oblong to falcate conidia with a single, simple setula at each end. When grown in culture, the conidia become irregularly ellipsoidal, slightly inflated in the middle and possess up to five setulae inserted also dorsally and ventrally and occasionally they can branch. It resembles *C. terminalis*, but the later species differs from *C. phasma* in longer, falcate conidia. In the present phylogeny, the two species are shown as unrelated lineages.

**Figure 20:**
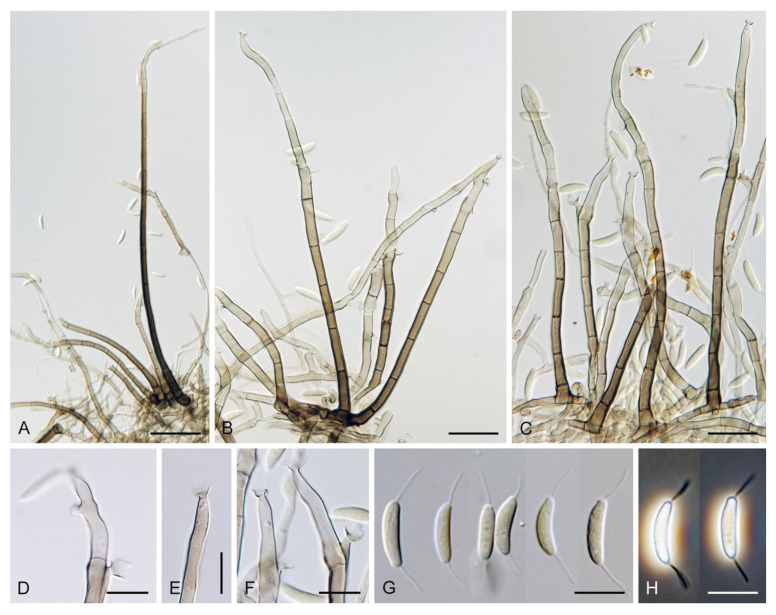
*Codinaea siamensis* (CBS 194.96). (**A**,**B**) Setae with conidiophores (**C**) conidiophores (**D**–**F**) conidiogenous cells with collarettes (**G**,**H**) conidia. Images: (**A**–**H**) on MLA after 6 weeks. Scale bars: (**A**) 50 μm; (**B**,**C**) 20 μm; (**D**–**H**) 10 μm.

***Codinaea siamensis*** (J. Yang, K.D. Hyde & J.K. Liu) Réblová & Hern.-Restr., **comb. nov.** MycoBank MB 842199. (Figure 13 and Figure 20).

Basionym. *Dictyochaeta siamensis* J. Yang, K.D. Hyde & J.K. Liu, Mycol. Prog. 15: 1159. 2016.

Culture characteristics: On CMD: colonies 35–42 mm diam, circular, flat, margin fimbriate, lanose becoming cobwebby, mucoid at the margin, beige-brown, reverse of the same color. On MLA: colonies 63–66 mm diam, circular, raised, margin entire, furrowed, lanose, funiculose, pale olivaceous grey, grey-brown at the margin, reverse dark brown. On OA: colonies 77–80 mm diam, circular, slightly raised, margin lobate, lanose, grey, olivaceous grey-brown at the margin, reverse dark grey to nearly black. On PCA: colonies 76–80 mm diam, circular, flat, margin entire, lanose to cobwebby, floccose, grey-brown, olivaceous brown at the margin, reverse dark olivaceous brown. The strain sporulated only on MLA after prolonged incubation (>6 weeks).

Colonies on MLA effuse, hairy, mycelium composed of branched, septate, hyaline to pale brown hyphae 1.5–3 μm diam. **Anamorph.** Setae 240–330 μm long, 6.5–9 μm wide near the base, arise singly or in groups of 2(–3), erect, straight or flexuous, septate, smooth, dark brown, thick-walled, paler and thinner-walled toward the apex, unbranched, apex pale brown to subhyaline, with a terminal or 1–2 lateral phialidic openings. Conidiophores 67–125(–153) μm long, 3.5–5 μm wide near the base, macronematous, single or arise in fascicles of 3–6 around the base of the setae, unbranched, erect, straight or flexuous, septate, smooth, medium brown, paler toward the apex. Conidiogenous cells 12.5–30 × 3.5–4.5(–5) μm tapering to 1.5–2.5 μm below the collarette, integrated, terminal, mono- or polyphialidic with 1–3 lateral apertures while internally septa can be formed, extending percurrently and sympodially, cylindrical, pale brown, subhyaline at the apex; collarettes 3.5–5 μm wide, 2–2.5(–3) μm deep, funnel-shaped, subhyaline, apical part evanescent with age. Conidia 11.5–14.5 × 2.5–4 μm (mean ± SD = 12.8 ± 0.8 × 3.1 ± 0.3 μm), suballantoid to oblong, slightly curved, asymmetrical, slightly obtuse at the base with an inconspicuous basal scar, aseptate, hyaline, with straight or gently curved setula at each end (4.5–)5–8.5(–10) μm long, inserted terminally at the apex, subterminally at the base, conidia accumulate in slimy whitish fascicles. **Teleomorph.** Unknown.

Specimen examined: PAPUA NEW GUINEA, Madang Province, Finisterre Range, soil in the tropical rain forest, November 1995, A. Aptroot, (culture CBS 194.96).

Habitat and geographical distribution: This species occurs in soil, on submerged leaves of *Pandanus* sp. and decaying wood of an unidentified host, and is known from Australasia and Southeast Asia: Papua New Guinea and Thailand ([47,48], this study).

According to GlobalFungi, the identical sequences were found in 55 samples of soil (89%) or root (11%) collected in forest (98%) and freshwater aquatic (2%) habitat in Central America and the Caribbean (30 samples, Puerto Rico, Panama), South America (eight samples, Brazil, French Guyana, Trinidad and Tobago), Asia (12 samples, South China, Papua New Guinea, Thailand, Malaysia), Australia (four samples, Tasmania), Africa (one sample, Madagascar) and North America (one sample, Minnesota), originating from 13 studies. The localities mostly have tropical or temperate climate (MAT avg. 23 °C and MAP avg. 2319 mm).

Notes: *Codinaea siamensis* has been reported from two collections, which differ in shape and length of conidia and setulae. In the ex-type strain MFLUCC 15-0614, conidia were described as falcate, 15.5–21 × 2.5–4 μm with setulae 7–12 μm long [47], while in the other isolate MFLUCC 16-0371, conidia were allantoid, cylindrical or long fusiform and shorter, 8–17 × 2–5 μm with setulae 1–10 μm long [48]. Conidia observed in the strain CBS 194.96 on MLA correspond to the shape and size of conidia given for the non-type collection of *C. siamensis* by Tibpromma et al. [48]. The conidiophores formed in culture were longer than those from nature due to the phialides, which frequently elongated percurrently. The ITS sequences of all three strains of *C. siamensis* are nearly identical and correlate with 99.57% sequence identity, which is equal to a difference of two base pairs between two sequences. The *tef1-α* sequence identity between MFLUCC 16-0371 and CBS 194.96 was 99.78%; the *tef1-α* sequence of the ex-type strain was not available.

*Codinaea paniculata* [46] is similar to *C. siamensis*, but differs in shorter setae and narrower conidia. *Codinaea siamensis* is also comparable to *C. terminalis*, which differs in shorter setae, monophialidic conidiogenous cells and longer conidia in their upper range.

***Codinaea terminalis*** (C.G. Lin & K.D. Hyde) Réblová & Hern.-Restr., **comb. nov.** MycoBank MB 842200.

Basionym. *Dictyochaeta terminalis* C.G. Lin & K.D. Hyde, Mycosphere 10: 672. 2019.

For description and illustration, see Lin et al. [30].

Habitat and geographical distribution: Saprobe on decaying leaves, known so far from Asia, China [30]. According to GlobalFungi, the identical sequences were found in two soil samples from the cropland and forest habitats in South China [161,162]. The localities have a temperate climate (MAT avg. 20 °C, MAP avg. 1719 mm).

Notes: *Codinaea terminalis* is characterized by setae and conidiophores growing together in bundles (C1 morphotype) and is similar to *C. assamica* and *C. siamensis*. However, the two latter species differ in having longer setulae and wider conidia. For detailed comparison with other species, see Table S4.

***Codinaea vermispora*** (Z.F. Yu & R.F. Castañeda) Réblová & Hern.-Restr., **comb. nov.** MycoBank MB 842201.

Basionym. *Phialolunulospora vermispora* Z.F. Yu & R.F. Castañeda, MycoKeys 76: 23. 2020.

For description and illustration, see Zheng et al. [50].

Habitat and geographical distribution: The species was isolated from submerged leaves of a dicotyledonous plant and is known only from Asia, China [50]. The GlobalFungi database does not contain similar (≥98%) sequences of this species.

Notes: *Codinaea vermispora* was initially accommodated in the monotypic genus *Phialolunulospora* [50] and distinguished from *Codinaea* by vermiform to sigmoid conidia bearing an eccentric basal appendage. Although the authors emphasized the single basal appendage of the conidium, the photograph ([50], Figure 2) shows that the tip of the conidium is strongly attenuated and the transparency and demarcation of this region from the rest of the conidium is identical to the basal appendage. Phylogenetic analysis suggests placement of this species in the strongly supported *Codinaea* clade. Characteristics of conidia (shape, size and the basal appendage) cause *C. vermispora* to be well distinguished among other members of the genus.

#### 3.5.2. The Genus Codinaeella

***Codinaeella*** Réblová & Hern.-Restr., **gen. nov.** MycoBank MB 842000.

Type species. *Codinaeella minuta* (Tubaki) Réblová & Hern.-Restr.

Etymology: *Codinae*- and -*ella* (L) diminutive but here used as a name-forming suffix, referring to fungi morphologically similar to *Codinaea*.

Description: Colonies on natural substrate effuse, lanose, brown to reddish-brown, composed of conidiophores and setae, mycelium semi-immersed or immersed. **Anamorph.** Setae present or occasionally absent, grow singly or in small groups from repent hyphae or knots of hyphal cells, erect, straight or flexuous, septate, brown, unbranched, always fertile with a terminal or several lateral phialidic openings, setae rarely absent. Conidiophores macronematous, mononematous, crowded, arise singly or in groups from repent hyphae or knots of hyphal cells, scattered among the setae if present, unbranched, occasionally branched, erect, straight or flexuous, sometimes geniculate, brown, septate, smooth. Conidiogenous cells integrated, terminal on conidiophores or short phialide-bearing branches, or discrete, lateral on conidiophores or 1–2-celled stalks, mono- and polyphialidic, extending percurrently and sympodially, pigmented, often with persistent remnants of the collarettes; collarettes flared, funnel-shaped, the apical part may become soon evanescent. Conidia falcate, cylindrical-fusiform, curved, slightly asymmetrical, tapering toward both ends, slightly truncate at the base with an inconspicuous scar, aseptate, hyaline, with straight or gently curved setula at each end inserted terminally at the apex and subterminally at the base, accumulate in slimy fascicles. **Teleomorph.** Unknown.

Habitat and geographical distribution: Members of *Codinaeella* are saprobes, found on decaying bark, wood, woody fruits, leaves, petioles or palm fronds of various plants, but also in soil and living roots, and have a worldwide distribution in Holarctic realm, but also occur in the subtropical and tropical geographic zones of Africa, Asia, Australasia, and South America.

Notes: Two morphotypes were found among *Codinaeella* species (Figure 8). Species with the predominant morphotype CA1 (e.g., *Ca. minuta*, *Ca. lutea*, *Ca. parvilobata*) form unbranched conidiophores that grow singly or in small groups from repent hyphae or knots of hyphal cells and are usually scattered among longer, darker and thicker-walled unbranched setae. The setae resemble conidiophores; they are always fertile and terminate into a mono- or polyphialide. Although conidiophores are simple on material from nature, in *Ca. minuta* and *Ca. lambertiae* conidiophores often branch in culture. The other morphotype CA2 is less widespread and it is represented by *Ca. filamentosa* only. It is characterized by single, branched conidiophores with a sterile setiform extension and lower fertile part, and they are accompanied by shorter, unbranched conidiophores. Conidiogenous cells are monophialidic, discrete, lateral and also integrated, terminal on stalks or short phialide-bearing branches and shorter conidiophores. The teleomorph of *Codinaeella* is unknown.

Eight species were accepted in *Codinaeella* and all were verified with DNA sequences. Several other species closely resemble *Codinaeella* and although their systematic placement is unknown and molecular data are unavailable, their inclusion in *Codinaeella* should be considered based on future phylogenetic arguments. Species such as *Dictyochaeta gamundiae* [163], *D. pakhalensis* [164], and *D. taiwanensis* [165] match the CA1 morphotype. *Codinaea sinensis* [166], *Dictyochaetopsis* (*Di*.) *elegantissima*, *Di. intermedia* [32] and *Di. menisporoides* [26] correspond to the CA2 morphotype with branched conidiophores and discrete phialides.

*Codinaeella* is separated from *Codinaea* by molecular and morphological characters. The two genera differ in the arrangement of setae (if present) and conidiophores on the natural substrate and partly also in the production of pigments in vitro. The morphology of *Codinaea* is more complex and consists of four morphotypes, but none of them is comparable with those of *Codinaeella*. *Codinaeella* often forms bright yellow, ochre, orange to dark burgundy pigments dispersed in agar, whereas in *Codinaea* the pigments are rarely formed. A synopsis table with diagnostic features of accepted species of *Codinaeella* is provided in the Supplementary File: Table S5.

**Figure 21:**
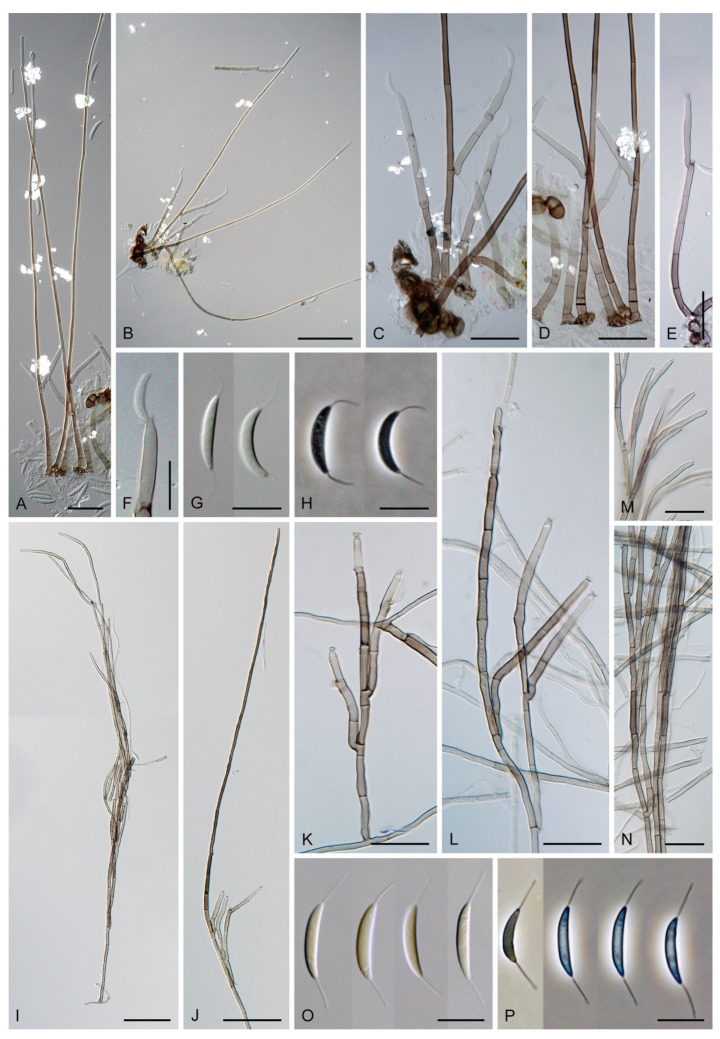
*Codinaeella filamentosa* (CBS 147265). (**A**–**E**,**I**–**N**) Conidiophores (**F**) conidiogenous cell (**G**,**H**,**O**,**P**) conidia. Images: (**A**–**H**) from nature (**I**–**P**) on MLA after 4–8 weeks. Scale bars: (**A**) 20 μm; (**B**,**I**,**J**) 50 μm; (**C**–**E**,**K**–**N**) 20 μm; (**F**–**H**,**O**,**P**) 10 μm.

**Figure 22:**
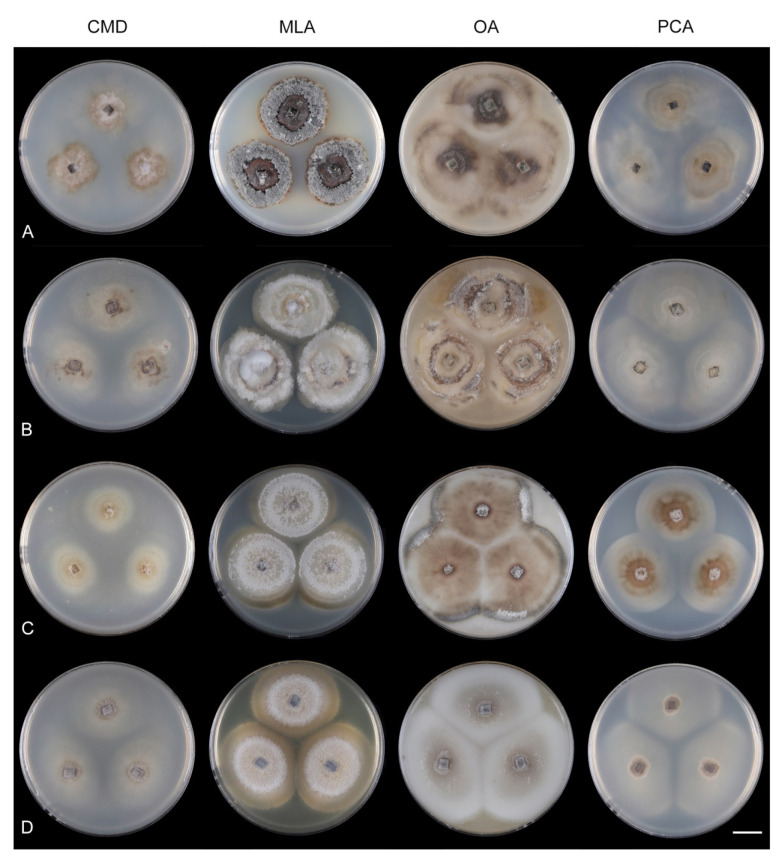
Colony morphology of *Codinaeella* spp. after 4 weeks. (**A**) *Ca. filamentosa* CBS 147265 (**B**) *Ca. lambertiae* CBS 143419 ex-type (**C**) *Ca. mimusopis* CBS 143435 ex-type (**D**) *Ca. pini* CBS 138866 ex-type. Scale bar: (**A**–**D**) 1 cm.

***Codinaeella filamentosa*** (Onofri) Réblová & Hern.-Restr., **comb. nov.** MycoBank MB 842202. (Figure 21 and Figure 22).

Basionym. *Codinaea filamentosa* Onofri, Mycotaxon 14: 120. 1982.

≡ *Dictyochaetopsis filamentosa* (Onofri) Aramb. & Cabello, Mycotaxon 38: 12. 1990.

Description on the natural substrate: Colonies effuse, grey-brown, composed of conidiophores, mycelium immersed. **Anamorph.** Setae comparable to the upper layer of branched setiform conidiophores. Conidiophores macronematous, grow singly or in groups of 2–3, erect, straight or flexuous, septate, brown, smooth, form two distinct layers. Conidiophores of the upper layer 252–275 μm long, 3.5–4 μm wide near the base, branched, the upper part of the conidiophore is sterile with a setiform extension, apex obtuse and subhyaline, lower part of the conidiophore is fertile, lateral branches and grow just below the septa, they are either sterile and similar to the main stalk, tapering, up to 166 μm long, or shorter phialide-bearing branches 29.5–51 × 3.5–4 μm. Conidiophores of the lower layer 28–85 μm long, 3–4 μm wide, pale brown, paler toward the apex, unbranched with a terminal phialidic opening, occasionally with discrete phialides. Conidiogenous cells 13–35 × 2.5–3.5 μm tapering to 1.5–2 μm below the collarette, integrated, terminal or discrete, lateral, monophialidic, rarely polyphialidic with one lateral opening, elongating percurrently and sympodially, cylindrical, pale brown, subhyaline toward the apex; collarettes ca. 3 μm wide, 1.5–2 μm deep, funnel-shaped, hyaline. Conidia (13.5–)14.5–17.5(–18) × 2–2.5 (mean ± SD = 16 ± 1.6 × 2.5 ± 0.2 μm), falcate, tapering toward both ends, aseptate, hyaline, with a straight or gently curved setula at each end, 5–8 μm long, inserted terminally at the apex, subterminally at the base, conidia accumulate in slimy whitish fascicles.

Culture characteristics: On CMD: colonies 35–38 mm diam, circular, flat, margin entire, velvety, later mucoid and smooth, whitish-brown, beige toward the margin, reverse beige with a dark brown zone. On MLA: colonies 25–27 mm diam, circular, flat, raised margin, margin entire to weakly undulate, mucoid-waxy to cobwebby, lanose at the margin, colony centre dark burgundy-brown, whitish-grey to grey at the margin with a dark ochre outer zone, dark burgundy (centre) and pale ochre pigment (margin) diffusing into agar, reverse dark burgundy to black. On OA: colonies 50–53 mm diam, circular, flat, slightly raised, margin entire, mucoid, velvety to cobwebby centrally and at the margin, zonate, irregularly dark brown at the centre with beige zones of sparse growth, reverse brown. On PCA: colonies 40–42 mm diam, circular, flat, margin entire, cobwebby, slightly funiculose becoming mucoid, zonate, beige-brown, isabelline at the margin, reverse of the same colors. Sporulation was sparse on MLA, OA, PCA, absent on CMD.

Colonies on MLA effuse, mycelium composed of branched, septate, pale brown hyphae 2–2.5 μm diam. Conidiophores 160–493 μm long, 2.5–3.5 μm wide near the base, tapering upwards, sometimes with a sterile, dark brown, setiform extension up to 377 μm long, apex narrowly rounded, erect, straight or flexuous, septate, smooth, medium brown, simple or branched; phialide-bearing branches arise in the lower part of the conidiophore, secondary and tertiary branches formed frequently, parallel, extending upwards percurrently, slightly curved downwards at the apical part. Conidiogenous cells 18–42 × 3–3.5 μm tapering to 1.5–2 μm below the collarette, monophialidic, pale brown to subhyaline; collarettes 3–4 μm wide, 1.5–2.5 μm deep, subhyaline, funnel-shaped, apical part often evanescent. Conidia similar to those from nature, 13.5–17 × 2–3 μm (mean ± SD = 15 ± 0.9 × 2.4 ± 0.3 μm), falcate, setulae 5.5–8.5 μm long, accumulate in slimy whitish fascicles.

Specimen examined: USA, Louisiana, Baton Rouge, Burden Plantation, on buried leaves of *Quercus* sp., 11 February 2002, K.A. Seifert K.A.S. 1504 (culture CBS 147265).

Habitat and geographical distribution: A saprobe on leaf litter of *Anthonotha fragrans*, *Calpocalyx brevibracteatus*, *Chrysophyllum taiense*, *Quercus* sp. and other unknown hosts, known from Africa and North America: Ivory Coast and the USA, Louisiana ([32,35], this study).

According to GlobalFungi, this species was found in six soil samples from forest habitat in Eastern North America in New York, Massachusetts [119,167]. The sites have temperate or humid continental climate (MAT avg. 10 °C, MAP avg. 1238 mm). In addition, *Ca. filamentosa* is identical with *Ca. minuta* in the ITS1 barcode. Thus, only the ITS2 region was used to study its biogeography.

Notes: On the natural substrate, *Ca. filamentosa* forms two kinds of conidiophores, of which the longer are branched and have a sterile setiform extension; the branches are also sterile and similar to the main stalk. These longer conidiophores can be considered equivalent to the setae (although, apically always fertile) observed in other species of *Codinaeella*, such as *Ca. lutea*, *Ca. mimusopis*, *Ca. minuta* and *Ca. parvilobata*. *Codinaeella filamentosa* has a distinctive characteristic of colony on MLA. The flat, central part of the colony is dark burgundy-brown, mucoid becoming cobwebby in older cultures. Aerial mycelium is usually confined to the periphery where it is abundant, lanose and forms a raised, grey margin. During the analysis of the secondary metabolites, we identified naphthoquinones bioactive compounds produced from the central pigmented part of the colony (A. Čmoková et al., in preparation).

Four other species, such as *C. sinensis*, *Dictyochaetopsis elegantissima*, *Di. Intermedia,* and *Di. menisporoides*, resemble *Ca. filamentosa* in having discrete, lateral phialides, falcate, aseptate, hyaline conidia with setulae and sometimes branched conidiophores. Sometimes longer conidiophores are accompanied by shorter simple conidiophores or stalked phialides. *Dictyochaetopsis elegantissima* differs from *Ca. filamentosa* in conidia with bifid setulae at each end [32]. *Dictyochaetopsis intermedia* differs from *Ca. filamentosa* in having conidiophores with a terminal phialidic opening and wider conidia. *Codinaea sinensis* has unbranched conidiophores and shorter conidia [166] compared to *Ca. filamentosa*. *Dictyochaetopsis menisporoides* differs from *Ca. filamentosa* in polyphialidic conidiogenous cells, unbranched conidiophores with mostly sessile phialides, sometimes formed on branches [26]. The systematic placement of these species is unknown; the living cultures or molecular data are not available.

**Figure 23:**
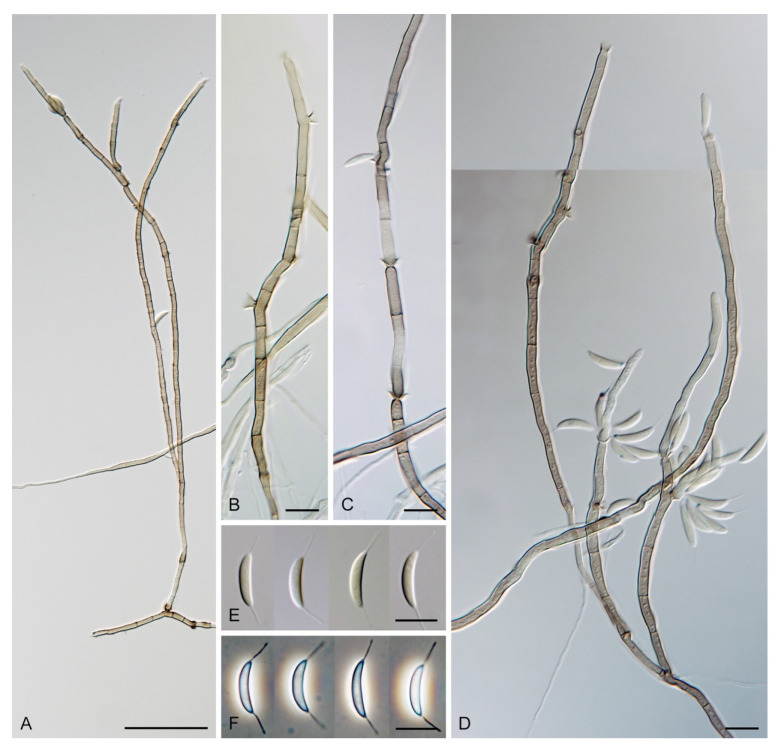
*Codinaeella lamberiae* (CBS 143419 ex-type). (**A**–**D**) Conidiophores (**E**,**F**) conidia. Images: (**A**–**D**) on OA after 4 weeks. Scale bars: (**A**) 50 μm; (**B**–**F**) 10 μm.

***Codinaeella* *lambertiae*** (Crous) Réblová & Hern.-Restr., **comb. nov.** MycoBank MB 842203. (Figure 22 and Figure 23).

Basionym. *Codinaea lambertiae* Crous, Persoonia 39: 399. 2017.

Culture characteristics: On CMD: colonies 62–64 mm diam, circular, flat, margin entire, mucoid to cobwebby, brown centrally, beige with irregular brown spots, reverse of the same color. On MLA: colonies 57–58 mm diam, circular, convex, margin entire, sparsely lanose, mucoid at the margin, zonate, furrowed, white-ochre with irregular dark brown to orange-brown intermediate zones, with an isabelline outer zone of submerge growth, reverse amber with dark brown rings. On OA: colonies 60–62 mm diam, circular, raised, margin entire, velvety to cobwebby, floccose, locally mucoid with a sparse growth, pink-brown, whitish-brown toward the margin sometimes with orange-brown outer zone, light ochre-pink pigment diffusing into the agar, reverse ochre-yellow to yellow-orange. On PCA: colonies 59–60 mm diam, circular, flat, margin entire, cobwebby to mucoid, zonate, beige, locally olivaceous beige at the margin, reverse beige. Sporulation was moderate on MLA, OA, and PCA, absent on CMD.

Colonies on OA effuse, mycelium composed of branched, septate, hyaline to pale brown hyphae 1.5–2.5 μm diam. **Anamorph.** Setae absent. Conidiophores 128–362 μm long, 2.5–3 μm wide near the base, usually tapering toward the base, macronematous, single, simple or branched, erect, septate, straight or flexuous, geniculate toward the apex due to numerous lateral phialidic openings, smooth, pale brown. Conidiogenous cells 19–34 × 3–3.5 μm tapering to 1.5–2(–2.5) μm below the collarette, integrated, terminal, mono- and polyphialidic with 1–6 lateral openings while internally septa can be formed, extending percurrently and sympodially, cylindrical, pale brown; collarettes 4–5.5 × 2–3(–4) μm, funnel-shaped, pale brown to subhyaline. Conidia 11.5–14.5 × 2.5–3 μm, falcate, tapering toward both ends, slightly truncate at the basal end with an inconspicuous scar, aseptate, hyaline, with a straight or gently curved setula at each end, 4–6.5 μm long, inserted terminally at the apex, subterminally at the base, accumulate in slimy whitish fascicles. **Teleomorph.** Unknown.

Specimen examined: AUSTRALIA, New South Wales, Fitzroy Falls, Morton National Park, on leaves of *Lambertia formosa*, 26 November 2016, P.W. Crous (culture ex-type CBS 143419).

Habitat and geographical distribution: Saprobe on fallen leaves of *Lambertia formosa*, known from Australia only [10]. According to GlobalFungi, this species was found mostly in Southeast Australia and Tasmania in 14 samples, mostly in soil in forest or woodland environments [117,168,169]. A single soil sample originates from Asia (East China) from a cropland biome. The sites have temperate or humid continental climate (MAT avg. °C, MAP avg. 1091 mm).

Notes: When grown on OA, the conidiophores of *Ca. lambertiae* (CBS 143419) branch; the branches are fertile and curved upwards. Similar branching pattern was observed in *Ca. filamentosa* and *Ca. minuta* (this study). Phialides extend percurrently and later also sympodially and the upper part of the conidiophore acquires a slightly geniculate appearance. In the protologue of *Ca. lambertiae* [10], the conidiophores were described simple, conidiogenous cells monophialidic, rarely with a lateral phialidic aperture and the conidia were given longer ((13–)14–15(–18) × (2.5)–3 μm. Because of these differences we present a description of *Ca. lambertiae* on OA.

*Codinaeella lambertiae* is similar to *Ca. parvilobata* but differs in having slightly longer and wider conidia with shorter setulae. While *Ca. lambertiae* mainly occurs in Australasia, *Ca. parvilobata* has a wide range of distribution.

**Figure 24:**
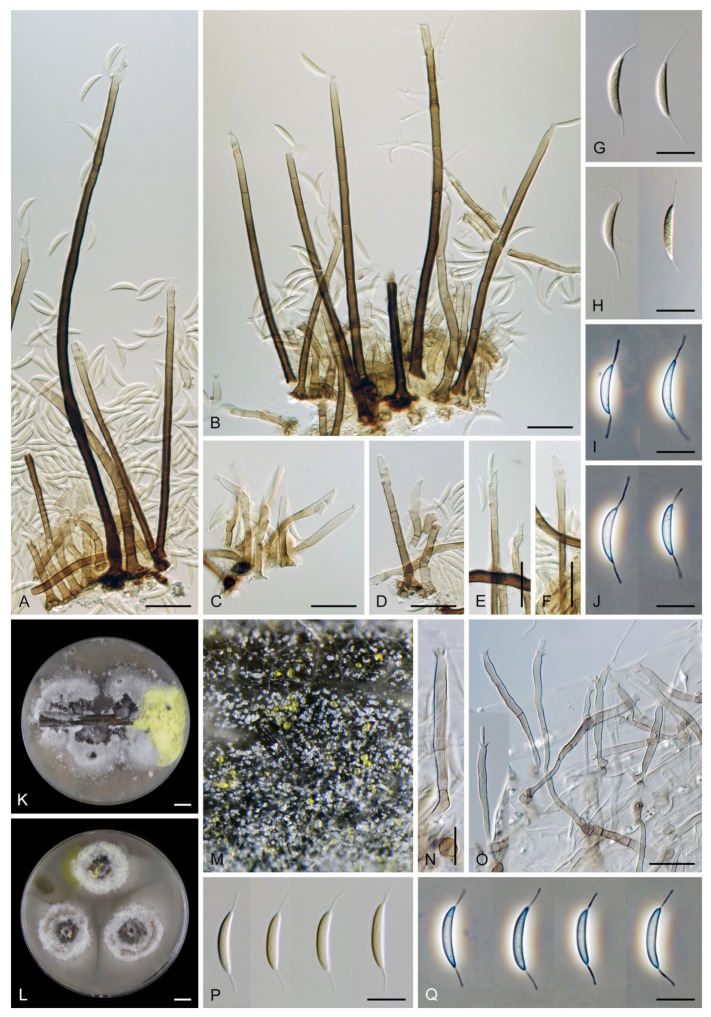
*Codinaeella lutea*. (**A**–**F**,**N**,**O**) Conidiophores (**G**–**J**,**P**,**Q**) conidia (**K**–**M**) colonies. Images: (**A**–**J**) from nature (**K**,**M**–**Q**) on stems of *U. dioica* on CMA after 8 weeks (**L**) on OA after 4 weeks (**A**–**L**) from CBS 146618 ex-type (**M**–**Q**) from CBS 624.77. Scale bars: (**A**–**F**,**O**) 20 μm; (**K**,**L**) 1 cm; (**N**,**P**,**Q**) 10 μm.

**Figure 25:**
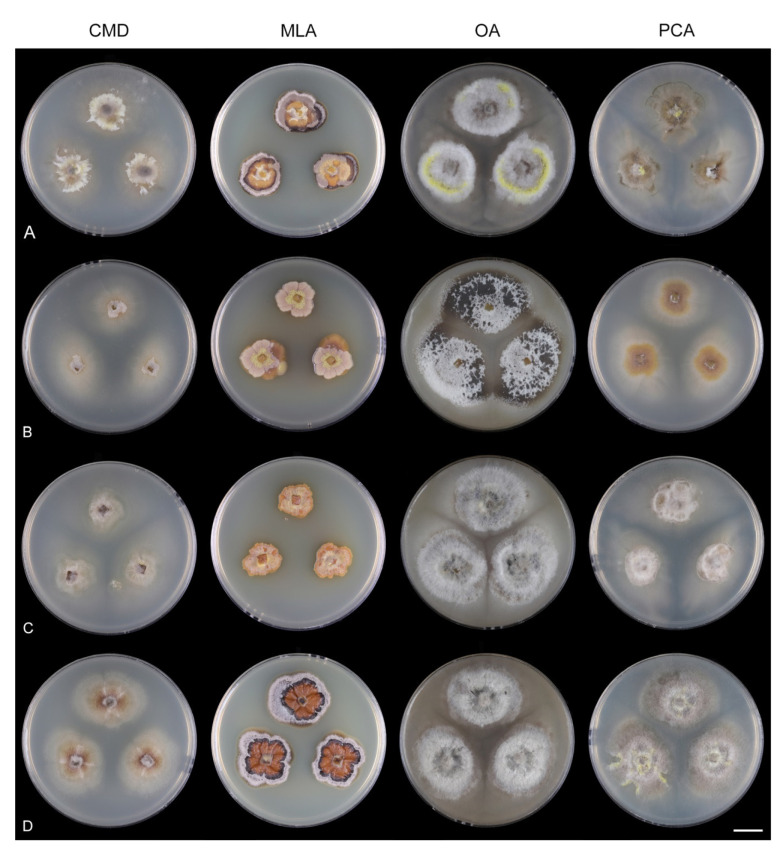
Colony morphology of *Codinaeella lutea* after 4 weeks. (**A**) CBS 146618 ex-type (**B**) CBS 624.77 (**C**) ICMP 14613 (**D**) ICMP 15544. Scale bar: (**A**–**D**) 1 cm.

***Codinaeella* *lutea*** Réblová & Hern.-Restr**., sp. nov.** MycoBank MB 842204. (Figure 24 and Figure 25).

Typification: CZECH REPUBLIC, South Bohemian region, Novohradské hory Mts., Horní Stropnice, Bedřichovský potok Natural Monument, on decaying cupule of *Quercus* sp., 5 October 2018, M. Réblová M.R. 3982 (**holotype** PRA-20986, culture ex-type CBS 146618).

Etymology: *Luteus* (L) yellow, referring to the yellow pigment diffusing into agar, and staining conidial masses and aerial mycelium in vitro.

Description on the natural substrate: Colonies effuse, grey-brown, composed of setae and conidiophores, mycelium semi-immersed. **Anamorph.** Setae 125–250 μm long, (3.5–)4.5–5.5 μm wide near the swollen base, grow singly or in groups, erect, straight or flexuous, septate, smooth, dark brown and thick-walled, paler and thinner-walled toward the apex, unbranched, always fertile, apex pale brown to subhyaline with a single terminal or 1–5 lateral phialidic openings. Conidiophores 39–102 μm long, 3.5–4.5 μm wide near the base, macronematous, crowded, arise singly or in groups of 2–3, scattered among the setae, unbranched, erect, straight or flexuous, slightly geniculate in the apical part, septate, smooth, pale to medium brown, paler toward the apex. Conidiogenous cells 19.5–29(–32) × 3.5–4.5 μm tapering to 1.5–2 μm below the collarette, integrated, terminal, mono- or polyphialidic with 2–8 lateral openings while internally septa can be formed, extending sympodially, cylindrical, slightly swollen below the collarette, pale brown, subhyaline at the apex, usually bearing persistent remnants of the collarettes; collarettes 3–3.5 μm wide, 2–2.5 μm deep, funnel-shaped, subhyaline to pale brown, the apical part soon evanescent. Conidia 12.5–17.5 × 2–2.5(–3) μm (mean ± SD = 15.6 ± 1.4 × 2.6 ± 0.2 μm), falcate, asymmetrical, tapering toward both ends, slightly truncate at the base with an inconspicuous basal scar, aseptate, hyaline, with straight or gently curved setula at each end (4.5–)5–8.5(–10) μm long, inserted terminally at the apex, subterminally at the base, conidia accumulate in slimy whitish fascicles. **Teleomorph.** Unknown.

Culture characteristics: On CMD: colonies 35–40 mm diam, circular, flat, margin entire to fimbriate, sparsely lanose, somewhat funiculose, mucoid toward the periphery, whitish-beige centrally, pale cinnamon when mucoid, beige to isabelline toward the margin, pale yellow-gold pigment diffusing into agar, reverse cinnamon. On MLA: colonies 15–18 mm diam, circular, raised, margin entire to lobate, velvety-lanose becoming mucoid-waxy, glossy, furrowed, pink-beige, apricot to cinnamon with remnants of yellow to white-yellow aerial mycelium at the inoculation block, sometimes with a dark grey to dark burgundy outer zone when mucoid and beige-brown lanose margin, diffusing apricot pigment into the agar, more intense color at the margin, reverse ochre, apricot to cinnamon. On OA: colonies 60–65 mm, circular, raised, margin entire, velvety-lanose, floccose, occasionally partially mucoid, furrowed, sometimes ridges with small cracks, white to beige-brown centrally, dark brown in the mucoid part, with a yellow ring or irregular yellow patches of aerial hyphae with numerous yellow exudates, sometimes yellow to pale ochre pigment diffusing locally into agar, reverse orange-brown. On PCA: colonies 69–73 mm diam, circular, raised, margin entire to fimbriate, lanose, floccose, white-beige, sometimes mucoid and cinnamon centrally, with irregular yellow tufts of aerial mycelium, beige toward the margin, pale ochre pigment diffusing into agar, reverse olivaceous grey-brown to cinnamon. Sporulation was sparse on PCA, absent on CMD, MLA, and OA.

Colonies on CMA with *U. dioica* stems effuse, mycelium composed of branched, septate, hyaline to pale brown hyphae 1–2 μm diam. **Anamorph.** Setae absent, conidiophores, conidiogenous cells, and conidia similar to those from nature. Conidiophores 36–83 μm long, 2–4 μm wide above the base, usually tapering toward the swollen base, unbranched, erect, septate, smooth, pale brown to subhyaline, sometimes reduced to single conidiogenous cells. Conidiogenous cells 16–32 × 3.5–4.5 μm tapering to 1.5–2.5 μm below the collarette, integrated, terminal, mono-, occasionally polyphialidic with 1–2 lateral openings, subcylindrical, extending percurrently and sympodially, pale brown to subhyaline; collarettes 3.5–5 μm wide, 1.5–2.5 μm deep, funnel-shaped. Conidia 14.5–17 × 2–3 μm (mean ± SD = 15.8 ± 0.8 × 2.6 ± 0.3 μm), falcate, hyaline, aseptate, setulae 4–7.5 μm long, accumulate in slimy whitish or yellow fascicles. **Teleomorph.** Unknown.

Other specimens examined: NEW ZEALAND, Canterbury region, Christchurch, Christchurch Botanical Gardens, on a dead leaf of *Quercus ilex*, 2 September 2002, J.A. Cooper JAC8343 (PDD 80130, culture ICMP 14613). NEW ZEALAND, Tasman region, Murchison, Dough Boy Track, on dead cupule of *Quercus* sp., 15 May 2004, J.A. Cooper JAC9057 (PDD 80612, culture ICMP 15540). THE NETHERLANDS, Utrecht, Fort Rijnauwen, ingang (1b), on cupule of *Quercus robur*, 22 October 1977, W. Gams (CBS H-746, culture CBS 624.77).

Habitat and geographical distribution: *Codinaeella lutea* is a saprobe on decaying leaves and woody fruits of *Quercus ilex*, *Q. robur*, *Quercus* sp. And other unknown host. It is known from Australasia, Europe, and Southeast Asia: China, Czech Republic, New Zealand and The Netherlands.

According to GlobalFungi, the identical sequences were found in 53 samples of roots (72%), soils (26%), or water (2%) originating mostly from forest (81%) and grassland (13%) habitats in Europe (49 samples, Belgium Denmark, Wales) [170,171,172], Asia (3 samples, Iran) [115]. It was also found in the single water sample from North America (North Carolina) [173]. The sites have the temperate climate (MAT avg. 11 °C, MAP avg. 85 mm).

Notes: The four strains of *Ca. lutea* examined in this study produce yellow pigment, which diffuses into the agar and also stains aerial mycelium in vitro. This character is conspicuous in the ex-type strain on OA (Figure 24K,L). In other isolates the yellow-colored hyphae formed sparsely, either on MLA or PCA (Figure 25). In addition, yellow conidial masses formed when grown on *Urtica* stems on CMA (Figure 24M). The four isolates of *Ca. lutea* from Asia, Europe and New Zealand have identical ITS sequences, although their *tef1-α* sequences show certain variability corresponding to 99.35–99.78% sequence identity; the *tef1-α* sequence of the Asian isolate is unavailable.

*Codinaeella lutea* resembles *Ca. minuta*, but differs in longer setae, narrower conidia, bright yellow color of the conidial masses in vitro and especially colony characteristics in vitro including the color of the pigment released into the agar. The color ranges from yellow to pale ochre, yellow-gold to apricot in *Ca. lutea*, but it is usually ochre, deep orange to bright carrot orange in *Ca. minuta*. On MLA, the growth of *Ca. lutea* is considerably slower (15–18 mm) than that of *Ca. minuta* (42–45 mm). *Codinaeella parvilobata* also bears a close resemblance to *Ca. lutea*, but differs in shorter setae, shorter conidia and colony characteristics. For a detailed comparison of these two species, see notes to *Ca. parvilobata*. *Dictyochaeta pakhalensis* [164] resembles *Ca. lutea*, but differs in shorter and narrower conidia. In the present phylogeny, *Ca. lutea*, *Ca. minuta* and *Ca. parvilobata* form distinct lineages. Molecular data and culture of *D. pakhalensis* are unavailable.

**Figure 26:**
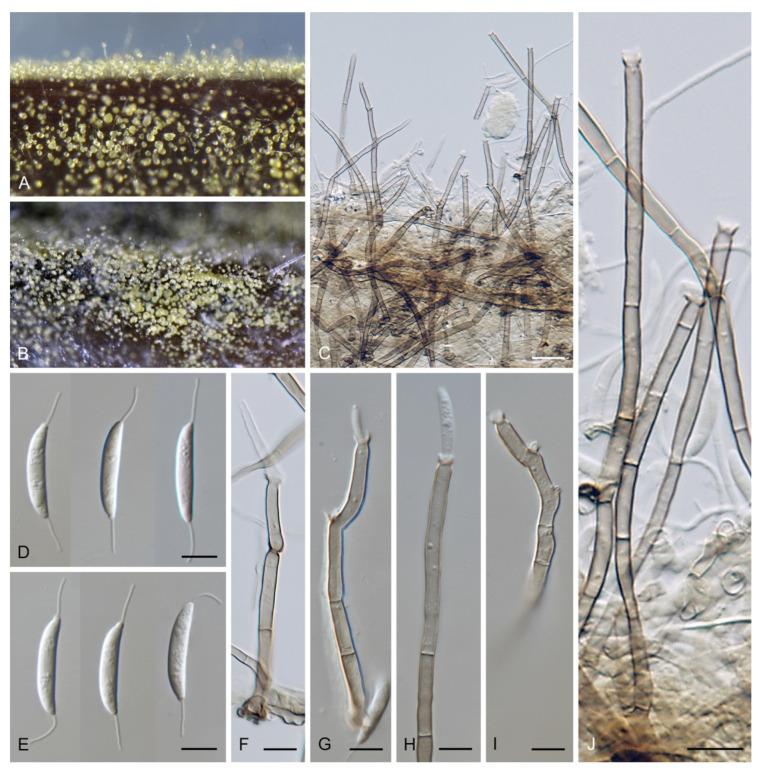
*Codinaeella mimusopis* (CBS 143435 ex-type). (**A**,**B**) Colonies (**C**,**F**–**J**) conidiophores (**D**,**E**) conidia. Images: (**A**) on a pine needle on SNA after 8 weeks (**B**) on a stem of *U. dioica* on CMA after 8 weeks. Scale bars: (**C**,**F**–**I**) 20 μm; (**D**,**E**) 5 μm; (**J**) 10 μm.

***Codinaeella mimusopis*** (Crous & M.J. Wingf.) Réblová & Hern.-Restr., **comb. nov.** MycoBank MB 842205. (Figure 22 and Figure 26).

Basionym. *Dictyochaeta mimusopis* Crous & M.J. Wingf., Fungal Syst. Evol. 1: 185. 2018.

For description and additional illustrations, see Crous et al. [82].

Culture characteristics: On CMD: colonies 25–28 mm diam, circular, flat, margin entire, lanose on the inoculation block, mucoid, zonate, whitish becoming pale olivaceous brown, isabelline toward the periphery, reverse of similar colors. On MLA: colonies 38–39 mm diam, circular, flat, raised margin, margin entire, velvety-lanose, partly mucoid, whitish, pale olivaceous brown at the margin, reverse zonate, pale brown, cinnamon at the margin. On OA: colonies 61–63 mm diam, circular, flat, margin entire, lanose becoming mucoid, aerial mycelium restricted to the centre and margin of the colony, brown, olivaceous grey to white-grey centrally and at the margin, reverse olivaceous brown. PCA: colonies 57–58 mm diam, circular, flat, margin entire, lanose at the inoculation block, mucoid and smooth toward the periphery, zonate, whitish centrally, cinnamon, beige toward the margin, reverse brown. Sporulation was moderate on MLA and OA, absent on CMD and PCA.

Specimen examined: SOUTH AFRICA, Eastern Cape Province, Haga Haga, on leaves of *Mimusopis caffra*, December 2010, M.J. Wingfield (culture ex-type CBS 143435).

Habitat and geographical distribution: On leaves of *Mimusopis caffra* known so far from Africa, South Africa [82]. The GlobalFungi database does not contain similar (≥97%) sequences of this species.

Notes: Our observations based on the ex-type strain match the protologue in all details, except for the conidiophores. Two kinds of conidiophores were observed on sterile stems of *Urtica* on CMA. Longer, setiform conidiophores up to 245 μm long were accompanied by shorter conidiophores 38–130 μm vs. conidiophores 40–150 μm [82]. However, the longer conidiophores were not different from the shorter ones. They have almost the same color, width near the base and wall thickness. They could be considered equivalent to the setae that form regularly in other species of *Codinaeella* in nature. The phialides are terminal, elongating percurrently and sympodially to form 1–3 lateral openings. Pigments diffusing from the colony into agar were not produced on any of the used media. Conidia formed on *Urtica* stems on CMA accumulated in yellow slimy fascicles (Figure 26A,B), while those formed on pine needles on SNA and other media were white (not shown). *Codinaeella mimusopis* resembles *Ca. lambertiae* [10], but differs in shorter conidiophores and longer conidia [82].

**Figure 27:**
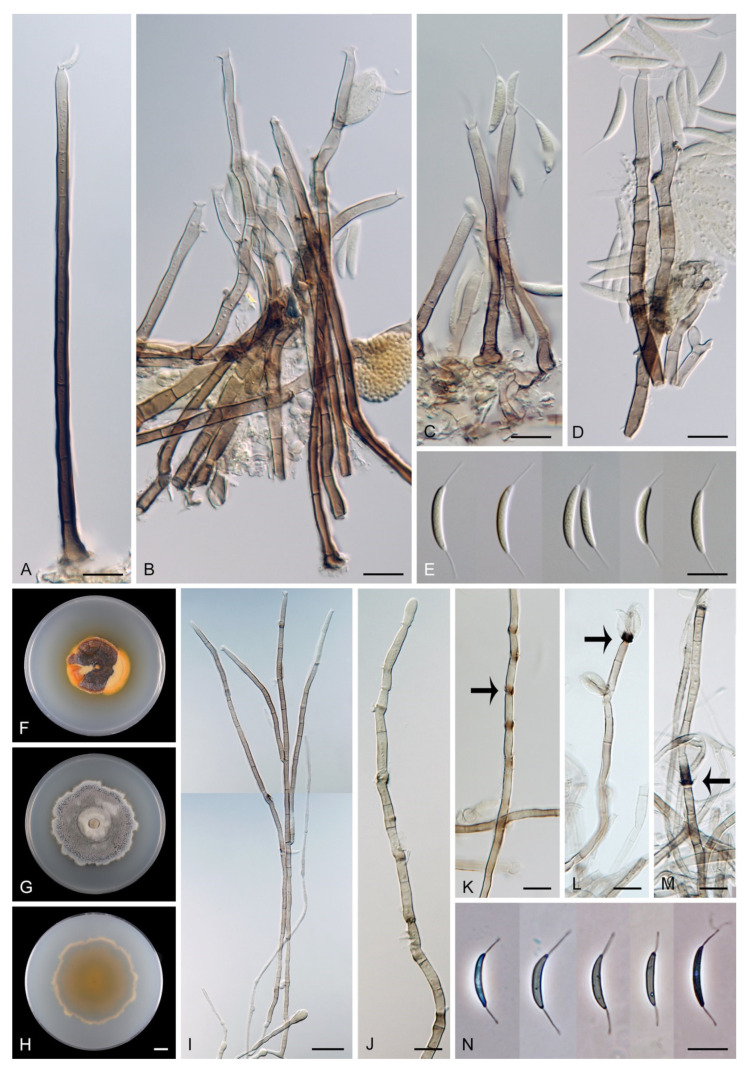
*Codinaeella minuta*. (**A**–**D**,**I**–**M**) Conidiophores (**E**,**N**) conidia (**F**–**H**) colonies. Images: (**A**–**E**) from nature (**F**–**H**) on MLA after 4 weeks (**I**–**N**) on a stem of *U. dioica* on CMA after 4–8 week (**A**–**E**) from CBS 145099 (**F**) from CBS 146619 (**G**,**H**,**L**,**M**) from DAOM 148141 (**I**–**K**,**N**) from CBS 146619. Scale bars: (**A**–**E**,**J**–**N**) 10 μm; (**F**–**H**) 1 cm; (**I**) 20 μm.

**Figure 28:**
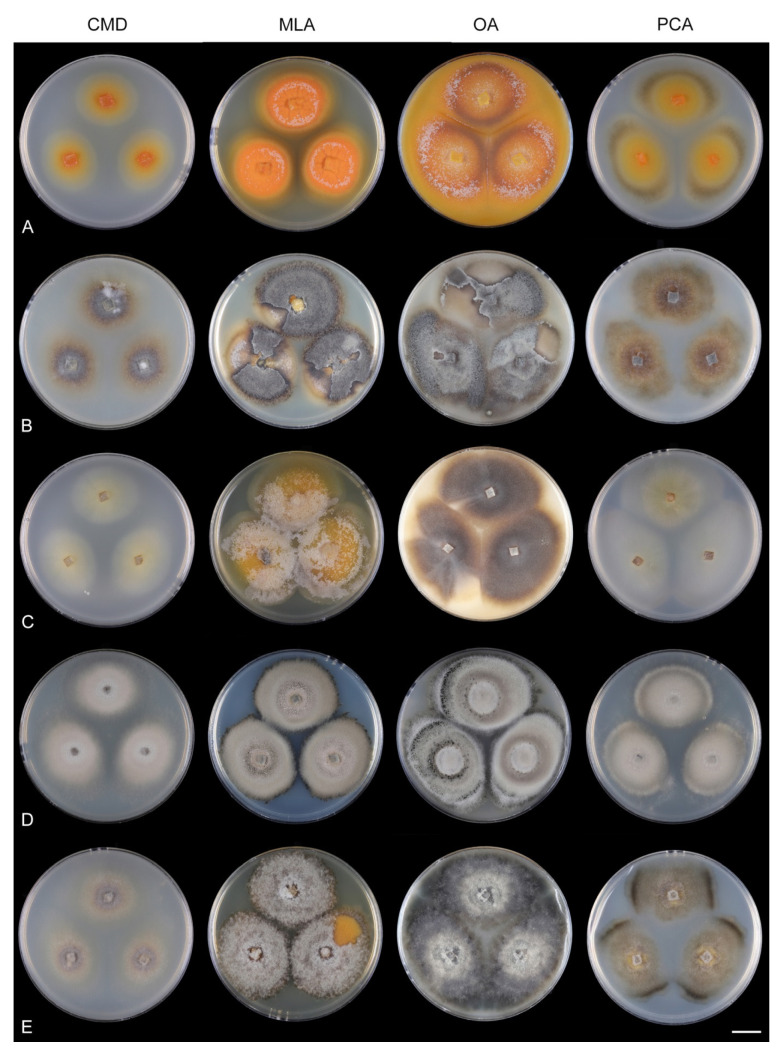
Colony morphology of *Codinaeella minuta* after 4 weeks. (**A**) CBS 966.69 (**B**) CBS 146621 (**C**) CBS 115959 (**D**) DAOM 148141 (**E**) ATCC 20960. Scale bar: (**A**–**E**) 1 cm.

***Codinaeella* *minuta*** (Tubaki) Réblová & Hern.-Restr., **comb. nov.** MycoBank MB 842207 (Figure 27 and Figure 28).

Basionym. *Menispora minuta* Tubaki, J. Hattori bot. Lab. 20: 166. 1958.

Description on the natural substrate: Colonies effuse, grey-brown to olivaceous grey, composed of setae and conidiophores, mycelium semi-immersed or immersed. **Anamorph.** Setae 123–184 μm long, 3–4.5 μm wide near the base, crowded, grow singly or in groups of two, erect, straight or flexuous, septate, smooth, dark brown, paler and thinner-walled toward the apex, unbranched, apex pale brown to subhyaline with a terminal or 1–3 lateral phialidic openings. Conidiophores 32–108 μm long, 3–4 μm wide near the base, macronematous, crowded, arise singly or in groups of 2–3, scattered among the setae, sometimes aggregate in loose columns, unbranched, erect, straight or flexuous, septate, smooth, pale brown, subhyaline toward the apex, sometimes reduced to single conidiogenous cells. Conidiogenous cells 20.5–35 × 2.5–3.5 μm tapering to 1.5–2 μm below the collarette, integrated, terminal, mono- and polyphialidic with 1–5 lateral openings while internally septa can be formed, extending percurrently and sympodially, cylindrical, pale brown, subhyaline toward the apex, usually bearing persistent remnants of the collarettes; collarettes 3–4 μm wide, ca. 2 μm deep, funnel-shaped, subhyaline, the apical part soon evanescent. Conidia 13–18 × 2.5–3.5 μm (mean ± SD = 16.2 ± 1.1 × 2.8 ± 0.3 μm), falcate, tapering toward both ends, slightly truncate at the base with an inconspicuous scar, aseptate, hyaline, with a straight or gently curved setula at each end, 5.5–8.5(–10) μm long, inserted terminally at the apex, subterminally at the base, conidia accumulate in slimy whitish fascicles. **Teleomorph.** Unknown.

Culture characteristics: On CMD: colonies 61–65 mm diam, circular, flat, margin entire, lanose, floccose, cobwebby toward the margin, becoming mucoid, varying in color due to the absence of aerial mycelium, beige brown to pale banana yellow, occasionally orange with a yellow-gold margin, pale ochre to orange pigment diffusing into agar, reverse beige, ochre-beige to orange. On MLA: colonies 42–45 mm diam, circular, flat, margin entire to fimbriate, lanose, floccose becoming mucoid, grey-brown to dark brown when aerial mycelium is present, deep carrot orange to dull orange brown with orange-gold margin when mucoid, pale orange pigment diffusing into agar, reverse orange to dark amber. On OA: colonies 58–63 mm diam, circular, raised, margin entire, sparsely lanose becoming mucoid, beige-grey to dark grey toward the margin when aerial mycelium is present, orange with tawny margin when mucoid, sometimes orange to ochre pigment diffusing into agar, reverse dark grey-brown or amber. On PCA: colonies 70–73 mm diam, circular, flat, margin entire to fimbriate, sparsely lanose becoming mucoid, grey-brown, tawny or orange with olivaceous brown margin on mucoid spots, pale orange pigment diffusing into agar, reverse dark olivaceous brown to amber. Sporulation was abundant on all media.

Description on CMA with *U. dioica* stems. Colonies effuse, mycelium composed of branched, septate, hyaline to subhyaline hyphae 1.5–3 μm diam. **Anamorph.** Setae absent, conidiophores, conidiogenous cells, and conidia similar to those from nature. Conidiophores 46–297(–450) μm long, 3–3.5 μm wide near the base, branched or unbranched, erect, septate, smooth, pale to medium brown. Conidiogenous cells 13.5–32.5 × 3.5–4.5 μm tapering to 1.5–2 μm below the collarette, integrated, terminal, extending percurrently with 1–10 proliferations, and sympodially with 1–4 lateral openings, cylindrical, pale brown to subhyaline, with persistent remnants of the collarettes; collarettes 3.5–5 μm wide, 1.5–2.5 μm deep, funnel-shaped, dark brown becoming hyaline upon aging. Conidia 11.5–17.5(–18) × 2–3 μm (mean ± SD = 14.9 ± 1.1 × 2.5 ± 0.4 μm), falcate, setulae 4.5–7 μm long, accumulate in slimy whitish fascicles. **Teleomorph.** Unknown.

Specimens examined: CZECH REPUBLIC, Central Bohemian region, Úvaly, Škvorecká obora-Králičina Nature park, on decaying acorn of *Quercus* sp., 21 April 2018, M. Réblová M.R. 3944 (PRA-20991, culture CBS 146619); *Ibid*., M.R. 3946 (PRA-20992, culture CBS 146620); *Ibid*., M.R. 3948 (PRA-20993, culture CBS 146621); *Ibid*., South Bohemian region, Novohradské hory Mts., Horní Stropnice, Bedřichovský potok Natural Monument, on decaying acorn of *Quercus* sp., 5 October 2018, M. Réblová M.R. 3983 (PRA-20990). FRANCE, Ariège, Pyrenées Mts., Rimont, La Maille brook, alt. 550 m, on decaying acorn of *Quercus* sp., 28 May 2018 (incubated for 2 weeks in a damp chamber), J. Fournier M.R. 3951 (PRA-20994, culture CBS 145099); *Ibid*., M.R. 3952 (PRA-20995, culture CBS 145100). ITALY, on branch of *Quercus suber*, F. Marras (culture CBS 115959). JAPAN, Chiba Prefecture, Kiyozumi, on dead leaves of *Lithocarpus edulis*, Aug. 1954, K. Tubaki (**authentic strain** of *Menispora minuta* MUCL 9903 = MUCL 15759 = CBS 280.59). THE NETHERLANDS, Gelderland province, Rheden, National Park Veluwezoom, on decaying acorn of *Quercus* sp., 6 September 1969, V. Holubová-Jechová (CBS 966.69); *Ibid*., Utrecht province, Utrecht, Utrecht Science park near Westerdijk Fungal Biodiversity Institute, on a fallen leaf of *Quercus* sp., August 2018, M. Hernández-Restrepo M.H.R. 18063 (culture CBS 147518). USA, Louisiana, New Orleans, Audobon Park, on leaf litter of *Quercus virginiana*, 19 April 1974, R. Bandoni & K.A. Pirozynski 6120 B/K.A.S. 7210 (culture DAOM 148141). USA, Texas, Houston, E. & A. Taylor Park, on a dead leaf of *Quercus virginiana*, date unknown, G. Bills (culture TTI-0830). USA, West Virginia, Fayette Co., Fayette Station, hardwood leaf litter, date unknown, collector unknown (culture ATCC 20960 = MF5247 = Mycosearch 1673).

Habitat and geographical distribution: *Codinaeella minuta* is a saprobe on fallen leaves and woody fruits of *Fagus sylvatica*, *Lithocarpus edulis*, *Quercus petraea*, *Q. robur*, *Q. virginiana*, *Quercus* sp., and other unknown hosts. It occasionally occurs on bark and wood of *Q. suber*. It is known from Asia, Europe, and North America: the Czech Republic, France, Japan, Italy, The Netherlands, Slovak Republic and USA ([26,174], this study).

According to GlobalFungi, the identical sequences were found in 20 samples of soil (80%), litter (15%) and root (5%) from forest (95%) or grassland (5%) habitats in Europe (60% of all samples, Denmark, France, Hungary, Poland, Sweden), Asia (15%, Iran), and in western part of North America (25%, Florida, Michigan). The samples originate from eight studies and the locations have temperate or humid continental climate (MAT avg. 13 °C, MAP avg. 864 mm). *Codinaeella minuta* is identical with *Ca. filamentosa* in ITS1 barcode. Thus, only ITS2 region was used to study *C. minuta* biogeography.

Notes: This species was described as *Menispora minuta* from dead leaves of *Lithocarpus edulis*, an evergreen tree native to Japan [174]. Although Tubaki [174] characterized this species in culture only, we update its description based on observations from nature based on the newly acquired material. Among members of *Codinaeella*, *Ca. minuta* is well distinguished by the production of deep orange pigment on nutrient media, especially on MLA and OA (Figure 28, Supplementary Files: Figures S3 and S4). *Codinaeella minuta* is similar to *Ca. lutea*, but it differs in shorter setae, slightly wider conidia and colony characteristics in vitro. For a detailed comparison, see notes to *Ca. lutea*.

Interestingly, majority of the strains of *Ca. minuta* included in this study were originally identified as *Codinaea simplex* [2]. It is possible that the concept of the latter species has been misinterpreted in the literature. Our suspicions were confirmed by the revision of the isotype of *C. simplex* (DAOM 96020g), which revealed that *Ca. minuta* and *C. simplex* are two different species. *Codinaea simplex* forms a single layer of conidiophores up to 115 μm long (usually 35–68 µm); conidiophores are several-septate but often they are reduced to conidiogenous cells with only 1–2 supporting cells. On the other hand, the conidiophores of *Ca. minuta* vary in height and form two layers, setiform conidiophores 123–184 μm long referred to as setae and shorter ones up to 108 μm long. At maturity, setae and conidiophores of *Ca. minuta* may cluster in loose columns, which are partly shown on Figure 27B; they adhere to each other and are difficult to separate. Holubová-Jechová [26] reported a collection of *C. simplex* from the Czech Republic with longer conidiophores 30–155(–230) μm adhering in loose columns similar to synnemata. It is likely that this collection represents in fact *Ca. minuta*, which is fairly common in the Czech Republic. Conidiophores of different heights were also observed in culture, in strains growing on stems of *U. dioica* on CMA. Solitary, dichotomously branched conidiophores occur frequently in vitro when growing on agar medium. Our observations agree with those of Tubaki [174]. Using morphological and geographical data from GlobalFungi, the systematic placement of *C. simplex* was revaluated and the species was transferred to *Tainosphaeria* in this study.

We observed undescribed variability in colony characteristics in strains of *Ca. minuta* originating from different geographic regions (Figures S3 and S4). The presence of orange pigment diffusing into the agar is widespread among strains, and it is most pronounced on MLA and OA. The color of the pigment differs in intensity and ranges from bright carrot orange or deep orange to yellow-orange, pale orange-brown to dark ochre. We observed that colony color is correlated with the development of aerial mycelium. Both isolates from The Netherlands, CBS 966.69 and CBS 147518, abundantly produced deep orange pigment on all media. In these isolates, aerial mycelium was scarce, restricted to the periphery or central zone of the colonies, soon became mucoid and the surface of the colonies turned deep orange (Figure S3). In other strains the aerial mycelium was more or less abundant, lanose, locally mucoid, and only then the mucoid, bright orange-colored part of the colony with the diffusing pigment was visible. The development of orange pigment and lack of aerial mycelium differed even within a single strain and were found to be correlated with the volume of the nutrient medium and number of colonies on one plate; the pigment production was pronounced when the colonies grew singly on 10 cm Petri dish (Figure 27F) vs. in a triple on the 6 cm Petri dish (Figure 28). Although pigment was sparse or absent on all media in the North American strain DAOM 148141 from the frontal view (Figure 28D), it formed just below the colony, causing the orange coloration of the reverse side (Figure 27G,H).

In the phylogeny based on ITS-28S-*tef1-α* sequences, *Ca. minuta* is represented by 12 strains from Asia, Europe, and North America. While 11 of these strains were grouped with the ex-type strain CBS 298.61 in a monophyletic, well-supported clade, the strain ATCC 20960 clustered at the base of *Ca. minuta* clade and the relationship was not statistically supported. All 12 strains of *Ca. minuta* have identical ITS1 and ITS2 sequences, except for the isolate ATCC 20960, which differs in one base pair in the ITS1. More significant sequence divergence was observed in the *tef1-α* gene. However, the strain ATCC 20960 is well comparable with other strains of *Ca. minuta* in morphology of conidiophores, conidiogenous cells and conidia in culture, colony characteristics and production of orange pigment. In material collected in different countries the size of conidia of *Ca. minuta* in vitro was comparable: 13–15.5(–16) × 2–3 μm (Czech Republic), 13–16 × 2–3 μm (France), 10–16 × 2–3 μm (Japan, Tubaki 1958), 11.5–14 × 2–3 μm (USA, DAOM 148141) and 13–16.5(–17) × 2–3 μm (USA, TTI-0830). However, their conidia from nature are slightly larger, 13–18 × 2.5–3.5 μm (Czech Republic, France). In culture, conidia of the strain ATCC 20960 were slightly longer at their upper limit, 13.5–17.5(–18) × 2–3 μm (mean ± SD = 15.4 ± 1.1 × 2.4 ± 0.3 μm) with setulae 4.5–7.5 μm long. Wild type of ATCC 20960 is unknown; the herbarium specimen from which the strain was isolated is not available for study.

Despite the variability of ATCC 20960 in the *tef1-α* gene, its morphology and geographic distribution based on ITS analysis of environmental sequences compares well with *Ca. minuta*. Therefore, we prefer to keep this strain as a representative of this species. The strain ATCC 20960 is of a particular interest, because it has been studied in detail in terms of bioactive compounds and their potential in disease treatment. It produces β-1,3-d-glucan synthesis inhibitor with antifungal and anti-*Pneumocystis* activity [175,176].

**Figure 29:**
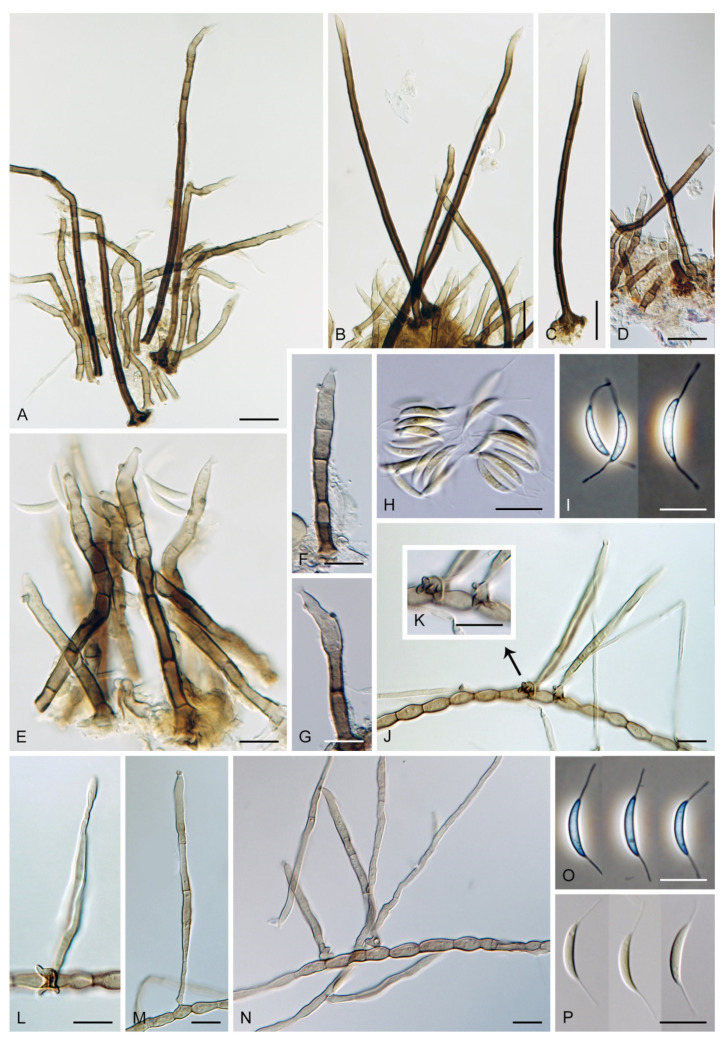
*Codinaeella parvilobata* (CBS 144536 ex-type). (**A**–**G**) Conidiophores (**H**,**I**) conidia (**J**–**N**) conidiophores (**O**,**P**) conidia. Images: (**A**–**I**) from natural substrate (**J**–**P**) on MLA after 4 weeks. Scale bars: (**A**–**D**) 20 μm; (**E**–**P**) 10 μm.

**Figure 30:**
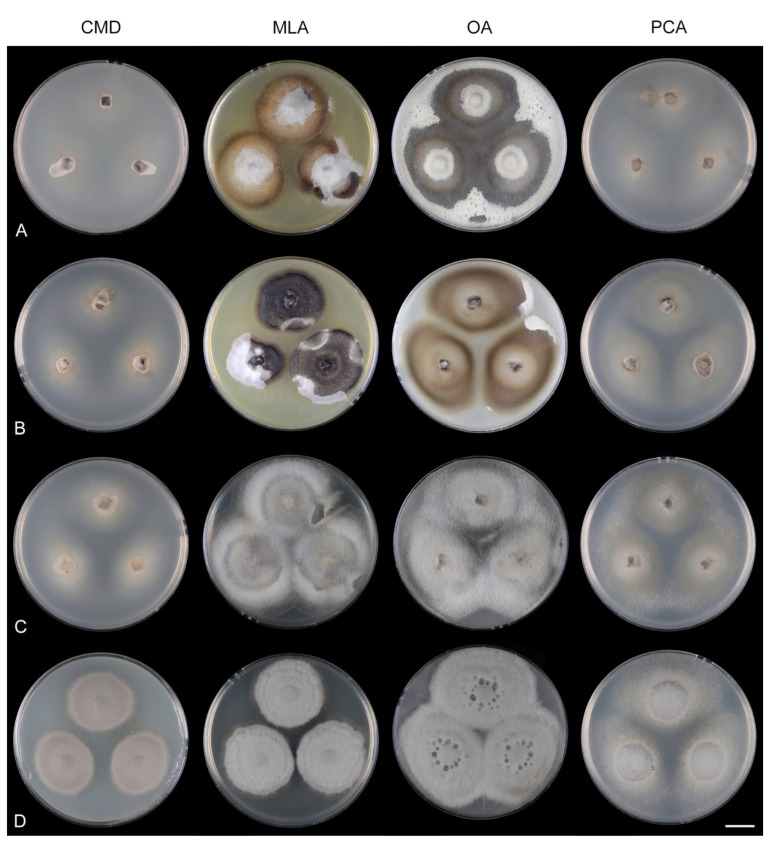
Colony morphology of *Codinaeella parvilobata* after 4 weeks. (**A**) CBS 144536 ex-type (**B**) CBS 144792 (**C**) CBS 144658 (**D**) MUCL 28054. Scale bar: (**A**–**D**) 1 cm.

***Codinaeella parvilobata*** Réblová & Hern.-Restr., **sp. nov.** MycoBank MB 842208. (Figure 29 and Figure 30).

Etymology: *Parvus* (L) small, *lobus* (L) lobe, referring to small lobes at the base of conidiophores in vitro.

Typification: CZECH REPUBLIC, South Bohemian region, Novohradské hory Mts., Dobrá voda, Hojná voda National nature monument, on decaying cupule of *Fagus sylvatica*, 29 September 2017, M. Réblová M.R. 3900 (PRA-20987 **holotype**, culture ex-type CBS 144536).

Description on the natural substrate: Colonies effuse, brown-grey, composed of setae and conidiophores, mycelium semi-immersed. **Anamorph.** Setae 140–205 μm long, 4.5–5.5 μm wide near the swollen base, crowded, arise singly or in groups, erect, straight, flexuous or bent, septate, smooth, dark brown and thick-walled, paler and thinner-walled toward the apex, simple, apex pale brown with a terminal or 1–6 lateral phialidic openings, sometimes apex remains sterile, obtuse and hyaline. Conidiophores 40–117 μm long, 3–4.5(–5) μm wide near the base, macronematous, crowded, arise singly or in groups, scattered among the setae, unbranched, erect, straight, usually flexuous or bent, geniculate to undulate toward the apex, septate, smooth, mid to pale brown, paler toward the tip. Conidiogenous cells 9–34.5 μm × 3.5–4.5 μm tapering to 1–2 μm below the collarette, integrated, terminal, monophialidic, or polyphialidic with 1–7 lateral openings while internally septa can be formed, extending percurrently and sympodially, cylindrical, pale brown, subhyaline at the apex, usually with persistent remnants of the collarettes; collarette 2–2.5 μm wide, 1.5(–2) μm deep, funnel-shaped, hyaline, the apical part soon evanescent. Conidia 10.5–13.5 μm × 2–2.5(–3) μm (mean ± SD = 12.4 ± 0.8 μm × 2.0 ± 0.2 μm), falcate, tapering toward both ends, slightly truncate at the base with an inconspicuous scar, aseptate, hyaline, with a straight or gently curved setula at each end, 6.5–9 μm long, inserted terminally at the apex, subterminally at the base, conidia accumulate in slimy whitish fascicles. **Teleomorph.** Unknown.

Culture characteristics: On CMD: colonies 40–43 mm diam, circular, flat, convex centrally, margin fimbriate, velvety-lanose, mucoid toward the periphery, whitish, beige to beige-grey, ochre-beige to brown when mucoid, isabelline toward the margin, reverse isabelline. On MLA: colonies 32–36 mm diam, circular, flat, convex centrally, margin fimbriate, lanose becoming mucoid, wrinkled, whitish grey, buff yellow, cinnamon to olivaceous brown when mucoid, pale yellow to pale ochre pigment diffusing into agar, sometimes the pigment is missing, reverse dark ivory to ochre, dark brown margin. On OA: colonies 78–80 mm diam, circular, flat, convex centrally, margin entire, velvety-lanose becoming mucoid, zonate, smoke grey, cinnamon to dark olivaceous brown when mucoid, aerial hyphae with colorless exudates, reverse amber to dark brown. On PCA: colonies 46–50 mm diam, circular, flat, convex centrally, margin entire to fimbriate, velvety-lanose, somewhat funiculose at the centre, mucoid to cobwebby toward the periphery, pale olivaceous beige, reverse beige to dark grey. Sporulation was moderate on MLA and PCA, absent on CMD and OA.

Colonies on MLA effuse, mycelium composed of branched, septate, subhyaline to pale brown hyphae 1.5–3.5 μm diam, or monilioid hyphae 4–5.5 μm diam. **Anamorph.** Setae absent, conidiophores, conidiogenous cells and conidia similar to those from nature. Conidiophores 38–84 μm long, 1.5–3 μm wide near the base, sometimes tapering toward the base, arise singly from vegetative or monilioid hyphae, usually attached by small lobes at the base, unbranched, erect, septate, smooth, pale brown. Conidiogenous cells 20–31 × 2.5–3.5 μm tapering to 1.5–2 μm, monophialidic, pale brown; collarette 2–2.5 μm wide, 1.5–2 μm deep, funnel-shaped, pale brown. Conidia 10.5–14 × 2–2.5 μm (mean ± SD = 12.6 ± 0.8 × 2.3 ± 0.2 μm), falcate, hyaline, aseptate, setulae 4–8 μm long, accumulate in slimy whitish fascicles. **Teleomorph.** Unknown.

Other specimens examined: CZECH REPUBLIC, South Bohemian region, Novohradské hory Mts., Dobrá voda, Hojná voda National nature monument, on decaying seed of *Fagus sylvatica*, 4 October 2012, M. Réblová M.R. 3757 (PRA-20988, culture CBS 144658 = MUCL 56490). CZECH REPUBLIC, Central Bohemian region, Úvaly, Škvorecká obora-Králičina Nature park, on decaying acorn of *Quercus* sp., 21 April 2018, M. Réblová M.R. 3947 (PRA-20989, culture CBS 144792). BELGIUM, Wallonie, Brabant wallon, Louvain-la-Neuve, Bois de Lauzelle, on decaying leaf in forest stream, date unknown, date unknown, G.L. Hennebert (culture MUCL 28054).

Habitat and geographical distribution: A saprobe on decaying seeds and wooden fruits often buried in soil and on leaves of *Fagus sylvatica*, *Quercus* sp. and an unknown host in terrestrial and freshwater biotopes, known from Europe from Belgium and the Czech Republic.

According to GlobalFungi, the identical sequence was found in 227 samples of soil (65%), litter (13%), root (11%), and deadwood (9%) collected mostly in forest (90%), grassland (4%) or anthropogenic (4%) habitats. Most of the samples were from Europe (177 samples, Czech Republic, Belgium, Denmark, France, Germany, Wales), 25 samples were from North America (Massachusetts, New Jersey) and 25 samples were from Asia (Eastern China, Japan and South Korea). The samples originated from 25 studies. The sites have temperate climate (MAT avg. 8.4 °C, MAP avg. 851 mm).

Notes: In culture, some conidiophores of *Ca. parvilobata* are attached to the pigmented, monilioid hyphae with a lobate, swollen base, a character that was not observed in other species of the genus. The pale yellow to pale ochre pigment diffusing in the agar on MLA was produced only in two strains, including the ex-type strain. The pigment production and visibility is correlated with the absence of aerial mycelium; the colonies become mucoid and their surface changes from whitish-grey to buff yellow, cinnamon to olivaceous brown.

It is challenging to distinguish *Ca. parvilobata* and *Ca. lutea* based on morphological characters, because the size of their conidiophores, conidiogenous cells, and also conidia partially overlap under natural conditions. *Codinaeella lutea* differs in having longer setae and longer conidia in their upper range. However, both species differ especially in colony characteristics and pigment production on MLA and OA. *Codinaeella parvilobata* can also be compared with *Ca. minuta*, but the latter species differs in longer conidia and production of ochre to deep orange pigment.

**Figure 31:**
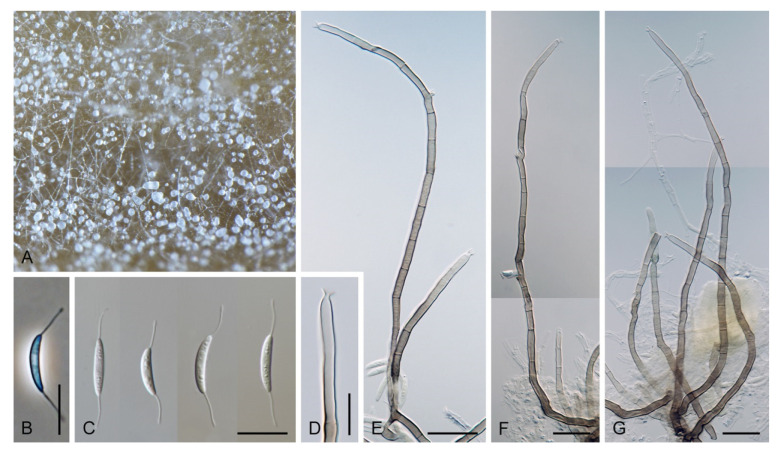
*Codinaeella pini* (CBS 138866 ex-type). (**A**) Colony (**B**,**C**) conidia (**D**) conidiogenous cells (**E**–**G**) conidiophores. Images: (**A**–**I**) on a stem of *U. dioica* on CMA after 8 weeks. Scale bars: (**B**–**D**) 10 μm; (**E**–**G**) 20 μm.

***Codinaeella pini*** (Crous & M.J. Wingf.) Réblová & Hern.-Restr., **comb. nov.** MycoBank MB 842210 (Figure 22 and Figure 31).

Basionym. *Codinaea pini* Crous & M.J. Wingf., Persoonia 33: 251. 2014.

For description and additional illustrations, see Crous et al. [8].

Culture characteristics: On CMD: colonies 60–61 mm diam, circular, raised, margin entire, sparsely lanose, floccose becoming mucoid, beige to olivaceous grey centrally, isabelline-beige toward the periphery, reverse brown. On MLA: colonies 76–77 mm diam, circular, raised, margin entire, lanose, floccose, mucoid at the margin, finely furrowed, whitish, ochre-beige at the margin, pale yellow-ochre pigment diffusing into the agar, reverse brown. On OA: colonies 76–78 mm diam, circular, flat, margin entire, cobwebby to powdery centrally becoming mucoid, beige, whitish toward the margin, reverse pale brown. On PCA: colonies 78–79 mm diam, circular, flat, margin entire, sparsely lanose becoming mucoid, pale brown centrally, isabelline toward the periphery, reverse whitish. Sporulation was abundant on PCA, moderate on CMD, MLA, and OA.

Specimen examined: UGANDA, on dead needles of *Pinus patula*, January 2014, M.J. Wingfield (culture ex-type CBS 138866).

Habitat and geographical distribution: Saprobe on dead needles of *Pinus patula*, known so far from Africa, Uganda [8]. According to GlobalFungi, the identical sequences were found in 58 soil and five shoot samples (*Pinus taeda* needles) in the forests of North America (North Carolina, Louisiana, Ontario, British Columbia) originating from studies of Wilhelm et al. [177] and Oono et al. [178]. The sites have temperate, hemiboreal, or humid continental climate (MAT avg. 17 °C, MAP avg. 1144 mm).

Notes: Our observations of the ex-type strain match the protologue. However, conidiophores on MLA formed two distinct layers compared to a single layer of conidiophores 30–100 μm long reported by Crous et al. [8]. On MLA, the upper layer consisted of unbranched, flexuous or bent, setiform conidiophores 130–232 × 2.5–3.5 μm terminating into a mono- or polyphialide with 1–2 lateral apertures, while the lower layer comprised of shorter conidiophores 56–98 × 2.5–3.5 μm with a terminal phialidic aperture. The strain sporulated on all nutrient media included in this study and also on stems of *Urtica* on CMA and pine needles on SNA. Conidia accumulated in slimy whitish fascicles.

***Codinaeella yunnanensis*** (Z.L. Luo, K.D. Hyde & H.Y. Su) Réblová & Hern.-Restr., **comb. nov.** MycoBank MB 842211.

Basionym. *Codinaea yunnanensis* Z.L. Luo, K.D. Hyde & H.Y. Su, Fungal Divers. 99: 590. 2019.

For description and illustrations, see Luo et al. [29].

Habitat and geographical distribution: Saprobe on submerged decaying wood, known only from Asia, China [29]. According to GlobalFungi, the identical sequences were found in three soil samples from forests in Asia (Central China Hubei province and Iran) [115,179]. The localities have temperate climate (MAT avg. 16 °C, MAP avg. 1094 mm).

Notes: *Codinaeella yunnanensis* resembles *Ca. lutea* and *Ca. mimusopis* in conidial characters, however, the size of their conidia significantly overlaps. For morphological comparison, see Table S5. In the phylogenetic analysis, all three species formed separate lineages; *Ca. yunnanensis* nested in a clade with *Ca. lutea* and *Ca. pini*.

#### 3.5.3. The Genus Nimesporella

***Nimesporella*** Réblová & Hern.-Restr., **gen. nov.** MycoBank MB 842002.

Type species. *Nimesporella capillacea* Réblová & Hern.-Restr.

Etymology: An anagram of *Menisporella*, a synonym of *Codinaea*.

Description: Colonies effuse, brown, composed of conidiophores, mycelium immersed. **Anamorph.** Setae absent. Conidiophores macronematous, mononematous, single or in groups, erect, straight or flexuous to geniculate in the upper part, unbranched, septate, smooth, brown, paler toward the apex. Conidiogenous cells integrated, terminal, polyphialidic, extending sympodially, paler than the conidiophore, often with persistent remnants of the collarettes; collarettes flared, soon evanescent. Conidia ellipsoidal, rounded at the apical end, papillate at the basal end, aseptate, hyaline, with a straight or gently curved setula at each end, basal setulae positioned ventrally, conidia accumulate in slimy whitish fascicles. **Teleomorph.** Unknown.

Habitat and geographical distribution: *Nimesporella* occurs on decaying plant material in freshwater and terrestrial habitats, and it is known from Africa.

Notes: *Nimesporella capillacea* is the only species of the genus available to represent this uncommon morphotype in the present phylogeny. It forms a basal lineage to the *Codinaea* clade but their relationship is not statistically well supported. *Nimesporella* differs from *Codinaea* mainly by the characters of conidiogenous cells with multiple phialidic apertures, early evanescent collarettes and papillate, broadly ellipsoidal conidia with a simple setula at each end, inserted terminally at the apex and somewhat ventrally at the base. When grown in culture, the phialides extend sympodially for a short distance, producing densely aggregated phialidic openings encircled by tubular, apically flared collarettes. Similar polyphialides were also recorded in *Multiguttulispora* [49].

Species such as *Dictyochaeta aliformis*, *D. daphnioides* and *D. tumidospora* known only from Southeast Asia from Malaysia [22] bear a striking resemblance to *N. capillacea* in all characteristics. On material from nature [22], conidiogenous cells frequently elongate sympodially, while internally septa may be formed. As a result, 1–2 intercalary phialidic openings are formed per each cell in the upper part of the conidiophore. After careful comparison of their original descriptions and illustrations with *N. capillacea* we suggest that they are likely congeneric. Their transfer to *Nimesporella* should be supported by DNA data, which are unavailable for study at the moment. The strain of *N. capillacea* was originally deposited under the name *D. daphnioides*.

**Figure 32:**
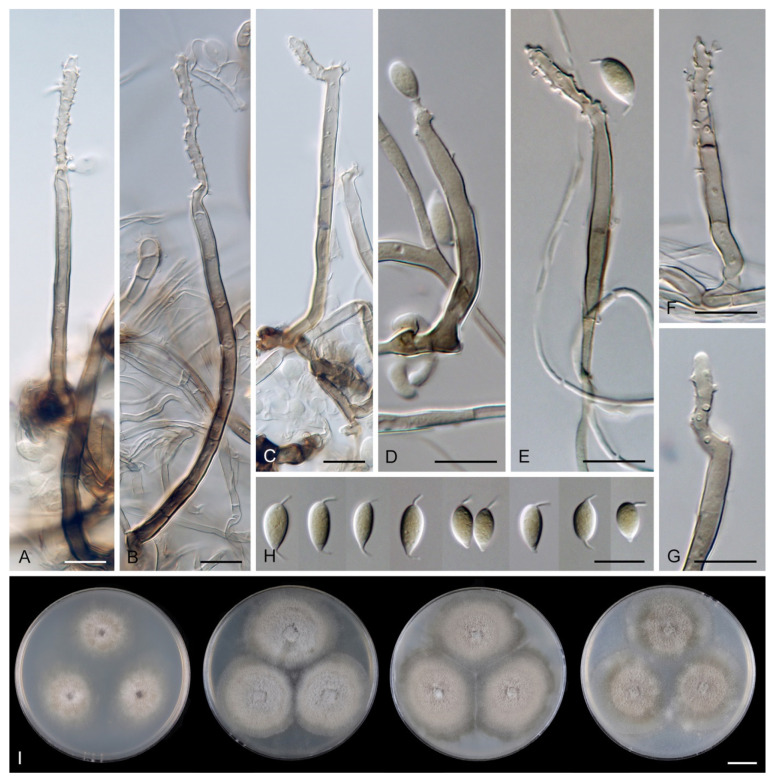
*Nimesporella capillacea* (IMI 358908 ex-type). (**A**–**G**) Conidiophores (**H**) conidia (**I**) colonies on CMD, MLA, OA and PCA after 4 week (from left to right). Images: (**A**–**H**) on PCA after 4–6 weeks. Scale bars: (**A**–**H**) 10 μm; (**I**) 1 cm.

***Nimesporella* *capillacea*** Réblová & Hern.-Restr., **sp. nov.** MycoBank MB 842212. (Figure 32).

Etymology: *Capillus* (L) hair or thread, -*acea* (L) indicating resemblance, meaning hair-like or thread-like, referring to conidial setulae.

Typification: IVORY COAST, Tai National Park, leaf litter, 11 March 1992, S. Pagano (**holotype** IMI 358908 microscopic slides, ex-type culture IMI 358908).

Culture characteristics: On CMD: colonies 41–42 mm diam, circular, flat, margin fimbriate, sparsely lanose becoming cobwebby, white, beige toward the periphery, reverse grey-beige, isabelline at the margin. On MLA: colonies 38–39 mm diam, circular, convex, margin entire, lanose, floccose, furrowed, cobwebby at the margin, whitish centrally, beige-grey to ochre-beige with an olivaceous beige outer zone, reverse dark amber. On OA: colonies 65–68 mm diam, circular, flat, margin entire to weakly fimbriate, velvety-lanose, floccose to funiculose centrally, mucoid at the margin, beige with an olivaceous outer zone, reverse olivaceous beige. On PCA: colonies 51–52 mm diam, flat, circular, flat, similar to colonies on OA, sparsely lanose, floccose to funiculose centrally, whitish-beige becoming olivaceous beige, with an isabelline outer zone of submerged growth, reverse zonate, olivaceous beige. Sporulation was abundant on MLA, OA, PCA, sparse on CMD.

Colonies on MLA effuse, mycelium composed of branched, septate, hyaline hyphae 1.5–2.5 μm diam. **Anamorph.** Setae absent. Conidiophores 28–130 μm long, 3–4.5 μm wide near the base, macronematous, occasionally reduced to conidiogenous cells, grow solitarily from vegetative hyphae or arise in groups of 2–3 from dark knots of hyphal cells, erect, straight or slightly bent, septate, smooth, brown, paler toward the tip. Conidiogenous cells 22–35(–45) × 3–3.5 μm, slightly tapering upwards, 2–3.5 μm wide below the fertile apical part, integrated, terminal, extending sympodially over a short distance, polyphialidic with 5–22 lateral openings, fertile part 5.5–17(–23.5) × 2–3.5 μm, occasionally dichotomously branched, with persistent remnants of the collarettes; collarettes 1.5–2 μm wide, 1.5–2 μm deep, hyaline, minute, tubular, apically flared, evanescent, the apical part sometimes attached to the bottom of the conidium. Conidia 8–11.5 μm × 4.5–5 μm (mean ± SD = 9.5 ± 0.9 × 4.5 ± 0.2 μm), ellipsoidal, broadly rounded at the apical end, papillate or beak-like at the basal end, aseptate, hyaline, with a straight or gently curved setula at each end, 2–3.5 μm long, positioned terminally at the apex and subterminally at the base, conidia accumulate in slimy whitish fascicles. **Teleomorph.** Unknown.

Habitat and geographical distribution: Saprobe on leaf litter in freshwater habitat, known in Ivory Coast.

Notes: *Nimesporella capillacea* closely resembles *N. aliformis*, *N. daphnioides* and *N. tumidospora*, all known only from Malaysia [22], but which differ in having longer conidiophores and larger conidia.

#### 3.5.4. The Genus Stilbochaeta

***Stilbochaeta*** Réblová & Hern.-Restr., **gen. nov.** MycoBank MB 842001.

Etymology: *Stilbos* (Greek) glistening, referring to slimy droplets of conidia that are glistening when fresh; *chaeta* (Greek) hair, bristle.

Type species. *Stilbochaeta malaysiana* (Kuthub.) Réblová & Hern.-Restr.

Description on the natural substrate: Colonies effuse, hairy, brown, composed of setae and conidiophores, occasionally ascomata, mycelium immersed or semi-immersed. **Anamorph.** Setae grow singly or in groups from stromatic cells or knots of hyphal cells, erect, straight or flexuous, septate, brown, paler toward the apex, unbranched, apex sterile, bluntly rounded, sometimes with a terminal or several lateral phialidic openings. Conidiophores macronematous, mononematous, single or grow in fascicles from stromatic cells or knots of hyphal cells around the base of the setae, erect, straight or flexuous to slightly undulating, unbranched, septate, smooth, brown, paler toward the apex. Conidiogenous cells integrated, terminal, mono- or polyphialidic, extending percurrently and sympodially, paler than the conidiophores; collarettes flared, funnel-shaped, often slightly stipitate or tubular near the base. Conidia falcate, oblong-falcate, ellipsoidal-fusiform, curved, tapering toward both ends, slightly truncate at the base, with an inconspicuous basal scar, 0–1(–3)-septate, hyaline, with a straight or gently curved setula at each end, setulae simple, bifid or trifid, inserted terminally at the apex and subterminally at the base, conidia accumulate in slimy fascicles. **Teleomorph.** Ascomata perithecial, non-stromatic, superficial, globose to subglobose, papillate, dark brown, setose; setae sterile, rounded at the apex. Ostiole periphysate. Ascomatal wall fragile, carbonaceous, two-layered. Paraphyses disappearing with age, septate, tapering, longer than the asci. Asci unitunicate, cylindrical-clavate, shortly-stipitate, apically rounded, ascal apex with a non-amyloid apical annulus. Ascospores fusiform, hyaline, transversely septate, without gelatinous sheath or appendages.

Habitat and geographical distribution: Members of *Stilbochaeta* occur in freshwater and terrestrial habitats, they are saprobes on leaf litter, bamboo and decaying bark and wood. They are widespread throughout the world, especially in tropical and temperate regions of Africa, Asia, Australasia, the Caribbean and South America ([2,17,19,28,29,31,81,180], this study).

Notes: *Stilbochaeta* is introduced for a group of former *Codinaea* and *Dictyochaeta* species. It is morphologically and phylogenetically well-delimited and characterized by arrangement of setae and conidiophores in bundles growing from dark stromatic cells or knots of hyphal cells. Setae have sterile, bluntly rounded apices that are sometimes modified into mono- or polyphialides, while in *Codinaea* the setae are tapering toward the apices, which may be also fertile. The conidia are always septate, although aseptate and septate conidia can sometimes occur together in one strain, in contrast to morphologically similar *Codinaea* with exclusively aseptate conidia and setae. In culture, representatives of *Stilbochaeta* often form yellow, gold, cinnamon, or amber pigments that diffuse into the agar. The aerial mycelium that forms in vitro is often pigmented and the surface of the colonies is usually olivaceous cinnamon, olivaceous gold or beige-orange.

Eight species are assigned to the genus, all of which were verified using molecular data. Three other species closely resemble *Stilbochaeta* but have setae with acute, rarely obtuse apices and include *Dictyochaeta tortuosa* [7] with 1–2-septate conidia, and *D. caatingae* [121] and *D. matsushimae* [27] with 2–3-septate conidia. These species have not yet been evaluated based on molecular data. A synopsis table with diagnostic features of accepted species of *Stilbochaeta* is provided as a Supplementary File: Table S6.

**Figure 33:**
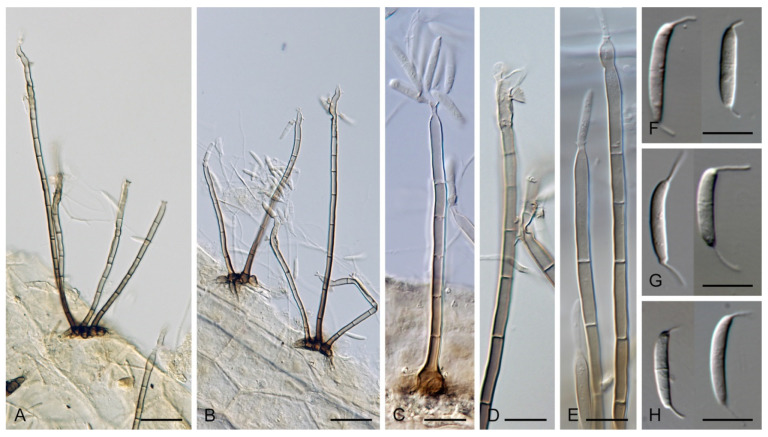
*Stilbochaeta aquatica* (CBS 114070, ex-type strain of *Dictyochaeta curvispora*). (**A**,**B**) Setae with conidiophores (**C**–**E**) conidiophores (**F**–**H**) conidia. Images: (**A**–**H**) on a stem of *U. dioica* on CMA after 6–8 weeks. Scale bars: (**A**,**B**) 25 μm (**C**–**H**) 10 μm.

**Figure 34:**
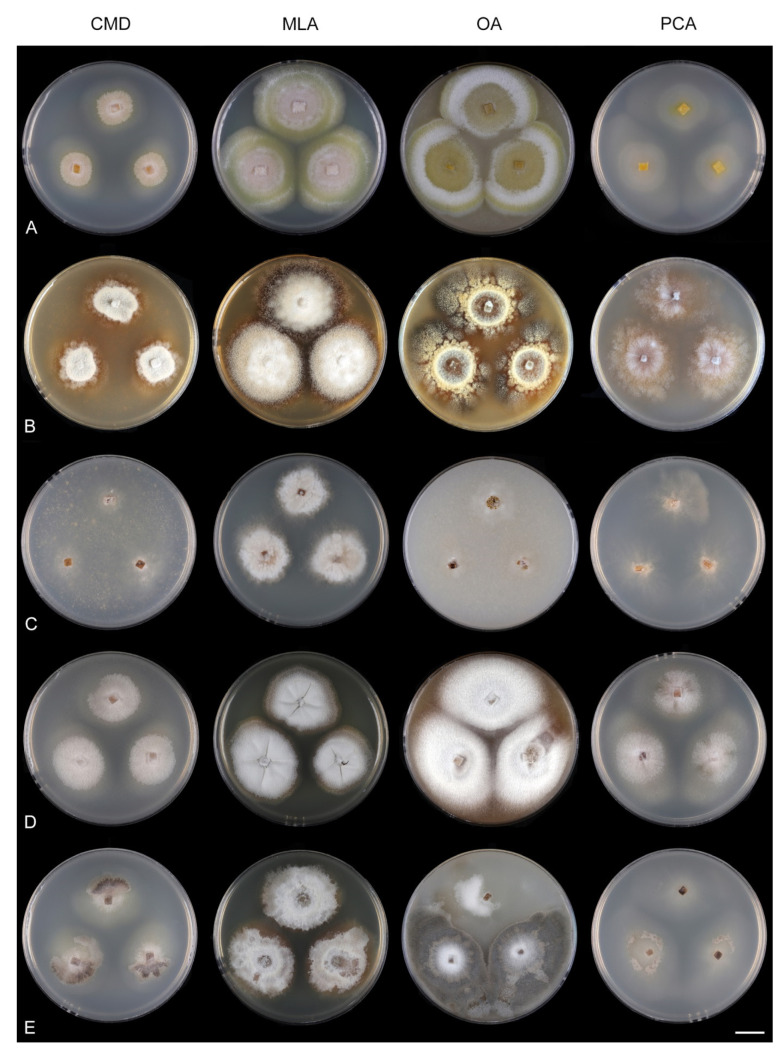
Colony morphology of *Stilbochaeta* spp. after 4 weeks. (**A**) *S. aquatica* CBS 114070 (**B**) *S. malaysiana* IMI 312436 ex-type (**C**) *S. ramulosetula* IMI 313452 ex-epitype (**D**) *S. septata* CBS 143386 ex-epitype (**E**) *S. septata* CBS 146716. Scale bar: (**A**–**E**) 1 cm.

***Stilbochaeta* *aquatica*** (W. Dong & H. Zhang) Réblová & Hern.-Restr., **comb. nov.** MycoBank MB 842213. (Figure 33 and Figure 34).

Basionym. *Dictyochaeta aquatica* W. Dong & H. Zhang, Phytotaxa 362: 193. 2018.

Culture characteristics: On CMD: colonies 28–32 mm diam, circular, flat, margin entire, velvety, somewhat floccose, mucoid at the margin, whitish with a pale apricot tinge, yellow toward the margin, with an outer zone of submerged growth, pale yellow pigment diffusing into the agar, reverse pale apricot. On MLA: colonies 49–50 mm diam, circular, flat, slightly raised centrally, velvety becoming cobwebby, mucoid at the margin, whitish with an apricot-gold tinge centrally, whitish-yellow toward the periphery, pale gold pigment diffusing into the agar, reverse gold to honey. On OA: colonies 45–48 mm diam, circular, convex centrally, flat margin, margin entire, velvety, cobwebby to mucoid at the centre, zonate, yellow centrally, white toward the margin with yellow-white outer zone, pale yellow pigment diffusing into the agar, reverse bright yellow. On PCA: colonies 52–56 mm diam, circular, flat, margin entire, mucoid, yellow-gold centrally, isabelline toward the margin, yellow pigment diffusing into the agar at the centre of the colony, reverse of the same colors. Sporulation was absent on all media.

Colonies on CMA with *U. dioica* stems effuse, hairy, mycelium composed of branched, septate, hyaline to subhyaline hyphae 1.5–2 μm diam. **Anamorph.** Setae 122–182 μm, 3.5–4.5 μm wide above the base, arise singly or in groups of two from knots of dark brown hyphal cells, erect, straight or flexuous, septate, smooth, dark brown and thick-walled, paler and thinner-walled toward the apex, unbranched, apex pale brown, with a terminal or 1–3 lateral phialidic openings. Conidiophores 45–102 μm long, 3–4 μm wide near the base, macronematous, single or arise in fascicles of 2–4 from knots of dark hyphal cells around the base of the setae, erect, straight or flexuous, septate, smooth, medium to pale brown, paler toward the tip. Conidiogenous cells 10–29 × 3–3.5 μm tapering to 1.5–2 μm below the collarette, integrated, terminal, mono-, or polyphialidic with 1–4 lateral openings, extending sympodially, occasionally percurrently, subcylindrical, pale brown, subhyaline at the apex, sometimes with persistent remnants of the collarettes; collarettes 2.5–4.5 μm wide, 2–3.5 μm deep, funnel-shaped to slightly tubular, subhyaline. Conidia 13.5–17.5 × 2.5–3 μm (mean ± SD = 16.0 ± 1.1 × 2.7 ± 0.6 μm), falcate to oblong-falcate, with an inconspicuous basal scar, 0–1-septate, hyaline, with a straight or gently curved setula at each end, 5–8.5 μm long, positioned terminally at the apex and slightly subterminally at the base, conidia accumulate in slimy whitish fascicles. **Teleomorph.** Unknown.

Specimen examined: PHILIPPINES, Negros Occidental, Bario Alegria, Lupit River, submerged bamboo stems, 18 August 2001, L. Cai (CBS 114070 ex-type strain of *Dictyochaeta curvispora*, contamination).

Habitat and geographical distribution: Saprobe on submerged wood and bamboo culm, known from Southeast Asia from the Philippines and Thailand ([28], this study). According to GlobalFungi, the identical sequences were found in one soil sample in forest in Asia (South China) [162]. The location has a temperate climate (MAT avg. 22 °C, MAP avg. 1627 mm).

Notes: Cai et al. [181] described *D. curvispora* for a hyphomycete with pigmented, unbranched conidiophores terminating into a monophialide and producing aseptate, hyaline, cylindrical, crescent-shaped to falcate conidia without setulae. According to the authors, the ex-type strain CBS 114070 did not sporulate in culture. It formed greyish-yellow colonies on potato-dextrose agar, a yellow pigment staining agar around the colony and pale orange-brown reverse. These culture characteristics, in particular the presence of the yellow pigment diffusing into the agar, correspond with our observations. However, the morphology of *D. curvispora* is not congruent with the placement of its ex-type strain among *Stilbochaeta* species. We therefore suspected that the ex-type strain might have been contaminated during isolation. Cultivation of the strain CBS 114070 on *Urtica* stems on CMA yielded sporulating colonies of a fungus with the *Stilbochaeta* morphology and provided the evidence that the ex-type strain of *D. curvispora* was indeed contaminated. Based on comparison with morphologically similar species and their DNA data, this isolate was identified as *S. aquatica*. Based on their protologues and original illustrations, *D. curvispora* and *S. aquatica* are two different species. For reasons explained above, the strain CBS 114070 cannot be used to represent *D. curvispora*; the systematic placement of this species remains unknown.

**Figure 35:**
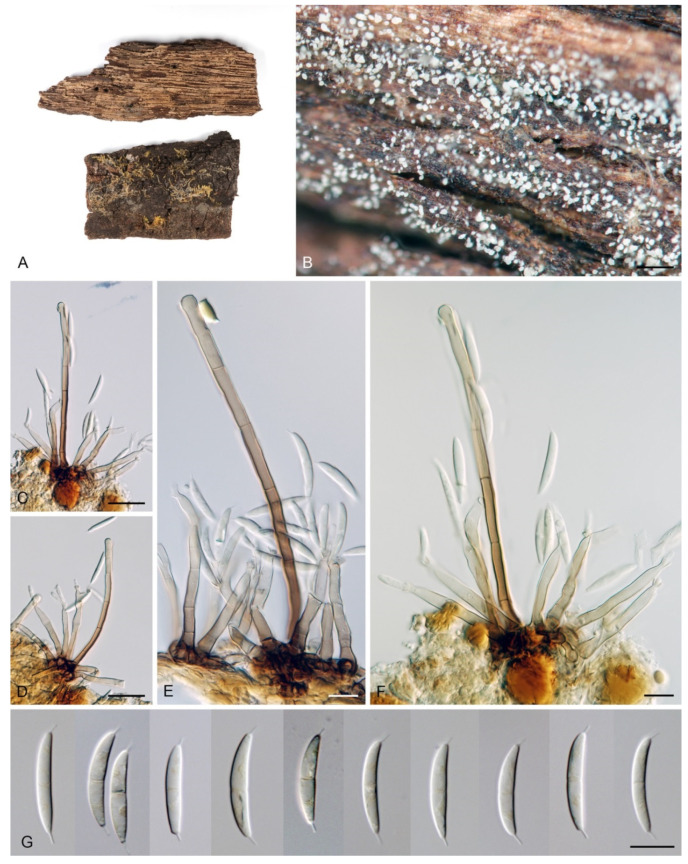
*Stilbochaeta brevisetula* (DAOM 96420a isotype). (**A**) Substrate with colonies (**B**) colony on wood (**C**–**F**) setae and conidiophores (**G**) conidia. Images: (**A**–**G**) from nature. Scale bars: (**B**) 500 μm; (**C**,**D**) 25 μm; (**E**–**G**) 10 μm.

**Figure 36:**
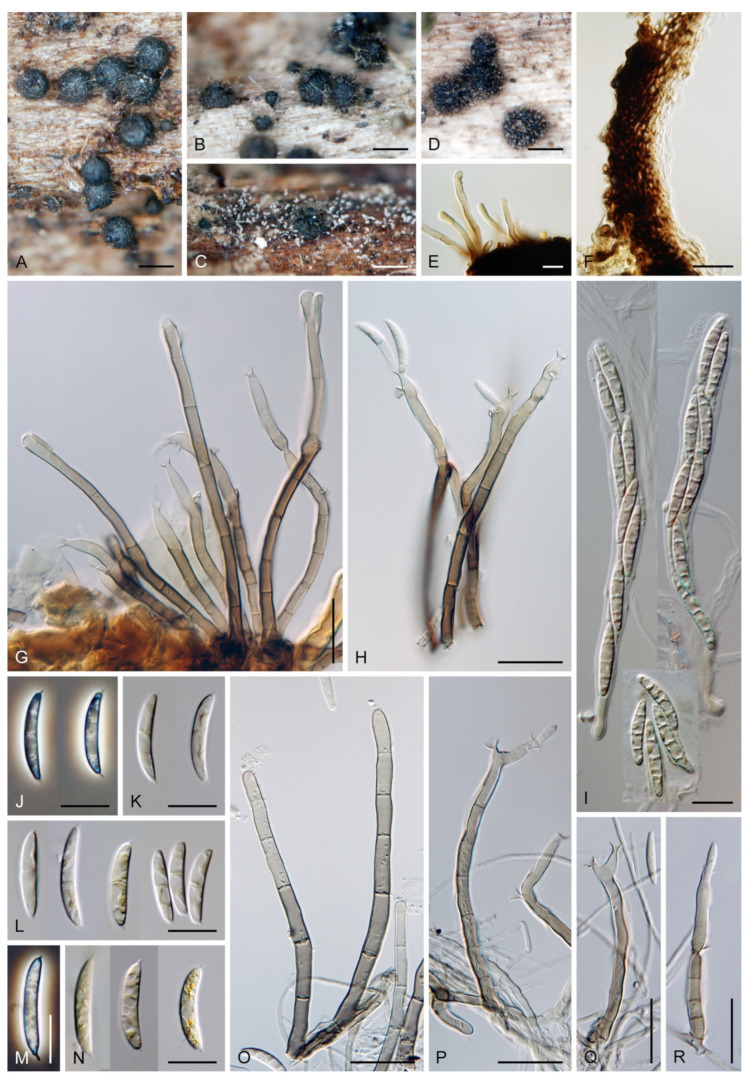
*Stilbochaeta brevisetula*. (**A**–**D**) Ascomata (**E**) setae on the ascomal wall (**F**) vertical section of the ascomal wall (**G**,**H**,**O**–**R**) setae and conidiophores (**I**) asci with ascospores (**J**–**N**) conidia. Images: (**A**–**K**) from nature (**L**–**R**) on MLA after 6–8 weeks (**A**,**L**) from ICMP 22548 (**B–E**,**G**,**H**,**J**,**K**) from ICMP 22549 ex-epitype (**F**,**I**,**M**,**N**,**R**) from ICMP 22551 (**O**–**Q**) from ICMP 22552. Scale bars: (**A**–**D**) 250 μm; (**E**–**H,O**–**R**) 20 μm; (**I**–**N**) 10 μm.

**Figure 37:**
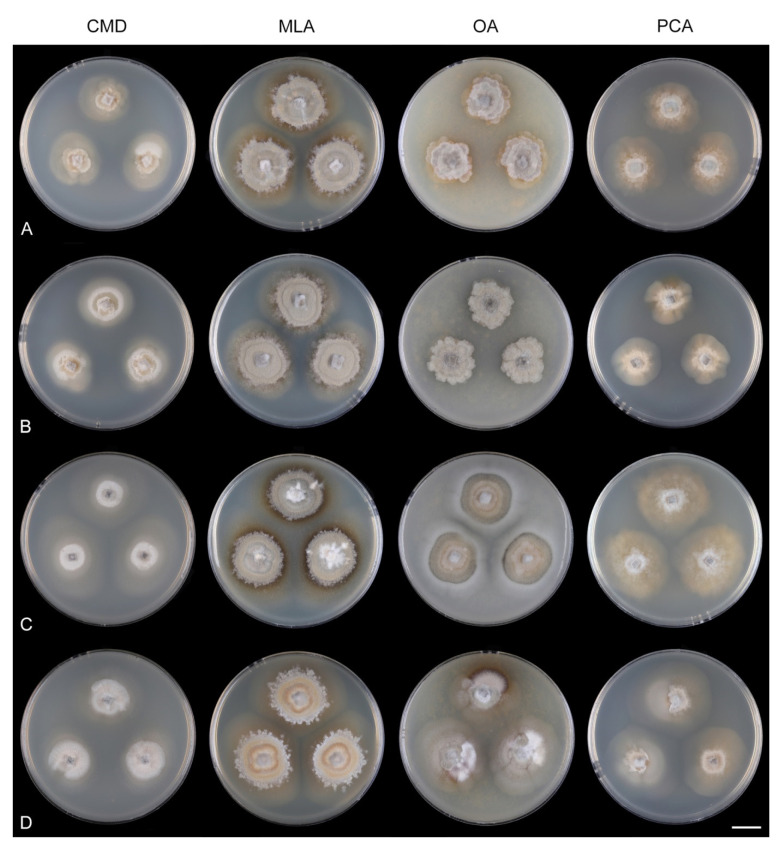
Colony morphology of *Stilbochaeta brevisetula* after 4 weeks. (**A**) ICMP 22548 (**B**) ICMP 22549 ex-epitype (**C**) ICMP 22551 (**D**) ICMP 22552. Scale bar: (**A**–**D**) 1 cm.

***Stilbochaeta brevisetula*** (S. Hughes & W.B. Kendr.) Réblová & Hern.-Restr., **comb. nov.** MycoBank MB 842214. (Figure 35, Figure 36 and Figure 37).

Basionym. *Codinaea brevisetula* S. Hughes & W.B. Kendr., N. Z. J. Bot. 6: 338. 1968.

≡ *Dictyochaeta brevisetula* (S. Hughes & W.B. Kendr.) Aramb. & Cabello, Mycotaxon 34: 681. 1989. (Nom. inval., Art. 41.4).

≡ *Dictyochaeta brevisetula* (S. Hughes & W.B. Kendr.) Whitton, McKenzie & K.D. Hyde, Fungal Divers. 4: 137. 2000.

Typification: NEW ZEALAND, Wellington region, Tongariro National Park, Ohakune Mountain Road (alt. 762 m), on bark of *Nothofagus fusca*, 7 March 1963, S.J. Hughes 439a (**holotype** of *Codinaea brevisetula* PDD 20652, **isotype** DAOM 96420a). NEW ZEALAND, Otago region, Queenstown-Lakes district, Mount Aspiring National Park, Makarora, Mt. Shrimpton track, on decaying wood of a trunk of *Nothofagus* sp., 24 March 2005, M. Réblová M.R. 3347/NZ 620 (**epitype** designated here MBT 10004617, PDD 119677, culture ex-epitype ICMP 22549).

Description on the natural substrate: Colonies effuse, grey-brown to olivaceous grey, composed of setae, conidiophores and ascomata. **Anamorph**. Setae 94–150 μm long, 3.5– μm wide above the base, single or arise in groups of 2–3 from knots of dark brown hyphal cells, erect, straight or slightly flexuous, septate, smooth, medium brown, thick-walled, darker at the base, paler and thinner walled toward the apex, unbranched, occasionally extending percurrently, apex subhyaline, bluntly rounded and slightly inflated, sterile. Conidiophores 43–100 μm long, 3.5–4.5 μm wide above the base, macronematous, arise single or in fascicles of 4–9 from knots of dark brown hyphal cells around the base of the setae, erect, straight to slightly undulating, septate, smooth, pale brown, paler toward the tip. Conidiogenous cells 16.5–32 × 3.5–4.5 μm tapering to (1.5–)2 μm below the collarette, integrated, terminal, mono- or polyphialidic with 1–3 lateral openings, extending percurrently and sympodially, cylindrical, pale brown, subhyaline at the apex; collarettes 3.5–4 μm wide, 2–2.5(–3) μm deep, funnel-shaped, subhyaline. Conidia 17–22 × 3–4 μm (mean ± SD = 19.2 ± 1.6 × 3.3 ± 0.3 μm), oblong-falcate, slightly tapering toward both ends, with an inconspicuous basal scar, 1-septate, hyaline, with a straight or gently curved setula at each end, 1–1.5 μm long, inserted terminally at the apex and subterminally at the base, conidia accumulate in slimy whitish fascicles. **Teleomorph**. Ascomata 250–330 μm diam, non-stromatic, superficial, globose to subglobose, papillate, dark brown, clothed with setae that are similar to those accompanying the conidiophores of the anamorph, becoming glabrous with age; setae yellow-brown, pale brown to subhyaline toward the apex, apex bluntly rounded, slightly inflated, sterile. Ostiole periphysate. Ascomatal wall fragile, carbonaceous, 24–30 μm thick, two-layered. Outer layer consisting of brown, polyhedral cells with opaque walls. Inner layer consisting of several rows of thin-walled, elongated, hyaline cells. Paraphyses 3.5–5 μm wide tapering to 1.5–3 μm, septate, longer than the asci. Asci 100–114 × 7.5–8.5 μm (mean ± SD = 108.0 ± 4.9 × 8.0 ± 0.5 µm), 83–98 μm (mean ± SD = 92.0 ± 4.4 µm) long in the sporiferous part, cylindrical-clavate, stipitate, apically rounded, ascal apex with a shallow, non-amyloid apical annulus 2.5–3 μm wide, 1–1.5 μm high. Ascospores (15–)16–24 × 3–4(–4.5) μm (mean ± SD = 18.7 ± 2.1 × 3.4 ± 0.3 µm), fusiform, hyaline, 3-septate, 2-seriate or obliquely uniseriate in the ascus.

Culture characteristics: On CMD: colonies 32–35 mm diam, circular, flat, raised centrally, margin entire, velvety-lanose, mucoid locally, cobwebby at the margin, with a prominent outer zone of submerged growth, finely furrowed, white to pale ochre-beige, isabelline at the margin, reverse pale olivaceous beige. On MLA: colonies 33–36 mm diam, circular, flat, convex centrally, margin fimbriate, lanose, olivaceous ochre to olivaceous cinnamon, with an olivaceous brown to apricot outer zone of submerged growth, pale ochre-brown pigment diffusing into agar, reverse dark olivaceous orange. On OA: colonies 32–34 mm diam, circular, convex, margin entire to undulate, lanose, somewhat floccose, aerial mycelium occasionally reduced, colonies cobwebby to mucoid, pale olivaceous apricot to olivaceous grey-brown with an orange tinge, pale ochre/apricot pigment diffusing into agar, reverse olivaceous-ochre to cinnamon, isabelline at the margin. On PCA: colonies 30–32 mm diam, circular, flat, raised centrally, margin undulate, lanose, cobwebby toward the periphery, white-beige at the centre, pale beige-apricot to pale sepia toward the periphery with an outer zone of submerged growth, pale apricot pigment diffusing into agar, reverse olivaceous apricot. Sporulation was abundant on MLA, OA, moderate on PCA, absent on CMD.

Colonies on OA effuse, hairy, mycelium composed of branched, septate, subhyaline to hyaline hyphae 2–3 μm diam. **Anamorph**. Setae, conidiophores, conidiogenous cells and conidia similar to those from nature. Setae 116–132 μm long, 4–5 μm wide above the base, extending percurrently, sterile. Conidiophores 49–87(–105) μm long, 3.5–4.5 μm wide above the base, arise singly or in small groups, interspersed among the setae. Conidiogenous cells 14–34 × 3.5–5 μm tapering to 2–2.5 μm below the collarette, integrated, terminal, mono- or polyphialidic with 1–2 lateral openings, extending percurrently and sympodially, cylindrical, pale brown, subhyaline at the apex; collarettes 4–4.5 μm wide, 2–2.5 μm deep, funnel-shaped, subhyaline. Conidia (15.5–)17–22 × 3–4 μm (mean ± SD = 19.2 ± 1.6 × 3.4 ± 0.3 μm), 1-septate, setulae 1–1.5 μm long, accumulate in slimy whitish fascicles. **Teleomorph**. Not observed.

Specimens examined: NEW ZEALAND, Otago region, Queenstown-Lakes district, Mount Aspiring National Park, Makarora, Mt. Shrimpton track, on decaying wood of *Nothofagus* sp., 24 March 2005, M. Réblová M.R. 3339/NZ 611 (PDD 119678, ICMP 22548); *Ibid.*, M.R. 3338/NZ 610, M.R. 3344/NZ 616, M.R. 3349/NZ 622. NEW ZEALAND, Otago region, Queenstown-Lakes District, Makarora, Makarora Bush Walk, on decaying wood of a trunk, 30 March 2005, M. Réblová M.R. 3410/NZ 691 (PDD 119679, ICMP 22551). NEW ZEALAND, West Coast region, Buller district, Victoria Forest Park, Lyell, Lyell Historical Walk, on decaying wood of a branch, 6 April 2005, M. Réblová M.R. 3455/NZ 742 (PDD 119680, ICMP 22552). NEW ZEALAND, West Coast region, Buller district, Victoria Forest Park, Murray Creek Track, on decaying wood of *Nothofagus* sp., 21 February 2003, M. Réblová & K.A. Seifert M.R. 2587/NZ 49 (PDD 81887). NEW ZEALAND, West Coast region, Buller district, Victoria Forest Park, Palmer’s Hut ca. 18 km SW of Springs Junction on unpaved road, Lake Christabel track, on decaying wood of *Nothofagus* sp., 28 February 2003, M. Réblová M.R. 3423/NZ 162 (PDD 119681, ICMP 15125). NEW ZEALAND, West Coast region, Westland district, Otira, Kelly Shelter, Cockayane Nature Walk MR, on decaying wood, 11 April 2005, M. Réblová M.R. 3466/NZ 754.

Habitat and geographical distribution: Saprobe on decaying bark and wood of *Nothofagus fusca*, *Nothofagus* sp. and other unidentified hosts, known only from Australasia from New Zealand ([2], this study). The database GlobalFungi does not contain similar sequences (≥98%) of this species.

Notes: *Stilbochaeta brevisetula* is the only species of the genus known to have a teleomorph, which is reported for the first time in this study. Ascomata are often covered by setae that resemble those associated with the conidiophores of the anamorph, although with age the setae disappear and ascomata become glabrous. The anamorph-teleomorph relationship was experimentally verified. *Stilbochaeta brevisetula* is well distinguishable from other members of the genus by the shortest conidial setulae (1–2.7 μm) and setae that are sterile, apically bluntly rounded and slightly inflated.

The teleomorph of *S. brevisetula* is remarkably similar to *Chaetosphaeria (Ch.*) *hebetiseta* [182], a saprobic lignicolous species with a *Chloridium*-like anamorph. Their ascomata are covered with always sterile, brown, septate setae that terminate into rounded subhyaline cells and they also share 3-septate, hyaline, fusiform ascospores of comparable size. However, the ascospores of *S. brevisetula* are smooth-walled, while those of *Ch. hebetiseta* are verrucose, also their anamorphs clearly differ in characters of conidia. The two species are shown distantly related in the phylogenetic tree (Figure 2).

***Stilbochaeta cangshanensis*** (Z.L. Luo, K.D. Hyde & H.Y. Su) Réblová & Hern.-Restr., **comb. nov.** MycoBank MB 842215.

Basionym. *Dictyochaeta cangshanensis* Z.L. Luo, K.D. Hyde & H.Y. Su, Fungal Divers. 99: 592. 2019.

For description and illustrations, see Luo et al. [29].

Habitat and geographical distribution: Saprobe on submerged decaying wood, known only from Asia, China [29]. According to GlobalFungi, the identical sequences were found in 13 soil and one shoot samples collected in forests of Europe (five samples, South Italy), Asia (seven samples, Iran, South China), and Eastern North America (one sample, Tennessee) [115,117,162,183,184]. The sites have temperate or Mediterranean climate (MAT avg. 17 °C, MAP avg. 842 mm).

Notes: This species closely resembles *S. aquatica* and it is difficult to distinguish them based solely on morphological characters, especially when the size of their conidia significantly overlap, i.e., 14–18 × 2–3 μm with setulae 8–13 μm long in *S. aquatica* [28] vs. 15–18 × 2.5–3.5 μm in *S. cangshanensis* [29]; the length of the setulae of the latter species were not given in the protologue. In the phylogenetic analysis, the two species form distinct lineages.

**Figure 38:**
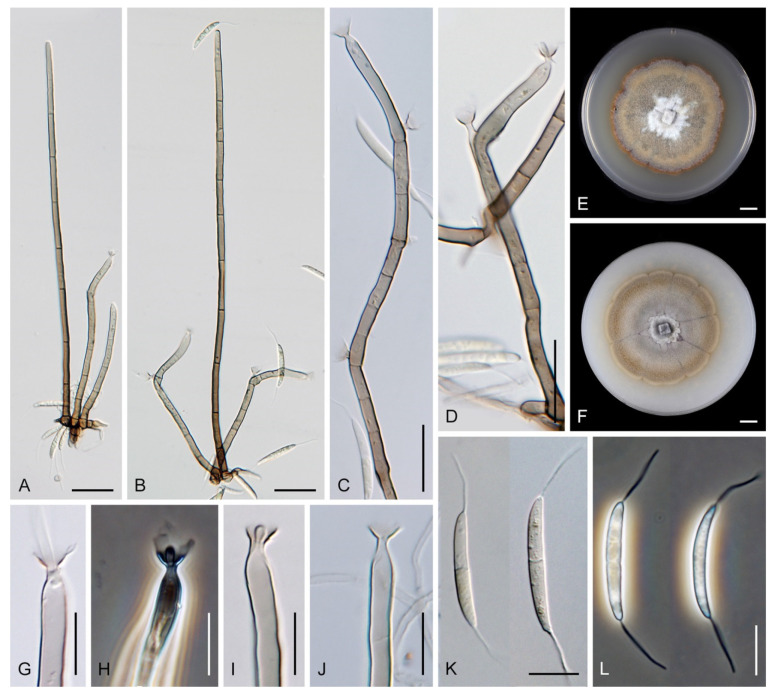
*Stilbochaeta malaysiana* (IMI 312436 ex-type). (**A**,**B**) Setae and conidiophores (**C**,**D**) conidiophores with conidiogenous cells, in detail (**E**,**F**) colonies (**G**–**J**) tip of the conidiogenous cells with collarettes (**K**,**L**) conidia. Images: (**A**–**D**,**G**–**L**) on CMD after 4–6 weeks (**E**) on MLA after 4 weeks (**F**) on OA after 4 weeks. Scale bars: (**A**,**B**) 25 μm; (**C**,**D**) 20 μm; (**E**,**F**) 1 cm; (**G**–**L**) 10 μm.

***Stilbochaeta malaysiana*** (Kuthub.) Réblová & Hern.-Restr., **comb. nov.** MycoBank MB 842216. (Figure 34 and Figure 38).

Basionym. *Dictyochaeta malaysiana* Kuthub., Trans. Br. mycol. Soc. 89: 356. 1987.

Culture characteristics: On CMD: colonies 24–25 mm diam, circular, raised, margin undulate, lanose, cobwebby at the margin, grey-beige centrally, beige toward the margin, ochre pigment diffusing into agar, reverse dark cinnamon. On MLA: colonies 62–63 mm diam, circular, raised, flat margin, margin fimbriate, lanose, zonate, finely furrowed, whitish-beige becoming olivaceous ochre-beige toward the periphery, dark cinnamon at the margin, caramel pigment diffusing into the agar, reverse dark cinnamon. On OA: colonies 61–62 mm diam, circular, raised, margin lobate, lanose, floccose, furrowed, zonate with a central cobwebby ring surrounded by small lanose areas of radially spreading mycelium, ochre-grey centrally, pale ochre-beige to olivaceous cinnamon toward the periphery, aerial hyphae with numerous pale ochre exudates, dark cinnamon pigment diffusing into the agar, reverse dark amber to brown. On PCA: colonies 32–38 mm diam, flat, circular, margin undulate, sparsely lanose to cobwebby, ochre-beige, pale cinnamon pigment diffusing into the agar, reverse cinnamon. Sporulation was moderate on CMD and PCA, absent on MLA and OA.

Colonies on CMA with *U. dioica* stems effuse, hairy, mycelium composed of branched, septate, hyaline to subhyaline hyphae 1.5–3 μm diam. **Anamorph.** Setae 230–281 μm long, 4–5.5 μm wide near the swollen base, single or arise in groups of two from knots of dark brown hyphal cells, erect, straight or flexuous, septate, smooth, dark brown and thick-walled, paler and thinner-walled toward the apex, unbranched, apex pale brown, sterile. Conidiophores 78–113 μm long, 4–5.5 μm wide near the base, macronematous, single or arise in fascicles of 2–5 from knots of dark brown hyphal cells around the base of the setae, erect, straight or bent, septate, smooth, medium to pale brown, paler toward the tip. Conidiogenous cells 17–21(–37) × 3.5–4.5(–5) μm tapering to 1.5–2 μm below the collarette, integrated, terminal, mono- or polyphialidic with 1–3 lateral openings while internally septa can be formed, extending percurrently and sympodially, subcylindrical, pale brown; collarettes 4.5–7 μm wide, 3.5–4.5 μm deep, funnel-shaped to slightly campanulate, tubular at the base, pale brown. Conidia 21–26 × 2.5–3.5 μm (mean ± SD = 23.6 ± 1.3 × 3.0 ± 0.2 μm), falcate, with an inconspicuous basal scar, 1-septate, hyaline, with a straight or gently curved setula at each end, 12–18 μm long, positioned terminally at the apex and slightly subterminally at the base, conidia accumulate in slimy whitish fascicles. **Teleomorph.** Unknown.

Specimen examined: MALAYSIA, Selangor, Kepong Forest Reserve, on decaying leaves, October 1986, A.J. Kuthubutheen KUM 534 (**holotype** IMI 312436, ex-type strain IMI 312436).

Habitat and geographical distribution: Saprobe on decaying leaves known from Southeast Asia, Malaysia [19]. According to GlobalFungi, the identical sequences were found in 13 soil samples collected in forests of the Caribbean (12 samples, Puerto Rico, Trinidad and Tobago [119,185] and South Asia (one sample, India) [117]. The locations have tropical climate (MAT avg. 22 °C, MAP 3124 mm).

Notes: Kuthubutheen [19] listed two specimens with different localities in the protologue, however, only one number to indicate the holotype IMI 312436. By comparing the protologue with the type material deposited in the Kew herbarium, it was found that the locality of a specimen listed second in order is the place of collection of the holotype. Kuthubutheen [19] did not indicate an ex-type strain of his new species. Strain of *S. malaysiana*, with identical designation IMI 312436 as the holotype and accompanying metadata, deposited by A.J. Kuthubutheen, was obtained from the CABI culture collection. We confirm its identity and connect the holotype with the ex-type strain in this study.

*Stilbochaeta novae-guineensis* closely resembles *S. malaysiana,* but differs from it by shorter conidia. *Stilbochaeta aquatica* is also comparable with *S. malaysiana*, but differs in smaller 0–1-septate conidia.

**Figure 39:**
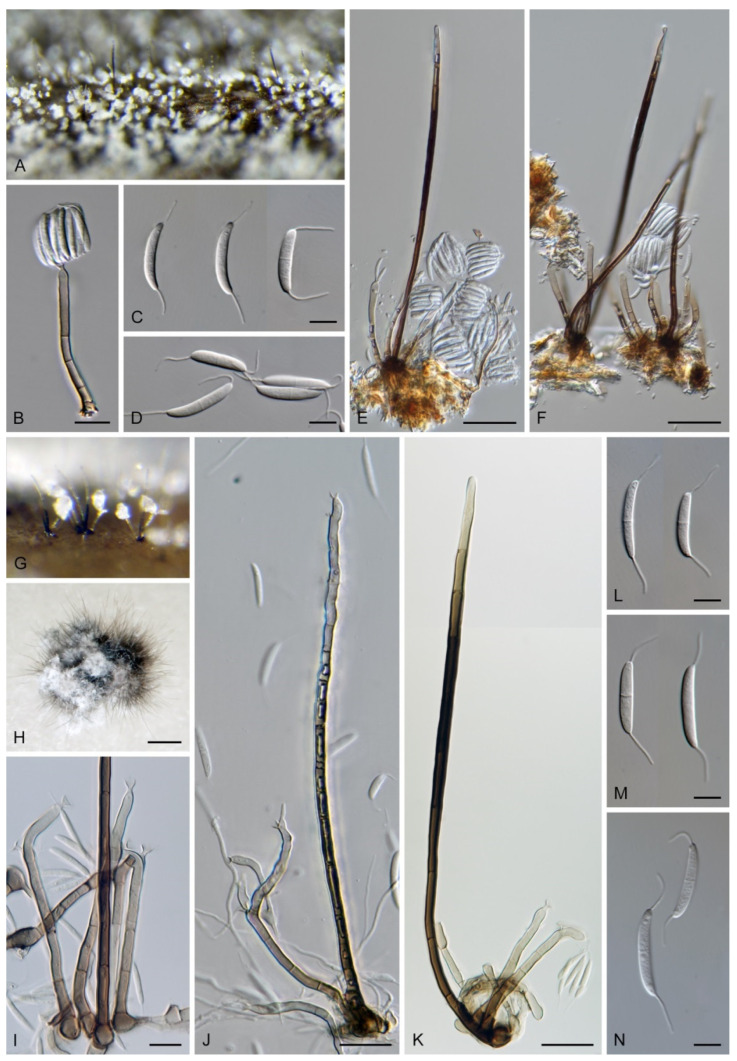
*Stilbochaeta novae-guineensis*. (**A**,**G**) Colonies (**B**,**I**) conidiophores (**C**,**D**,**L**–**N**) conidia (**E**,**F**,**J**,**K**) setae with conidiophores (**H**) setae and conidiophores arranged in a sporodochium-like. Images: (**A**–**F**) from nature (**G**,**J**,**L**–**N**) on a stem of *U. dioica* on CMA after 4 weeks (**H**,**I**,**K**) on OA after 8 weeks (**A**,**G**,**J**,**L**–**N**) from CBS 147515 (**H**,**I**,**K**) from CBS 147517. Scale bars: (**B**,**I**) 10 μm; (**C**,**D**,**L**–**N**) 5 μm; (**E**,**F**) 25 μm; (**H**) 500 μm; (**J**,**K**) 20 μm.

**Figure 40:**
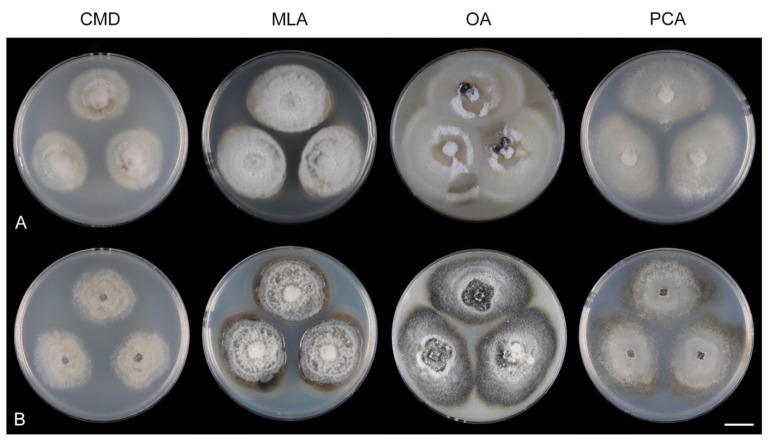
Colony morphology of *Stilbochaeta novae-guineensis* after 4 weeks. (**A**) CBS 147515 (**B**) CBS 147517. Scale bar: (**A**,**B**) 1 cm.

***Stilbochaeta* *novae-guineensis*** (Matsush.) Réblová & Hern.-Restr., **comb. nov.** MycoBank MB 842217 (Figure 39 and Figure 40).

*Basionym*. *Codinaea novae-guineensis* Matsush., Microfungi of the Solomon Islands and Papua-New Guinea: 14. 1971.

≡ *Dictyochaeta novae-guineensis* (Matsush.) A.I. Romero, Boln Soc. argent. Bot. 22: 76. 1983.

Description on the natural substrate: Colonies effuse, brown, composed of setae and conidiophores, mycelium semi-immersed. **Anamorph.** Setae 95–162 μm long, 3–5 μm wide near the swollen base, arise singly or in groups of two from dark brown stromatic cells, erect, straight or flexuous, septate, smooth, dark brown and thick-walled, paler and thinner-walled toward the apex, unbranched, apex pale brown, sterile or with a terminal phialidic opening. Conidiophores 27.5–58 μm long, 3–5 μm wide near the base, macronematous, arise singly or in groups of 5–7 from stromatic cells around the base of the setae, erect, straight or slightly bent, septate, smooth, medium to pale brown, paler toward the tip. Conidiogenous cells 17–30 × 2.5–5 μm tapering to 1–2 μm below the collarette, integrated, terminal, mono- or polyphialidic with 1–2(–3) lateral opening, extending sympodially, cylindrical, medium to pale brown; collarettes 2.5–5 μm wide, 1.5–3.5 μm deep, funnel-shaped, pale brown, the apical part often evanescent or broken off and attached to the conidial mass. Conidia 14–19 × 2–4 μm (mean ± SD = 17.1 ± 3.1 × 1.6 ± 0.3 μm), falcate to oblong-falcate, with an inconspicuous basal scar, 0–1-septate, hyaline, with a straight or gently curved setula at each end, 6–12 μm long, conidia accumulate in slimy whitish fascicles. **Teleomorph.** Unknown.

Culture characteristics: On CMD: colonies 25–25 mm diam, circular, flat, margin fimbriate, lanose to cobwebby, zonate, beige-brown to isabelline with darker concentric rings, reverse dark ivory. On MLA: colonies 32–35 mm diam, circular, convex, margin entire, lanose, floccose, finely furrowed centrally, whitish-grey to grey-brown, with a prominent outer zone of submerged growth, reverse olivaceous brown. On OA: colonies 49–68 mm diam, circular, raised, margin entire, velvety-lanose, floccose, with a central zone of sparse growth, whitish to dark grey-brown, sometimes the dark pigment in subsurface mycelium is absent, colonies becoming partially mucoid, whitish-beige, reverse pale brown to dark olivaceous grey. On PCA: colonies 49–52 mm diam, circular, flat, margin fimbriate to lobate, mucoid centrally, cobwebby toward the margin, isabelline to whitish-beige, pale ochre-brown at the margin, reverse of the same colors. Sporulation was moderate on MLA, OA and PCA, absent on CMD.

Colonies on CMA with *U. dioica* stems effuse, hairy, mycelium composed of branched, septate, hyaline to subhyaline hyphae 2–3 μm diam. **Anamorph.** Setae, conidiophores, conidiogenous cells and conidia similar to those from nature. Setae 100–202 μm long, 3–5 μm wide near the base, sterile or with a terminal or several lateral phialidic openings. Conidiophores 24–92 μm long, 3–5 μm wide near the base, arise in fascicles of 2–5 from knots of dark hyphal cells around the base of the setae, unbranched. Conidiogenous cells 10–30 × 2.5–5 μm tapering to 1.5–2 μm below the collarette, mono- or polyphialidic with 1–5 lateral openings while internally septa can be formed, pale brown to subhyaline; collarettes 2–4 μm wide, 1.5–3.5 μm deep, funnel-shaped, subhyaline. Conidia 14.5–33 × 2–3.5 μm (mean ± SD = 19.86 ± 2.97 × 3.42 ± 0.34 μm), falcate to oblong-falcate, 0–1-septate, setulae 3–14 μm long, accumulate in slimy whitish fascicles. **Teleomorph.** Unknown.

Specimens examined: PUERTO RICO, on decaying wood, 19 July 2018, M. Hernández-Restrepo M.H.R. 18004 (CBS H-24746, culture CBS 147515); *Ibid*., on decaying twig, 19 July 2018, M. Hernández-Restrepo M.H.R. 18018 (CBS H-24748, culture CBS 147517).

Habitat and geographical distribution: Saprobe on decaying leaves, wood and bark of *Castanopsis* sp. and other unidentified hosts, found in the tropical geographical areas of Africa, Australasia, the Caribbean and South America: Brazil, Cuba, Ivory Coast, Puerto Rico and Solomon Islands ([17,121,134,146,147,180,186], this study).

According to GlobalFungi, the identical sequences were found in six soil samples in forests of South America (Peru) [116] and the Caribbean (Puerto Rico) [119]. The sites have tropical climate (MAT avg. 22 °C, MAP avg. 3059 mm).

Notes: Onofri and Zucconi [180] emended the species diagnosis with polyphialidic conidiogenous cells, which were described as exclusively monophialidic in the protologue [17]. In our material of *S. novae-guineensis*, the conidiogenous cells were predominantly monophialidic, occasionally with 1–2(–3) lateral phialidic apertures. When grown in culture, the polyphialides with up to five lateral openings formed frequently. *Stilbochaeta malaysiana* resembles *S. novae-guineensis* but differs in longer conidia. It is challenging to distinguish *S. novae-guineensis* from *S. septata* as the size of their conidia considerably overlaps; for their comparison see notes to the latter species.

**Figure 41:**
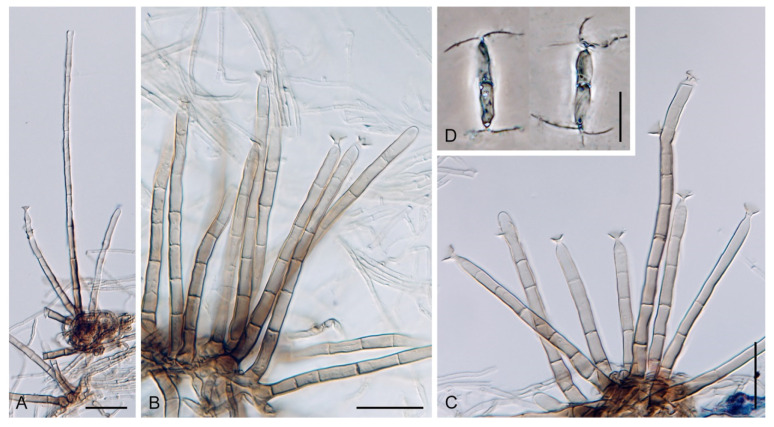
*Stilbochaeta ramulosetula* (IMI 313452 ex-epitype). (**A**) Seta with conidiophores (**B**,**C**) conidiophores (**D**) conidia. Images: (**A**–**C**) on CMD after 5 months. Scale bars: (**A**) 25 μm; (**B**,**C**) 20 μm; (**D**) 10 μm.

***Stilbochaeta ramulosetula*** (Kuthub.) Réblová & Hern.-Restr., **comb. nov.** MycoBank MB 842218. (Figure 34 and Figure 41).

Basionym. *Dictyochaeta ramulosetula* Kuthub., Trans. Br. mycol. Soc. 89: 353. 1987.

Typification: MALAYSIA, Pahang, Lepar Forest Reserve, on decaying submerged leaf, July 1986, A. J. Kuthubutheen KUM 515 (**holotype** IMI 312058). MALAYSIA, Pahang, Lepar Forest Reserve, on decaying submerged leaf, July 1986, A. J. Kuthubutheen (**epitype** designated here MBT 10004619, IMI 313452 dried culture and microscopic slide, ex-epitype culture IMI 313452).

Culture characteristics: On CMD: colonies 29–31 mm diam, circular, flat, margin fimbriate, cobwebby, mucoid toward the periphery, whitish, reverse isabelline. On MLA: colonies 26–30 mm diam, circular, raised, fimbriate to weakly undulate, velvety-lanose, floccose, finely furrowed, whitish or with irregular salmon patches at the centre, ochre-beige at the margin, pale ochre pigment diffusing into agar, reverse pale ochre-beige. On OA: colonies 17–20 mm diam, circular, flat, margin entire, velvety to cobwebby, aerial mycelium on the inoculation block with numerous cinnamon exudates, whitish, pale ochre pigment diffusing into agar, reverse pale cinnamon. On PCA: colonies 27–33 mm diam, circular, flat, margin rhizoidal, cobwebby becoming mucoid, ochre-beige, reverse of similar color. Sporulation was moderate on CMD, absent on MLA, OA, and PCA.

Colonies on CMD effuse, hairy, mycelium composed of branched, septate, hyaline hyphae 1.5–3 μm diam. **Anamorph.** Setae 170–225 μm long, 4.5–5 μm wide near the base, single or arise in groups of two from dark hyphal stromata, erect, straight or flexuous, septate, smooth, brown and thick-walled, paler and thinner-walled toward the apex, unbranched, apex subhyaline, bluntly rounded, sterile, occasionally terminating into a monophialide. Conidiophores 46–97 μm long, 4–5 μm wide near the base, macronematous, arise in fascicles of 3–10 from dark hyphal cells, usually aggregated around the base of the setae, erect, straight or flexuous, septate, smooth, pale brown, paler toward the tip. Conidiogenous cells 12.5–19(–33) × 3.5–4.5 μm tapering to 1.5–2 μm below the collarette, integrated, terminal, mono- rarely polyphialidic with one lateral opening while internally septa can be formed, extending sympodially, rarely percurrently, cylindrical, pale brown, subhyaline at the apex; collarettes 4.5–6.5 μm wide, 1.5–3.5 μm deep, widely funnel-shaped, slightly stipitate, pale brown to subhyaline. Conidia 16–19.5 × 2.5–3.5 μm (mean ± SD = 17.9 ± 1.1 × 3.0 ± 0.4 μm), oblong-falcate, 1-septate, slightly constricted at the septum, hyaline, rounded at each end, with a bifid setula at each end, occasionally with a bifid setula at one end and a trifid setula at the other, setulae straight or gently curved, 8–15 μm long, positioned terminally, conidia accumulate in slimy whitish fascicles. **Teleomorph.** Unknown.

Habitat and geographical distribution: Saprobe on submerged leaves, known from Malaysia only [19]. According to GlobalFungi, the identical sequences were found in two soil samples from the forest biome in the Caribbean (Trinidad and Tobago) [185]. The site has a tropical climate (MAT avg. 26 °C, MAP avg. 2511 mm).

Notes: Kuthubutheen [19] listed two specimens in the protologue of *S. ramulosetula* but only one number to indicate the holotype IMI 312058. Based on comparison with the holotype material in the Kew herbarium, the locality of the first specimen is the place of collection of the holotype. An unpublished strain of *S. ramulosetula* IMI 313452, collected in the same locality as the holotype and deposited by A.J. Kuthubutheen, was obtained from the CABI culture collection. A specimen in the Kew herbarium with the identical accession number, deposited as the microscopic slide and dried culture, is selected as the epitype in this study.

*Stilbochaeta ramulosetula* is well distinguished from other members of the genus by bifid and trifid setulae inserted at each end of conidia. It is closely related to *S. malaysiana* and *S. novae-guineensis*; these species share 1-septate conidia and setae that are predominantly sterile but occasionally terminate into a monophialide.

**Figure 42:**
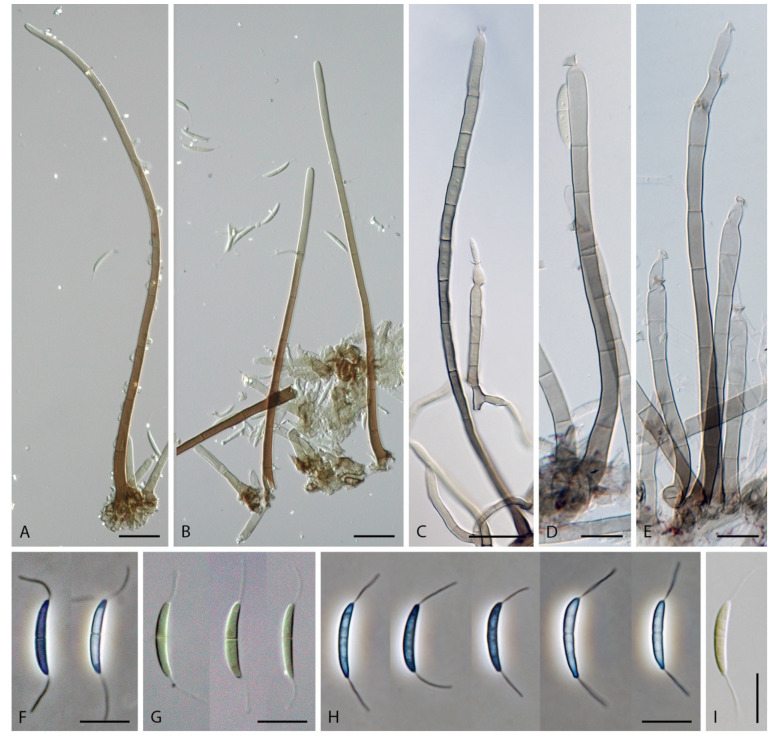
*Stilbochaeta septata* (CBS 146716). (**A**–**E**) Setae with conidiophores (**F**–**I**) conidia. Images: (**A**,**B**,**F**,**G**) from nature (**C**,**H**,**I**) on PCA after 4 weeks (**D**,**E**) on OA after 4 weeks. Scale bars: (**A**–**C**) 20 μm; (**D**–**I**) 10 μm.

***Stilbochaeta septata*** (B. Sutton & Hodges) Réblová & Hern.-Restr., **comb. nov.** MycoBank MB 842219. (Figure 34 and Figure 42).

Basionym. *Codinaea septata* B. Sutton & Hodges, Nova Hedw. 26: 520. 1975.

≡ *Dictyochaeta septata* (B. Sutton & Hodges) Aramb. & Cabello, Mycotaxon 34: 682. 1989. (Nom. inval., Art. 41.4).

≡ *Dictyochaeta septata* (B. Sutton & Hodges) Whitton, McKenzie & K.D. Hyde, Fungal Divers. 4: 148. 2000.

Description on the natural substrate: Colonies effuse, hairy, brown composed of setae and conidiophores, mycelium immersed. **Anamorph.** Setae 245–260 μm long, 3–5 μm wide near the base, arise singly or in groups of 2–3 from knots of dark brown hyphal cells, erect, straight or flexuous, septate, smooth, dark brown and thick-walled, paler and thinner-walled toward the apex, unbranched, apex subhyaline to hyaline, sterile, bluntly rounded. Conidiophores macronematous, unbranched, usually reduced to sessile or stalked conidiogenous cells, 34–37 × 3–4 μm tapering to ca. 1.5 μm below the collarette, arise singly or in groups of 2–6 from knots of hyphal cells around the base of the setae, smooth, integrated, terminal, mono- occasionally polyphialidic with one lateral aperture, extending sympodially, cylindrical, pale brown to subhyaline, paler toward the tip; collarettes 2.5–3 μm wide, ca. 2 μm deep, funnel-shaped, subhyaline. Conidia 14–15.5 × 2–2.5(–3) μm (mean ± SD = 14.9 ± 0.6 × 2.3 ± 0.2 μm), falcate, tapering toward both ends, with an inconspicuous basal scar, 1-septate, hyaline, with a straight or gently curved setula at each end 5.5–9 μm long, inserted terminally at the apex, subterminally at the base, conidia accumulate in slimy whitish fascicles. **Teleomorph.** Unknown.

Culture characteristics: On CMD: colonies 28–30 mm diam, circular, flat, margin entire to weakly fimbriate, velvety-lanose, mucoid at the margin, white becoming irregularly beige to brown toward the periphery due to densely aggregated sporulating conidiophores, isabelline at the margin, with a prominent outer zone of submerged growth, reverse pale ochre, isabelline toward the periphery. On MLA: colonies 33–34 mm diam, circular, raised or crateriform, margin entire, velvety-lanose, furrowed, later developing cracks in folds, white, sepia at the margin, pale ochre pigment diffusing into the agar, reverse dark cinnamon. On OA: colonies 49–50 mm diam circular, raised, margin entire, velvety-lanose, locally mucoid, smooth, white becoming ash grey due to densely aggregated sporulating conidiophores, cinnamon at the margin, sometimes pale cinnamon pigment diffusing into agar, reverse ochre-brown. On PCA: colonies 48–49 mm diam, circular, flat, margin entire, velvety becoming cobwebby, mucoid toward the periphery, white-ochre, pale olivaceous isabelline at the margin, with a zone of submerged growth, reverse ochre. Sporulation was abundant on OA and PCA, moderate on MLA, absent on CMD.

Colonies on CMD effuse, hairy, mycelium composed of branched, septate, hyaline to subhyaline hyphae 1.5–3 μm diam. **Anamorph.** Setae similar to those from nature, 138–192 μm long, 3.5–4.5 μm wide near the base, arise in groups from knots of dark brown cells, unbranched, apex pale brown to subhyaline, sterile, bluntly rounded or terminating into a mono- or polyphialide. Conidiophores 34–76(–92) μm long, 3–4.5 μm wide near the base, macronematous, single or arise in fascicles of 2–4 from knots of dark hyphal cells around the base of the setae, erect, straight or flexuous, septate, smooth, unbranched, brown, paler toward the tip. Conidiogenous cells 20–26.5 × 3.5–4 μm tapering to 1.5–2 μm below the collarette, integrated, terminal, monophialidic, occasionally polyphialidic with 1–2 lateral openings, extending percurrently and sympodially, subcylindrical, pale brown, subhyaline toward the tip; collarettes 3.5–4 μm wide, 1.5–2.5 μm deep, funnel-shaped, subhyaline. Conidia similar to those from nature, 13.5–16 × 2–3 μm (mean ± SD = 14.7 ± 0.8 × 2.3 ± 0.3 μm), falcate, 1-septate, setulae 6.5–10 μm long, accumulate in slimy whitish fascicles. **Teleomorph.** Unknown.

Specimens examined: AUSTRALIA, New South Wales, Yarramulong, Nunkeri Native Flowers, on a fallen leaf of *Melaleuca viminalis*, 23 August 1999, K.A. Seifert K.A.S. 1112 (culture CBS 146716). CHILE, on leaves of *Eucalyptus grandis* × *urophylla*, June 2010, M.J. Wingfield (culture ex-epitype CBS 143386).

Habitat and geographical distribution: Saprobe on leaves of *Eucalyptus grandis*, *E. grandis* × *urophylla*, *Eucalyptus* sp. and *Melaleuca viminalis*, known from Africa, Australasia and South America: Australia, Brazil, Chile, and South Africa ([31,81,187], this study).

According to GlobalFungi, the identical sequences were found in 33 samples, in forest soil in Australia (29 samples) [169,188,189] and rhizosphere soil from grasslands in Africa (four samples, South Africa) [190]. The sites have temperate climate (MAT avg. 16.4 °C, MAP avg. 884 mm).

Notes: Our specimen CBS 146716 from Australia differs from the holotype described from fallen *Eucalyptus* sp. leaves in Brazil [31] in slightly longer setae and shorter conidia. The setae have sterile, subhyaline, obtusely rounded apex on material from nature, in culture the apex is modified into a mono- or polyphialide. The conidial length corresponds to the lower range given in the protologue, (14.5–)17.5–23 μm [31]. In the present phylogeny, our strain grouped with the ex-epitype strain of *S. septata* [82]; they share nearly identical ITS and *tef1-α* sequences, which differ in one base pair in each gene.

*Stilbochaeta septata* resembles *S. novae-guineensis* in septate conidia with a simple setula at each end and sterile or fertile setae, but conidia of the latter species are 0–1-septate vs. 1–2-septate and slightly wider with longer setulae. It is difficult to distinguish *S. septata* from *S. cangshanensis* [29] because the size of their setae, conidiophores and conidia overlap. The two species are shown to be closely related in the present phylogeny but are well distinguished using ITS and *tef1-α* sequences. The genetic distances between the ex-type strain of *S. cangshanensis* and ex-epitype strain of *S. septata* are 4.0% in the ITS and 3.6% in *tef1-α* genes corresponding to the sequence identities of 96.6% and 96.5%, respectively.

***Stilbochaeta submersa*** (Z.L. Luo, K.D. Hyde & H.Y. Su) Réblová & Hern.-Restr., **comb. nov.** MycoBank MB 842220.

Basionym. *Dictyochaeta submersa* Z.L. Luo, K.D. Hyde & H.Y. Su, Fungal Divers. 99: 597. 2019.

For description and illustration, see Luo et al. [29].

Habitat and geographical distribution: Saprobe on decaying wood, known only from Asia, China [29]. The database GlobalFungi did not contain similar sequences (≥98%) of this species.

Notes: Although the conidia were described as aseptate, the photographs accompanying the protologue [29] show 0–1-septate conidia, which is in accordance with the generic concept of *Stilbochaeta*. *Stilbochaeta submersa* resembles *S. aquatica* [28] in the conidial size and monophialidic conidiogenous cells, but the latter species differs in shorter conidiophores.

#### 3.5.5. The Genus Tainosphaeria

***Tainosphaeria*** F.A. Fernández & Huhndorf, Fungal Divers. 18: 44. 2005.

Notes: *Tainosphaeria* is a holomorphic genus characterized by perithecial ascomata, unitunicate asci, hyaline, transversely septate ascospores, and *Codinaea*-like anamorphs [57]. Members of the genus form effuse colonies of pigmented conidiophores terminating into a mono- or polyphialides and have hyaline, aseptate conidia with setulae. *Tainosphaeria* became heterogeneous after the acceptance of several anamorphic species with different morphology [29,30]. Réblová et al. [49] re-evaluated the generic concept of *Tainosphaeria* and showed correlation between molecular and morphological data, followed by the segregation of its species into three genera, namely *Tainosphaeria* s. str., *Phialoturbella* [49] and *Phialogeniculata* [191]. Li et al. [192] described another two *Tainosphaeria* species that do not match the concept of the genus. They are treated in this study, see *Tainosphaeriella*.

Revision of three former *Codinaea* species, namely *C. parva*, *C. simplex,* and *C. vulgaris*, described by Hughes and Kendrick [2] from decaying plant material from New Zealand revealed that they are remarkably similar to members of *Tainosphaeria*. Although their cultures or DNA data are not available, based on a detailed morphological comparison of their type material, original descriptions, illustrations and data on geographical distribution, they were transferred to *Tainosphaeria* and new combinations are proposed below. For additional information, see Section 4.5.

Several other species, such as *Dictyochaeta brachysetula* and *D. chinensis* [193], *D. coffeae* [8], *D. longispora* [2], and *D. unisetula* [194] resemble *Tainosphaeria* in characters of conidiophores, conidiogenous cells and conidia to some extent. However, their cultures or molecular data to verify such relationships are not available.

**Figure 43:**
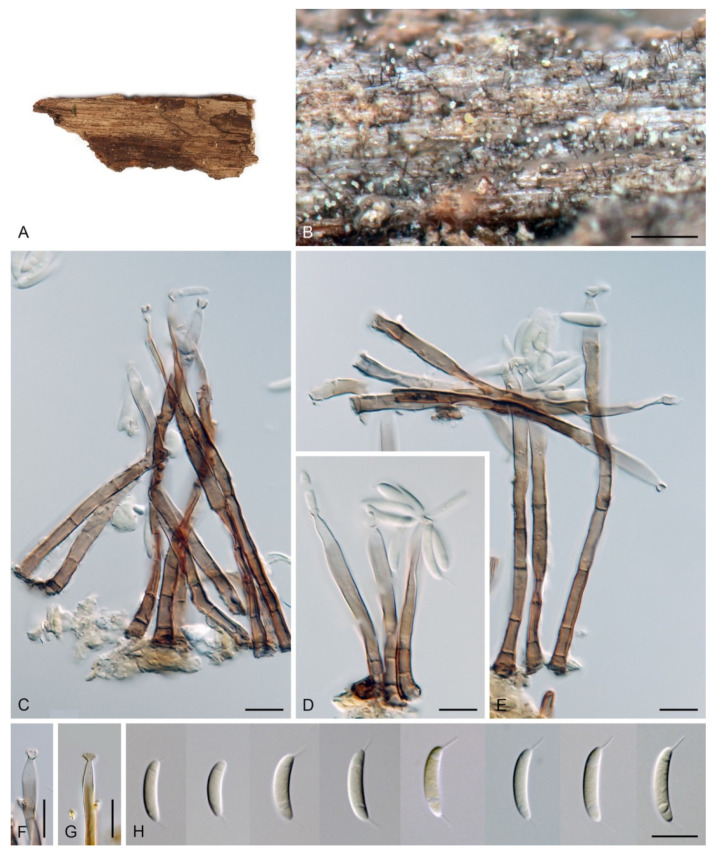
*Tainosphaeria parva* (DAOM 93565d isotype). (**A**) Substrate with colonies (**B**–**E**) conidiophores (**F**,**G**) tips of the conidiogenous cells (**H**) conidia. Images: (**A**–**H**) from nature. Scale bars: (**B**) 500 μm; (**C**–**H**) 10 μm.

***Tainosphaeria parva*** (S. Hughes & W.B. Kendr.) Réblová & Hern.-Restr., **comb. nov.** MycoBank MB 842221. (Figure 43).

Basionym. *Codinaea parva* S. Hughes & W.B. Kendr., N. Z. J. Bot. 6: 354. 1968.

≡ *Dictyochaeta parva* (S. Hughes & W.B. Kendr.) Hol.-Jech., Česká Mykol. 42: 204. 1988.

For description and illustrations, see Hughes and Kendrick [2].

Specimen examined: NEW ZEALAND, West Coast region, Westland district, Pukekura, Lake Ianthe, on decaying bark of *Weinmannia racemosa*, 8 April 1963, S.J. Hughes 569d (**isotype** of *Codinaea parva* DAOM 93565d).

Habitat and geographical distribution: *Tainosphaeria parva* is a saprobic species found mainly in the Southern Hemisphere. Originally, it was described from the bark of *Weinmannia racemosa* in New Zealand [2]. Additional specimens have been recorded on fallen leaves of *Nothofagus solandri* also from New Zealand [145] and *Pandanus hornei* and *P. tectorius* from Seychelles [44]. Holubová-Jechová [195] recorded this species on a dead petiole of *Calyptrogyne dulcis* in Cuba.

Notes: Our observations of the isotype match the protologue of this species. *Tainosphaeria parva* is characterized by mono- and polyphialidic conidiogenous cells and conidia with short appendages, which are the shortest (2–4 μm long) among other species of the genus.

**Figure 44:**
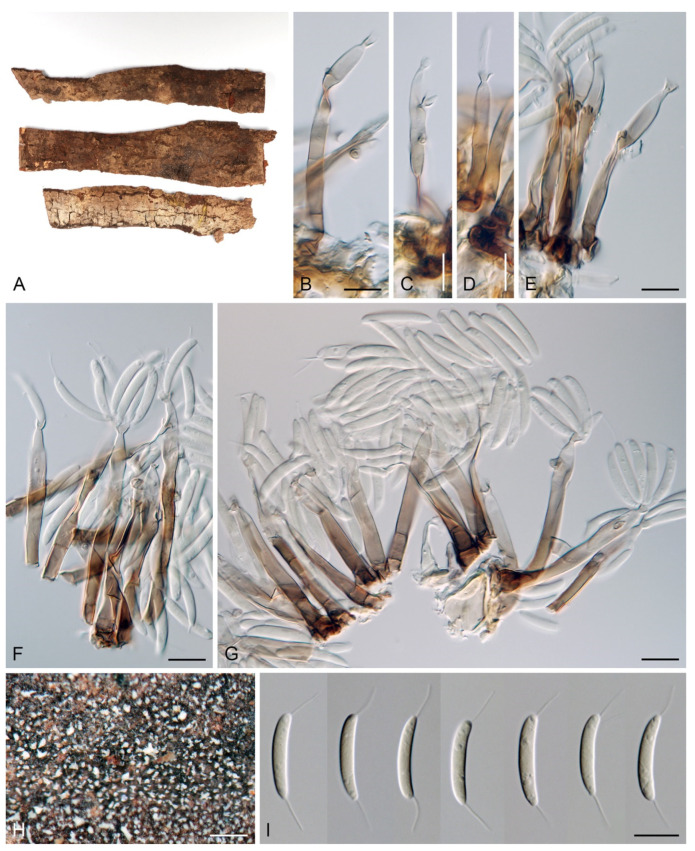
*Tainosphaeria simplex* (DAOM 96020g isotype). (**A**) Substrate with colonies (**B**–**G**) conidiophores (**H**) colony on wood (**I**) conidia. Images: (**A**–**H**) from nature. Scale bars: (**B**–**G,I**) 10 μm; (**H**) 500 μm.

***Tainosphaeria simplex*** (S. Hughes & W.B. Kendr.) Réblová & Hern.-Restr., **comb. nov.** MycoBank MB 842222. (Figure 44).

Basionym. *Codinaea simplex* S. Hughes & W.B. Kendr., N. Z. J. Bot. 6: 362. 1968.

≡ *Dictyochaeta simplex* (S. Hughes & W.B. Kendr.) Hol.-Jech., Folia geobot. phytotax. 19: 434. 1984.

For description and illustrations, see Hughes and Kendrick [2].

Specimen examined: NEW ZEALAND, West Coast region, Westland district, Pukekura, Lake Ianthe, on bark of *Weinmannia racemosa*, 8 April 1963, S.J. Hughes 560g (**isotype** of *Codinaea simplex* DAOM 96020g).

Habitat and geographical distribution: *Tainosphaeria simplex* is a saprobe, originally described from bark and wood of an evergreen shrub *Weinmannia racemosa* [2]. In addition, it occurs on fallen leaves, herbaceous stems and decaying woody fruits of *Castanopsis* sp., *Cedrela odorata*, *Didelotia idea*, *Diospyros sanza-minika*, *Dichapetalum toxicarium*, *Eucalyptus* sp., *Fagus sylvatica*, *Freycinetia banksia*, *Memecylon donianum*, *Nothofagus fusca*, *Pandanus furcatus*, *P. seychellarum*, *P. tectorius*, *Podocarpus macrophyllus*, *Q. virginiana*, *Quercus* sp., *Rubus* sp., and *Socratea* sp.

According to literature, it is one of the most common *Tainosphaeria* species and has been recorded from many localities in the temperate, subtropical and tropical geographical zones. According to Kirk [196], *T. simplex* is the most common species of the genus in British Isles based on herbarium records in the Kew Herbarium. The species is known from Australia, Brazil, China, Cuba, Germany, Japan, Ivory Coast, Malaysia, New Zealand, Papua New Guinea, Seychelles, Solomon Islands, Taiwan, and United Kingdom [2,5,17,25,31,35,36,37,44,121,134,145,146,147,148,196,197,198].

A possible misinterpretation of the species concept of *T. simplex* ([2], as *Codinaea simplex*) with *Codinaeella minuta* is discussed in notes to the latter species. *Codinaeella minuta* resembles *T. simplex*, but differs in having an upper layer of setiform conidiophores. Analysis of environmental ITS sequences from GlobalFungi supports the theory that *Tainosphaeria* and *Ca. minuta* have distinct regions of distribution; *Tainosphaeria* is primarily distributed in Australasia and Southeast Asia and in Central America, while *Ca. minuta* has a much wider range of distribution in the temperate Northern Hemisphere. Given the similarity of the two species, published records on distribution of *T. simplex* should be verified using DNA data.

Notes: Our observations of the isotype are in agreement with the protologue. The conidiophores were densely aggregated on the substrate surface, but slightly shorter ca. 38–68 μm long, mostly monophialidic or with 1(–2) phialidic apertures and conidia 15–20.5 × 2.5–3 μm with setulae 6–8(–9) μm long compared to the protologue.

**Figure 45:**
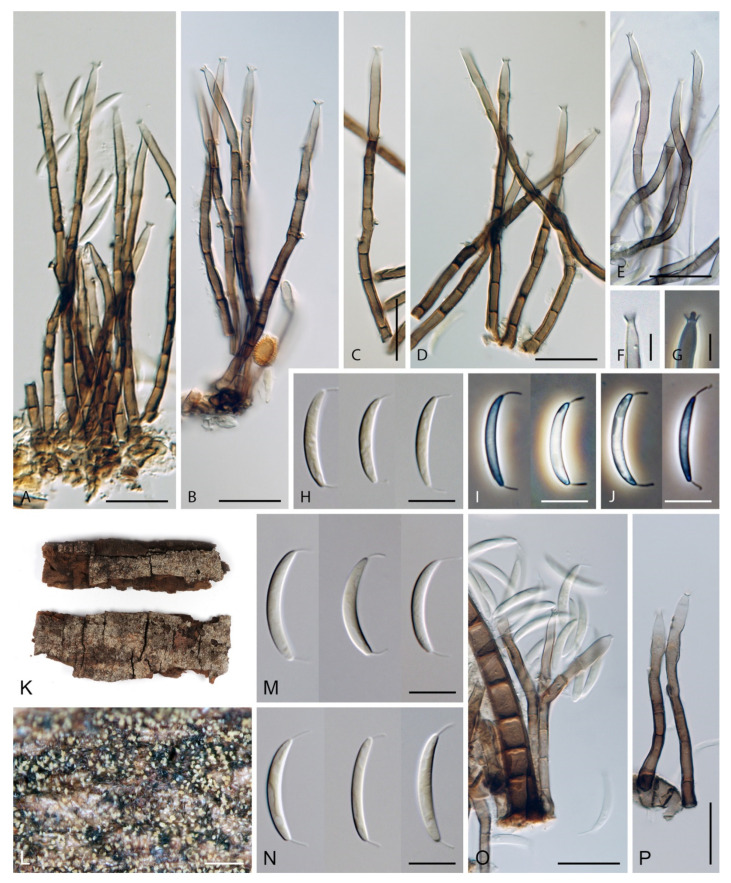
*Tainosphaeria vulgaris*. (**A**–**E**,**O**,**P**) Conidiophores (**O**) associated with conidiophores of *Helicoma* sp. (**F**,**G**) tips of the phialides (**H**–**J**,**M**,**N**) conidia (**K**) substrate with colonies (**L**) colony on wood, in detail. Images: (**A**–**D**,**H**,**I**,**K**–**P**) from nature (**E**–**G**,**J**) on PCA after 4 weeks; (**A**–**J**) from PDD 119682 (**K**–**P**) from DAOM 97315b isotype. Scale bars: (**A**–**E**,**O**,**P**) 20 μm; (**F**,**G**) 5 μm; (**H**–**J**,**M**,**N**) 10 μm.

***Tainosphaeria* *vulgaris*** (S. Hughes & W.B. Kendr.) Réblová & Hern.-Restr., **comb. nov.** MycoBank MB 842223. (Figure 45).

Basionym. *Codinaea vulgaris* S. Hughes & W.B. Kendr., N.Z. J. Bot. 6: 367. 1968.

≡ *Dictyochaeta vulgaris* (S. Hughes & W.B. Kendr.) Aramb. & Cabello, Mycotaxon 34: 682. 1989. (Nom. inval., Art. 41.4).

≡ *Dictyochaeta vulgaris* (S. Hughes & W.B. Kendr.) Whitton, McKenzie & K.D. Hyde, Fungal Divers. 4: 151. 2000.

Description on the natural substrate: Colonies effuse, olivaceous-brown to amber, composed of conidiophores, mycelium immersed. **Anamorph.** Setae absent. Conidiophores 62–120 μm long, 4–5 μm wide near the base, macronematous, crowded, arise singly or in groups, sometimes aggregate in loose columns, unbranched, erect, straight or flexuous, slightly geniculate, septate, smooth, brown, paler toward the apex. Conidiogenous cells 14.5–30 × 3–3.5 μm tapering to 1.5–2 μm below the collarette, integrated, terminal, mono- and polyphialidic with 2–15 lateral openings while internally septa can be formed, extending percurrently and sympodially, cylindrical, pale brown, subhyaline at the apex, usually bearing persistent remnants of the collarettes; collarettes 3–4 μm wide, 1.5–2 μm deep, funnel-shaped, subhyaline to pale brown, the apical part soon evanescent. Conidia (16–)18–20 × 2–2.5 μm (mean ± SD = 18.4 ± 1.0 × 2.3 ± 0.2 μm), falcate, tapering toward both ends, narrowly rounded apically, slightly truncate at the base with an inconspicuous basal scar, aseptate, hyaline, with straight or gently curved setula at each end 2.5–4.5 μm long, inserted terminally at the apex, subterminally at the base, conidia accumulate in slimy yellow-ochre fascicles. **Teleomorph.** Unknown.

Culture characteristics: Colonies on PCA effuse, mycelium composed of branched, septate, subhyaline to pale brown hyphae 1.5–2.5 μm diam. Setae absent, conidiophores, conidiogenous cells and conidia similar to those from nature. Conidiophores 90–120(–150) μm long, 3.5–4 μm wide above the base, unbranched, erect, straight, flexuous or undulating, mid brown, paler toward the apex. Conidiogenous cells 15–33 × 3–4 μm tapering to 1.5–2 μm below the collarette, integrated, terminal, monophialidic, cylindrical, pale brown, subhyaline at the apex; collarettes 3–4.5 μm wide, 2–2.5 μm deep, funnel-shaped, subhyaline. Conidia (16.5–)18–21 × 2.5(–3) μm (mean ± SD = 19.1 ± 1.5 × 2.6 ± 0.2 μm), falcate, hyaline, aseptate, setulae 2–3.5 μm long, aggregated in slimy ochre fascicles.

Specimens examined: NEW ZEALAND, Wellington province, Tongariro National Park, Silica Springs Track, Whakapapanui stream, on bark of *Fuscospora cliffortioides*, 5 March 1963, S.J. Hughes 432b (**isotype** of *Codinaea vulgaris* DAOM 97315b). NEW ZEALAND, West Coast region, Buller district, Victoria Forest Park, Lyell, Lyell Historical Walk, on decaying wood of a branch, 6 April 2005, M. Réblová M.R. 3447/NZ 731 (PDD 119682).

Habitat and geographical distribution: Saprobe on decaying bark and wood of a wide range of hosts in New Zealand, i.e., *Fuscospora cliffortioides*, *Nothofagus truncata*, *Quintinia serrata*, *Rubus* sp., *Weinmannia racemosa* and other unknown hosts ([2], this study), and leaves of *Pandanus seychellarum* in Seychelles [44].

Notes: The species was recollected in New Zealand and isolated into axenic culture, where it yielded sporulating conidiophores similar to those from nature. Although conidiogenous cells were polyphialidic with numerous successive lateral apertures on the nature substrate, conidiogenous cells remained monophialidic when grown in culture. Unfortunately, the strain is no longer viable. A description and illustration based on the recent material is given above; our observations agree with the protologue of *T. vulgaris*.

*Tainosphaeria vulgaris* is similar to *T. parva* in the geniculate appearance of conidiophores and relatively short conidial setulae up to 5 μm long. *Tainosphaeria parva* differs from *T. vulgaris* in shorter conidia. The conidia of *T. vulgaris* are arranged in ochre fascicles, while the conidial heads of other *Tainosphaeria* species were reported white.

#### 3.5.6. The Genus Tainosphaeriella

***Tainosphaeriella*** Réblová & Hern.-Restr., **gen. nov.** MycoBank MB 842003.

Type species. *Tainosphaeriella aquatica* (X.D. Yu, C.X. Li & H. Zhang) Réblová & Hern.-Restr.

Etymology: *Tainosphaeria* and -*ella* (L) diminutive but here used as a name-forming suffix, referring to fungi morphologically similar to *Tainosphaeria*.

Colonies on natural substrate effuse, pale to dark brown, mycelium partly superficial, partly immersed. **Anamorph.** Setae absent. Conidiophores macronematous, mononematous, single, erect, straight or slightly flexuous, unbranched, septate, smooth, brown, paler toward the apex. Conidiogenous cells integrated, terminal, phialidic; collarettes flared, campanulate. Conidia falcate, cylindrical to fusiform or ellipsoidal-fusiform, slightly curved, transversely septate, hyaline, occasionally laurel green at maturity, with a straight or gently curved setula at each end, conidia accumulate in slimy whitish or ochre fascicles. (Adapted after Li et al. [192]). **Teleomorph.** Unknown.

Habitat and geographical distribution: Members of *Tainosphaeriella* occur on submerged decaying wood and are only known from Southeast Asia, Thailand [192].

Notes: The genus *Tainosphaeriella* (*Ta.*) is described for two species with 1–3-septate, setulate conidia borne on monophialidic conidiogenous cells with campanulate to almost disk-like collarettes and unbranched conidiophores. Originally, they were placed in *Tainosphaeria* by Li et al. [192], but molecular data do not support such relationship. In the present phylogenetic analysis, the two species are unrelated to *Tainosphaeria crassiparies*, the type species, and other species of *Tainosphaeria*. Instead, they grouped as a sister clade to *Phialogeniculata guadalcanalensis* and *Phialoturbella* spp.

Two *Dictyochaeta* species, *D. macrospora* and *D. variabilis* [23,24] resemble *Tainosphaeriella*. Both species are native to Malaysia and are found on submerged leaves or decaying palm fronds in terrestrial environments. Unfortunately, their living cultures or DNA sequences are not available for study.

***Tainosphaeriella aquatica*** (X.D. Yu, C.X. Li & H. Zhang) Réblová & Hern.-Restr., **comb. nov.** MycoBank MB 842224.

Basionym. *Tainosphaeria aquatica* X.D. Yu, C.X. Li & H. Zhang, Phytotaxa 509: 60. 2021.

For description and illustration, see Li et al. [192].

Habitat and geographical distribution: Saprobe on decaying submerged wood, known only from Southeast Asia, Thailand [192].

Notes: *Tainosphaeria aquatica* closely resembles *D. variabilis* [24], which differs in having longer conidia.

***Tainosphaeriella thailandensis*** (W. Dong, C.X. Li & H. Zhang) Réblová & Hern.-Restr., **comb. nov.** MycoBank MB 842225.

Basionym. *Tainosphaeria thailandensis* [as *thailandense*] W. Dong, C.X. Li & H. Zhang, Phytotaxa 509: 61. 2021.

For description and illustration, see Li et al. [192].

Habitat and geographical distribution: Saprobic on decaying submerged wood, known only from Southeast Asia, Thailand [192].

Notes: This species has lightly pigmented conidia that turn laurel green at maturity, making it easily distinguishable from *Ta. aquatica* with conidia hyaline at maturity.

#### 3.5.7. The Genus Xyladelphia

***Xyladelphia*** Réblová, A.N. Mill. & Hern.-Restr**., gen. nov.** MycoBank MB 842004.

Type species. *Xyladelphia longiseta* (F.A. Fernández & Huhndorf) Réblová, A.N. Mill. & Hern.-Restr.

Etymology: *Xýlon* (Greek) wood, referring to the lignicolous habitat; *adélphia* (Greek), siblings, referring to two morphs occurring together.

Colonies on natural substrate effuse, hairy, brown, composed of setae, conidiophores and ascomata, mycelium immersed. **Anamorph.** Setae grow singly, erect, straight or flexuous, septate, brown, unbranched, always sterile, tapering to an acute apex, sometimes two most apical cells dark brown. Conidiophores macronematous, mononematous, arise singly or in groups, scattered among the setae, unbranched, erect, straight or flexuous, septate, brown, smooth. Conidiogenous cells integrated, terminal, mono- and polyphialidic, extending percurrently and sympodially; collarettes flared. Conidia of two morphologically distinct types; macroconidia falcate to fusiform, aseptate, hyaline, with straight or gently curved setula at each end; microconidia (formed only in vitro) ellipsoidal, aseptate, hyaline to pale brown, without setulae; macroconidia and microconidia accumulate in slimy whitish fascicles. **Teleomorph.** Ascomata perithecial, non-stromatic, superficial, subglobose to broadly ovoid, papillate, dark brown, setose; setae sterile, acute at the apex, identical to those of the anamorph. Ostiole periphysate. Ascomatal wall, carbonaceous, two-layered. Paraphyses septate, tapering. Asci unitunicate, cylindrical-clavate, shortly-stipitate, apically rounded, ascal apex with a non-amyloid apical annulus. Ascospores broadly fusiform to ellipsoidal, hyaline, aseptate or transversely septate, without gelatinous sheath or appendages (Adapted after Fernández and Huhndorf [57]).

Habitat and geographical distribution: Saprobes on decaying plant matter, found in the Central, North and South America.

Notes: In the present phylogeny, *Xyladelphia* was shown distantly related to *Codinaea* and other morphologically similar genera. It formed a separate lineage near the base of the tree among taxa with non-setulate conidia.

Although the arrangement of setae and conidiophores is similar to members of *Codinaeella*, the setae are darker in the uppermost part and have sterile, acute apex. *Xyladelphia* clustered as a sister to *Dictyochaeta brevis* [30], which lack setae and somewhat resembles *Tainosphaeria*. Due to inconsistencies in morphology, the latter species is not accepted in *Xyladelphia* and for now we prefer to keep it separate until more morphological data, in vitro observations, teleomorph and other representatives are available for study.

***Xyladelphia longiseta*** (F.A. Fernández & Huhndorf) Réblová, A.N. Mill. & Hern.-Restr., **comb. nov.** MycoBank MB 842226.

Basionym. *Chaetosphaeria longiseta* F.A. Fernández & Huhndorf, Fungal Divers. 18: 28. 2005.

For description and illustration, see Fernández and Huhndorf [57].

Habitat and geographical distribution: Saprobic on decaying wood of twigs and branches, known from the tropical zone of Central and South America from Costa Rica, Ecuador and Puerto Rico, and also from the subtropical zone of the USA, South Carolina [57].

Notes: *Xyladelphia longiseta* is the only member of the genus and a known representative of this conspicuous morphotype. It somewhat resembles *Codinaea setosa* [2], which differs in septate conidia without setulae. *Dictyochaeta australiensis* [7] can be compared with the present species, but differs in longer setae that are undulate in the upper part and shorter conidia with longer setulae.

## 4. Discussion

### 4.1. Phylogeny and Taxonomy of Codinaea: An Exercise in Morphology

**Figure 46:**
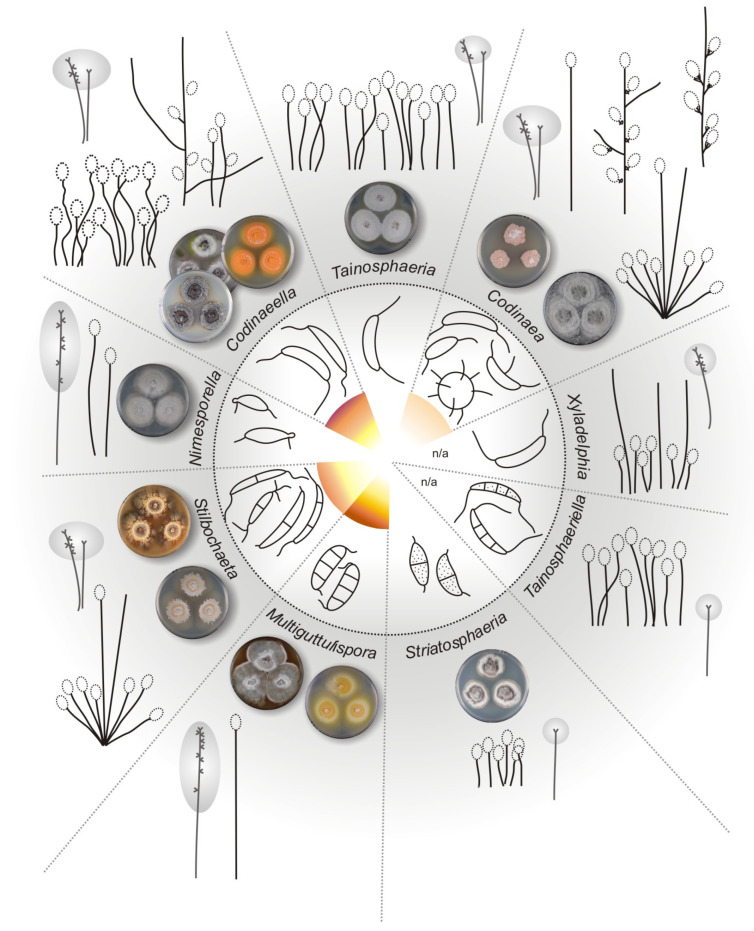
Morphotypes of *Codinaea* and similar genera with setulate conidia. Conidia and pigments formed in vitro are shown in the central (white) part of the wheel. The arrangement of conidiophores with setae, if present, of each genus, along with the morphology of the phialide (i.e., with a terminal or lateral opening) and colony characters are shown in the outer (grey) part of the wheel. Pigments diffusing in MLA and OA are shown by their respective colors and colony images; if no pigment is formed, the place at the centre is blank, n/a indicates that this information is not available.

**Figure 47:**
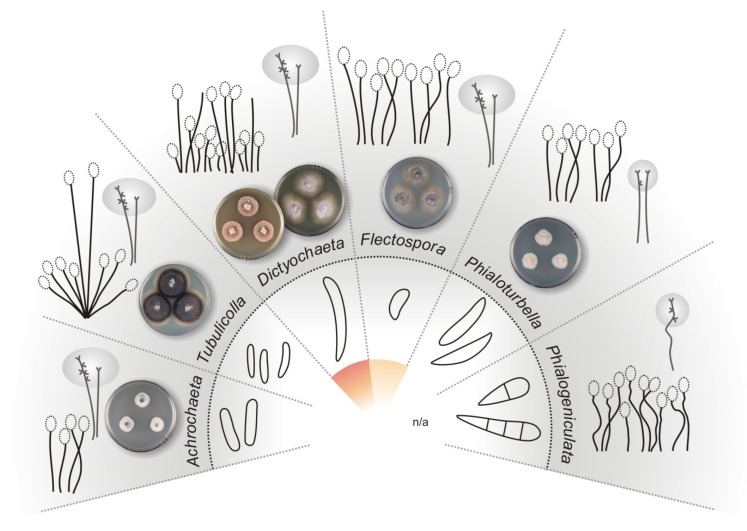
Morphotypes of *Dictyochaeta* and similar genera with non-setulate conidia. Legend as in Figure 46.

In this study, we provide evidence to resolve the intriguing complex of *Codinaea* and similar fungi. The genus *Codinaea* was re-evaluated and newly circumscribed using molecular, morphological and geographical data. Several other *Codinaea*-like, *Dictyochaeta*-like and *Tainosphaeria* species were reclassified and segregated into new genera in the Chaetosphaeriaceae, namely *Codinaeella*, *Nimesporella*, *Stilbochaeta, Tainosphaeriella* and *Xyladelphia*. The morphological separation of *Codinaea* and five other genera listed above is consistent with their phylogenetic relationships. The results of the phylogenetic analyses based on the three-locus dataset strongly support the synonymy of *Bahusutrabeeja*, *Codinaeopsis* and *Phialolunulospora* under *Codinaea*. In addition, new combinations of three other *Codinaea* species were proposed in *Tainosphaeria* based on detailed morphological and geographical comparison. Genera and their morphotypes, historically associated with *Codinaea* and *Dictyochaeta*, are illustrated in Figure 46 and Figure 47.

There are no known teleomorphs in *Codinaea*, *Codinaeella* and other segregates, and only one in *Stilbochaeta* and *Tainosphaeria*. Identification therefore depends largely on anamorphic microscopic characters, such as: (1) The presence or absence of setae, shape of the uppermost part and their arrangement with the conidiophores; (2) morphology of conidiophores, presence or absence of a sterile extension and their branching pattern; (3) shape, color and number of conidiogenous cells, position on the conidiophore, mode of elongation and morphology of the collarette; (4) septation, shape, color, and size of conidia; (5) presence or absence of setulae, their number and position on conidia; and finally (6) production of pigments in vitro.

The results of this study do not support the conclusions about the taxonomy of *Codinaea* proposed by Arambarri and Cabello [13]. Characters such as branched or unbranched conidiophores, mono- and polyphialides, percurrent or sympodial elongation of the conidiogenous cell were revealed to be uninformative at the generic level. On the other hand, some characteristics have been shown to be informative for distinguishing among genera, e.g., (1) the arrangement of setae and conidiophores, (2) morphology of the conidiogenous cell, (3) conidial septation (and to some extent also the shape), and (4) presence or absence of setulae. We found that conidial septation may be in some genera a diagnostic character, but in some genera like *Menispora*, species with both septate and aseptate conidia occur (ancestral polymorphism). Another example is the genus *Stilbochaeta*, where conidia with and without septa occur, but both types are present in every species. See also Section 3.3 and Section 4.4. on ancestral state reconstruction.

The phylogenetic analysis revealed that the evolution of some morphological traits is correlated and that these traits, which were previously used to delimit taxa at the genus rank, occur together in species newly shown to be congeneric. For example, we found that branched and unbranched conidiophores and lateral and terminal phialides can occur in species within the same genus. Of the above genera, only *Codinaea* and *Codinaeella* include species with more than one morphotype in terms of conidiophore morphology, arrangement of conidiophores and setae, and position of the phialides; and only *Codinaea* encompasses species with more than one conidial shape. On the other hand, morphology of species of the other genera is relatively uniform. Several different morphotypes and their combinations, which form a parallel to those revealed in *Codinaea* and *Codinaeella*, have evolved in genera such as *Menispora* and *Chloridium* sect. *Chloridium*, and offer an interesting comparison. In addition, these monophyletic genera reside in a strongly supported cluster (99/1.0) suggesting a shared evolutionary history. In Figure 2B, a robust clade (98/1.0) is marked, which includes most taxa with setulate conidia in the Chaetosphaeriaceae with a few exceptions, such as *Flectospora, Phialoturbella* and *Phialogeniculata*. Interestingly, taxa with setulate conidia are rare in the remaining part of the tree and are represented by *Dictyochaeta brevis* (inc. sedis) and *Xyladelphia longiseta*, which clustered near the base of the tree.

In the present phylogeny, *Menispora* (100/1.0) is represented by six species that exhibit four morphotypes (M1–M4, Figure 8) hitherto associated with the genus and distinguished based on the arrangement of setae and conidiophores and the conidiophore structure. Similar to *Codinaea*, phialides are integrated, terminal or discrete, lateral on the conidiophores, stalks or short branches. The *Menispora* morphotypes are as follows: (1) sterile acute setae are arranged in fascicles with simple, shorter conidiophores with a terminal phialide (morphotype M1: *M. britannica*), (2) the upper part of the conidiophore has a sterile, whip-like extension, while the lower part is fertile, phialides are lateral, borne singly directly on the conidiophore or stalks or they are terminal on short branches, conidiophores are sometimes accompanied by simple setae resembling conidiophores (M2: e.g., *M. caesia*, *M. ciliata*, *M. glauca*), (3) conidiophores are similar to the previous type, but setae are absent, phialides are lateral, borne in groups on densely branched stalks (M3: *M. tortuosa*), or (4) sterile setae with a whip-like sterile uppermost part are interspersed among shorter, simple conidiophores bearing a terminal phialide (M4: *M. uncinata*). Similar to *Codinaea*, conidia of *Menispora* also show a certain degree of variability, but mainly in the septation and presence or absence of setulae.

Although these four different morphotypes are widely recognized in *Menispora*, similar variability has never been considered in *Codinaea*. The *Codinaea* clade is delimited by a strongly supported node in the three-gene phylogeny and is associated with four different morphotypes (Figure 8C1–C4) that are irregularly distributed in this cluster. Molecular data suggest that the conidial shape varies within a genus. Conidia are predominantly falcate, lunate to navicular or occasionally vermiform, but sometimes also ellipsoidal, ellipsoidal-fusiform, and even globose to pyriform. In addition, the number of setulae and their position on conidia vary among species. However, color and septation seem to be constant features in the genus and conidia are always hyaline and aseptate. Species with the morphotypes C1, C3, and C4 always have falcate to lunate conidia, while the morphotype C2 is associated with a wide range of conidial shapes. The morphotypes C1 and C3 in *Codinaea* correlate with the morphotypes M1 and M3 in *Menispora*, respectively. However, while morphotype C1 in *Codinaea* is interspersed among the other three, in *Menispora* the equivalent morphotype M1 occupies a basal position and is shown as a sister to a more species-rich subclade. Although *M. britannica*, the only known representative of the M1 morphotype, was originally placed in *Codinaea* [199], later it was transferred to *Menispora* by Kirk [200].

Réblová [201] pointed out the remarkable similarity between *Codinaea* and *Menispora* and that species attributed to *Menispora* have exactly the same characteristics as *Codinaea* with the exception of the uppermost part of the phialide and the conidiophore structure. The author speculated that *Codinaea* could be included in *Menispora* as a synonym, but at the same time proposed that the relationship of *Codinaea* with *Codinaeopsis*, *Dictyochaetopsis* and *Menispora* required further investigation. The present phylogenetic analysis confirms the distinction of *Codinaea* and *Menispora* and that species, despite having similar morphotypes, should be retained in separate genera. The unifying feature of members of *Menispora* is the narrow, strongly recurved uppermost part of the conidiogenous cell ending in a short and indistinct collarette, having a beak-like appearance.

*Codinaeella* is another genus that shows similar variation in the conidiophore structure as *Menispora*. It has been associated with two morphotypes, CA1 and CA2, which are equivalent to M4 and M2 in *Menispora*, respectively (Figure 8). The characters of conidia of *Codinaeella* are uniform; species have hyaline, aseptate, falcate conidia with a single setula at each end.

*Codinaea* and *Codinaeella* are also comparable to *Chloridium*. In our phylogeny, four species and three morphotypes represent *Chloridium* sect. *Chloridium* (CH1–CH3, Figure 8). *Chloridium virescens*, the type species, is characterized by simple conidiophores with terminal monophialides interspersed among similar, but longer setiform conidiophores, which are sometimes associated in loose bundles (morphotype CH1). Morphotypes consisting of conidiophores forming a single layer are also common in *Chloridium* and they are represented by *Chl. submersum* (CH2) in the phylogeny. Although *Chloridium* historically included only species with unbranched conidiophores and terminal phialides [39], and later it was divided into three sections according to the presence of single or multiple conidiogenous loci and elongation of the conidiogenous cell [202], preliminary molecular analysis suggested that it is congeneric with *Gonytrichum* [203]. The latter genus included species with anamorphs and teleomorphs remarkably similar to those of *Chloridium*, but distinct in the presence of branched conidiophores and lateral conidiogenous cells arising on collar and nodose hyphae. In our phylogeny, *Chl. caesium* and *Chl. gonytrichii* represent the CH3 “*Gonytrichum*” morphotype. *Chloridium* and *Codinaea* (i.e., *C. gonytrichodes*) are the only genera in the family that include species with discrete phialides growing from collar and nodose hyphae. In both genera this morphological trait (C4 in *Codinaea* and CH3 in *Chloridium*) occurs together with a morphotype of unbranched solitary conidiophores without setae (C2 and CH2). The main *Chloridium* CH1 morphotype also correlates with the main CA1 morphotype of *Codinaeella* and partly also with the C1 of *Codinaea*. In these three genera we see that species with simple conidiophores and terminal phialides and species with branched conidiophores and lateral phialides occur together.

Although we have been able to collect a number of species that represent most of the known *Codinaea*, *Codinaea*-like and *Dictyochaeta*-like morphotypes, we lack information on the evolutionary relationships of species that exhibit morphotypes different from those analyzed in this study. For example, species of *Codinaea* with synnematous conidiophores [2,20,204] are missing in the current phylogeny. A unique morphotype of unknown systematic placement is represented by *Codinaea eucalypti* [31], which was described from leaves of *Eucalyptus saligna* from Brazil. The conidiogenous cells have flared to tubular collarette and are consistently intercalary along the entire length of the conidiophore with a single phialidic aperture per cell. The conidiophores are septate but the distance between the septa is significantly shorter than in the other known *Codinaea* or *Codinaea*-like species. Unfortunately, no ex-type or authentic strain or living cultures are available. Hernández-Restrepo et al. [81] introduced the genus *Xyladictyochaeta* in the Xylariales, which is similar to *C. eucalypti*, but differs in phialides with inconspicuous collarettes and denticles-like openings with multiple conidiogenous loci.

**Figure 48:**
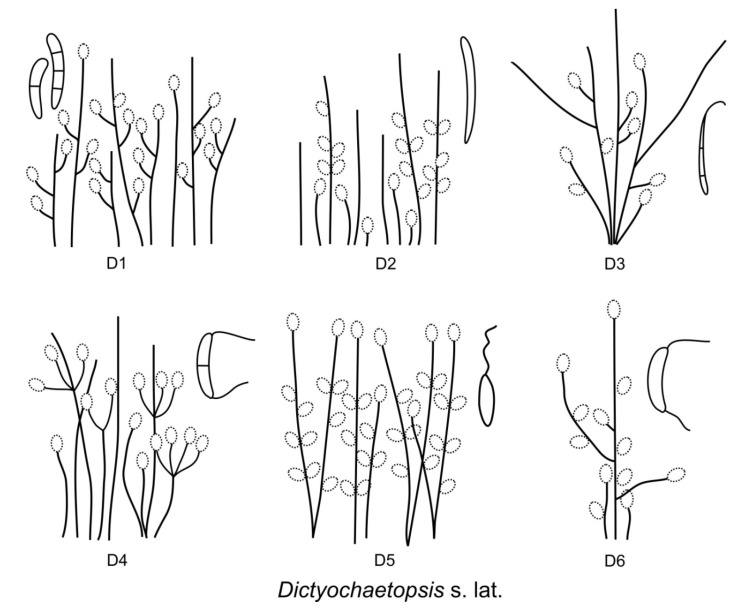
Morphotypes D1–D6 associated with *Dictyochaetopsis* s. lat. Morphotypes D1: *Di. apicalis*, *Di. glauconigra* (=*Dictyochaetopsis* s. str.), D2: *Di. antillana*, *Di. maharashtrensis*, D3: *Di. hamata*, D4: *Di. dingleyae*, D5: and D6: *Di. elegantissima*, *Di. intermedia* and *Di. menisporoides*.

In the past, newly discovered morphological character(s) or their combinations often led to a proliferation of a new genus distinguished from *Codinaea* and *Dictyochaeta*, such as *Codinaeopsis*, *Dictyochaetopsis*, and *Menisporella*. Moreover, *Dictyochaetopsis* has become a heterogeneous collection of species, whose phylogenetic affiliations are largely unknown. *Codinaeella filamentosa* (formerly *Dictyochaetopsis filamentosa*) is the only representative of *Dictyochaetopsis* in the present phylogenetic analysis. However, the species is only the tip of the iceberg. Approximately six morphotypes can be recognized in *Dictyochaetopsis*, however, relationships of species with these morphotypes need to be verified using DNA sequences. These morphotypes (D1–D6) are illustrated in Figure 48.

They include: *Dictyochaetopsis* s. str. (morphotype D1: *Di. apicalis*, *Di. glauconigra*) [132] and other five *Dictyochaetopsis*-like morphotypes (D2: *Di. antillana*, *Di. maharashtrensis*) [205,206], (D3: *Di. hamata*) [21], (D4: *Di. dingleyae*) [2], (D5: *Di. brasiliensis*) [207] and (D6: *Di. elegantissima*, *Di. intermedia* and *Di. menisporoides*) [26,32]. The three species of the D6 morphotype match the concept of *Ca. filamentosa* (morphotype CA2) and are discussed in the notes to the latter species. Relationships of other *Codinaea* and *Dictyochaeta* species lacking ex-type strains or living cultures and molecular data and that resemble the genera *Achrochaeta*, *Codinaea* s. str., *Dictyochaeta* s. str., *Phialoturbella*, *Tainosphaeria, Tainosphaeriella* and *Tubulicolla*, are discussed in this study in the notes to respective genera and also Réblová et al. [12,49].

### 4.2. Impact of Morphological Data Quality and Their Availability on Taxonomy

Taxonomy of *Codinaea* and similar fungi has been controversial and traditionally it has been based on microscopic morphology of structures observed on natural substrates and to a lesser extent in vitro, but rarely have both types of observations been included for comparison and evaluation. With the increasing use and availability of molecular data, new species have been described as either *Codinaea* or *Dictyochaeta*, but surprisingly without comparisons to previously described species. When sequences from new material do not match any of those in public repositories, new names were introduced but older and valid names were rarely considered. In several cases, the new taxa were based solely on in vitro features, which, as we have shown above, can be problematic when used alone. We therefore focused on evaluating both sets of traits, verifying strains obtained from culture collections, designating epitypes when available, and generating reference sequences, in particular ITS and *tef1-α* barcode sequences, to populate the genetic repositories enabling rapid and accurate species identification.

We faced several obstacles during this study. In general, working with non-type strains available in the culture collections poses a risk because they may be misidentified. A proverb “trust but verify” applies here more than ever. The revealed phylogenetic placement of several non-type strains of *Codinaea* and *Dictyochaeta* indicated that they are misidentified, which are now corrected here to avoid future erroneous conclusions. Their identification was challenging. Sometimes no herbarium material was deposited along with the strain and some strains were isolated from soil or plant tissues, thus only microscopic characters in vitro were available. Therefore, we grew strains on sterile stems of *U. dioica* on CMA and sterile pine needles on SNA. These media proved to be the best option to provide semi-natural conditions and induce sporulation and formation of conidiophores and setae. On other media used in this study, such as CMD, MLA, OA and PCA, fungi sporulated but often formed only conidiophores without setae. One example is *Codinaea acaciae* [53], where prolonged incubation was required for the development of all morphological structures. The species was isolated from leaf spots of *Acacia mangium*, but only simple conidiophores were described in the protologue. Its placement in the *Codinaea* clade, which contains species that commonly form bundles of setae and conidiophores under natural conditions, has raised the suspicion that there may be other features that we have not yet observed. Growth of this strain on CMA with *Urtica* stems for >8 weeks revealed a typical *Codinaea* C1 morphotype.

To sum up, cultivation and verification of several strains of *Codinaea* and *Dictyochaeta* allowed for their correct identification: CBS 139907 [53] ex-type strain of *C. acaciae* is *C. assamica*, ICMP 14613 and ICMP 15540 deposited as *C. fertilis* are *Ca. lutea*; MUCL 41171 [129] as *Codinaeopsis gonytrichodes* is *C. amazonensis*; CBS 194.96 as *C. vulgaris* is *C. siamensis*; MUCL 34876 as *D. fertilis* is *C. paniculata* [46]; MUCL 28054 as *D. simplex* is *Ca. parvilobata*; CBS 115959 as *D. parva* is *Ca. minuta*, IMI 233824 as *D. parva* is *C. fertilis*, and IMI 358908 as *D. daphnioides* is *N. capillacea*. In addition, strain GZCC 18-0017 [30] identified as *C. simplex* is *Ca. lutea*.

The identity of the ex-type strain CBS 114070 of *Dictyochaeta curvispora* was a bit of a mystery because it clustered with *Stilbochaeta* species, which have entirely different morphology. According to the protologue [181], *D. curvispora* has never sporulated in vitro and formed only sterile mycelium that diffused a yellow pigment into the agar. The yellow, gold, honey to pale apricot pigments were also produced on nutrient media used in this study. To reveal its identity, the species was inoculated on sterile *Urtica* stems on CMA, where it sporulated for the first time. We confirm that the ex-type strain of *D. curvispora* is a contamination and that the fungus deposited in its place is *Stilbochaeta aquatica* (Figure 33).

Revision of type and other herbarium material without the relevant DNA sequences and living cultures may be only of some benefit and may not yield the desired result; especially in groups such as *Codinaea*, *Tainosphaeria* and their allies, where the dimensions of conidia, conidiophores and setae often overlap. Although we proposed three new combinations in the absence of molecular data, our conclusions were supported by detailed morphological comparisons and geographical data. Revision of the holotypes of three former species of *Codinaea*, such as *C. parva*, *C. simplex* and *C. vulgaris*, indicated that they belong to *Tainosphaeria*. However, the proposed combinations have yet to be verified using DNA data.

The study of *Codinaea* and related fungi combining data from nature and culture is quite challenging. The diagnostic morphological traits develop best under natural conditions and do not appear to be significantly affected by host or substrate type. To reliably identify *Codinaea* and similar fungi, we therefore recommend obtaining microscopic characteristics from both natural substrate and culture, as in vitro observations alone may not be sufficient. Knowledge of the wild type is also necessary to identify older species with valid names whose sequences are not available. Endophytic strains or those isolated from soil, air, and other substrates should be inoculated on sterilized plant pieces placed on nutrient agar media to provide the best semi-nature conditions for development of diagnostic microscopic structures. The formation of pigments in culture is usually a supplementary character. The pigments were abundantly formed especially on MLA and OA in *Codinaeella* and *Stilbochaeta* and former *Codinaea* species now classified in *Multiguttulispora* [49]. It has been reported that the pigment production may be affected by cultivation techniques [208]. We observed a correlation between the volume of medium and intensity of the pigment diffused into the agar, and a reduction of aerial mycelium and also reverse color in *Ca. minuta* (Figure 27F–H and Figure 28) and *Ca. lambertiae* (not shown). Our observations are consistent with Raper and Fennell [208], who reported that the volume of media had an effect on colony diameter and also on reverse color, sporulation, production of exudates, and formation of sclerotia in *Penicillium*. For some other groups of hyphomycetous fungi, especially ubiquitous or medically important, in vivo morphology is not as essential and instead the emphasis is on techniques aimed at standardizing cultivation parameters to make taxonomic work reproducible, as is notoriously the case with *Penicillium* and *Aspergillus* e.g., [209].

### 4.3. CBC Species Concept in Codinaea, Codinaeella and Stilbochaeta

The species concepts of *Codinaea* and two of its segregates, *Codinaeella* and *Stilbochaeta*, were corroborated by morphological, molecular, and geographical data. In addition, the interspecific relationships were studied using the CBC species concept. The in-depth analysis of the ITS2 molecule was performed on these three genera because they are species-rich and have available DNA data.

The CBC species concept is based on the identification of canonical pairs that are subject to mutual substitution in helices H1−H3 of the ITS2 molecule [104,105,107,108]. The CBC substitutions can serve as molecular signatures and can be used to delimit biological species. At the RNA structural level, species are further characterized by hCBCs and non-CBCs, which evolve rapidly and occur more frequently than CBCs. However, members of these three genera, with one exception, do not form sexual stages under natural conditions and sexual mating has never been observed under laboratory conditions. Therefore, the CBC criterion was assessed independently of sexual reproduction [108].

The CBC analysis corroborated only one species in *Codinaea* (although 13 species were distinguished based on morphological and molecular data), one in *Codinaeella* (8 species), and two in *Stilbochaeta* (8 species). The lack of CBCs and a greater number of detected hCBCs and some non-CBCs substitutions among their members may indicate that the speciation process has not been completed. Caisová et al. [210] suggested that the fast evolving hCBCs and short-lived non-CBCs substitutions may enable faster ecological adaptations of organisms followed by changes in morphology.

In *Codinaea*, the CBC occurred only in *C. phasma*, other hCBCs and non-CBCs substitutions were detected in *C. amazonensis*, *C. gonytrichodes,* and *C. phasma*. These species, which have the major changes in their primary and secondary structure, clustered near the base of the *Codinaea* clade, and represent three distinct morphotypes, C3, C4, and C1, respectively. A similar situation was observed in *Codinaeella*. The only CBC occurred in *C. parvilobata*. With the exception of *Ca. pini*, *Ca. parvilobata* has the longest branch of all species, corresponding to the greatest number of genetic changes. However, hCBCs were detected in all species; they were particularly common in *Ca. lambertiae*, *Ca. mimusopis,* and *Ca. pini*. In *Stilbochaeta*, we identified two CBCs for *S. brevisetula* in H2 and one CBC for *S. aquatica* in H3. Both species also had the most hCBCs of all *Stilbochaeta* species. *Stilbochaeta brevisetula* has the longest branch compared to other species in the genus and is shown as a sister to a subclade containing other members of the genus. In addition, two non-CBCs events occurred in the area identified as an asymmetrical loop in H3. It contained nine unpaired nucleotides in *S. septata* and seven in *S. cangshanensis*, whereas in other species the loop was smaller and consisted of only five nucleotides.

### 4.4. Ancestral State Reconstruction

Our evolutionary and ancestral state reconstructions showed that the distribution of morphological characters corresponds largely with the phylogenetic clustering. The reconstructions of the ancestral state typically showed the presence of all states in the ancestral nodes and subsequent evolution toward the lineages with a rather homogenous set of a single character. The exception is the genus *Menispora*, which still retains the ancestral polymorphism, with high morphological variability between closely related species. This also shows that morphological characters defined here are stable and are taxonomically informative in most genera.

We showed that the studied fungi evolved in the temperate regions of Eurasia and Americas and that presence in the tropics and on other continents is secondary. Needless to say, our analyses may also be affected by the sampling bias (fewer samples from the tropics and Africa) and by the fact that many geographic records have not been validated with molecular data.

### 4.5. Global Biogeography of Codinaea and Related Fungi

In this study, we compared geographic range and ecology known from literature and field records with those retrieved from metabarcoding data in GlobalFungi. Most literature and field data originate from plant debris, while dataming showed that 64% of all samples were obtained from the bulk soil in forests and others from the non-forest sites. Soil samples represent 43% of all samples in GlobalFungi. Therefore, we assume that the relatively high affinity of the studied fungi for soil is not an artefact of sampling; rather these fungi should also be considered soil dwellers, where they live in the form of mycelia, conidia, or ascospores (chlamydospores have never been observed in culture or on natural substrate).

Concerning geographical distribution, we can conclude that GlobalFungi and the previously known distribution patterns correspond in the case of ten species, such as *C. amazonensis*, *C. assamica*, *C. fertilis*, *C. gonytrichodes*, *C. lignicola*, *C. phasma*, *C. terminalis*, *Ca. minuta*, *Ca. yunnanensis,* and *S. septata*. In five species, the area of distribution was confirmed and further expanded within the same climatic zone, i.e., *C. paniculata*, *Ca. lutea*, *Ca. parvilobata*, *S. cangshanensis*, *S. malaysiana*. Two species, *S. novae-guineensis* and *S. ramulosetula*, were not found in the same geographical areas, but were nevertheless identified in the same climatic zone. For the other species, GlobalFungi data confirmed the already known area of distribution and further expanded it in terms of geography and climate (*C. pandanicola*, *C. siamensis*). The only notable exception from the generally good fit was *Ca. pini*, which was so far reported from the tropical Uganda only, but GlobalFungi shows it in temperate zone of North America. In the case of *C. gonytrichodes* and *Ca. filamentosa*, GlobalFungi results correspond with the geographical origin of the studied strains and published records that were not verified by DNA data, and show their distribution also in other continents and climatic zones.

Assigning three species of *Codinaea*, such as *C. parva*, *C. simplex,* and *C. vulgaris* from New Zealand, to *Tainosphaeria* in the absence of molecular data was challenging. Their transfer, based on the study of type material and morphological characters, was supported by biogeographic data. In addition, it was revealed that the interpretation of *C. simplex* varied among the authors and that the fungus could be misidentified as *Codinaeella minuta* with the main area of distribution in Holarctic realm in Europe and North America and partially in Asia in the Near East and Japan. According to GlobalFungi, members of *Tainosphaeria* are widely distributed in Australasia and Southeast Asia. *Tainosphaeria jonesii* and related species are abundant in New Zealand and Southeast Asia, which is consistent with the published records of *T. jonesii* and similar fungi [29,30,47]. Based on known data [49,57], the second centre of distribution is in Central America, where *T. crassiparies*, the type species, and *T. cecropiae* occur. This is also in agreement with the origin of *T. parva*, *T. simplex* and *T. vulgaris*, whereas the geographical distribution of *Ca. minuta* does not extend into Australia and New Zealand.

In the BLASTn search, GlobalFungi used the complete sequence similarity rule, which may limit the observed distribution in the case of more genetically variable species. Thus, study of additional haplotypes of the particular species can increase its observed range accordingly. Other option is to use more relaxed similarity threshold (e.g., 99.4%) to cover broader intraspecies variability. However, such step increases the risk of joining more biological species together. Using our rigorous approach, we found that the geographical distribution of the fungi studied primarily follows the climatic zones. This pattern is typical of filamentous, saprobic fungi [117,211].

## 5. Conclusions

The genus *Codinaea* has become a large heterogeneous group of phialidic dematiaceous hyphomycetes with hyaline, setulate conidia, and for more than 30 years was considered synonymous with the genus *Dictyochaeta*. The taxonomy of these fungi has relied mainly on morphological criteria. Little was known about their systematics and global biogeography. The morphotypes that have been associated with different species of *Codinaea* over time have never been investigated using molecular data. We studied morphological, molecular, and biogeographic data of a large set of species, including all available ex-type strains. The present study shows that *Codinaea* is a highly polyphyletic taxon unrelated to *Dictyochaeta* and that its original delimitation based on a single morphotype of *C. aristata* is too narrow and unsustainable. In fact, three additional morphotypes were assigned to the strongly supported monophyletic clade of *Codinaea*. Phylogenetic analyses and ancestral state reconstruction showed that the development of some morphological traits, previously considered significant at the generic level, seem to be evolutionarily correlated and can occur is species of a single genus. In this context, the morphotypes associated with *Codinaea*, *Chloridium,* and *Menispora* share similar variability and were compared. In the phylogenetic analysis of three nuclear markers (ITS, 28S and *tef1-α*), several former *Codinaea* and *Dictyochaeta* species clustered in five unknown lineages and were introduced as new genera, i.e., *Codinaeella*, *Nimesporella, Stilbochaeta*, *Tainosphaeriella,* and *Xyladelphia*. In addition, we designated two epitypes (*S. brevisetula* and *S. ramulosetula*) and a lectotype (*C. aristata*), and we also proposed four new species and 28 new combinations. For all ex-type and validated non-type strains the reference ITS and *tef1-α* barcode sequences were generated to allow for fast and accurate identification. The studied genera comprise fungi commonly occurring on decaying plant matter, such as decaying wood, bark, wooden fruits (often buried in soil), stems, leaf litter, bamboo and palm fronds, some of them exhibit endophytic lifestyle, occasionally they were isolated from soil or occur as plant pathogens.

Our research highlights the importance of studying the combination of microscopic morphological characters developed in culture and under natural conditions for identification. Some diagnostic characteristics, especially setae, and their arrangement with conidiophores, occur mainly on the natural substrate. However, when grown in culture, these features may not develop or their formation is delayed by weeks or months. Nutrient media such as CMA with sterile stems of *U. dioica* and SNA with sterile pine needles proved useful in obtaining morphological characteristics similar to those in nature. To improve our knowledge of the genus *Codinaea* and similar taxa we would like to support general efforts to obtain living culture and molecular data of these interesting and morphologically variable fungi. In the long-term, we hope to initiate studies that will result in a rational phylogeny-based classification of *Codinaea* and find answers regarding the value of morphological characters traditionally used to delimit hyphomycetous genera.

This study, together with Réblová et al. [86], demonstrates that GlobalFungi shows very good agreement with known reports on geographic distribution and provides additional valuable information. In particular, datamining of ITS barcodes from cultivation independent studies can fundamentally increase our knowledge about the diversity, biogeography, and ecology of studied taxa. Exceptions to the low agreement between the two approaches can be explained by gaps in sampling, i.e., understudied areas of South America and Africa and limited knowledge of intraspecies variation. The newly used approach can be especially helpful in the study of inconspicuous and rare fungi. The processing of metabarcoding data has expanded the known distribution, identified new biodiversity hotspots, and added new information on species ecology. In particular, environmental sequence data confirm that members of *Codinaea*, *Codinaeella*, and *Stilbochaeta* live also as pure soil fungi, even in the cropland soil. Their distribution pattern is restricted to Holarctic realm including Nearctic and Palearctic, tropical climate zones including Neotropics and Paleotropics and some species also occur in South Africa, Southeast Asia and Australasia. Interestingly, their distribution never reaches boreal climatic zone. The reconstruction of the ancestral geographical distribution showed Eurasia or Eurasia and Americas as ancestral, whereas the presence in African and Australasian areas as derived. In our study, we optimized the GlobalFungi datamining workflow, which includes (1) identifying of the barcoding gap for each spacer, (2) BLASTn searching with the full-length coverage requirement, and (3) checking for potential artefacts due to the misidentified ITS1 and ITS2 spacer ends in the GlobalFungi database (see Section 2.7.).

## Figures and Tables

**Table 1:** Taxa, isolate information, and GenBank accession numbers for sequences. New sequences determined for this study and taxonomic novelties are given in bold face.

Taxon ^1^	Strain ^2^	Country	Host	Substrate ^3^	GenBank Accession Numbers		
					ITS	28S	*tef1-a*
*Achrochaeta talbotii*	ICMP 15161	New Zealand	unknown	W	MT454480	MT454495	**OL653988**
*Arcuatospora novae-zelandiae*	CBS 109474	Venezuela	*Nectandra* sp.	L	MW984569	MW984552	**OL653989**
*Arcuatospora seorsa*	CBS 147510 T	Thailand	broad leaf tree	L	MW984572	MW984555	**OL653990**
*Arcuatospora* sp.	CBS 694.74	Japan	unidentified	L	MW984573	MW984556	**OL653991**
*Arcuatospora* sp.	MUCL 43189	Nepal	unknown	U	MW984574	MW984557	**OL653992**
*Catenularia minor* ^a^	PRM 900544 T	Thailand	unidentified	Ba	MW987827	MW987822	**OL653993**
*Chaetosphaeria hebetiseta*	CBS 102340 T	Ukraine	*Fagus sylvatica*	W	AF178549	AF178549	**OL653994**
*Chalarodes obpyramidata*	PDD 119364	New Zealand	*Nothofagus* sp.	W	MW987828	MW987823	**OL653995**
*Codinaea amazonensis*	MUCL 41171	Brazil	unidentified	L	**OL654076**	**OL654133**	**OL653996**
*Codinaea assamica* ^b^	CBS 139907T	Malaysia	*Acacia mangium*	LS	**OL654077**	**OL654134**	**OL653997**
*Codinaea dwaya*	CBS 261.77 T	India	*Coffea arabica*	W/B	**OL654078**	**OL654135**	**OL653998**
*Codinaea fertilis*	CBS 242.66	Guadeloupe	*Musa* sp.	R	**OL654079**	**OL654136**	**OL653999**
*Codinaea fertilis*	IMI 233824	New Zealand	*Betula* sp.	R	**OL654080**	**OL654137**	**OL654000**
*Codinaea gonytrichodes*	CBS 593.93	Japan	unidentified	P	AF178556	AF178556	**OL654001**
*Codinaea paniculata*	CBS 145098 T	France	deciduous tree	SW	MT118230	MT118201	**OL654002**
*Codinaea paniculata*	CBS 126573	France	*Alnus glutinosa*	SW	MT118231	MT118202	**OL654003**
*Codinaea paniculata*	CBS 127692	France	*Fraxinus* sp.	SW	MT118232	MT118203	**OL654004**
*Codinaea paniculata*	MUCL 34876	United Kingdom	unidentified	SL	MT118233	MT118204	**OL654005**
*Codinaea phasma*	CBS 147516 T	Puerto Rico	unidentified	W	**OL654081**	**OL654138**	**OL654006**
*Codinaea siamensis*	CBS 194.96	Papua New Guinea	n/a	S	**OL654082**	**OL654139**	**OL654007**
*Codinaeella filamentosa*	CBS 147265	USA	*Quercus* sp.	L	**OL654083**	**OL654140**	**OL654008**
*Codinaeella lambertiae*	CBS 143419 T	Australia	*Lambertia formosa*	L	**OL654084**	**OL654141**	**OL654009**
*Codinaeella lutea*	CBS 146618 T	Czech Republic	*Quercus* sp.	C	**OL654085**	**OL654142**	**OL654010**
*Codinaeella lutea*	CBS 624.77	The Netherlands	*Quercus robur*	C	**OL654086**	**OL654143**	**OL654011**
*Codinaeella lutea*	ICMP 14613	New Zealand	*Quercus ilex*	L	**OL654087**	**OL654144**	**OL654012**
*Codinaeella lutea*	ICMP 15540	New Zealand	*Quercus* sp.	C	**OL654088**	**OL654145**	**OL654013**
*Codinaeella mimusopis*	CBS 143435 T	South Africa	*Mimusops caffra*	L	MH107888	MH107935	**OL654014**
*Codinaeella minuta*	ATCC 20960	USA	hardwood tree	L	**OL654089**	**OL654146**	**OL654015**
*Codinaeella minuta*	CBS 298.61 T	Japan	*Lithocarpus edulis*	L	**OL654090**	**OL654147**	**OL654016**
*Codinaeella minuta*	CBS 115959	Italy	*Quercus suber*	W	**OL654091**	**OL654148**	**OL654017**
*Codinaeella minuta*	CBS 145099	France	*Quercus* sp.	A	**OL654092**	**OL654149**	**OL654018**
*Codinaeella minuta*	CBS 145100	France	*Quercus* sp.	A	**OL654093**	**OL654150**	**OL654019**
*Codinaeella minuta*	CBS 146619	Czech Republic	*Quercus* sp.	A	**OL654094**	**OL654151**	**OL654020**
*Codinaeella minuta*	CBS 146620	Czech Republic	*Quercus* sp.	A	**OL654095**	**OL654152**	**OL654021**
*Codinaeella minuta*	CBS 146621	Czech Republic	*Quercus* sp.	A	**OL654096**	**OL654153**	**OL654022**
*Codinaeella minuta*	CBS 147518	The Netherlands	*Quercus* sp.	L	**OL654097**	**OL654154**	**OL654023**
*Codinaeella minuta*	CBS 966.69	The Netherlands	*Quercus* sp.	A	AF178559	AF178559	**OL654024**
*Codinaeella minuta*	DAOM 148141	USA	*Quercus virginiana*	L	**OL654098**	**OL654155**	**OL654025**
*Codinaeella minuta*	TTI-0830	USA	*Quercus virginiana*	L	**OL654099**	**OL654156**	**OL654026**
*Codinaeella parvilobata*	CBS 144536 T	Czech Republic	*Fagus sylvatica*	C	**OL654100**	**OL654157**	**OL654027**
*Codinaeella parvilobata*	CBS 144658	Czech Republic	*Fagus sylvatica*	Se	**OL654101**	**OL654158**	**OL654028**
*Codinaeella parvilobata*	CBS 144792	Czech Republic	*Quercus* sp.	C	**OL654102**	**OL654159**	**OL654029**
*Codinaeella parvilobata*	MUCL 28054	Belgium	unidentified	L	**OL654103**	**OL654160**	**OL654030**
*Codinaeella pini*	CBS 138866 T	Uganda	*Pinus patula*	N	**OL654104**	**OL654161**	**OL654031**
*Dendrophoma cytisporoides*	CBS 144107	Germany	*Buxus sempervivens*	B	MT118234	MT118205	**OL654032**
*Ellisembia folliculata*	CBS 147152	Czech Republic	*Carpinus betulus*	W	**OL654105**	**OL654162**	**OL654033**
*Flectospora laminata*	CBS 112964T	Thailand	unidentified	W	MW984576	MW984558	**OL654034**
*Flectospora* sp.	ICMP 23840	New Zealand	unidentified	W	MW984577	MW984559	**OL654035**
*Kionochaeta ramifera*	MUCL 39164	Cuba	unidentified	L	MW144421	MW144404	**OL654036**
*Menispora britannica*	PRA-20985	Czech Republic	*Fagus sylvatica*	C	**OL654106**	**OL654163**	**OL654037**
*Menispora caesia*	CBS 144659	Czech Republic	*Quercus* sp.	W	MW984578	MW984560	**OL654038**
*Menispora caesia*	CBS 145022	Czech Republic	*Carpinus betulus*	W	**OL654107**	**OL654164**	**OL654039**
*Menispora ciliata* ^c^	CBS 122131 T	Czech Republic	*Acer campestre*	W	EU488736	**OL654165**	**OL654040**
*Menispora ciliata*	CBS 145024	Czech Republic	*Quercus* sp.	A	**OL654108**	**OL654166**	**OL654041**
*Menispora glauca*	CBS 144543	Czech Republic	unidentified	W	**OL654109**	**OL654167**	**OL654042**
*Menispora tortuosa*	CBS 117552	Czech Republic	*Fraxinus angustifolia*	B	**OL654110**	**OL654168**	**OL654043**
*Menispora tortuosa*	CBS 117553	Canada	*Acer* sp.	B	**OL654111**	**OL654169**	**OL654044**
*Menispora uncinata*	ICMP 15140	New Zealand	unidentified	W	**OL654112**	**OL654170**	**OL654045**
*Menispora uncinata*	ICMP 18253	New Zealand	unidentified	W	**OL654113**	GU180637	**OL654046**
*Menisporopsis pirozynskii*	MUCL 47217	Congo	unidentified	L	MW984579	MW984561	**OL654047**
*Menisporopsis theobromae*	MUCL 41079	Venezuela	unidentified	L	MW984580	MW984562	**OL654048**
*Multiguttulispora dimorpha*	CBS 140002	Malaysia	*Eucalyptus* sp.	W/B	MW984582	MW984564	**OL654049**
*Multiguttulispora triseptata*	IMI 353690	Cuba	unidentified	L	MW984584	MW984566	**OL654050**
*Nimesporella capillacea*	IMI 358908 T	Ivory Coast	unidentified	L	**OL654114**	**OL654171**	**OL654051**
*Phialoturbella calva*	ICMP 23826 T	New Zealand	unidentified	B	MW984585	MW984567	**OL654052**
*Stilbochaeta aquatica* ^d^	CBS 114070 T	Philippines	bamboo	BaS	**OL654115**	**OL654172**	**OL654053**
*Stilbochaeta brevisetula*	ICMP 15125	New Zealand	*Nothofagus* sp.	W	**OL654116**	**OL654173**	**OL654054**
*Stilbochaeta brevisetula*	ICMP 22548	New Zealand	deciduous tree	W	**OL654117**	**OL654174**	**OL654055**
*Stilbochaeta brevisetula*	ICMP 22549 E	New Zealand	*Nothofagus* sp.	B	**OL654118**	**OL654175**	**OL654056**
*Stilbochaeta brevisetula*	ICMP 22551	New Zealand	deciduous tree	B	**OL654119**	**OL654176**	**OL654057**
*Stilbochaeta brevisetula*	ICMP 22552	New Zealand	deciduous tree	B	**OL654120**	**OL654177**	**OL654058**
*Stilbochaeta malaysiana*	IMI 312436 T	Malaysia	unidentified	L	**OL654121**	**OL654178**	**OL654059**
*Stilbochaeta* *novae-guineensis*	CBS 147515	Puerto Rico	unidentified	W	**OL654122**	**OL654179**	**OL654060**
*Stilbochaeta* *novae-guineensis*	CBS 147517	Puerto Rico	unidentified	W	**OL654123**	**OL654180**	**OL654061**
*Stilbochaeta ramulosetula*	IMI 313452 E	Malaysia	unidentified	L	**OL654124**	**OL654181**	**OL654062**
*Stilbochaeta septata*	CBS 146716	Australia	*Melaleuca viminalis*	L	**OL654125**	**OL654182**	**OL654063**
*Tainosphaeria cecropiae*	CBS 101687 T	Puerto Rico	*Cecropia* sp.	Pe	MW984586	MW984568	**OL654064**
*Thozetella cristata*	CBS 101112	Venezuela	unidentified	L	**OL654126**	**OL654183**	**OL654065**
*Thozetella effusa*	CBS 115044	Hong Kong	*Phoenix hanceana*	U	**OL654127**	**OL654184**	**OL654066**
*Thozetella tocklaiensis*	CBS 378.58 T	India	*Camellia sinensis*	F	**OL654128**	**OL654185**	**OL654067**
*Tracylla aristata*	CBS 141404 E	Australia	*Eucalyptus regnans*	L	**OL654129**	**OL654186**	**OL654068**
*Tracylla eucalypti*	CBS 144429 T	Colombia	*Eucalyptus urophylla*	L	**OL654130**	**OL654187**	**OL654069**
*Xyladelphia longiseta*	S.M.H. 1725	Puerto Rico	unidentified	W	**OL654131**	AF279416	**—**
*Xyladelphia longiseta*	S.M.H. 3854	Puerto Rico	unidentified	W	**OL654132**	AF279417	**—**

Notes: ^1 a^ holotype of *Chaetosphaeria trianguloconidia*, ^b^ ex-type strain of *Codinaea acaciae*, ^c^ ex-type strain of *Chaetosphaeria ciliata*, ^d^ ex-type strain of *Dictyochaeta curvispora*; ^2^ T, E denote ex-type and ex-epitype strains; ^3^ A: acorn, B: bark, Ba: bamboo culm, BaS: submerged bamboo culm, C: cupule, F: flower, L: leaves/leaf litter, LS: leaf spot, N: needles, P: decaying plant material, Pe: petiole, R: root, S: soil, Se: seed, SL: submerged leaf, SW: submerged wood, U: unknown, W: wood, W/B: wood and bark.

**Table 2:** Key to *Codinaea* and Similar Genera with Mononematous Conidiophores and Setulate Conidia.

1	The uppermost part of the phialide tapering, strongly recurved, ending in a short and indistinct collarette, having a beak-like appearance	*Menispora*
1	The uppermost part of the phialide straight, usually with a flared collarette	2
2	Phialides with an indistinct collarette and denticles-like conidiogenous loci, almost exclusively intercalary along the entire conidiophore length, with a single aperture to each cell, the apical cell of the conidiophore with a terminal aperture	*Xyladictyochaeta*
2	Phialides with a distinct funnel-shaped to campanulate or slightly tubular collarettes, phialidic apertures terminal or lateral, sometimes becoming intercalary after sympodial elongation of the phialide and formation of internal septa	3
3	Conidia septate	4
3	Conidia without septa	9
4	Setae present, rounded to slightly inflated at the apex, sterile, occasionally fertile with one to several phialidic openings; arranged in fascicles with conidiophores	*Stilbochaeta*
4	Setae absent	5
5	Conidia hyaline	*Multiguttulispora*
5	Conidia pigmented or hyaline	6
6	Conidia versicolor, middle cells brown, end cells hyaline	7
6	Conidia uniformly pigmented, sometimes hyaline	8
7	Conidia tetrahedral or obpyramidal stauroconidia	*Phaeonawawia*
7	Conidia ellipsoidal-fusiform	*Anacacumisporium*
8	Conidia falcate, teleomorph unknown	*Tainosphaeriella*
8	Conidia ellipsoidal-fusiform, reniform to botuliform, teleomorph has dark brown ascospores with longitudinal ridges and furrows	*Striatosphaeria*
9	Phialides discrete, lateral on repent hyphae, associated with the base of the setae, setae have a dark, acute apex	*Obeliospora*
9	Phialides integrated, terminal or discrete, lateral on conidiophores	10
10	Conidia with angular outline	11
10	Conidia with round outline	12
11	Conidia adhere in basipetal chains	*Chalarodes*
11	Conidia adhere in slimy heads	*Nawawia*
12	Setae present	13
12	Setae absent	15
13	Setae always sterile, acute at the apex, apex darker than the rest of the seta, interspersed among conidiophores	*Xyladelphia*
13	Setae sometimes sterile, but usually terminating in one to several phialidic openings, paler toward the tip	14
14	Setae sterile or fertile, in fascicles with conidiophores	*Codinaea* (morphotype C1)
14	Setae always fertile, interspersed among conidiophores	*Codinaeella* (morphotype CA1)
15	Conidiophores unbranched, phialides terminal	16
15	Conidiophores branched, phialides terminal and lateral	18
16	Conidia ellipsoidal, basally papillate, basal setula positioned ventrally	*Nimesporella*
16	Conidia of a different shape	17
17	Conidia globose, obpyriform, ellipsoidal, ellipsoidal-fusiform, lunate to vermiform, conidiophores usually 170–290 µm long	*Codinaea* (morphotype C2)
17	Conidia falcate, conidiophores shorter, usually 33–130 µm long	*Tainosphaeria*
18	Lateral phialides sessile, directly on conidiophores, branches or stalks	*Codinaeella* (morphotype CA2)
18	Lateral phialides on branched unilateral stalks or nodose hyphae	19
19	Phialides on stalks that are densely branched, apex of the conidiophore sterile, setiform	*Codinaea* (morphotype C3)
19	Phialides on nodose and collar-like hyphae, apex of the conidiophore terminating into a phialide	*Codinaea* (morphotype C4)

## Data Availability

All sequences generated in this study were submitted to GenBank (ITS: OL654076–OL654132; 28S: OL654133–OL654187; *tef1-α*: OL653988–OL654069).

## Supplementary Materials

The following are available online at https://www.mdpi.com/article/10.3390/jof7121097/s1, Figure S1: Ancestral state reconstruction of conidial shape calculated by Multistate function in Bayestraits implemented is Rasp v.4.2, Figure S2: Detailed maps of the geographical distribution of *Codinaea*, *Codinaeella* and *Stilbochaeta* species based on the GlobalFungi database, Figure S3: Colony morphology of *Codinaeella minuta* after four weeks (country of origin of each strain is indicated), Figure S4: Colony morphology of *Codinaeella minuta* after four weeks (country of origin of each strain is indicated), Table S1: Taxa, isolate information and accession numbers for sequences retrieved from GenBank, Table S2: Estimates of evolutionary divergence between ITS rDNA and *tef1-α* sequences of members of *Codinaea*, *Codinaeella,* and *Stilbochaeta*, Table S3: The biogeography, substrate and habitat affinity of *Codinaea*, *Codinaeella,* and *Stilbochaeta* inferred from the GlobalFungi database, Table S4: A synopsis table of accepted species of *Codinaea* based on observations from nature and culture, Table S5: A synopsis table of accepted species of *Codinaeella* based on observations from nature and culture, Table S6: A synopsis table of accepted species of *Stilbochaeta* based on observations from nature and culture.
