# Supplementary material for: Phylogenetic Reassessment, Taxonomy, and Biogeography of Codinaea and Similar Fungi
Source: J Fungi (Basel). 2021 Dec 20;7(12):1097. doi: 10.3390/jof7121097 (PMC8704094; doi:10.3390/jof7121097)
Supplement: Supplementary file 1 [file jof-07-01097-s001.zip › Supplementary Table S1.pdf]

Table S1. Taxa, isolate information and accession numbers for sequences retrieved from GenBank. Taxonomic novelties are given in bold.

Taxon[1]	Strain	Status[2]	Country	Host	Substrate	GenBank accessions		
						ITS	28S	***tef1-α***
Adautomilanezia caesalpiniae	CC-LAMIC 102/12	T	Brazil	*Caesalpina echinata*	wood	KX821777	KU170671	—
Anacacumisporium appendiculatum	HMAS 245593	T	China	broad-leaved tree	dead stems	KP347129	KT001553	—
Anacacumisporium appendiculatum	HMAS 245602		China	broad-leaved tree	dead stems	KT001556	KT001554	—
Brunneodinemasporium brasiliense	CBS 112007	T	Brazil	unidentified	decaying leaf	JQ889272	JQ889288	—
Brunneodinemasporium jonesii	GZCC 16-0050	T	China	unidentified	decaying wood	KY026058	KY026055	—
Cacumisporium capitulatum	FMR 11339		Spain	unidentified	decaying wood	HF677176	HF677190	—
Calvolachnella guaviyunis	CBS 134695	T	Uruguay	*Myrcianthes pungens*	bark	KJ834524	KJ834525	—
Catenularia cubensis	S.M.H. 3258		Costa Rica	unidentified	decaying wood	MW987826	AF466067	—
Catenularia catenulata	DLUCC 0891	T	China	unidentified	submerged wood	MK828637	MK835838	—
Chaetosphaeria fusiformis	CBS 101429		Czech Republic	*Abies alba*	decaying bark	AF178554	AF178554	—
Chaetosphaeria innumera	M.R. 1175		Czech Republic	*Fagus sylvatica*	decaying wood	AF178551	AF178551	—
Chaetosphaeria lignomollis	S.M.H. 3015	T	Puerto Rico	unknown	decaying wood	EU037896	AF466073	—
Chaetosphaeria myriocarpa	CBS 264.76	N	The Netherlands	unidentified	decaying wood	AF178552	AF178552	—
Chaetosphaeria pygmaea	M.R. 1365		Czech Republic	*Fagus sylvatica*	decaying wood	AF178545	AF178545	—
Chloridium caesium	CBS 102339		Austria	*Salix cinerea*	decaying wood	AF178564	AF178564	—
Chloridium virescens	CBS 152.53		France	*Acer* sp.	unknown	MH857142	MH868678	—
Chloridium gonytrichii	CBS 195.60		South Africa	unidentified	unknown	MH857954	MH869503	—
Chloridium submersum	MFLUCC 16-1344	T	China	unidentified	submerged wood	MN860551	MN860556	—
Codinaea ellipsoidea	MFLUCC 18-1574	T	China	unidentified	submerged wood	MK828628	MK835828	—
Codinaea ellipsoidea	DLU 0104		China	unidentified	submerged wood	MK828626	MK835826	—
Codinaea ellipsoidea	KUMCC 15-0279		China	unidentified	submerged wood	MK828627	MK835827	—
Codinaea ellipsoidea	DLU 229		China	unidentified	submerged wood	MK828629	MK835829	—
Codinaea lignicola	DLUCC 0899	T	China	unidentified	submerged wood	MK828630	MK835830	MN194081
Codinaea pandanicola	KUMCC 16-0153	T	China	*Pandanus* sp.	dead leaf	MH388338	MH376710	MH388373
Codinaea siamensis	MFLUCC 15-0614	T	Thailand	unidentified	submerged twig	KX609955	KX609952	—
Codinaea siamensis	MFLUCC 16-0371		Thailand	*Pandanus* sp.	dead leaf	MH388339	MH376711	MH388374
Codinaea terminalis	GZCC 18-0085	T	China	unidentified	deacying leaves	MN104613	MN104624	—
Codinaea vermispora	CGMCC 3.19632	T	China	unidentified	submerged leaves	MH758194	MH758207	—
Codinaeella lutea	GZCC 18-0017		China	unidentified	decaying leaves	MN104609	MN104620	—
Codinaeella yunnanensis	MFLUCC 17-0468	T	China	unidentified	submerged wood	MK828623	MK835823	MN194076
Conicomyces pseudotransvaalensis	HHUF 29956	T	Japan	*Machilus japonica*	dead twig	LC001710	LC001708	—
Cryptophiale hamulata	MFLUCC 18-0098	E	Thailand	unknown	decaying leaf	—	MG386756	—
Cryptophiale udagawae	GZCC 18-0047		China	unidentified	decaying wood	MN104608	MN104619	—
Cryptophialoidea fasciculata	MFLU 18-1499		Thailand	unknown	submerged wood	MH758195	MH758208	—
Dictyochaeta brevis	GZCC 18-0096	T	China	unidentified	decaying wood	MN104614	MN104625	—
Dictyochaeta callimorpha	ICMP 15155		New Zealand	unidentified	decaying wood	MT454484	MT454499	MT454674
Dictyochaeta fuegiana	ICMP 15153	T	New Zealand	unidentified	decaying wood	MT454487	EF063574	MT454677
Dictyochaeta querna	CBS 145503		Czech Republic	*Quercus cerris*	acorn	MT454489	MT454503	MT454679
Dictyochaeta montana	CBS 145342	E	Czech Republic	*Fagus sylvatica*	decaying cupule	MT454488	MT454502	MT454678
Dictyochaeta detriticola	ICMP 14948	T	New Zealand	*Metrosideros* sp.	fallen leaves	MT454486	MT454501	MT454676
Dinemasporium cruciferum	HHUF 30001		Japan	*Arundo donax*	unknown	AB900895	AB934039	AB934089
Dinemasporium decipiens	CBS 592.73		Suriname	*Elaeis guineensis*	soil under	JQ889275	JQ889291	—
Dinemasporium pseudoindicum	CBS 127402	T	USA	tallgrass prairie	soil under	JQ889277	JQ889293	—
Ellisembia aurea	CBS 144403	T	France	*Sambucus nigra*	decaying wood	MH836375	MH836376	—
Eucalyptostroma eucalypti	CBS 142074	T	Malaysia	*Eucalyptus pellita*	leaf spots	KY173408	KY173500	—
Exserticlava vasiformis	TAMA 450		Japan	unidentified	plant debris	—	AB753846	—
Fuscocatenula submersa	MFLUCC 18-1342	T	China	unidentified	submerged wood	MK828634	MK835835	MN194085
Infundibulomyces cupulatus	BCC 11929	T	Thailand	*Lagerstroemia* sp.	dead leaf	EF113976	EF113979	—
Infundibulomyces oblongisporus	BCC 13400	T	Thailand	unidentified, angiosperm	leaf litter	EF113977	EF113980	—
Kionochaeta castaneae	GZCC 18-0025	T	China	*Castanea mollissima*	decaying seed she	MN104610	MN104621	—
Kionochaeta microspora	GZCC 18-0036	T	China	unidentified	decaying wood	MN104607	MN104618	—
Menisporopsis anisospora	CBS 109475	T	Venezuela	*Wettinia praemorsa*	decaying petiole	MH862827	MH874421	—
Menisporopsis breviseta	GZCC 18-0071	T	China	unknown	decaying leaves	MN104612	MN104623	—
Menisporopsis dushanensis	GZCC 18-0084	T	China	unidentified	decaying leaves	MN104615	MN104626	—
Nawawia filiformis	MFLUCC 17-2394		Thailand	unidentified	decaying wood	MH758196	MH758209	—
Neopseudolachnella acutispora	MAFF 244358	T	Japan	*Pleioblastus chino*	dead twigs	AB934065	AB934041	AB934091
Neopseudolachnella magnispora	MAFF 244359	T	Japan	*Sasa kurilensis*	dead twigs	AB934066	AB934042	AB934092
Paliphora intermedia	CBS 896.97	I	Australia	unidentified	leaf litter	MH862682	EF204501	—
Paragaeumannomyces garethjonesii	MFLUCC 15-1012	T	Thailand	Fabaceae	seed pod	KY212751	KY212759	—
Paragaeumannomyces sabinianus	ILLS121384	T	USA	unidentified	decaying wood	MT118243	MT118225	—
Paragaeumannomyces panamensis	S.M.H. 3596	T	Panama	unidentified	decaying wood	AY906948	MT118218	—
Paragaeumannomyces rubicundus	S.M.H. 3221	T	Costa Rica	unidentified	decaying wood	MT118242	MT118224	—
Paragaeumannomyces raciborskii	S.M.H. 3119		Puerto Rico	unidentified	decaying wood	AY906953	AY436402	—
Phaeostalagmus cyclosporus	CBS 663.70		The Netherlands	*Quercus* sp.	decaying bark	MH859892	—	—
Phialosporostilbe scutiformis	MFLUCC 17-0227	T	China	unidentified	submerged wood	MH758194	MH758207	—
Phialogeniculata guadalcanalensis [e]	MFLUCC 18-0260	T	Thailand	unidentified	decaying wood	MK828625	MK835825	MN194078
Phialoturbella aseptata	GZCC 18-0044	T	China	unidentified	decaying wood	MN104611	MN104622	—
Phialoturbella lunata	MFLUCC 18-0642	T	China	unidentified	submerged wood	MK828624	MK835824	MN194077
Polynema podocarpi	CBS 144415	T	New Zealand	*Podocarpus totara*	unknown	MH327797	MH327833	—
Pseudodinemasporium fabiforme	CBS 140010	T	Malaysia	*Acacia mangium*	leaf spots	KR611889	KR611906	—
Pseudolachnea fraxini	CBS 113701, HHUF 30116	T	Sweden	*Fraxinus excelsior*	unknown	JQ889287	JQ889301	AB934096
Pseudolachnea hispidula	MAFF 244365		Japan	*Morus bombycis*	dead twig	AB934072	AB934048	AB934098
Pseudolachnella asymmetrica	MAFF 244366		Japan	*Phyllostachys nigra* var. *henonis*	dead twig	AB934073	AB934049	AB934099
Pseudolachnella scolecospora	MAFF 244379		Japan	*Sasa* sp.	dead twigs	AB934086	AB934062	AB934112
Pyrigemmula aurantiaca	CBS 126743	T	Hungary	*Vitis vinifera*	bark	HM241692	HM241692	—
Rattania aquatica	MFLUCC 21-0006	T	Thailand	unidentified	submerged wood	MW260331	MW287235	—
Rattania setulifera	GUFCC 15-501	T	India	*Calamus thwaitesii*	leaves	GU191794	HM171322	—
Sporoschisma hemipsilum	MUCL 56487		Martinique	unidentified	wood	MW987829	MW987824	—
Sporoschisma longicatenatum	MFLUCC 16-0180	T	Thailand	unidentified	submerged wood	KX505871	KX358077	—
Sporoschisma mirabile	CBS 144794		France	*Alnus glutinosa*	submerged wood	MW987830	MW987825	—
Stilbochaeta aquatica	MFLUCC 15-0983	T	Thailand	unidentified	submerged wood	MH476572	MH476569	—
Stilbochaeta cangshanensis	MFLUCC 17-2214	T	China	unidentified	submerged wood	MK828632	MK835832	MN194083
Stilbochaeta septata	CBS 143386	E	Chile	*Eucalyptus grandis*	leaves	MH107889	MH107936	MH108015
Stilbochaeta submersa	DLUCC 1014	T	China	unidentified	submerged wood	MK828631	MK835831	MN194082
Striatosphaeria castanea	CBS 145352	T	French Guinea	woody liana	decaying periderm	MT118244	MT118229	—
Striatosphaeria codinaeophora	M.R. 1230		Puerto Rico	*Dacryodes excelsa*	decaying wood	AF178546	AF178546	—
Tainosphaeria crassiparies	S.M.H. 1934	T	Puerto Rico	*Hymenaea* sp.	seed pod	—	AF466089	—
Tainosphaeria jonesii	GZCC 16-0065	P	China	unidentified	submerged wood	KY026060	KY026057	—
Tainosphaeria jonesii	GZCC 16-0053		China	unidentified	decaying wood	KY026059	KY026056	—
Tainosphaeria siamensis	MFLUCC 15-0607	T	Thailand	unidentified	submerged wood	KX609956	KX609953	—
Tainosphaeriella aquatica	MFLUCC 17-2370	T	Thailand	unidentified	submerged wood	MZ161197	MZ161195	MZ170694
Tainosphaeriella thailandense	MFLUCC 18-1282	T	Thailand	unidentified	submerged wood	MZ161196	MZ161198	MZ170695
Verhulstia trisororum	CBS 143234	T	The Netherlands	n/a	soil	MG022160	MG022181	—
Zanclospora iberica	CBS 130426	T	Spain	unidentified	decaying wood	KY853480	KY853544	MW147327
Zanclospora novae-zelandiae	ICMP 15781	E	New Zealand	*Fuscospora cliffortioides*	decaying wood	MW144429	MW144411	MW147330
Zanclospora ramifera	ICMP 22738	T	New Zealand	unidentified	decaying wood	MW144433	MW144415	MW147334

Notes: [1] [e] holotype of *Tainosphaeria obclavata* ; [2] T, E, I, N and P denote ex-type, ex-epitype, ex-isotype, ex-neotype and ex-paratype strains.
