# Supplementary material for: Phylogenetic Reassessment, Taxonomy, and Biogeography of Codinaea and Similar Fungi
Source: J Fungi (Basel). 2021 Dec 20;7(12):1097. doi: 10.3390/jof7121097 (PMC8704094; doi:10.3390/jof7121097)
Supplement: Supplementary file 1 [file jof-07-01097-s001.zip › Supplementary Table S4.pdf]

Table S4. A synopsis table of accepted species of *Codinaea* based on observations from nature and culture.

**Species**	**Setae**	**Conidiophores**		**Phialides**	**Conidia**			**Reference**[1]
	**Size (μm)**	**Size (μm)**	**Structure**	**Position**	**Size (μm)**	**Setulae**	**Shape**	
C. amazonensis	absent	150–250 × (3–)4.5–6	simple	lateral on stalks	9–17 × 1.5.–2.5	5–10	lunate	[128]
	absent	260–320 × 4.5–6.5	simple	lateral on stalks	9–11.5× 1.5–2(–2.5)	5.5–9	falcate	This study.*
C. aristata	250 and longer	up to 100	simple	terminal	12–14 × 2	4–6 apical, 1 basal	falcate	[1]
C. assamica	up to 400 × up to 7.8	up to 140 × 4.5	simple	terminal	14.6–16.8 × 2.6–2.8	9.6–12.8	falcate	[2]
	273–360 × 5–7	42–125 × 3.5–4.5(–5)	simple	terminal	14–18 × 2.5–3.5	(5.5–)7–13.5	falcate	This study.*
C. dwaya	absent	170–290 × 5.5–9	simple	terminal	15–16 × 15–16	numerous	globose to pyriform	[120]
	absent	(33–)74–254 × 5–10(–11.5)	simple	terminal	15–16 × 15–16	numerous	globose to pyriform	This study.*
C. ellipsoidea	absent	254–288 × 8–10	simple	terminal	13–15 × 5.5–6.5	n/a	ellipsoidal	[29]
C. fertilis	up to 310 × up to 5.6	up to 112 × up to 4.8	simple	terminal	9–15.4 × 2–3	5–10	falcate	[2]
	152–340 × 4.5–6.6	66–146 × 4–5(–5.5)	simple	terminal	(10–)11–14.5 × 3.5–4.5	3.5–6.5(–9)	falcate	This study.*
C. gonytrichodes	absent	173–490 × 3.8–7	branched	lateral on collar hyphae, terminal	10–13(16.4) × 1.2–2.3	5.3–13.8	falcate	[6]
	absent	160–355 × 4–7	branched	lateral on collar hyphae, terminal	12–14.5 × 2–2.5(–3)	7.5–11.5	falcate	This study.*
C. lignicola	absent	204–276 × 7.5–8.5	simple	terminal	13–15 × 4.5–5.5		navicular to fusiform	[29]
C. pandanicola	n/a	up to 260 × n/a	simple	terminal	16–26 × 4–6	8–13.5	falcate	[48]
C. paniculata	230–290 × 6–7.5	62–127 × 3.5–4.5	simple	terminal	13.5–17(–17.5) × (2–)2.5–3.	5–8	falcate	[46]
	absent	95–150(–195) × 3.5–4.5	simple	terminal	11–15.5(–17) × 2.5–3.5	3.5–5.5(–7.5)	falcate	[46]*
C. phasma	160–380 × 5–10	35–97 × 2.5–4.5	simple	terminal	13.5–18 × 3–4	5–13	oblong to falcate	This study
	97–236 × 3.5–6	44–104 × 3.5–7	simple	terminal	12–18.5 × 4.5–8	3.5–11	irregularly ellipsoidal	This study.*
C. siamensis	165–365 × 3–6	60–100 × 2–5	simple	terminal	15.5–21 × 2.5–4	7–12	cylindrical or fusiform	[47]
	240–330 × 6.5–9	67–125(–153) × 3.5–5	simple	terminal	11.5–14.5 × 2.5–4	(4.5–)5–8.5(–10)	falcate	This study.*
C. terminalis	150–320 × 3.7–9.4	31–171 × 2.7–8.7	simple	terminal	14.7–20.7 × 2.9–4.2	4–9.5	long fusiform, curved	[30]
C. vermispora	absent	up to 150 × 3–4	simple	terminal	31–55 × 2.5–3.5	1.5–4.6	lunate to vermiform	[50]*

Note: [1]In vitro observations are marked with an asterisk (*).
