# Supplementary material for: Phylogenetic Reassessment, Taxonomy, and Biogeography of Codinaea and Similar Fungi
Source: J Fungi (Basel). 2021 Dec 20;7(12):1097. doi: 10.3390/jof7121097 (PMC8704094; doi:10.3390/jof7121097)
Supplement: Supplementary file 1 [file jof-07-01097-s001.zip › Supplementary Table S5.pdf]

Table S5. A synopsis table of accepted species of *Codinaeella* based on observations from nature and culture.

**Species**	**Setae[1]**	**Conidiophores**		**Phialides**	**Conidia**			**References[1]**
	**Size (μm)**	**Size (μm)**	**Structure**	**Position**	**Size (μm)**	**Setulae (μm)**	**Shape**	
Ca. filamentosa	absent	210–475 × 3.5–4	branched	lateral and terminal	14.5–16.5 × 2–2.7	up to 11	falcate	[32]
	absent	160–493 × 2.5–3.5	branched	lateral and terminal	13.5–17 × 2–3	5.5–8.5	falcate	This study.*
Ca. lambertiae	absent	100–200 × 3	branched	terminal	(13–)14–15(–18) × (2.5–)3	5–8	falcate	[10])*
Ca. lutea	125–250 × (3.5–)4.5–5.5	39–102 × 3.5–4.5	simple	terminal	12.5–17.5 × 2–2.5(–3)	(4.5–)5–8.5(–10)	falcate	This study.
	absent	36–83 × 2–4	simple	terminal	14.5–17 × 2–3	4–7.5	falcate	This study.*
Ca. mimusopis	n/a (present)	40–150 × 3–4	simple	terminal	(11–)16–18(–20) × 2.5–3(–3.5	(6–)7(–8)	fusoid	[82]*
Ca. minuta	123–184 × 3–4.5	32–108 × 3–4	simple	terminal	13–18 × 2.5–3.5	5.5–8.5(–10)	falcate	This study.
	absent	46–297(–450) × 3–3.5	branched	terminal	11.5–17.5(–18) × 2–3	4.5–7	falcate	This study.*
Ca. parvilobata	140–205 × 4.5–5.5	40–117 × 3–4.5(–5)	simple	terminal	10.5–13.5 × 2–2.5(–3)	6.5–9	falcate	This study.
	absent	38–84 × 1.5–3	simple	terminal	10.5–14 × 2–2.5	4–8	falcate	This study.*
Ca. pini	n/a (present)	30–100 × 2.5–4	simple	terminal	(12–)13–15 × (2–)2.5(–3)	8–9(–10)	fusoid	[8]*
Ca. yunnanensis	n/a	(62–)83–127 × 4.5–5.5	simple	terminal	15–17 × 2.5–3.5	n/a	fusiform	[29]

[1]Note: In vitro observations are marked with an asterisk (*).
