# Supplementary material for: Phylogenetic Reassessment, Taxonomy, and Biogeography of Codinaea and Similar Fungi
Source: J Fungi (Basel). 2021 Dec 20;7(12):1097. doi: 10.3390/jof7121097 (PMC8704094; doi:10.3390/jof7121097)
Supplement: Supplementary file 1 [file jof-07-01097-s001.zip › Supplementary Table S6.pdf]

Table S6. A synopsis table of accepted species of *Stilbochaeta* based on observations from nature and culture.

Species	Setae	Conidiophores		Phialides	Conidia				Reference[1]
	Size (μm)	Size (μm)	Structure	Position	Size (μm)	Setulae	Shape	Septation	
S. aquatica	120–180 × 3.3–4.2	47–68 3–4.5	simple	terminal	14–18 × 2–3	8–13	falcate	0–1-septate	[28]
	122–182 × 3.5–4.5	45–102 3–4	simple	terminal	13.5–17.5 × 2.5–3	5–8.5	falcate	0–1-septate	This study.*
S. brevisetula	up to 135 × 4–5	up to 80 3.5–4	simple	terminal	(18–)21.0–24.5(–27) × 3–3.7	1.4–2.7	curved	1(2–3)-septate	[2]
	116–132 × 4–5	49–87(–105) × 3.5–4.5	simple	terminal	(15.5–)17–22 × 3–4	1–1.5	falcate	1-septate	This study.*
S. cangshanensis	125–175 × 4.5–6.5	39–53 3.5–4.5	simple	terminal	15–18 × 2.5–3.5	n/a	fusiform, curved	1-septate	[29]
S. malaysiana	up to 200 × up to 5	up to 100 3–5	simple	terminal	24–32 × 3–4	up to 18	falcate	1-septate	[19]*
S. novae-guineensis	100–250 × 4–5	40–85 3–4	simple	terminal	13–20 × (2.2–)2.5–3.5(–5)	9–16	falcate	0–1-septate	[17]
	100–202 × 3–5	24–92 × 3–5	simple	terminal	14.5–33 × 2–3.5	3–14	falcate	0–1-septate	This study.*
S. ramulosetula	up to 300 × up to 5.5	up to 90 4–5	simple	terminal	16–22 × 3–4	up to 18	falcate	1-septate	[19]*
S. septata	up to 105 × 4–6	30–50 4–6	simple	terminal	(14.5–)17.5–23 × 2	5.5–10	falcate	1–2-septate	[31]
	245–260 × 3–5	34–37 3–4	simple	terminal	14–15.5 × 2–2.5(–3)	5.5–9	falcate	1-septate	This study.*
S. submersa	present	62–122(–152) 3–5	simple	terminal	13.5–16.5 × 2.5–3.5	n/a	fusiform, curved	0–1septate	[29]

[1]Note: In vitro observations are marked with an asterisk (*).
