# Supplementary material for: Phylogenetic Reassessment, Taxonomy, and Biogeography of Codinaea and Similar Fungi
Source: J Fungi (Basel). 2021 Dec 20;7(12):1097. doi: 10.3390/jof7121097 (PMC8704094; doi:10.3390/jof7121097)

Figure S1. Detailed maps of the geographical distribution of *Codinaea, Codinaeella* and *Stilbochaeta* species based on the GlobalFungi database.

Codinaea amazonensis

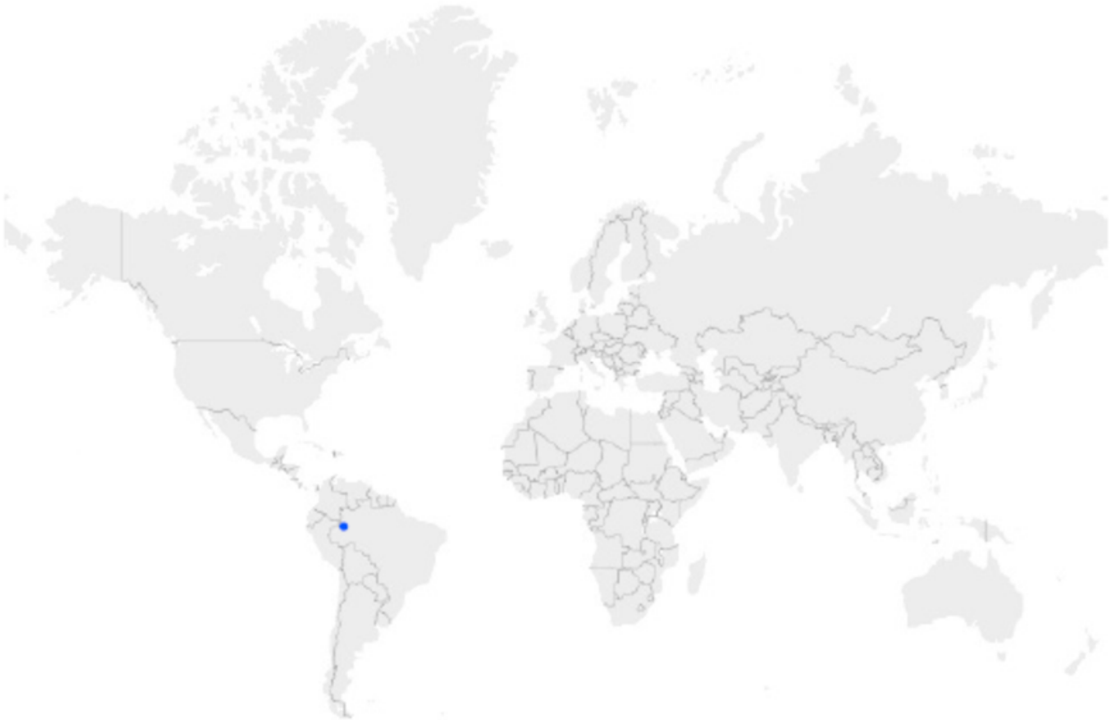

Codinaea assamica

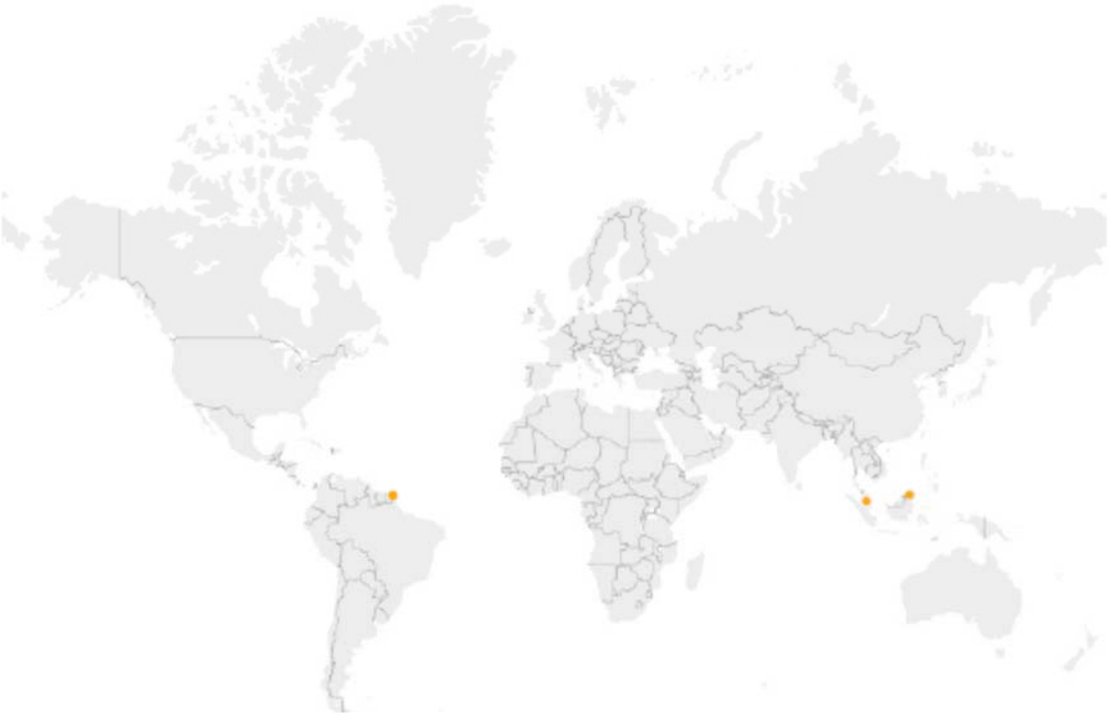

Codinaea fertilis

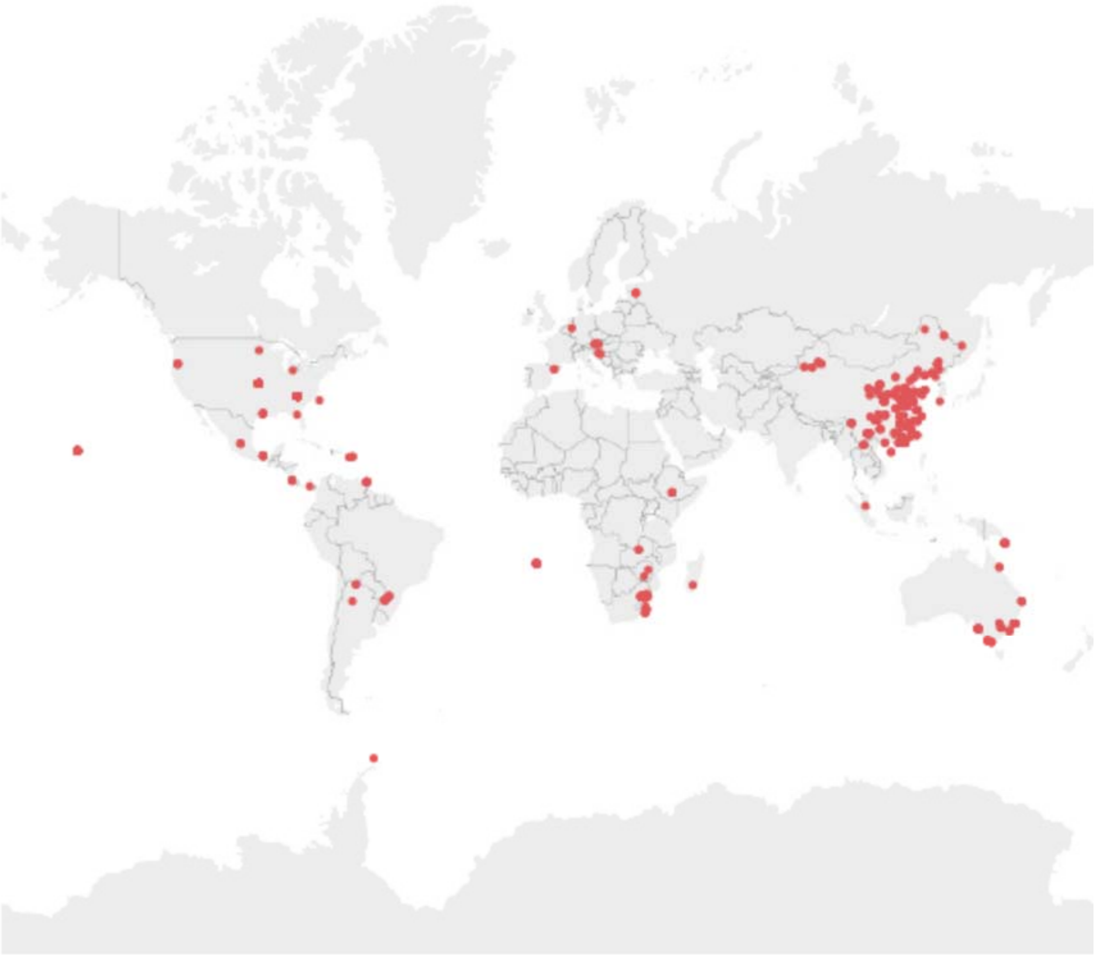

Codinaea lignicola

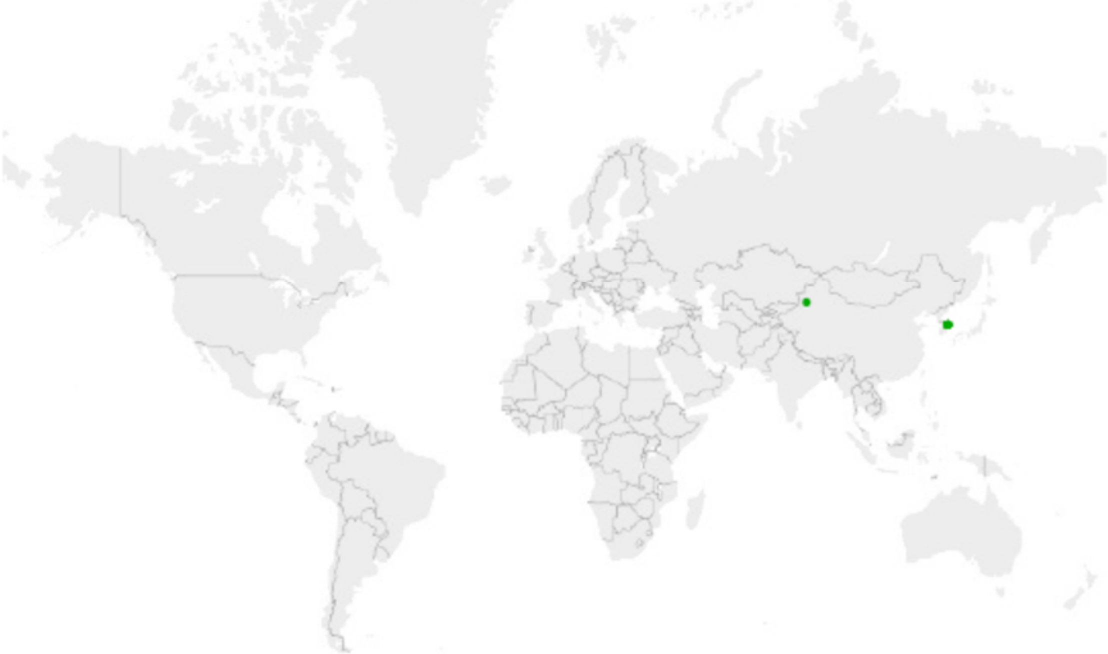

Codinaea pandanicola

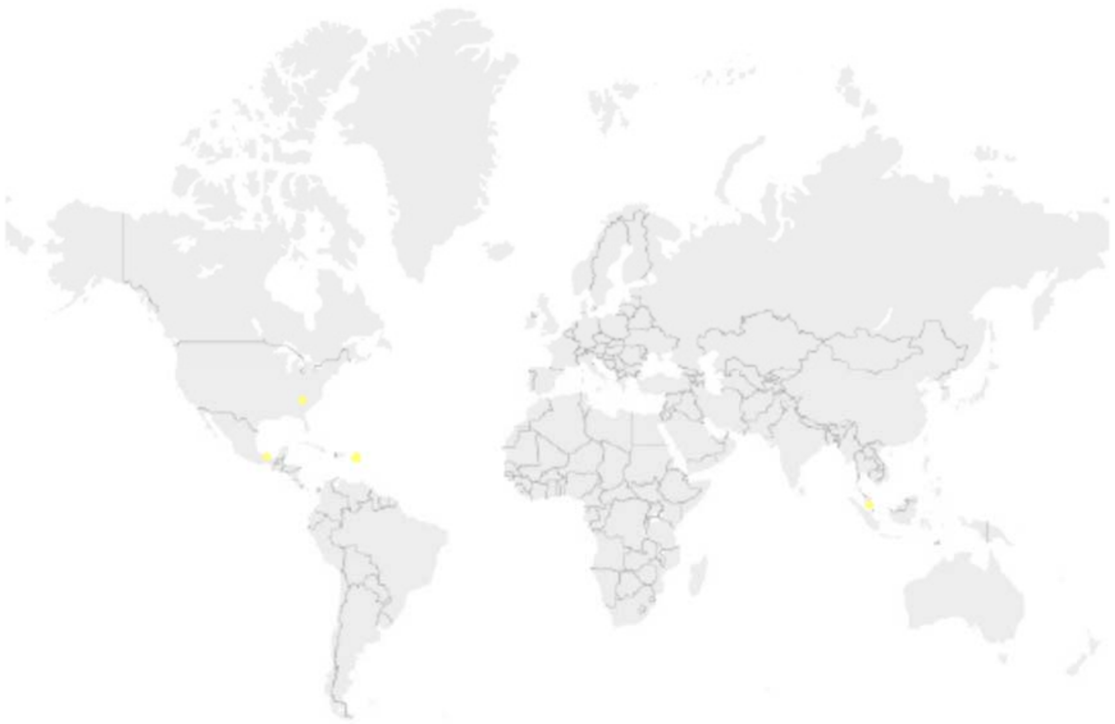

Codinaea paniculata

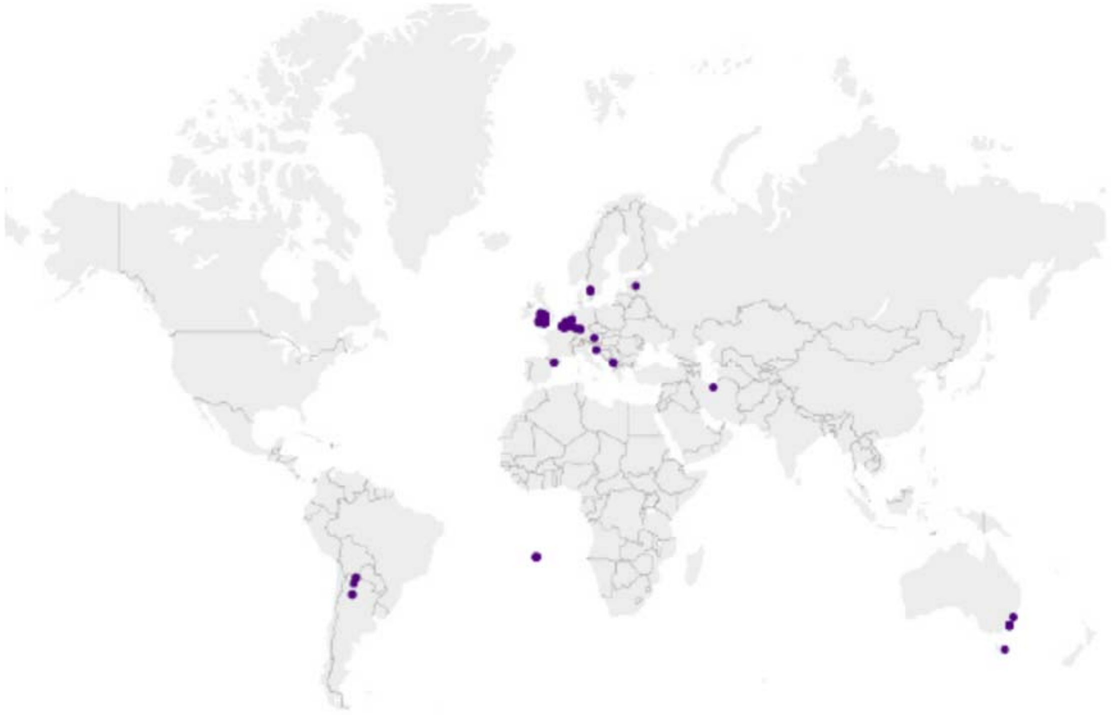

Codinaea phasma

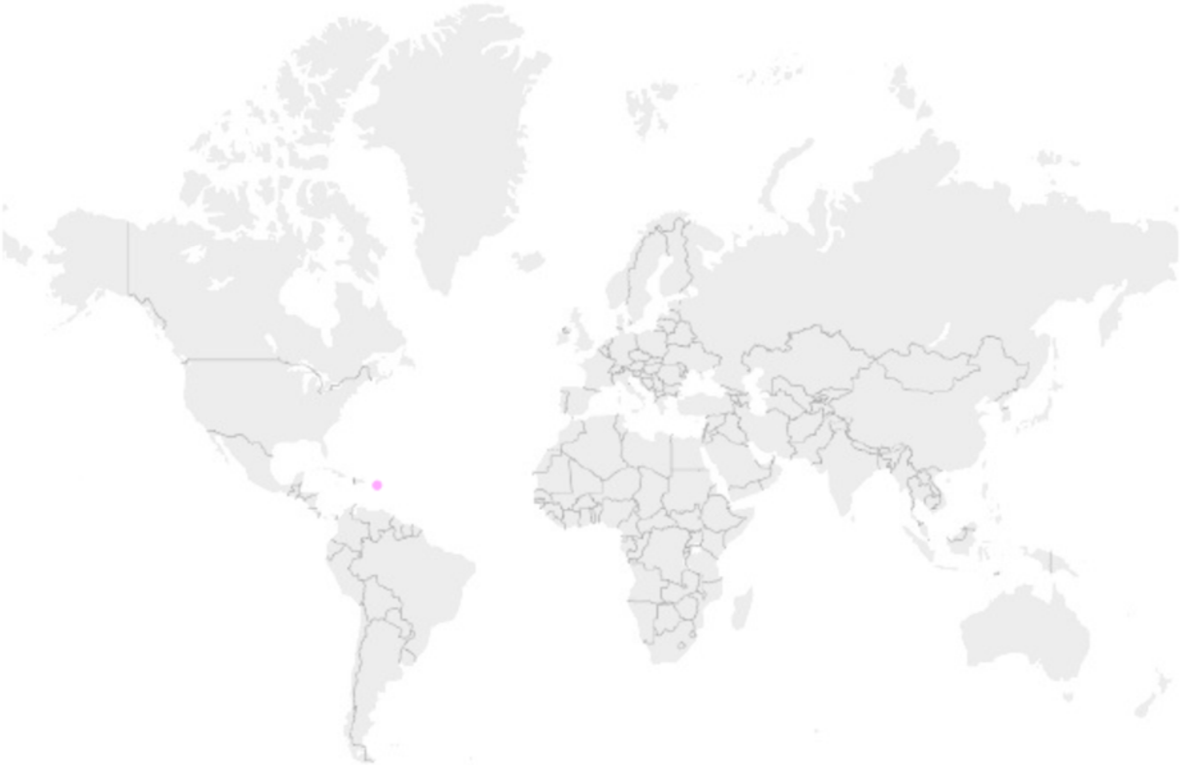

Codinaea siamensis

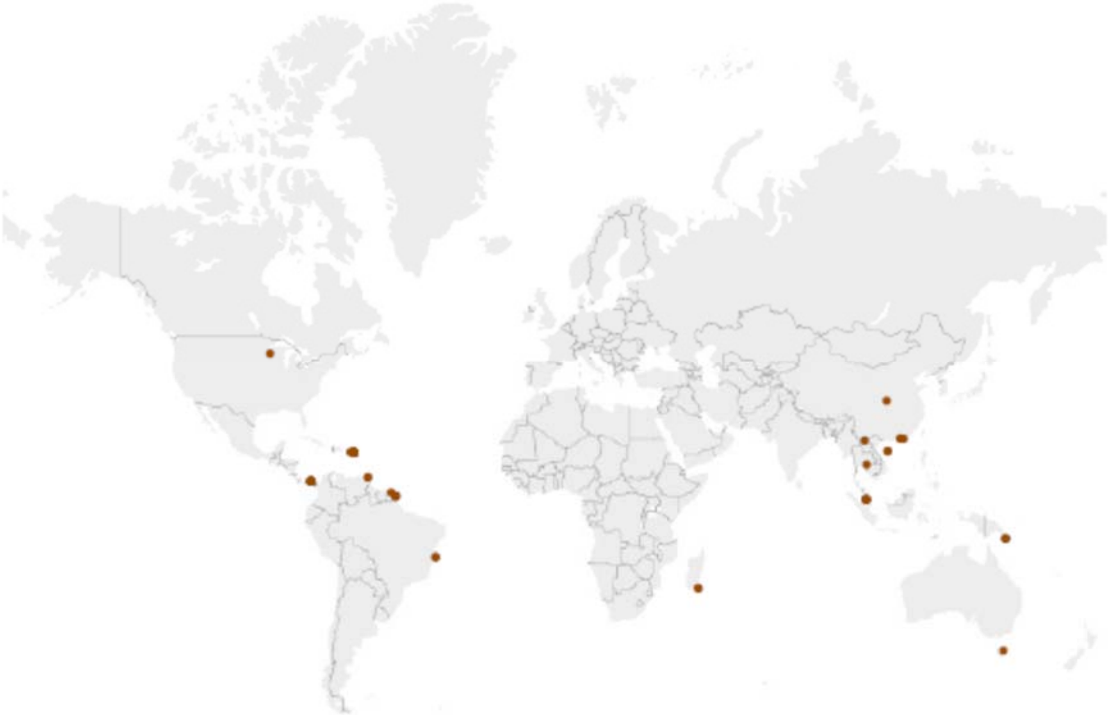

Codinaea terminalis

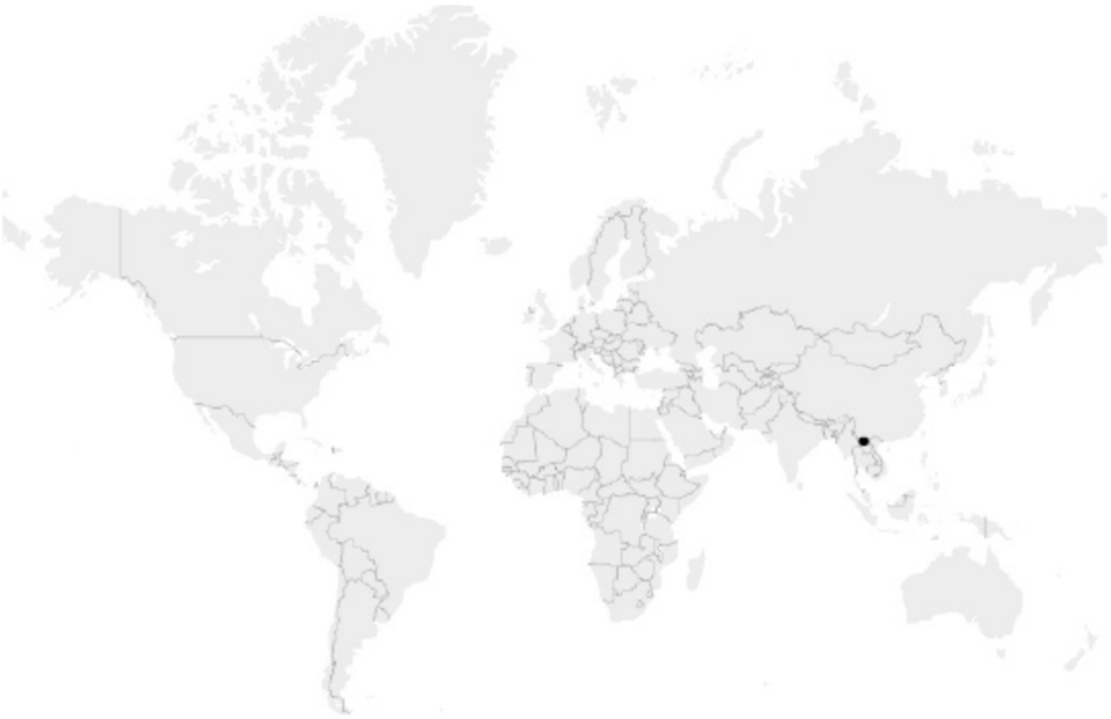

Codinaeella yunnanensis

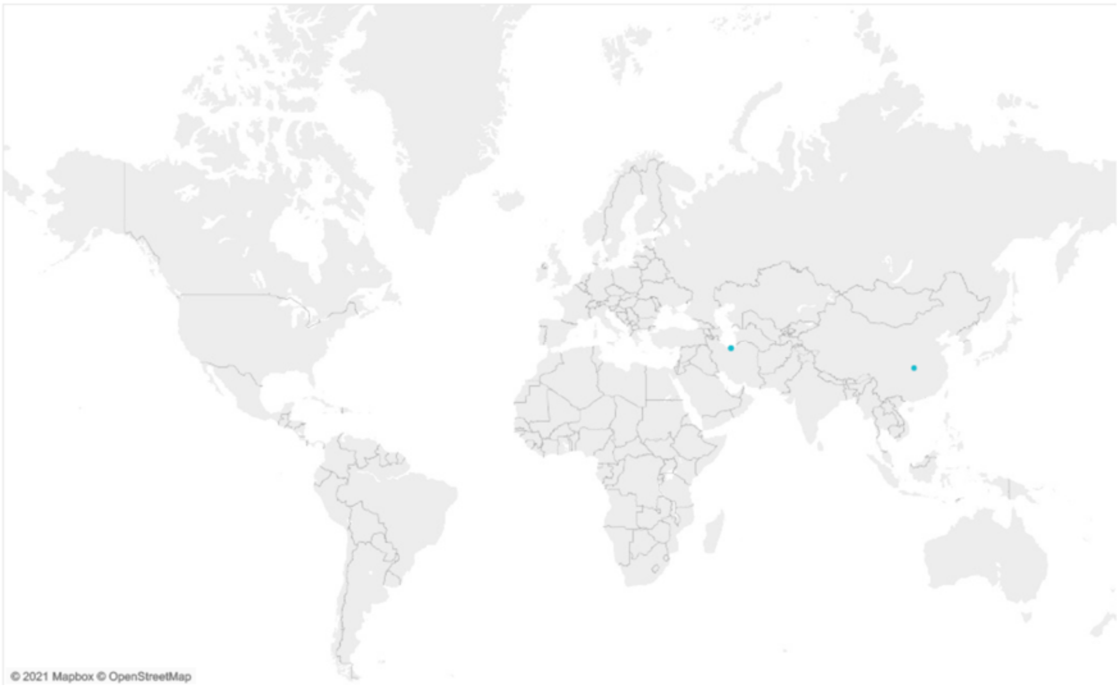

Codinaeella filamentosa

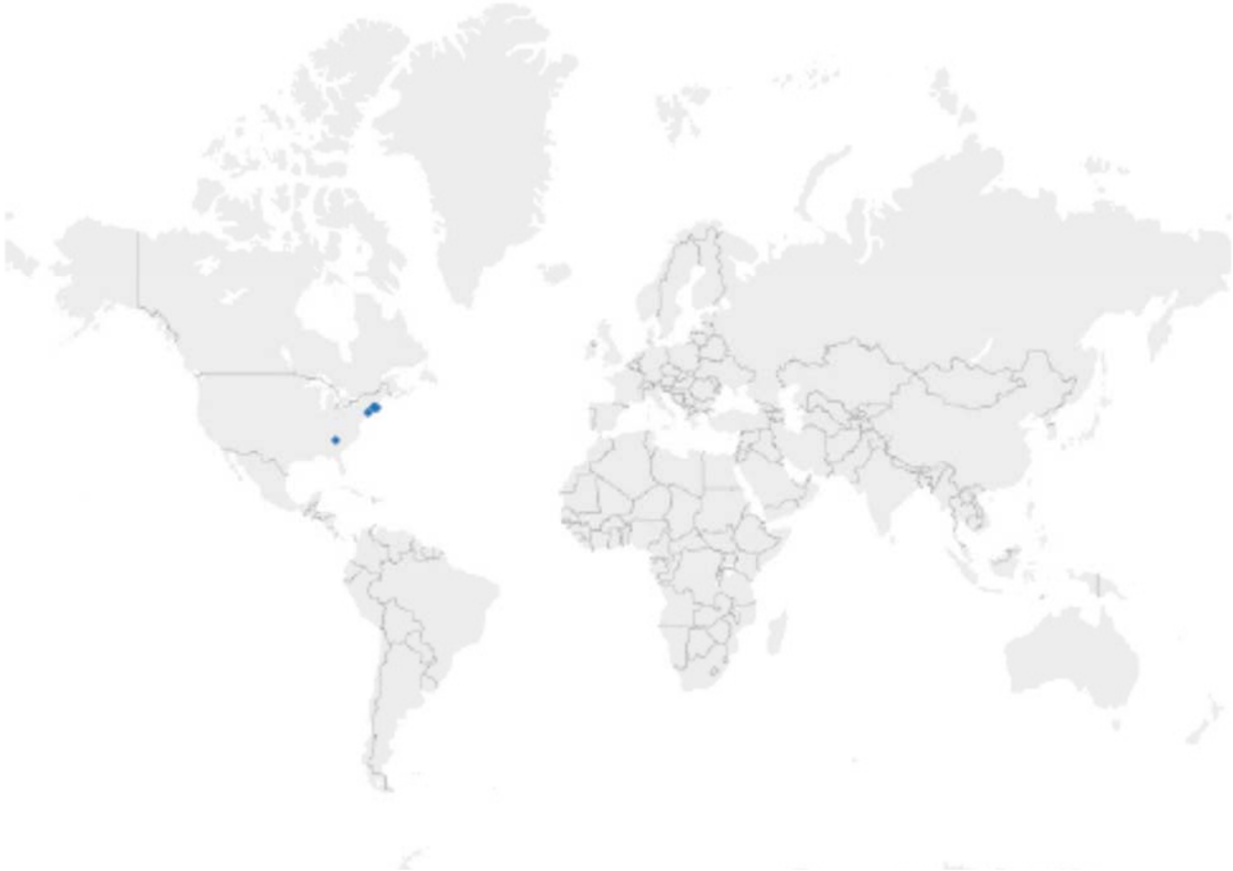

Codinaeella lambertiae

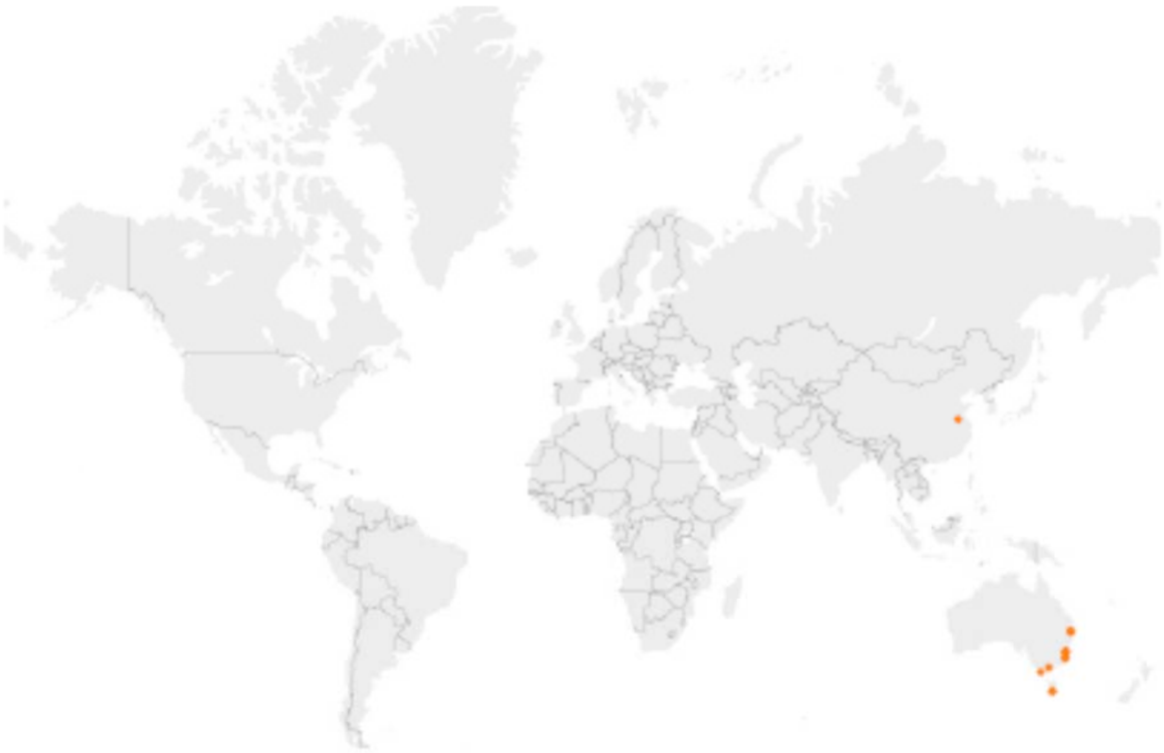

Codinaeella lutea

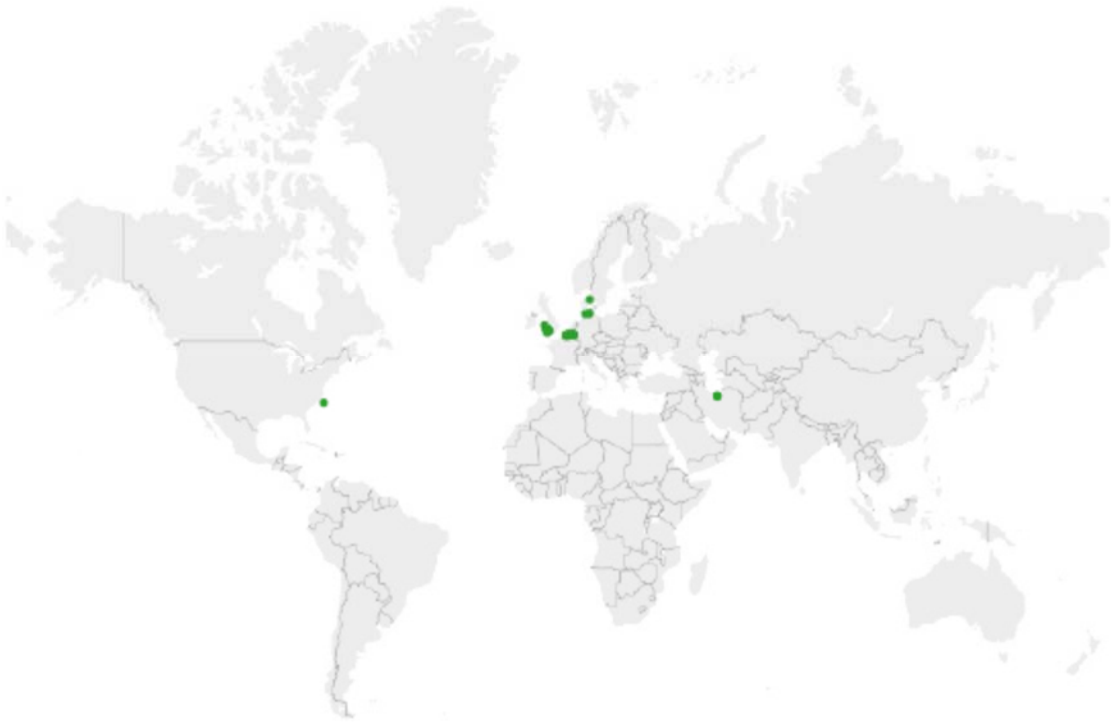

Codinaeella minuta

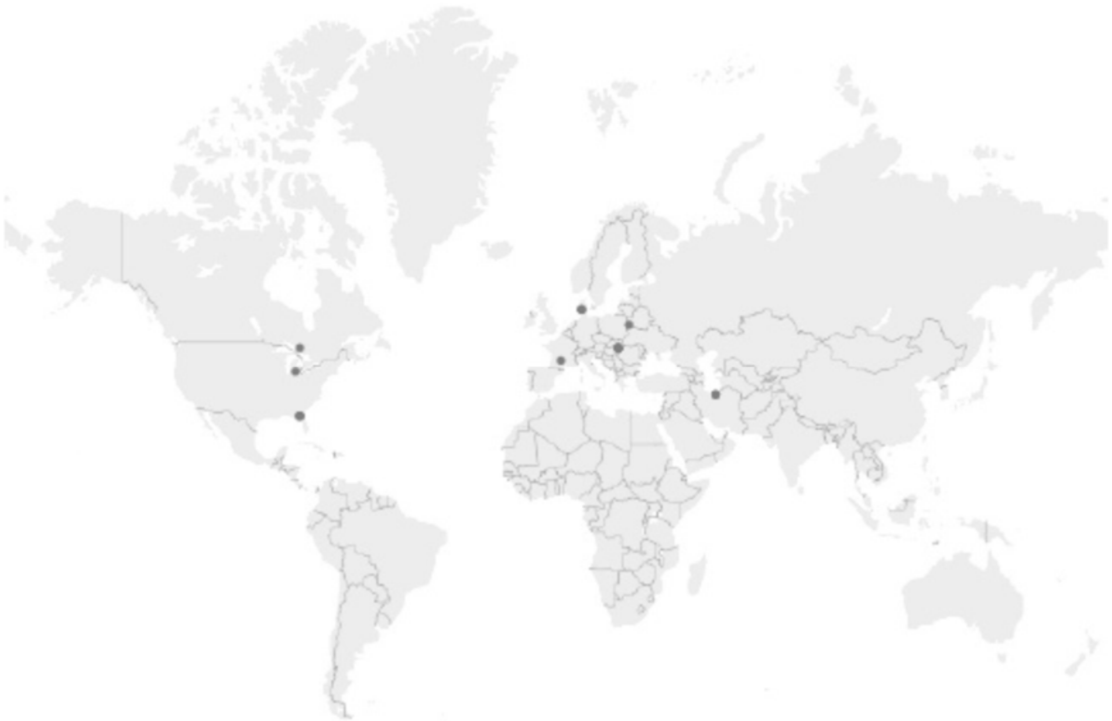

Codinaeella parvilobata

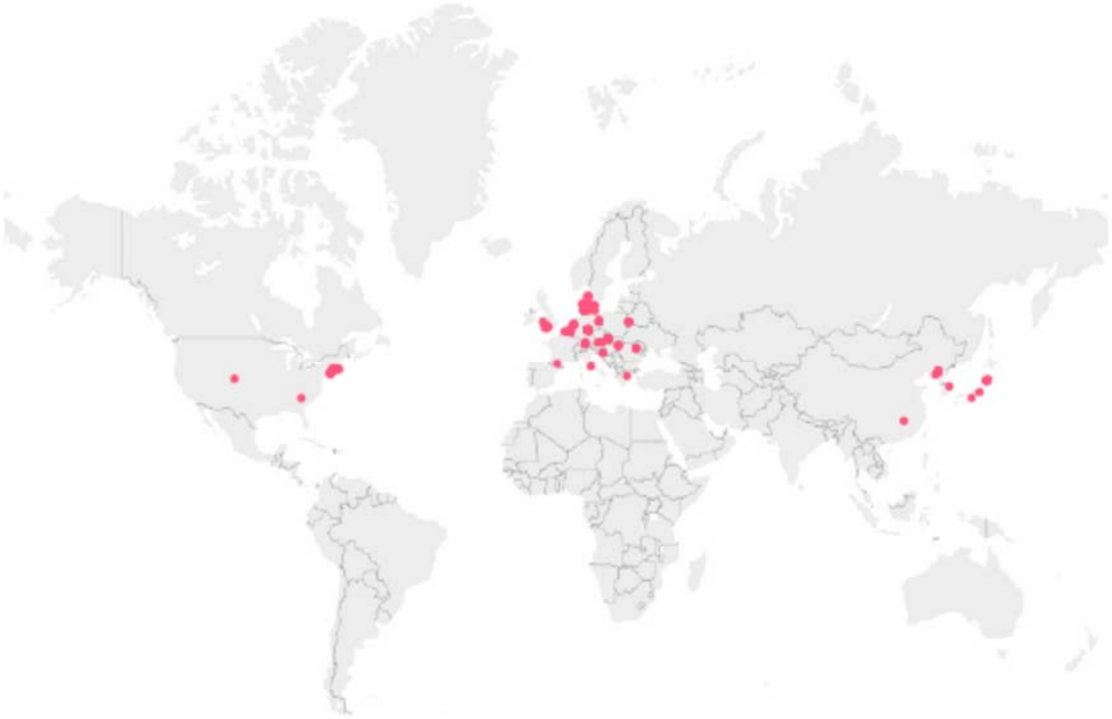

Stilbochaeta aquatica

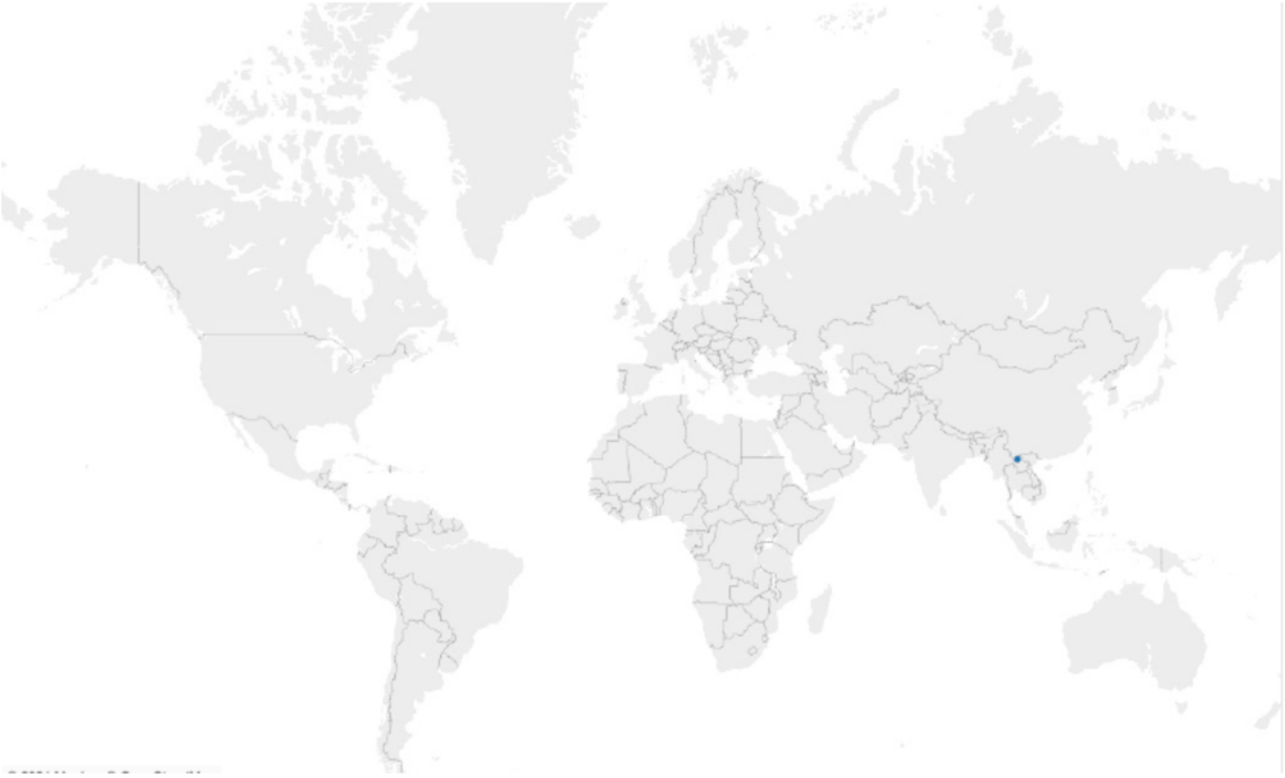

Stilbochaeta cangshanensis

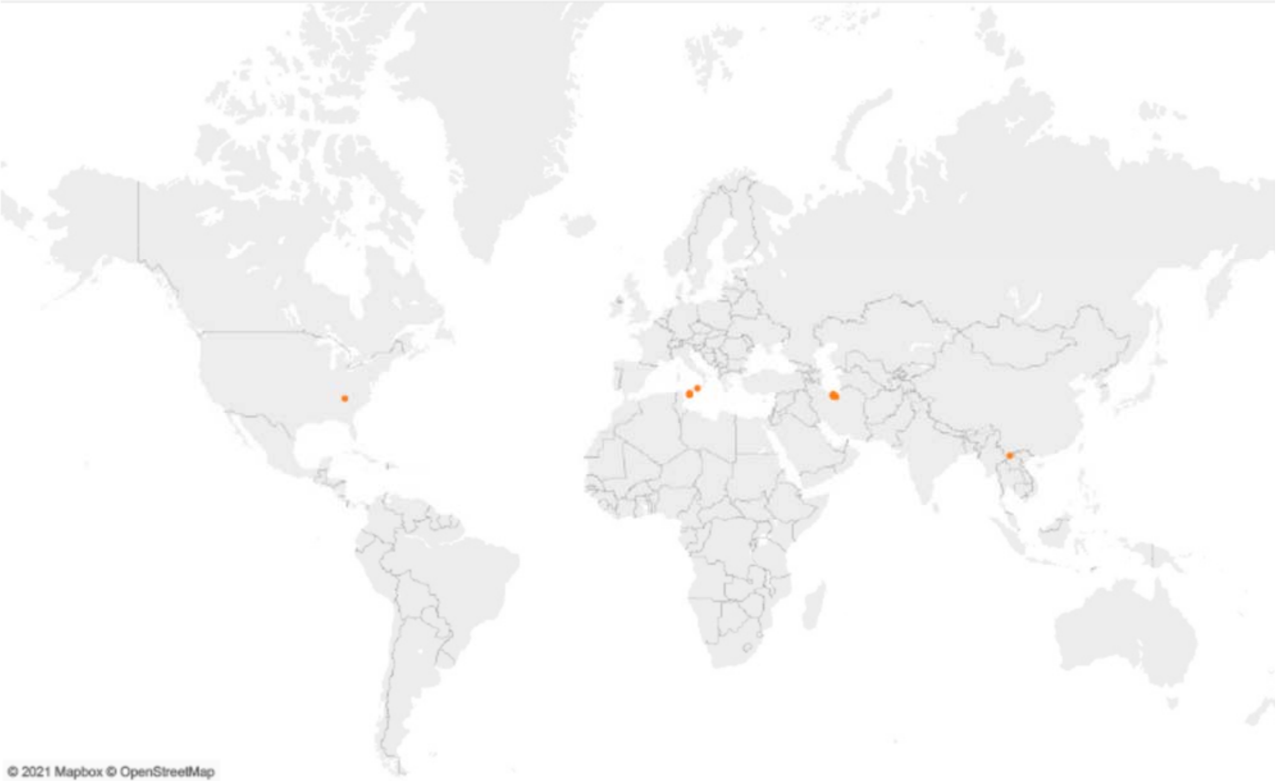

Stilbochaeta malaysiana

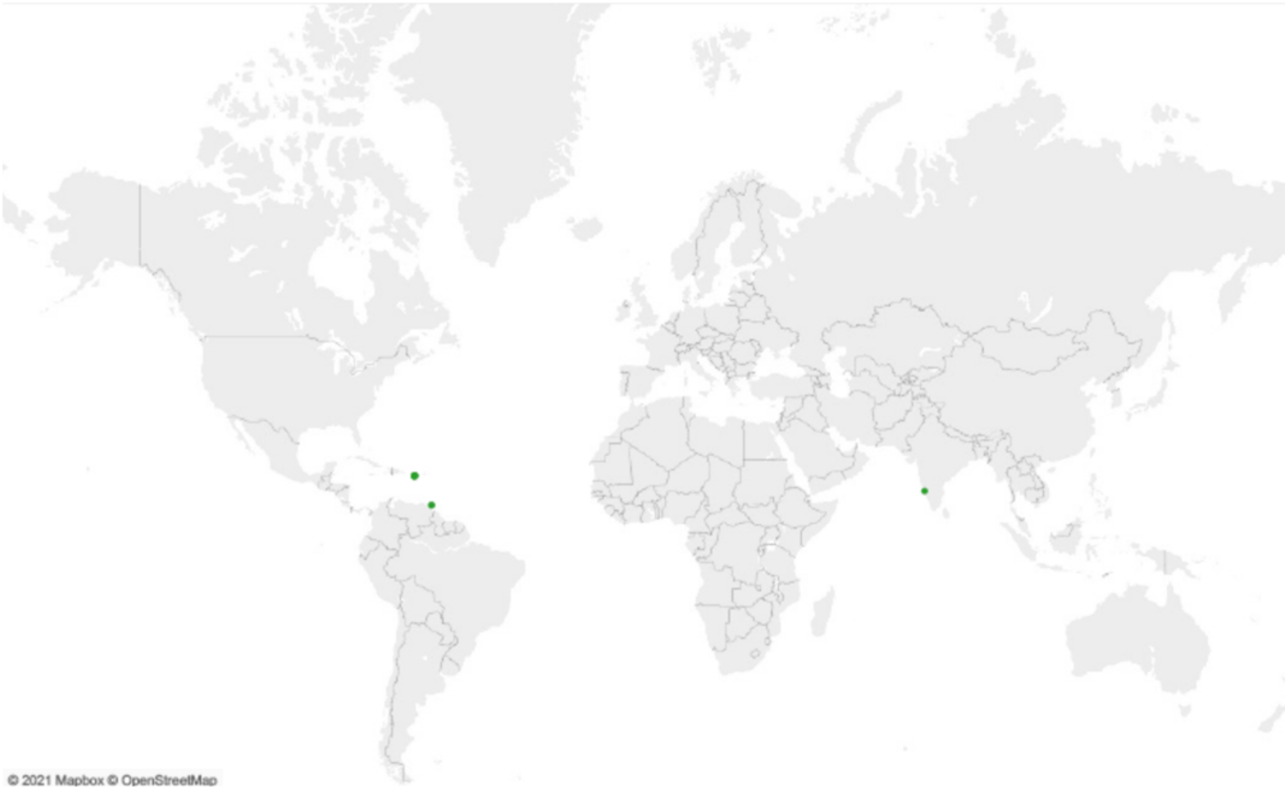

Stilbochaeta novae-guineensis

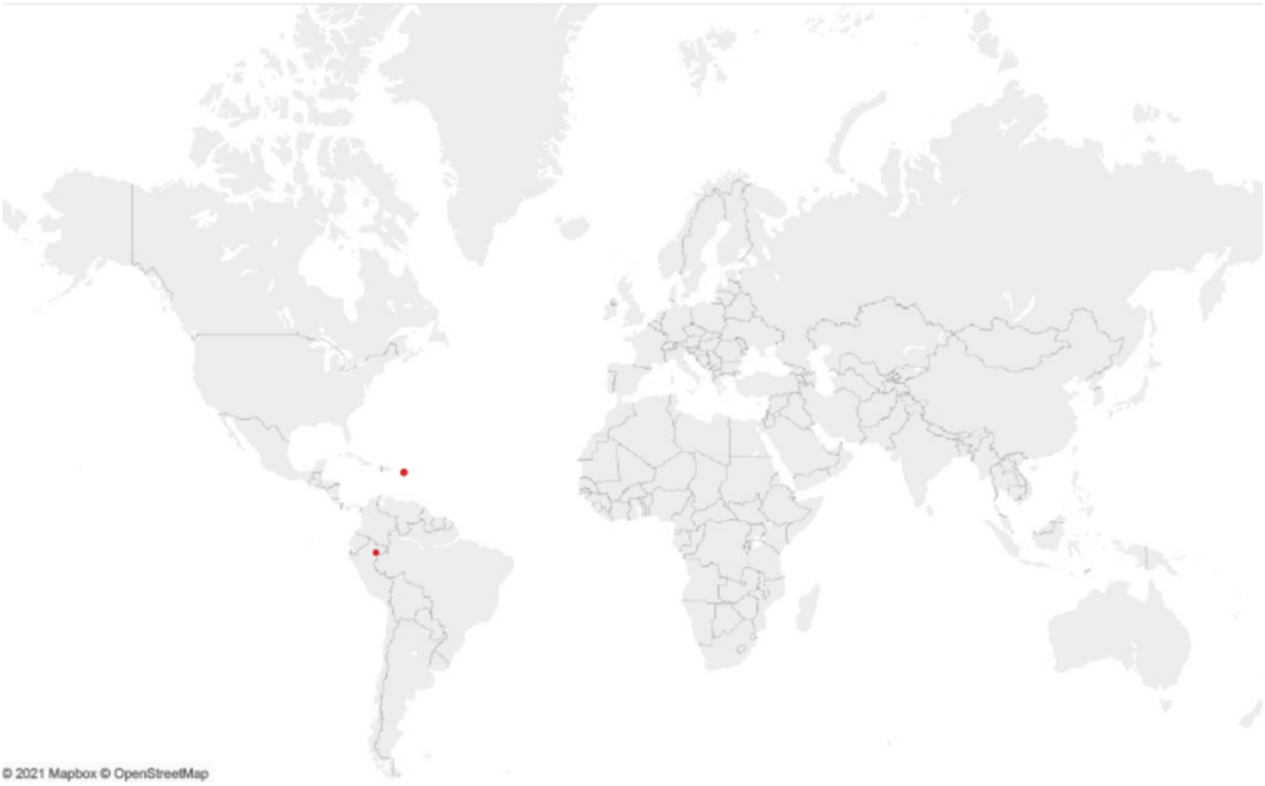

Stilbochaeta ramulosetulosa

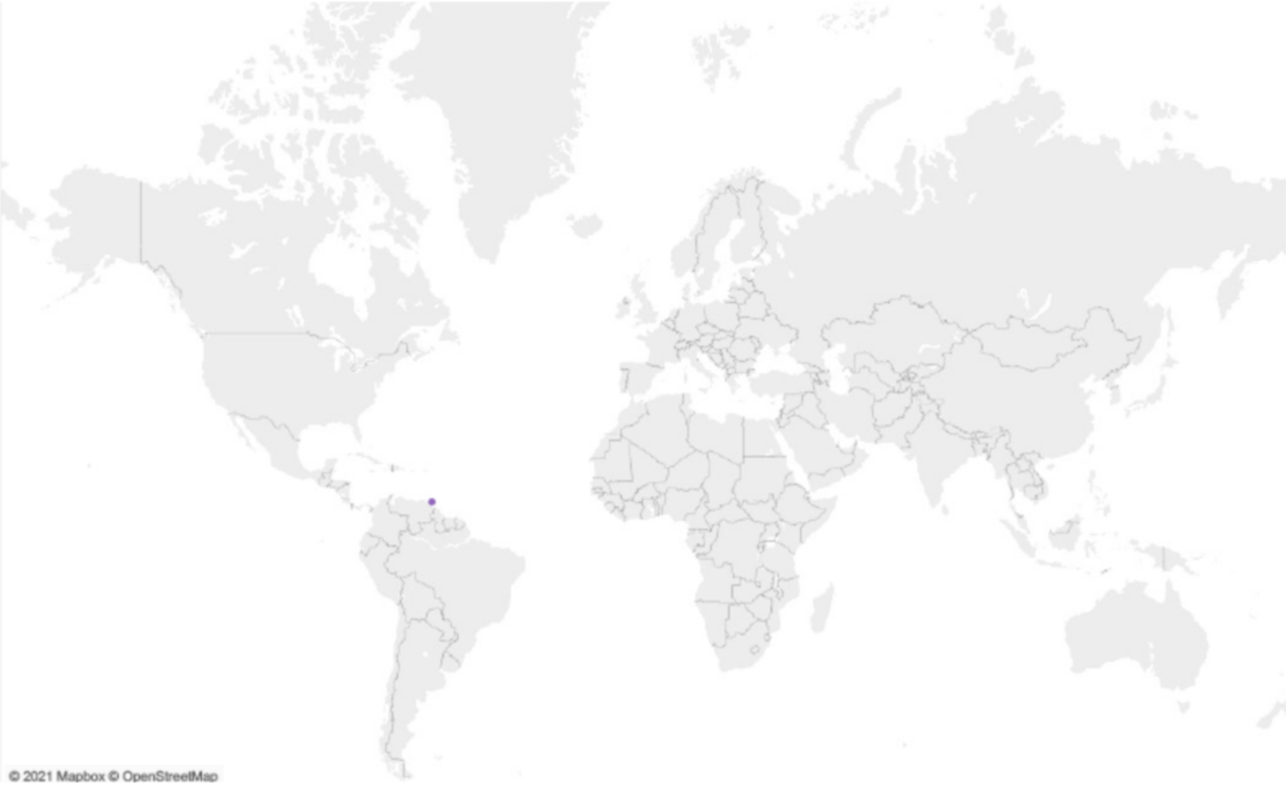

Stilbochaeta septata

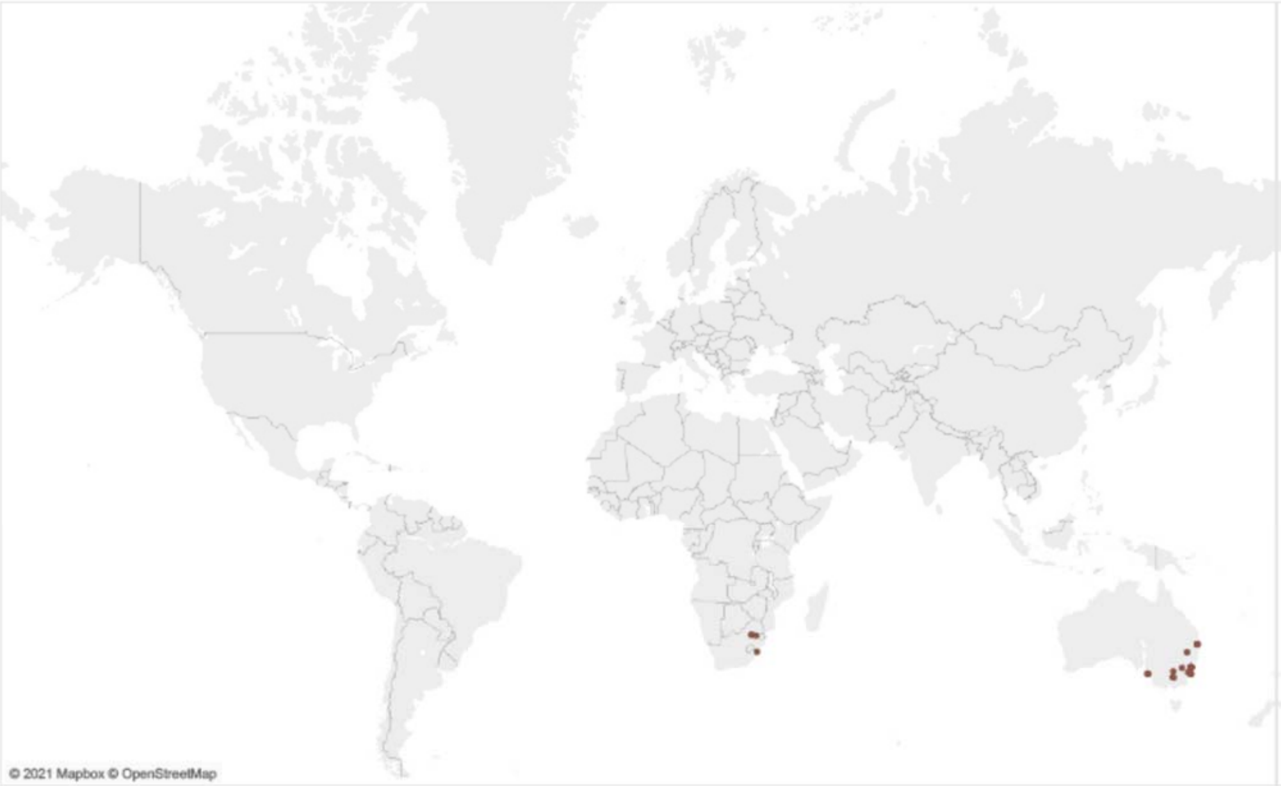

Supplement: Supplementary file 1 [file jof-07-01097-s001.zip › Suppl_Fig_S2.pdf]
