# Supplementary figures and images for: Phylogenetic Reassessment, Taxonomy, and Biogeography of Codinaea and Similar Fungi
Source: J Fungi (Basel). 2021 Dec 20;7(12):1097. doi: 10.3390/jof7121097 (PMC8704094; doi:10.3390/jof7121097)

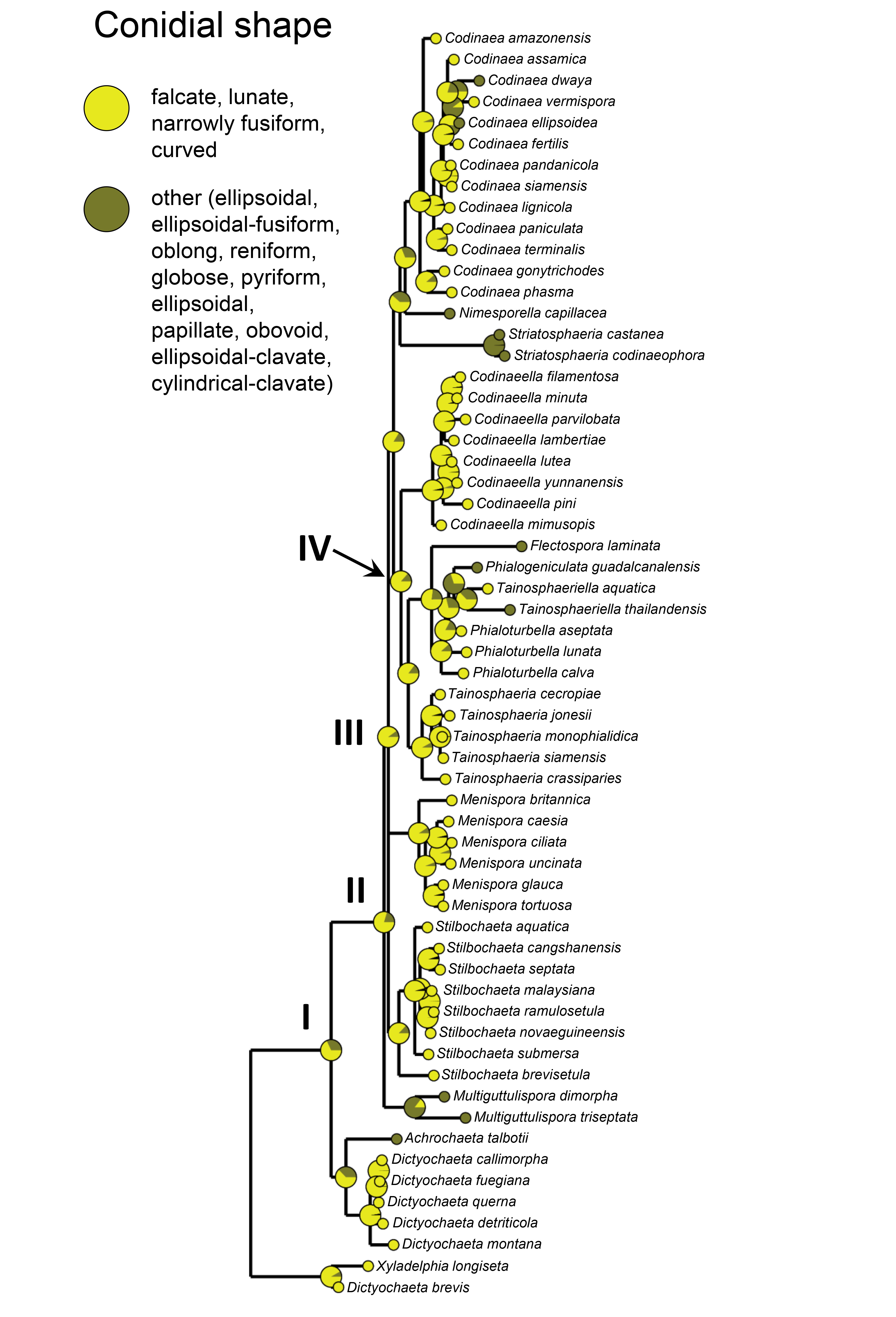

Supplement: Supplementary file 1 [file jof-07-01097-s001.zip › Suppl_Fig_S1.tif]

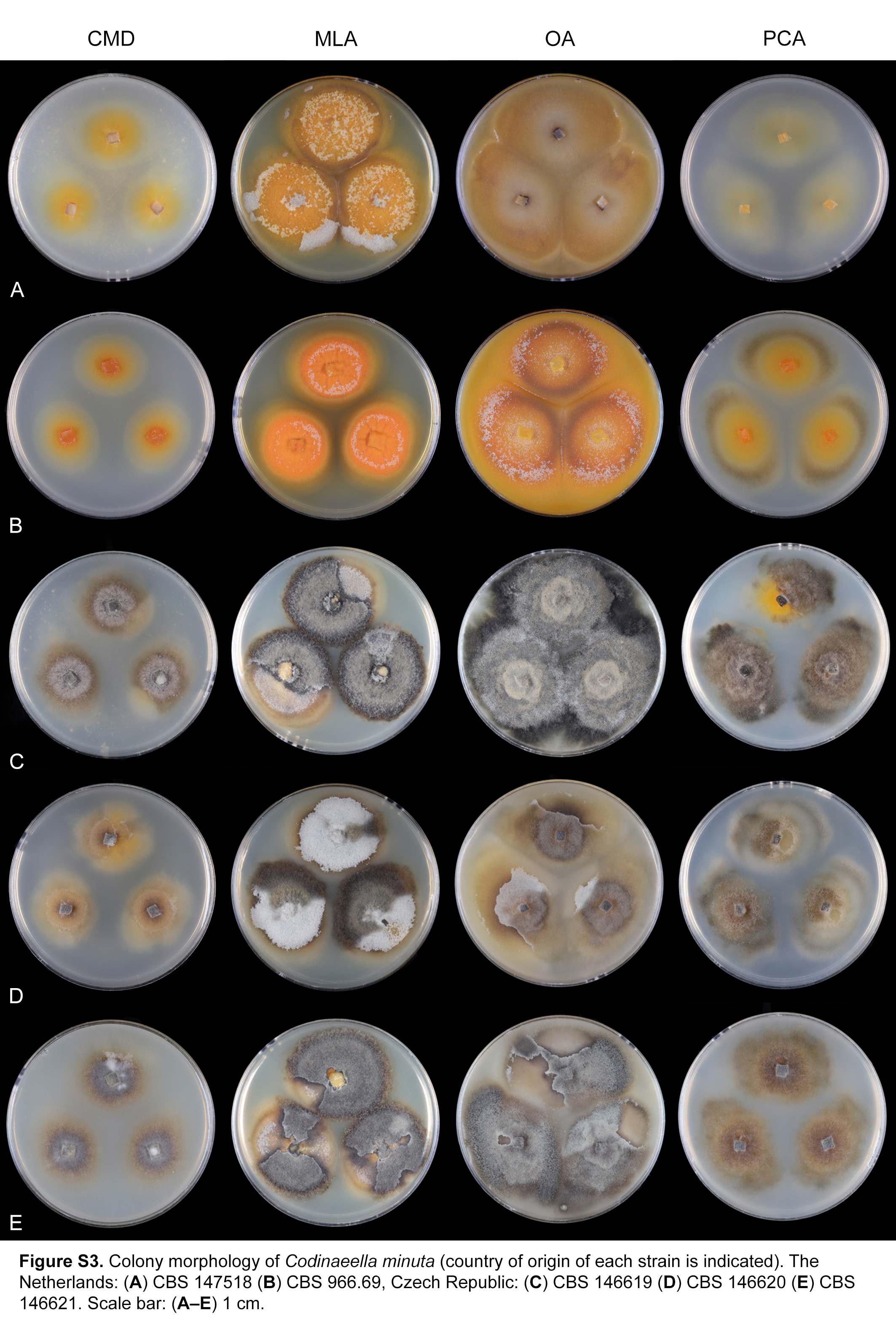

Supplement: Supplementary file 1 [file jof-07-01097-s001.zip › Suppl_Fig_S3.jpg]

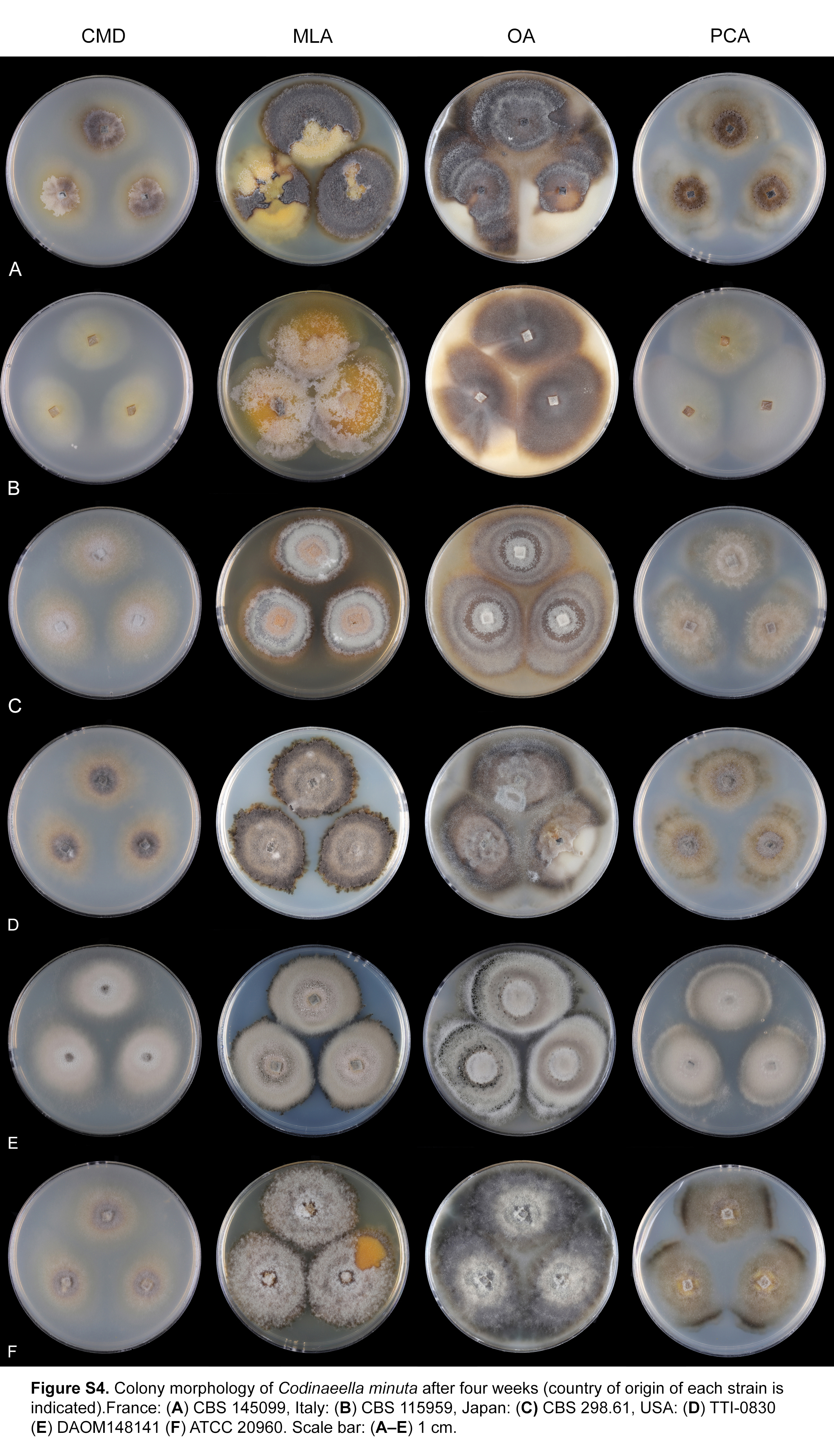

Supplement: Supplementary file 1 [file jof-07-01097-s001.zip › Suppl_Fig_S4.jpg]
